# Mass Production of Uniform Embryoid Bodies by Acoustic Standing Waves

**DOI:** 10.1002/smtd.202501283

**Published:** 2025-08-11

**Authors:** Johannes Hahn, Ebru Aksoy, Sarkawt Hamad, Christoph Kuckelkorn, Alejandro Gómez Montoya, Mira Ritter, Kurt Pfannkuche, Horst Fischer

**Affiliations:** ^1^ Department of Dental Materials and Biomaterials Research RWTH Aachen University Hospital Pauwelsstrasse 30 52074 Aachen Germany; ^2^ Centre for Physiology and Pathophysiology Institute for Neurophysiology University of Cologne Medical Faculty and University Hospital 50937 Cologne Germany; ^3^ Marga‐and‐Walter‐Boll Laboratory for Cardiac Tissue Engineering University of Cologne 50937 Cologne Germany; ^4^ Biology Department Faculty of Science Soran University Soran Kurdistan Region 44008 Iraq; ^5^ Department of Pediatric Cardiology University Hospital of Cologne 50937 Cologne Germany; ^6^ Center for Molecular Medicine (CMMC) 50931 Cologne Germany

**Keywords:** acoustic cell assembly, acoustic patterning, embryoid bodies, induced pluripotent stem cells, standing waves

## Abstract

Embryoid bodies (EBs) derived from human induced pluripotent stem cells (hiPSCs) provide the basis to obtain any type of organotypic cells and even complex multicellular organoids in 3D cell culture. However, traditional methods for generating EBs are labor‐intensive and often lack control over size uniformity. Here, this work presents a novel method for generating thousands of uniform EBs using acoustic standing waves in a single step. By utilizing piezoelectric ceramics to create controlled acoustic fields, this work achieves rapid, scaffold‐free aggregation of hiPSCs. This work demonstrates precise control of EB size by adjusting the ultrasound frequency and the cell seeding density, resulting in EB diameters ranging from 70 to 320 microns. This method enables the simultaneous formation of up to 28 000 EBs with more uniform size compared to those formed by the established ultra‐low‐attachment plate method. The generated EBs maintain pluripotency after 24 h of ultrasound exposure, as indicated by successful staining of key pluripotency markers. The EBs are successfully differentiated into functional, spontaneously contracting cardiomyocyte clusters. This novel method offers a low‐cost, scalable and efficient approach to produce a large amount of functional and uniform EBs serving as a starting material to produce cell clusters and organoids in suspension cultures or bioreactors.

## Graphical abstract

This innovative method for the generation of human induced pluripotent stem cell (hiPSC)‐derived embryoid bodies provides a scalable, flexible and cell‐friendly approach for the mass production of highly uniform cell clusters. It maintains the high cell viability and pluripotent state of hiPSCs while allowing successful differentiation into cardiomyocytes, achieving uniformity comparable to low‐attachment plates in a cost‐effective manner.

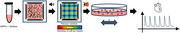

## 1 Introduction

In recent years, tissue engineering and regenerative medicine have made remarkable progress. Novel approaches of cell assembly and 3D tissue culture have contributed to the advancement of the field. A key player in regenerative medicine are human induced pluripotent stem cells (hiPSCs). This results from their unlimited self‐renewal capabilities and their ability to differentiate into any cell type across all three germ layers: ectoderm, endoderm, and mesoderm.^[^ ^1^ ^]^ Progenitor cells from the mesodermal layer are of decisive importance in cardiovascular tissue engineering, as most heart cells, including cardiomyocytes (CMs), develop from them.^[^ ^2^ ^]^ hiPSCs‐derived CMs (hiPSC‐CMs) display functional attributes of cardiac cells as contractility, expression of cardiac ion channel and intact response to adrenergic and muscarinic signaling.^[^ ^3^ ^]^ Cell aggregates present notable advantages over conventional 2D culture, particularly in drug screening and disease modeling.^[^ ^4^ ^]^ Moreover, 3D‐cell cultures more accurately replicate the natural cellular environment in terms of cellular signaling, cell‐matrix interaction and nutrient and oxygen diffusion.^[^ ^5^ ^]^ In addition, the local cell density is closer to the natural model.^[^ ^6^ ^]^ Large‐scale production of CMs from hiPSCs is therefore critical for a wide range of applications in research, medicine, and industry.^[^ ^7^ ^]^ It enables high‐throughput drug discovery and toxicity testing in human‐relevant models, and supports regenerative therapies, including the repair of damaged cardiac tissue, which requires hundreds of millions or even billions of cells.^[^ ^8^ ^]^ Furthermore, large‐scale production is essential for the bioengineering of cardiac patches or whole heart tissues and the generation of consistent and reproducible disease models.^[^ ^9^ ^]^ It also meets the growing demand from the pharmaceutical and biotechnology industries for standardized cell sources, facilitating cost reduction through scalability and ensuring sufficient supply for diverse experimental needs. Ensuring cell quality and functionality throughout the scaling process remains critical for all these applications.^[^ ^9^ ^]^

Many studies focus on aggregating hiPSCs for diverse applications, showcasing the broad potential of these cells.^[^ ^10^ ^]^ In particular, the formation of embryonic bodies (EBs) from hiPSCs itself is a key step in directing the differentiation of these cells into various lineages.^[^ ^11^ ^]^ However, traditional EB generation methods, such as spontaneous aggregation in non‐adhesive environments by gentle shaking or rocking, often result in significant heterogeneity, uncontrolled EB size, and reduced differentiation efficiency.^[^ ^12^ ^]^ Another traditional method for EB formation is the hanging drop culture, which can produce EBs of uniform size if the initial cell concentration per drop is carefully controlled. However, the hanging drop method is limited in scalability due to the small volume of each drop and requires a large number of drops to increase EB production, resulting in a significant labor workload, especially for non‐automated scaling.^[^ ^13^ ^]^ Additionally, reproducibility may be compromised due to pipetting‐induced delays between the preparation of the first and last hanging drops.^[^ ^14^ ^]^ To address these challenges, forced aggregation methods have emerged as an alternative approach. A prominent technique involves seeding a specific number of hiPSCs into ultra‐low attachment U‐ or V‐bottomed 96‐well plates, followed by centrifugation to promote uniform aggregation. This approach provides precise control over EB size, leading to more consistent differentiation results.^[^ ^15^ ^]^ However, the high cost of disposable microwell plates remains a barrier to large‐scale experiments, making these methods prohibitively expensive for many research laboratories.^[^ ^16^ ^]^ Since EB size is crucial for differentiation, it is important to develop techniques that can generate homogenous EBs in a size‐controlled manner.^[^ ^11^ ^]^ Physicochemical factors such as temperature, pH, and oxygen supply influence cell‐ cell, cell‐extracellular matrix, and cell‐soluble factor interactions. Since these factors can be interpreted as a function of the EB size.^[^ ^17^ ^]^ A potential strategy to reduce size variability in EB production, is the use of remote fields such as ultrasound and magnetic fields to augment controlled cell aggregation.^[^ ^18^, ^19^ ^]^ By using external force fields, it is possible to move cells within a surrounding medium.^[^ ^20^ ^]^ However, only acoustic fields can facilitate the movement of millions of cells in a label‐free manner.^[^ ^21^ ^]^ These are usually generated by ultrasound standing waves. This method allows the patterning of cells into defined arrays.^[^ ^22^ ^]^ The interference of ultrasound waves stemming from piezoelectric transducers can result in a patterned field of standing waves with higher and lower pressure regions inside of an ultrasound conducting medium. In case of cell manipulation, the predominant force is the acoustic radiation force.^[^ ^23^ ^]^ The general expression for the time‐averaged acoustic radiation force acting on a particle with volume V_p_ is the gradient of the acoustic potential U_rad_.^[^ ^24^ ^]^

(1) $$F_{rad}=-\nabla U_{rad}$$

(2) $$U_{rad}=V_{p}\;\left[f_{1}\frac{1}{2}\kappa_{m}\langle {\left|p_{0}\right|}^{2}\rangle -f_{2}\frac{3}{4}\rho_{m}\langle {\left|{\vec{v}}_{0}\right|}^{2}\rangle \right]$$

where p_0_ and v_0_ are the time‐averaged pressure and velocity fields, respectively. κ_m_ and ρ_m_ denote the compressibility and density of the medium, while κ_p_ and ρ_p_ represent the compressibility and density of the particles. The compressibility f_1_ and the density factor f_2_ are given by:

(3) $$f_{1}=1-\frac{1}{\gamma \beta^{2}};\;\;f_{2}=\frac{2\gamma -2}{2\gamma +1}\;$$

where β and γ are defined as the ratios of the speed of sound and the density, respectively:^[^ ^25^ ^]^

(4) $$\beta =\frac{c_{p}}{c_{m}};\;\gamma =\frac{\rho_{p}}{\rho_{m}}$$

These parameters determine whether particles migrate toward the locations of maximum acoustic potential (pressure antinodes) or toward locations of minimum acoustic potential (nodes) of the standing wave.^[^ ^26^ ^]^ This behavior can be predicted using the acoustic contrast factor Φ. If Φ is less than zero, particles move toward the nodes, otherwise, they move toward the antinodes. If Φ equals zero, the standing wave has no effect on the particle movement.^[^ ^27^ ^]^

(5) $$\Phi =\frac{5\gamma -2}{2\gamma +1}-\frac{1}{\gamma \beta^{2}}$$

The technique of ultrasound standing waves has been successfully used for the formation of aggregates, as it can force cells into a 3D structure in a controlled and efficient manner without damaging the cells.^[^ ^28^, ^29^, ^30^ ^]^ However, recent research has not yet been able to exploit the full potential of this method.

Wu et al.^[^ ^31^ ^]^ and Chen et al.^[^ ^32^ ^]^ were able to generate up to 12.000 spheroids in one run with a surface‐acoustic‐wave (SAW) based device. However, the cell aggregates were only loosely assembled and were prone to damage during transferal into a petri‐dish for further incubation time. Additionally, there were limits to the spheroid sizes that could be achieved using this method, and upscaling would be difficult due to the characteristics of SAW‐devices.^[^ ^31^, ^32^ ^]^ To address this issue bulk‐acoustic‐waves (BAW) can be used instead.^[^ ^33^ ^]^ In the cited study, a multi‐trap acoustic levitation setup was used to aggregate human mesenchymal stem cells (hMSCs) in levitation. The number of assemblies was limited to 30 spheroids due to the geometry of the polydimethylsiloxane (PDMS) device.^[^ ^30^ ^]^ Park et al. have successfully removed this limitation by using a larger device and creating more than just one spheroid per node. However, using this technique results in irregularities in size of the assemblies.^[^ ^34^ ^]^ Cai et al. presented the superimposition of two standing waves perpendicular to each other to hold cells at the pressure nodes and to allow them to mature there. In this case, cross‐linking hydrogels prevented the cells from moving apart after the ultrasound was switched off.^[^ ^35^ ^]^ By using a combination of standing waves in all three spatial directions and utilizing Gelatine‐Methacryloyl (GelMa) for cell immobilization, Miao et al.^[^ ^28^ ^]^ demonstrated the feasibility of enhancing spheroid production with acoustic fields. However, this method required the use of a hydrogel coupled with a quite extensive incubation time, spanning 3 days to attain a 90% efficiency rate in spheroid formation, and even 7 days for the development of dense spheroids.^[^ ^28^ ^]^ Apart from that, the use of animal‐derived products prevents a translation to potential clinical applications.^[^ ^36^ ^]^ Thus, the novel approach presented here might fill this critical gap. To date, no studies have investigated the generation of EBs using acoustic standing waves in a scaffold‐free approach.

The approach presented in this manuscript addresses the limitations described above by using constant acoustic forces acting from four sides, creating a scaffold of acting forces. This ensured a stable support structure and increases the effectiveness of the system throughout the complete duration of EB formation. Therefore, we used four piezoelectric ceramics with frequencies between 1 and 2.5 MHz glued to a customized polymethyl methacrylate (PMMA) part. The working frequencies were precisely determined using a newly implemented frequency analyzer method. This ensured fine‐tuning of the device´s resonance conditions, improving field stability and reproducibility. To counter the risk of heat accumulation due to the constant activation of the piezo elements, a customized cooling unit was integrated into the setup. This cooling system maintained the temperature below physiological thresholds (<37 °C) through the spheroid production process, maintaining high cell viability and functionality. The adjustable size of the spheroids ranged from 70 to 320 µm. The ultrasound is continuously activated throughout the entire spheroid production process, which can last up to 24 h. The effect of the constant acoustic forces allowed an unprecedented speed in the production of cell aggregates in the range of thousands per run. In addition, the proposed method achieves high uniformity in size and roundness and the EBs show high viability.

Together, these technical advances, optimized piezoelectric material selection, improved device design, precise frequency tuning, and the implementation of active cooling, represent a significant improvement over existing acoustic aggregation methods, providing scalable, reproducible, and clinically relevant EB production in a single, streamlined platform.

## 2 Results

### 2.1 Working Mechanism of Acoustic Cell Clustering

**Figure 1:**
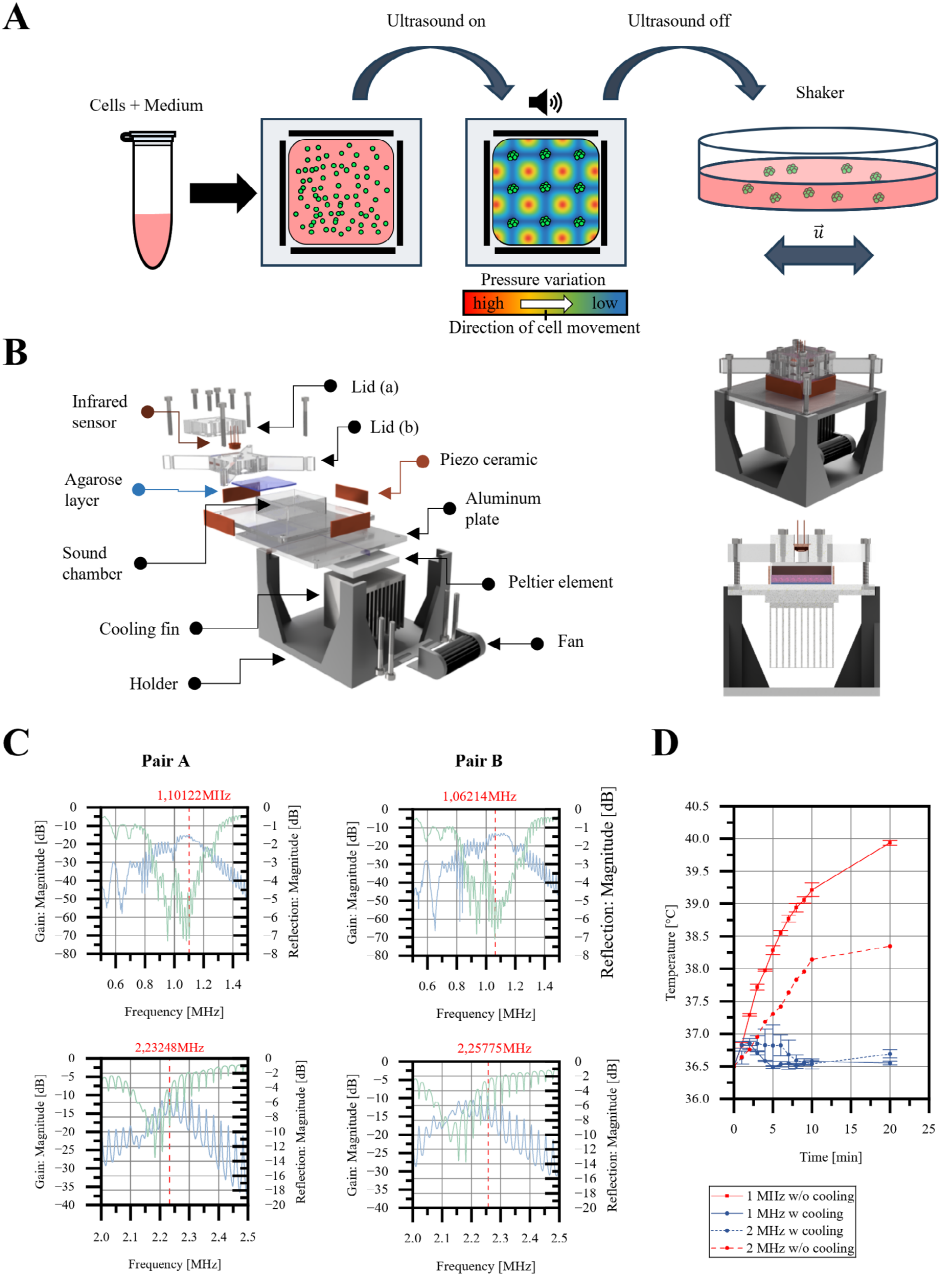
Working set‐up of acoustic cell clustering. A) Schematic of EB generation. B) Basic design of device for acoustic cell manipulation. C) Electrical characterization of piezoelectric‐ceramic‐pairs. D) Temperature input of piezoelectric ceramics w and w/o cooling system (n = 3). Data are presented as mean ± SD.

The ultrasound devices consisted of a PMMA sound chamber with four mounted piezoceramic elements (**Figure** **1**B). Ultrasound effectively immobilized the cells, leading to the formation of solid cell clusters, which were then transferred to petri dishes for further culture (Figure 1A). To ensure stable experimental conditions, the heat generated by the ceramics was efficiently dissipated via a cooling unit, preventing exposure to non‐physiological temperatures. For the 1 MHz device, the measured operating frequencies were 1.10 122 MHz and 1.0 6214 MHz, with a gain of 15 dB, while the 2 MHz setup operated at 2.23 248 MHz and 2.25 775 MHz, with a gain of 10 dB (Figure 1C). Although reflection values were comparable for both devices, the 2 MHz system exhibited periodic fluctuations, with reflection dropping as low as −14 dB but settling at ≈−6 dB at the operating point. Temperature monitoring showed that, without cooling, device operation caused temperatures to exceed 37 °C, peaking at 40 °C. However, with active cooling, the temperature remained consistently below 37 °C, preventing overheating (Figure 1D). This system successfully generated up to 28 000 EBs, depending on the frequency and size of the piezoelectric ceramic.

### 2.2 Influence of Frequency and Amplitude on Pressure Distribution

**Figure 2:**
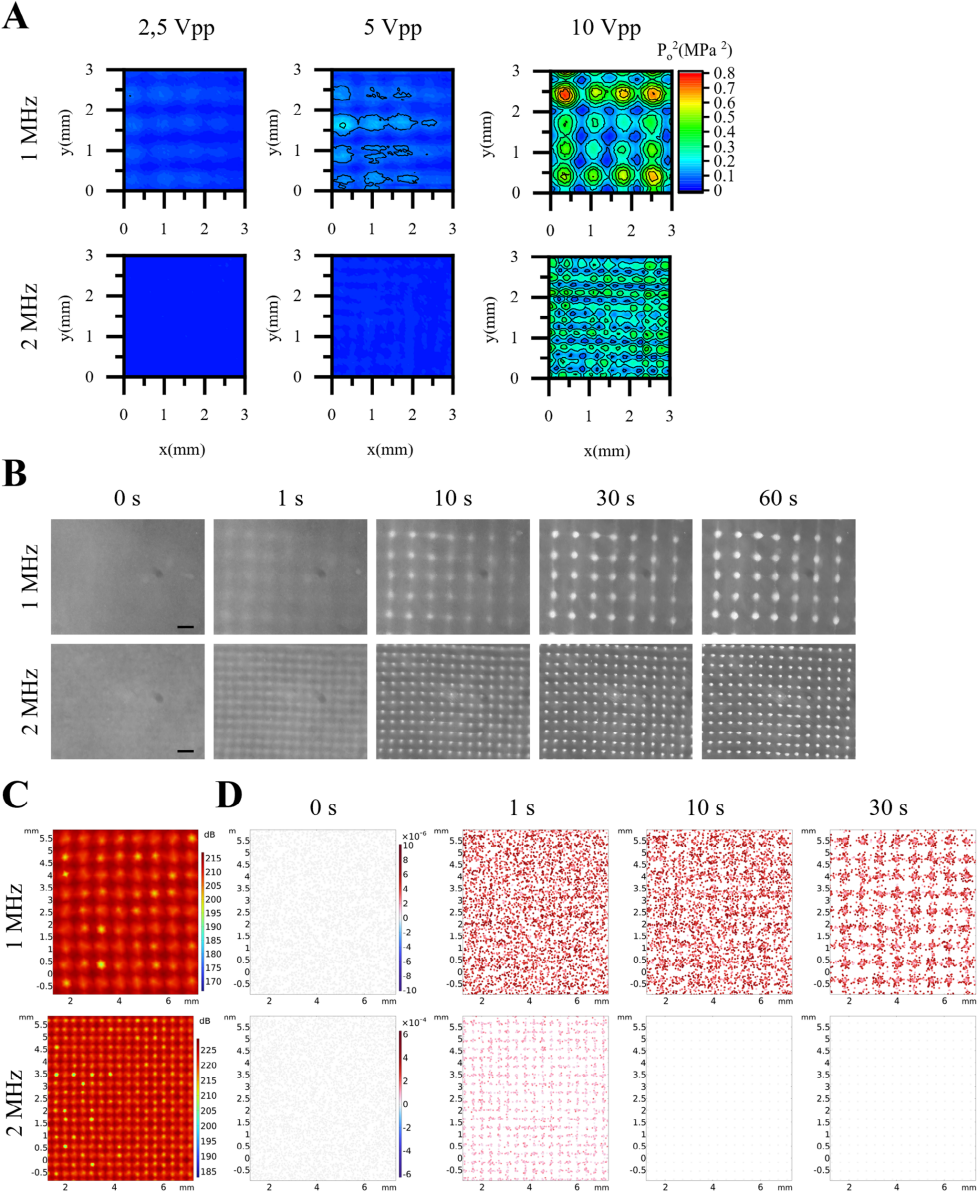
A) Measured pressure field of 1 and 2 MHz sound chambers at 2.5, 5, and 10 Vpp input. (Vpp: Volt‐peak‐to‐peak, that is, the voltage difference between the positive and negative peak values of an alternating voltage.) B) Formation of hiPSCs pattern during 60 s of ultrasound excitation at different frequencies. C) A sectional view showing the simulated sound pressure distribution for device 1 and device 2, modeled using COMSOL. D) Simulation of cell pattern formation and corresponding velocity distribution.

We investigated the influence of the frequency and amplitude of the ultrasound signal on the pressure field (**Figure** **2**A). A needle hydrophone was used to scan a small area of the chamber, which was maintained at a temperature of 36.5 °C to replicate realistic experimental conditions. The amplitude increase resulted in a corresponding rise in pressure regardless of the frequency. The number of nodal points was consistent with theoretical predictions, with twice as many nodal points detected in the pressure field of the 2 MHz device. The highest absolute pressure was recorded at 1 MHz, where a value of 0.79 MPa^2^ was measured, compared to 0.47 MPa^2^ at 2 MHz Figure 2B shows the formation of cell patterns during 60 s of ultrasound exposure at the center of the sound chambers. In both cases, cell movement is noticeable just 1 s after the ultrasound is activated. Nevertheless, the pattern in the 2 MHz device formed more rapidly and with greater precision. After 60s, the cell patterns became clearly visible. Notably, the distance between the nodes was smaller in the 2 MHz device compared to the 1 MHz device, while cell accumulation was more pronounced in the 1 MHz device. Figure 2C,D displays the simulated sound pressure distribution in the middle section of the sound chambers and its effect on cell movement. In both cases, the nodes of the standing wave are clearly visible, with the 2 MHz device showing a higher maximum sound pressure. Particle tracing revealed that cells in the 2 MHz device moved significantly faster, forming the ideal pattern in less than 10 s, while in the 1 MHz device, cells were still moving even after 30 s. Near the walls, both the sound pressure distribution and cell pattern showed variations, where elongated patterns also emerged. This phenomenon was corroborated by live imaging of cell pattern formation (Figure S1, Supporting Information).

### 2.3 Effect of Seeding Density and Ultrasound Exposure on EB Appearance

**Figure 3:**
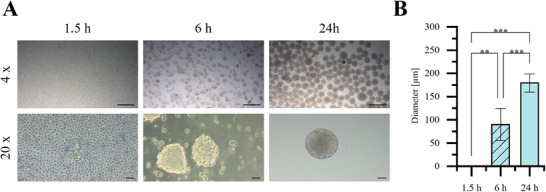
A) Appearance of EBs taken with different objective lenses. 4 x: Scale bar = 200 µm 20 x: Scale bar  =  50 µm. B) Distribution of EB diameter by increasing the duration of ultrasound exposure, 1.5 (no EBs have been found), 6 (n = 299), and 24 h (n = 136). Data are presented as mean ± SD (The *p*‐values were determined using one‐way ANOVA with a Tukey post hoc test, *p* < 0.05 (*), *p* < 0.01 (**), and *p* < 0.001 (***), “n.s.”: no significant difference.).

We have examined the influence of hiPSC seeding density and the duration of ultrasound exposure on the formation of EBs. Ultrasound exposure times of 1.5, 6, and 24 h were selected. The seeding densities used were 1, 3, 6, and 10 million cells in a respective amount of E8 medium. Ultrasound exposure exceeding 24 h and higher seeding densities than 10 million cells were not tested in this study due to the limited volume of medium in the acoustic chamber, which could potentially lead to nutrient deficiencies for the cells. The duration of ultrasound exposure significantly influenced the size and morphology of the EBs, as shown in **Figure** **3**. After 1.5 h of ultrasound exposure, few to no EBs are observable, though a few loosely attached clusters can be detected under higher magnification (Figure 3A). As the duration of ultrasound exposure increases, the formation of EBs becomes more pronounced. EBs formed after 24 h of continuous ultrasound are noticeably larger than those formed after 6 h of exposure. Additionally, the images reveal that the EBs in the 6‐h group are less round compared to those in the 24‐h group. The edges of the EBs are sharper and more defined in the 24‐h group, while in the 6‐h group, individual cell outlines are still visible.

**Figure 4:**
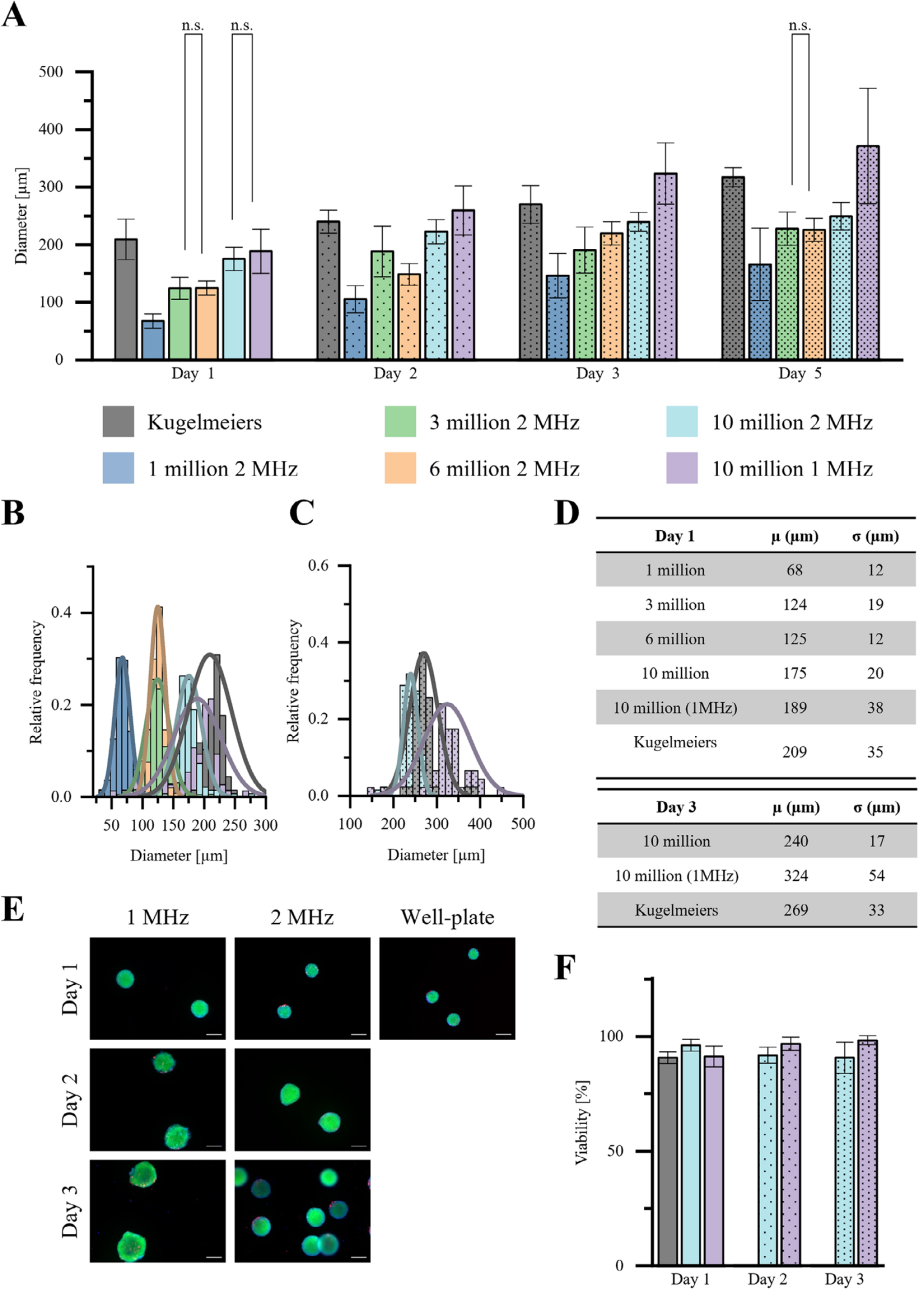
EB diameter and viability at different acoustic frequencies and cell seeding densities, compared with EBs produced using ultra‐low attachment plates from Kugelmeiers. A) Different cell seeding densities and the resulting EB diameters after 24 h of ultrasound exposure and up to 5 days on linear shaker (sample size is shown in Table S1, Supporting Information). Data are presented as mean ± SD (The *p*‐values were determined using one‐way ANOVA with a Tukey post hoc test. No annotation indicates that the p‐value was below *p* < 0.05, “n.s.”: no significant difference.). B) Distribution of EB diameters of all cell seeding densities after day 1 and C) of a cell seeding density of 10 million at 1 and 2 MHz on day 3. D) Expected value µ and standard deviation σ. E) Live/dead staining, with green cells indicating live cells and red‐stained cells representing dead cells. F) Live/dead ratio of EBs, generated either with acoustic standing waves with a seeding density of 10 million cells by 1 MHz and 2 MHz at different time points or by ultra‐low‐attachment well‐plates (n = 3). Data are presented as mean ± SD (The p‐values were determined using one‐way ANOVA with a Tukey post hoc test, *p* < 0.05 (*), *p* < 0.01 (**), and *p* < 0.001 (***). No annotation indicates that the difference was not statistically significant.). Scale bar = 200 µm.

The results of the analysis regarding the effect of cell seeding density were anticipated (**Figure** **4**). A higher seeding density resulted in a significantly larger EB diameter. Furthermore, the EBs continued to grow over time in culture, with diameters ranging from 70  to 320 µm. EBs with a seeding density of 1, 3, 6 and 10 million cells showed a standard deviation between 12 µm and 20 µm. EBs generated at 1 MHz had the highest standard deviation of 38 µm. EBs formed using ultra‐low‐attachment plates also exhibited a high standard deviation of 35 µm. Methods that resulted in higher standard deviations tended to produce the largest clusters. Using different frequencies offers the possibility of altering the size of the EBs, which is limited by the frequency. In this study, we used 1 and 2 MHz piezoelectric ceramics, meaning the distance between nodes in the 1 MHz chamber was twice that of the 2 MHz device. At a seeding density of 10 million cells, the 1 MHz acoustic device produced EBs with a diameter of 209 µm, while the 2 MHz chamber generated EBs with a diameter of 189 µm. Over time in culture on the shaker, a significantly higher size difference emerged between the EBs produced at the two frequencies. On day 2, the EBs in the 1 MHz setup had an average size of over 250 µm, while those produced at 2 MHz remained below 250 µm. By day 3, the 1 MHz EBs had grown to over 300 µm, whereas the 2 MHz EBs were still below 300 µm. On the day of differentiation, the standard deviation of the 1 MHz EBs was significantly higher than that of the 2 MHz EBs, as further confirmed by the size distribution related to frequency. The size and standard deviation of EBs produced using ultra‐low‐attachment plates fell between those of the two ultrasound applications. No negative impact of ultrasound at either 1z or 2 MHz on cell viability was observed by live/dead staining (Figure 4E). Over 90% of the cells remained alive both immediately after manipulation and throughout the culture period. The live/dead ratio was comparable to commercial and established EB production methods, with no significant differences detected (Figure 4F).

**Figure 5:**
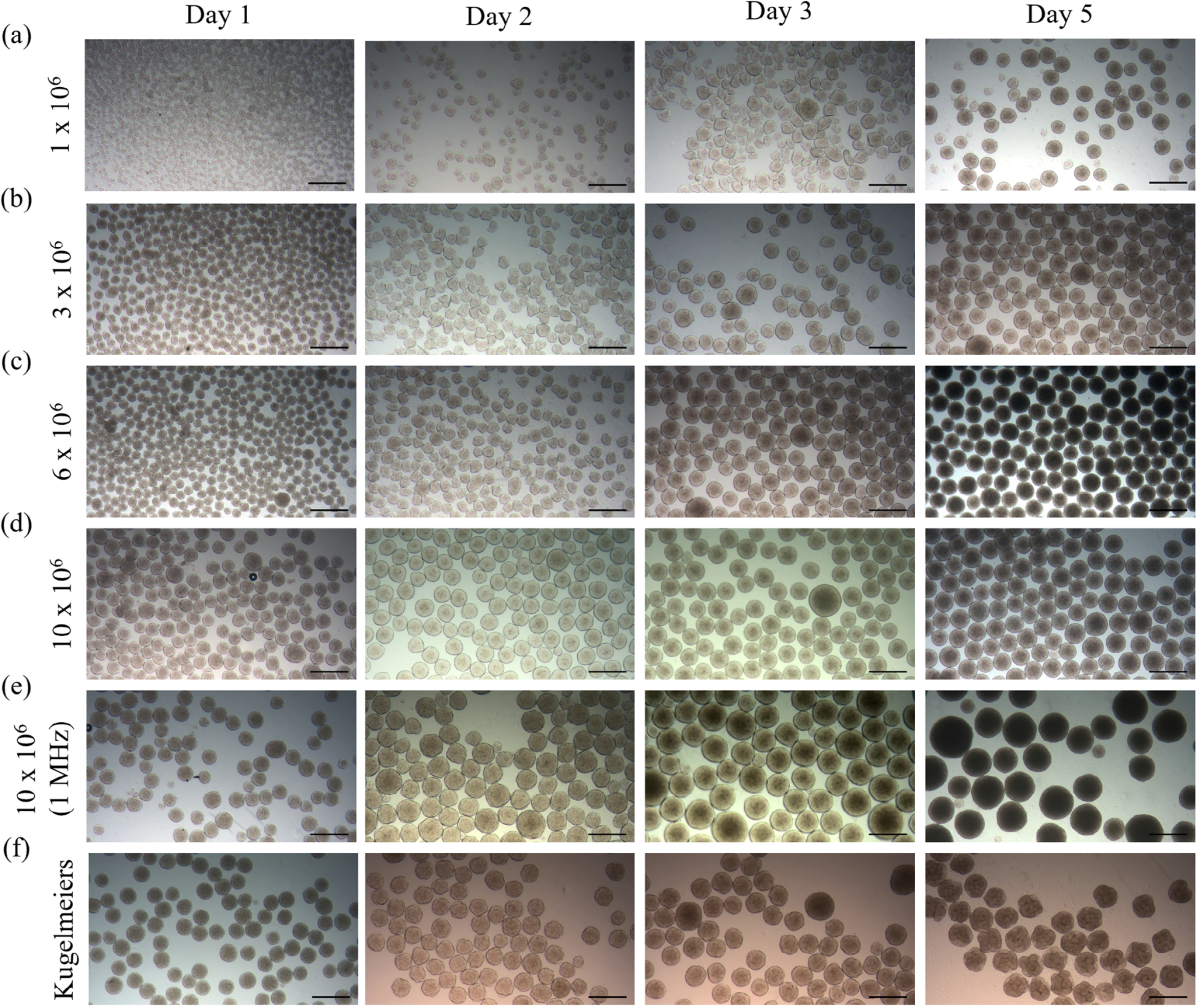
Appearance of EBs generated by acoustic standing waves during the time of culture. Scale bar  =  500 µm.

The appearance of EBs generated by acoustic forces and ultra‐low‐attachment plates are shown in **Figure** **5** over 3 days at seeding densities of 1, 3, 6, and 10 million cells. On day 1, EBs across all seeding densities displayed a uniform distribution with small, rounded structures. By day 2, size differences between the EBs became apparent, with the higher seeding densities of 6 million and 10 million cells (panels c, d, and e), along with the plate‐derived aggregates, showing noticeably larger clusters. The smaller EBs from the other ultrasound treatments exhibited a less round appearance, with rougher and more irregular outlines. On day 3, further growth and compaction of the EBs was observed, particularly in the 10 million cell group, where EBs reached the largest sizes, indicating a positive correlation between cell density and EB size. In panel (e), EBs generated with a 1 MHz acoustic frequency and a seeding density of 10 million cells were compared to those generated with 2 MHz (panels d). While growth patterns were similar, EBs in the 1 MHz group exhibited slightly larger diameters by days 2 and 3. A few EBs observed in these two groups were dramatically smaller than the rest, standing out significantly from the average size. On day 5, the EBs formed using ultra‐low‐attachment plates appeared distinct from those generated by acoustics. The center of these EBs displayed a structure that was notably less smooth, with a rougher texture compared to the rest of the cluster. Overall, EB size increased progressively from day 1 to day 3, with higher seeding densities and different acoustic frequencies leading to larger and more compact EBs, as observed in the figure.

### 2.4 Upscaling of Acoustic Chambers

**Figure 6:**
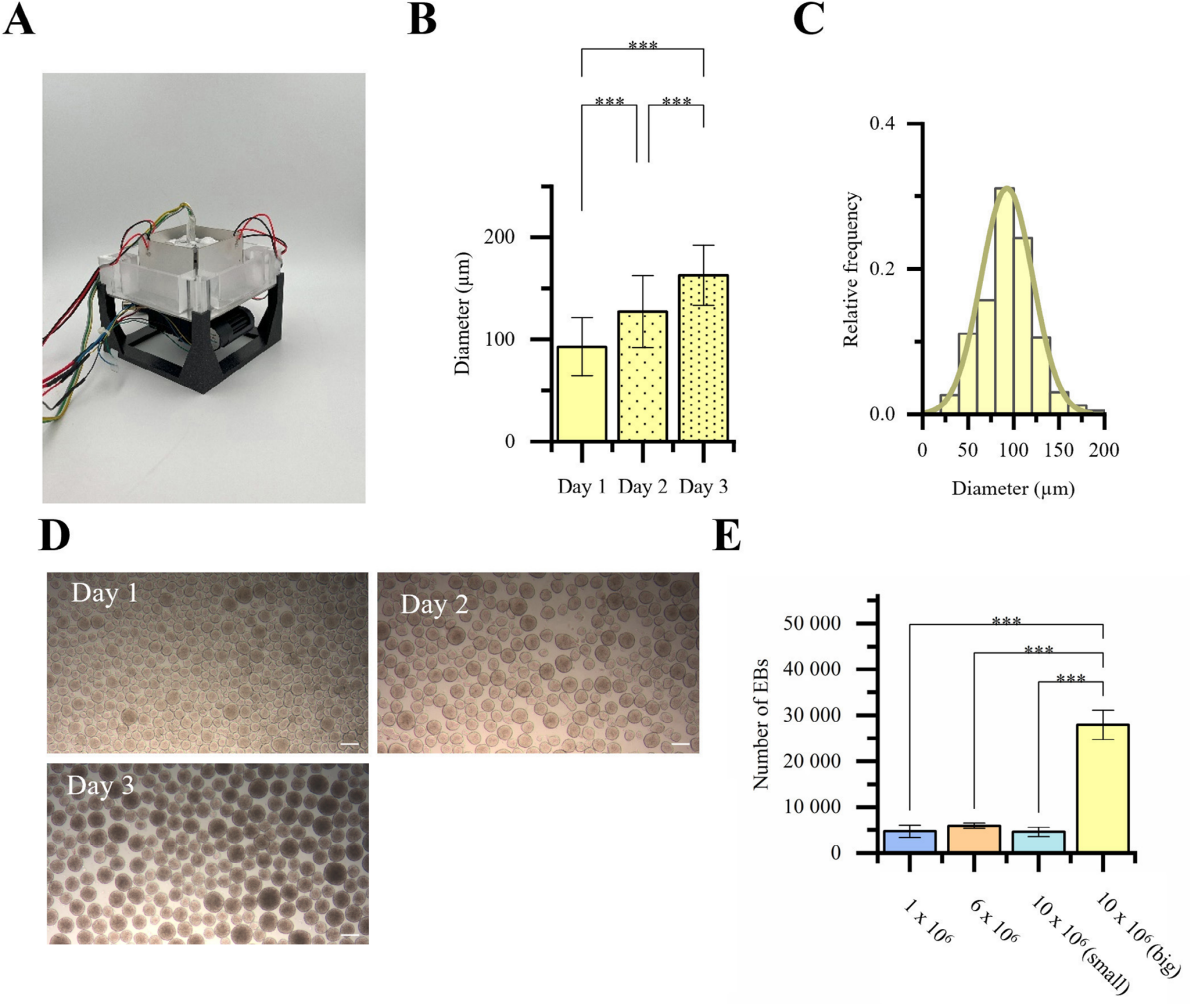
A) Acoustic device with four 60 × 40 × 1 mm^3^ piezoelectric ceramics. B) EB diameter on day 1 (n = 569), day 2 (n = 237) and day 3 (n = 177). Data are presented as mean ± SD (The *p*‐values were determined using one‐way ANOVA with a Tukey post hoc test, *p* < 0.05 (*), *p* < 0.01 (**), and *p* < 0.001 (***), “n.s.”: no significant difference.). and C) its distribution after 24 h of ultrasound exposure of 10 million cells at 2 MHz D) Appearance of EBs during the first three days of culture. E) EB yields after 24 h of ultrasound excitation at different cell seeding densities (n = 3). Data are presented as mean ± SD (The *p*‐values were determined using one‐way ANOVA with a Tukey post hoc test, *p* < 0.05 (*), *p* < 0.01 (**), and *p* < 0.001 (***), “n.s.”: no significant difference.). Scale bar = 200 µm.

The number of EBs that can be produced is limited by the number of nodal points within the acoustic field. The simplest way to increase this number is by using larger piezoelectric elements to generate a larger acoustic field (**Figure** **6**A). Doubling the width of the piezoelectric ceramics resulted in more than fivefold increase in EB yield. While the smaller devices produced ≈5000 EBs, the upscaled device achieved a yield of 28 000 EBs (Figure 6E). At a seeding density of 10 million cells, EBs formed in the larger device contained ≈500 hiPSCs each. In comparison, at seeding densities of 1 million, 6 million, and 10 million cells, the EBs formed in the smaller device consisted of ≈500, 1200, and 1500 cells, respectively. However, the upscaling also led to increased variability in EB morphology (Figure 6D). On day 1, the standard deviation was 28, compared to 20 in the smaller chamber at the same frequency (Figure 6C). Starting with a cell density of 10 million, EBs with an initial diameter of 92.7 µm were formed after 24 h of ultrasound exposure, growing to an average diameter of 163 µm by day 3 (Figure 6B). By increasing the dimensions of the piezoelectric ceramics, the yield of EBs generated within a 24‐h acoustic clustering cycle was significantly enhanced (Figure 6E). Specifically, the total EB count increased from 4615 in the smaller device to 27 917 in the upscaled system, both at a seeding density of 10 million cells. In contrast, variations in seeding densities within the smaller device did not result in statistically significant differences in EB yield. Despite comparable yields, the number of cells per EB differed across conditions. The lowest cell number per EB was observed at a seeding density of 1 million cells, with an average of 429 ± 144 cells. At 6 million cells, the average increased to 1016 ± 202 cells per EB. When using 10 million cells in the smaller device, EBs comprised 1689 ± 735 cells, whereas in the larger device, a lower average of 462 ± 231 cells per EB was recorded.

### 2.5 Immunofluorescence‐Microscopic Characterization of Pluripotent State of EBs

**Figure 7:**
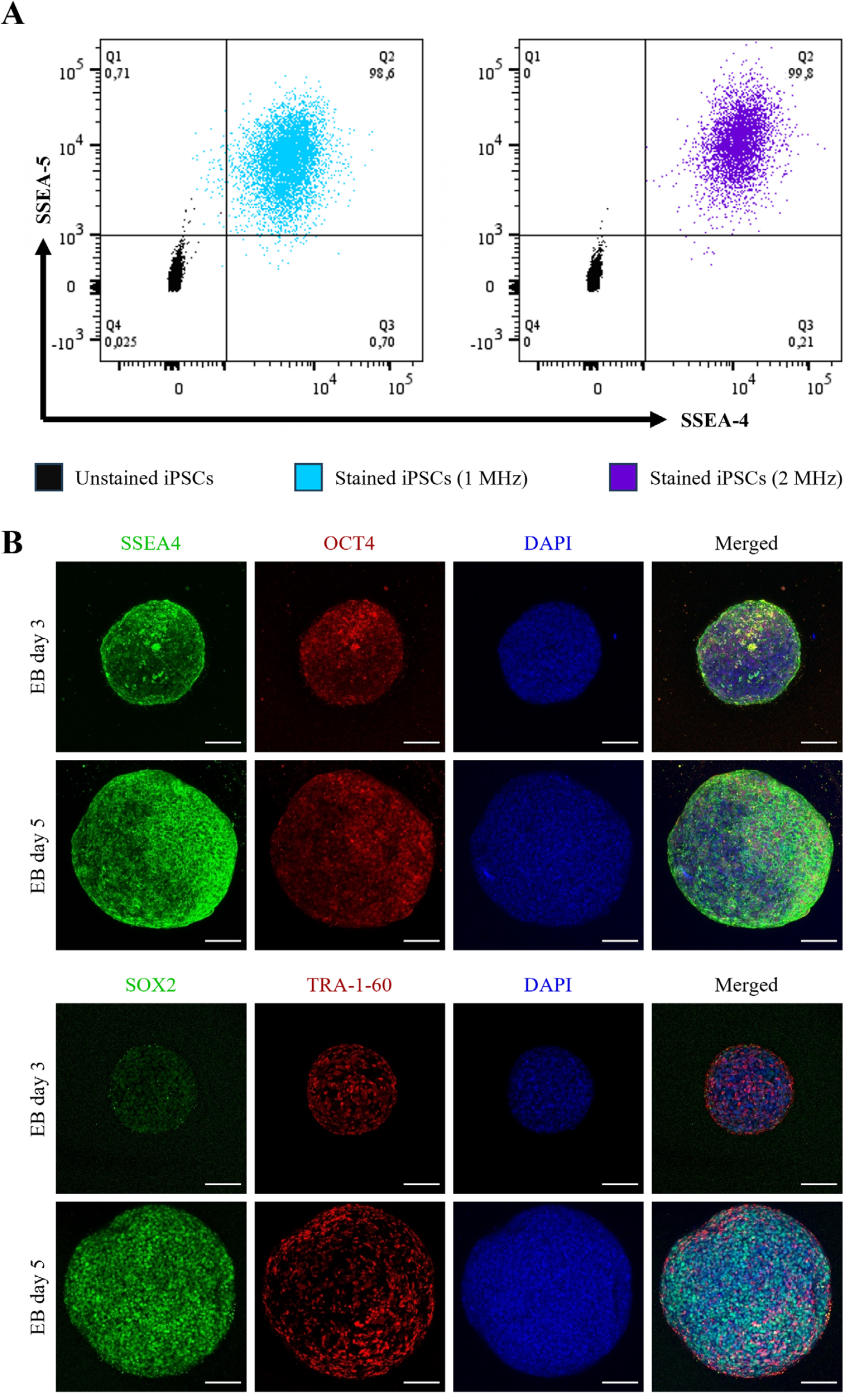
Proof of pluripotent state of ultrasound generated EBs. A) FC of the surface markers SSEA‐4 and SSEA‐5. B) Immunofluorescence staining of the pluripotency markers SSEA‐4, OCT4, SOX2, TRA‐1‐60 and DAPI. Scale bar = 100 µm.

Measurements of the surface markers SSEA‐4 and SSEA‐5 by flow cytometry (FC) showed frequency‐independent results, with more than 98% of the cells being double positive (**Figure** **7**). The control group showed 0.3% outliers, which were also detected as expressing the markers. Furthermore, EBs on day 3 and day 5 were labeled with antibodies against the genes SSEA4, OCT4, SOX2, and TRA‐1‐60, which are known to be highly expressed in iPSCs. Both the EBs on day 3 and day 5 showed expression of these genes. No significant differences were observed between the 2 days, except for the difference in EB size.

### 2.6 Differentiation of Ultrasound Generated EBs

**Figure 8:**
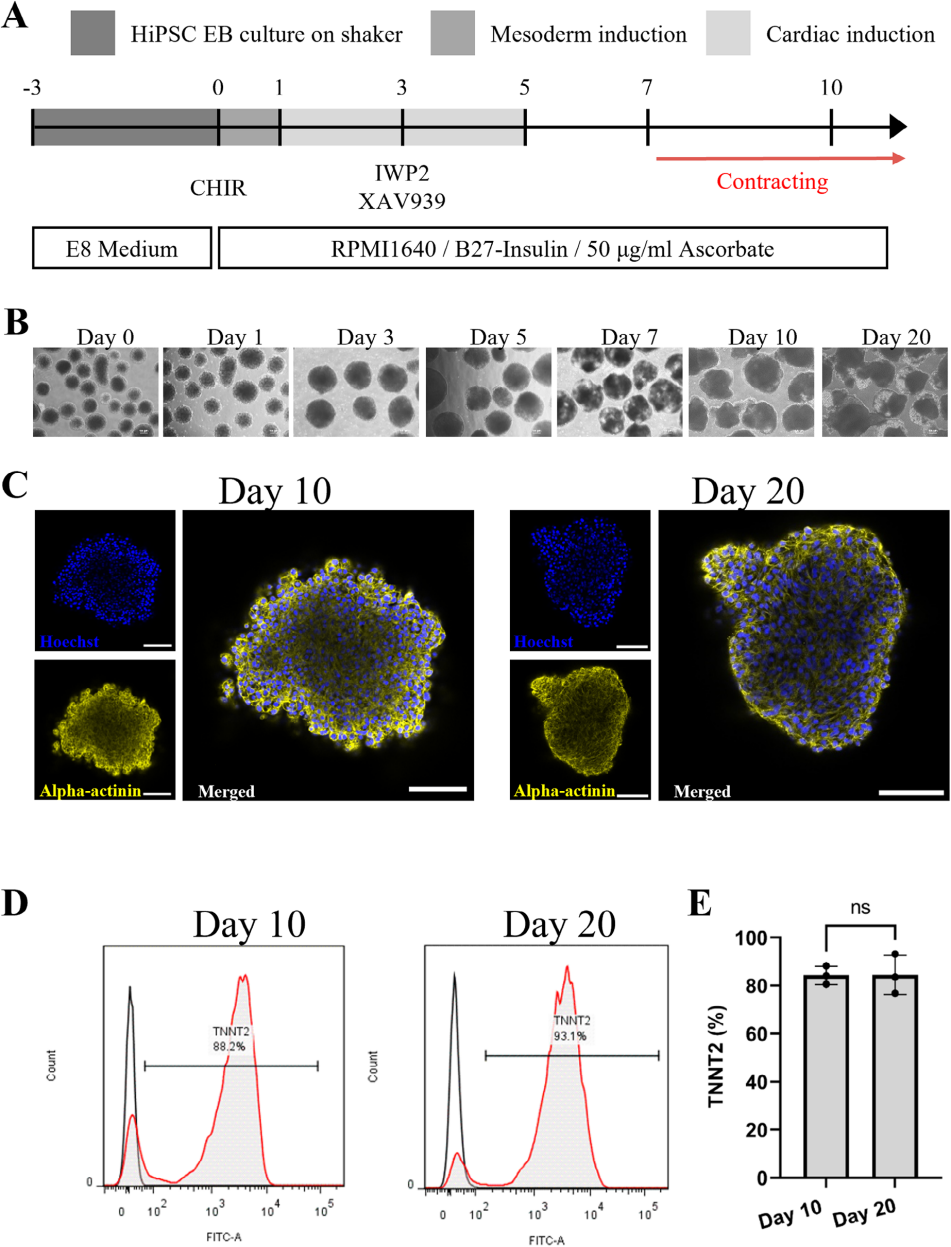
A) Schematic diagram illustrating the protocol for differentiating CMs in EBs. B) Brightfield images of developing organoid over 20 days of differentiation. Scale bars represent 100 µm. C) Immunostaining of EB‐CMs. The expression of α‐actinin (yellow) in whole‐mount stained EB‐CMs at day 10 and day 20 of differentiation is shown. Nuclei are stained with Hoechst 33 342 (blue). Images were captured using a SP8 Leica confocal microscope. D) FC analysis of differentiated CMs. E) Quantification of TNNT2 + CMs (n = 3). Data are presented as mean ± SD (The p‐values were determined using *t*‐test, *p* < 0.05 (*), *p* < 0.01 (**), and *p* < 0.001 (***), “n.s.”: no significant difference.).

Following EB formation by using a custom‐fabricated acoustic patterning device, they were cultured for two more days in a linear shaker in E8 medium. CM differentiation was initiated by inducing mesoderm formation by exposure to CHIR. This method efficiently directed the differentiation of mesodermal aggregates into cardiac cells in a monophasic process (**Figure** **8**A), as detailed previously described protocol.^[^ ^37^ ^]^ Induction of mesodermal progenitors by supplementing CHIR99021 as Wnt signaling stimulator, and ascorbate was highly efficient, the FC analysis revealed about 98% brachyury positive cells (Figure S3, Supporting Information).

Bright‐field imaging revealed a significant increase in the size of CMs throughout the differentiation protocol (Figure 8B). To determine the presence of cardiac cells, immunofluorescence staining was performed with antibodies against the cardiac‐specific marker Alpha actinin. Confocal microscopy for cardiac Alpha actinin showed that organoids had developed sarcomeres by day 10, and improved sarcomere organization was observed by day 20 (Figure 8C). Beating CMs were first detected as early as day 6 of differentiation, and robust and regular beating was observed in all organoids by day 10 and persisted for at least 20 days in culture (Supplementary Videos).

To assess the efficiency of CM specification, FC analysis was performed using the CM‐specific marker TNNT2 on days 10 and 20 of differentiation. On day 10, up to 88.2% of cells were TNNT2 positive, increasing to maximally 93.1% on day 20 (Figure 8D). Statistical analysis of FC showed that the fraction TNNT2 positive cells was 84.26 ± 3.1 (n = 3) at time point 10, while 84.46 ± 6.68 (n = 3) at time point 20 (Figure 8E).

## 3 Discussion

Commercial methods such as microwell, spinner flask,^[^ ^38^ ^]^ and micropatterned techniques are usually employed to form EBs in a controlled and reproducible manner. However, these methods are often time‐consuming and labor‐intensive, expensive and lack in uniformity of spheroid geometry that can be achieved.^[^ ^10^, ^39^ ^]^ The fast, size controlled, scalable and easy production of uniform EBs is of significant interest in mass production of iPSC‐derived cells. While previous research groups have produced cell clusters using similar approaches, those methods had limitations that this study has successfully overcome. Notably, we introduced the ability to perform acoustic clustering directly within a cell culture incubator using continuous active ultrasound.

For the first time, we applied a novel approach to determine the operating frequencies, which proved effective in this study. While the optimal working frequency can theoretically be calculated based on the spacing between piezo pairs, many factors affect its practical application.^[^ ^40^ ^]^ Variations in the actual distance between the piezo elements can result from inaccuracies during manufacture. Milling tolerances can be up to ± 50 µm, which is enough to cause deviations from the calculated optimal frequency.^[^ ^41^ ^]^ Additionally, adhesive layers were often assumed to be 100 µm thick in previous studies, but this is rarely verified. Moreover, the speed of sound in the cell culture medium is typically equated with that of water, despite no validation that the velocities are truly identical. As a result, many research groups rely on optical methods to identify the working frequency, though they use relatively large increments between tested frequencies.^[^ ^22^, ^42^ ^]^ While this approach can be effective, it is not the most precise solution. Our method allows for fine‐tuned frequency adjustments tailored to the chamber conditions, identifying areas of constructive interference. Temperature fluctuations of 0.5 °C should have a negligible effect, compared to water, this would cause a minor wavelength shift of ± 0.1 µm.^[^ ^43^ ^]^ Although we identified the optimal frequency, several factors still hinder the creation of a perfectly uniform acoustic field. One of the key challenges is variance in the piezoelectric ceramics themselves. Different regions of the ceramic surface result in different displacements, as highlighted in the study by Chansoria et al.^[^ ^44^ ^]^ Furthermore, physical constraints, such as ultrasound wave behaviors within rectangular structures, can lead to edge effects, as seen in experiments and in simulations. For instance, elongated cellular aggregates, rather than spherical clusters, can be observed at the edges of the acoustic chamber (Figure S1, Supporting Information). These findings are supported by simulation results, which have also shown the accumulation of cells in elongated structures.

All experiments were conducted at maximum voltage, with the highest induced pressure reaching 0.79 MPa^2^. Although hydrophone measurements indicated that the pressure should be highest in the 1 MHz device, both live imaging of pattern formation and simulation results of sound pressure and particle tracing suggest that the pressure is higher in the 2 MHz setup. The hydrophone measurements were likely affected by the needle hydrophone itself, as it disrupts the formation of the standing ultrasound wave. Additionally, the hydrophone's thickness of 500 µm is greater than the distance between two nodes in the 2 MHz system. More accurate measurements could be achieved in the future using a fiber‐optic hydrophone.

As illustrated in our study, continuous ultrasound exposure reduces size distribution inhomogeneities among the clusters. Between 6 and 24 h of exposure, significant differences were observed, suggesting that long‐standing waves are optimal for creating uniform clusters. The differences in size may result from the more loosely connected cells on the periphery of the 6‐h group, which could be washed away during the transfer process. Additionally, the iPSCs in this group have 18 fewer hours to proliferate compared to the 24‐h group. Furthermore, prolonged exposure to ultrasound could influence the proliferation kinetics, as has been observed in other cell types.^[^ ^45^ ^]^ Shorter exposure times, such as 1.5 h or less, result in loosely connected cell clusters which mostly were destroyed during transfer to a petri dish for further culture. Miao et al.^[^ ^28^ ^]^ also demonstrated reduced efficiency in cluster formation when support structures are removed too quickly. EBs of the 6‐h group exhibit a rougher surface which might become a bigger problem during dynamic culture on the linear shaker, as the rougher surface would lead to more friction between the EBs and the plastic of the petri dish. Therefore, 24 h of ultrasound was chosen for further experiments. One consequence of continuous ultrasound exposure is a rise in temperature within the acoustic chamber, potentially reaching non‐physiological levels. Levario Diaz et al.^[^ ^40^ ^]^ also observed that acoustic standing waves can cause a temperature increase, reporting a maximum rise of 2 °C. However, no further temperature increase was observed after 10 min. In addition to temperature, acoustic amplitude can also directly influence cell viability.^[^ ^40^, ^46^ ^]^ An amplitude of 10 Vpp after 10 min of ultrasound exposure was found to have a negative effect on cells, as shown in studies using HeLa and human dermal fibroblasts cells.^[^ ^40^ ^]^ Interestingly, these viability issues could not be attributed to temperature, as the chamber never exceeded 35 °C. In our study, we successfully maintained a natural cell culture environment by designing a cooling system that allowed extended ultrasound exposure in an incubator without harming the cells.

Beyond the duration of ultrasound exposure, the seeding density of hiPSCs also affects the final result. Similar to other studies, increasing the seeding density in our work resulted in the formation of larger clusters.^[^ ^31^ ^]^ Depending on the intended application, cluster sizes can be varied by adjusting the seeding density and time of culture, with diameters ranging from 100 to 320 µm. This size range is sufficient for many applications and could replace products like ultra‐low attachment plates. Currently, the most advanced ultra‐low attachment plates from Kugelmeiers can hold up to 1500 cells per microwell. However, producing larger clusters requires plates with limited throughput unless automated pipetting systems are used. In the case of acoustic clustering, cluster size is limited by the spacing between nodal points, which is frequency‐dependent.^[^ ^18^ ^]^ To address this, we developed a second setup with piezoelectric ceramics of a lower frequency, allowing us to produce significantly larger clusters. Additionally, post‐clustering culture on a shaker permits further size adjustment. Clusters generated by ultrasound remained stable after 24 h, even under dynamic culture conditions, which distinguishes our approach from previous mass production methods. With up to 28 000 clusters formed, our work sets a new benchmark in acoustic cluster generation. As demonstrated in our study, we successfully produced cell clusters of comparable or even superior quality and size to those generated using Kugelmeiers plates. The quality of cluster formation via ultrasound could be further enhanced by addressing the issue of inhomogeneous cluster formation near the walls of the sound chamber. By leveraging simulations, we can explore new solution strategies to make this method even more precise and reproducible. Furthermore, aggregates formed by ultrasound exhibit a smoother appearance, which became clearly visible after 5 days. This smoother morphology may indicate improved cell‐cell connection within the clusters created by acoustic forces. However, additional experiments are necessary to confirm this hypothesis and explore the potential advantages on cell‐cell connections of ultrasound‐induced cluster formation.

One current limitation observed in this study is the use of a linear shaker for further culture. Brightfield images show that EBs exhibit a rounded structure immediately after acoustic treatment, but by the first day of shaker culture, clusters from seeding densities of 1 and 3 million become irregular in shape. Optimal conditions for extended culture have yet to be identified. By the third day, the irregularities smoothed out and the EBs of a seeding density of 3 million cells regained a rounded form. It may be possible to substitute linear shaker culture with ultra‐low attachment plate culture, or to run experiments with shakers with varying stroke and speed. The work of Miao et al.^[^ ^28^ ^]^ currently represents the highest throughput achieved using acoustic standing waves. Their approach generated three standing waves in a cell‐laden GelMA gel, forming nodal points in the z‐direction as well. This method produced relatively uniform clusters, with the majority ranging from 90 to 120 µm in diameter. However, the maximum size limit remains unclear. As larger aggregates form, their increased weight may exceed the capability of the acoustic forces to keep them in place. This method requires 3 days to produce densely packed aggregates, with spheroid formation efficiency starting below 25% on day 1 and increasing to 90% by day 3. The resulting spheroids are described as loosely packed, which, according to the research group, necessitates further culture in ultra‐low attachment plates. In contrast, our method produces stable clusters within 24 h, likely due to the continuous application of acoustic forces, which keep the cells in constant contact. To allow recovery from acoustic stimulation and to be more closely resemble conventional protocols, EBs were incubated on a linear shaker for two additional days. Additionally, the ultraviolet (UV) crosslinking of the GelMA and subsequent retrieval, including GelMA lysis, may induce oxidative, genotoxic or mechanical stress, potentially promoting spontaneous differentiation of iPSCs. Although the UV light dose used here showed no significant effect on inducing cell death in tests with mammalian cells, additional evaluation under different conditions may be required.^[^ ^47^ ^]^ Beyond that, the use of animal‐derived products poses a barrier to translating these methods for clinical applications, limiting their suitability for human therapies.^[^ ^36^, ^48^ ^]^ In contrast, we are producing clusters without animal derived substances. Further research, such as that by Luo et al.,^[^ ^49^ ^]^ offers faster cluster formation in human breast adenocarcinoma cells (MCF‐7) within 12 h. However, these clusters show drawbacks in terms of compactness and roundness. Although compactness was not directly evaluated in this study, brightfield images clearly indicate that these are not dense spherical spheroids, especially when compared to MCF7 spheroids generated in microwells.^[^ ^16a^ ^]^ Similar approaches have been explored by Chen et al.^[^ ^32^ ^]^ and Wu et al.,^[^ ^31^ ^]^ who used standing SAWs in PDMS microchannels. Their cells form clusters within 9 h, followed by culture in low‐attachment plates for another day. However, the efficiency of spheroid formation in that approach was below 60%. Given that only up to 150 cells were clustered per node and the reported mean diameter after 1 day of culture was ≈180 µm, the resulting structures are unlikely to represent fully compact spheroids. In contrast, our study consistently generated aggregates with diameters ≈200 µm composed of ≈1700 cells, indicating a substantially higher degree of cellular compaction and structural integrity.

Other studies combining biomaterials with acoustic waves have utilized acoustically induced micro vortices around air bubbles in microfluidic systems to aggregate cells within collagen‐based matrices.^[^ ^50^ ^]^ However, these approaches are limited by the use of animal‐derived biomaterials. Substituting them with human‐based alternatives would significantly increase production costs. Furthermore, the group reported that their device, constrained by the properties of collagen, is only functional for up to 30 min. At a production rate of one cell aggregate every 10 s, this results in a maximum output of 180 aggregates per run. It would take 165 such systems operating in parallel to achieve comparable numbers, such as the 28.000 units produced in the present study. Each aggregate, after 12 h of additional cultivation, contained ≈100 to 200 cells and exhibited a diameter of ≈200 µm, reflecting a low degree of cellular compaction as previously noted. The flexibility of this method is also limited, as it does not allow for the generation of larger or more compact aggregates of different sizes, as demonstrated in the current approach. The maximum number of cells per aggregate appears to be limited by the physical properties of the system. In contrast, the generation of EBs and spheroids typically relies on cell‐only systems without exogenous matrices such as collagen, as this allows a simpler and highly reproducible process driven solely by intrinsic cell–cell interactions. While collagen can promote aggregation and increase structural stability, it also carries the risk of altering natural signaling pathways. It may interfere with endogenous gradients critical for differentiation or promote fibroblast overgrowth.^[^ ^51^ ^]^ Thus, the matrix‐free, cell‐only system presented in this study provides a more cost‐effective, scalable, and controlled environment to study natural processes and generate reliable results.

EB size is a critical determinant in the differentiation of hiPSCs, as specific sizes may preferentially direct the development of certain cell lineages. For example, previous studies have shown that larger EBs tend to support cardiac differentiation, while smaller EBs are more conducive to endothelial cell formation.^[^ ^52^ ^]^ Based on this understanding, our study focused on differentiating hiPSCs into CMs. Using our acoustic aggregation technique, we achieved precise control over EB size by adjusting seeding density, ultrasound exposure duration, and frequency. Moreover, as demonstrated, the entire process can be scalable. In our initial approach, we successfully produced 28 000 EBs per run. While we have not yet attempted to further increase the number of EBs in a single run, there should be no issues in scaling up the output. This could be achieved by either utilizing larger piezo elements or implementing additional layers. This method allowed us to produce uniformly sized EBs, facilitating efficient and reproducible CMs differentiation in a high throughput manner. The results of our experiments confirmed that hiPSCs aggregated using the acoustic system formed EBs showed high efficiency in cardiac differentiation. Notably, the expression levels of cardiac markers such as TNNT2 and α‐actinin were similar to those observed with standard EB formation methods, indicating that our technique does not alter fundamental differentiation pathways and give similar result with scalable system of EB‐CMs production.^[^ ^9^, ^53^, ^54^ ^]^ Furthermore, analyses of hiPSC‐derived aggregates at days 3 and 5 after aggregation revealed that the acoustic aggregation process preserved the pluripotency of the cells and guaranteed their further differentiation potential. Consistent with the findings of Hamad et al.,^[^ ^54^ ^]^ our approach effectively induced cardiac differentiation via modulation of Wnt signaling using a small molecule‐based protocol. This conformity with established research emphasizes the reliability of our method. Taking together, using the acoustic aggregation technique, we achieved up to 90% CM positive markers, demonstrating the robustness and effect of our approach. These results highlight the potential of acoustic aggregation as a superior method to generate uniform EBs and keep the efficiency of CM differentiation from hiPSCs. While our focus in this study is on cardiac differentiation, the acoustic aggregation platform can be easily adapted to other small molecule‐based differentiation protocols to generate various lineages from hiPSCs. Furthermore, this technique has potential applications beyond pluripotent stem cell aggregation and can be used to generate aggregates from other cell types.

## 4 Conclusion

In conclusion, our custom acoustic patterning device addresses the limitations of traditional EB formation methods by offering a scalable, reproducible, and cost‐effective solution for generating uniformly sized EBs, while ensuring the cells remain unharmed and unstressed. Furthermore, it improves upon existing acoustic techniques for cell cluster formation by resolving challenges such as heat dissipation, controlling the number of aggregates, fine‐tuning ultrasound parameters, avoiding the use of animal‐derived materials, and enabling scaffold‐free production of compact robust clusters. Using this method, we successfully generated EBs ranging from 70 to 320 µm in diameter, each consisting of up to ≈1600 cells, and achieved yields of up to 28 000 EBs following 24 h of continuous ultrasound exposure. Following this, the EBs were differentiated into functional, beating CMs. This approach holds significant promise for advancing high‐throughput differentiation protocols and facilitating more efficient generation of specific cell types from hiPSCs.

## 5 Experimental Section

### Acoustic Device Fabrication

A specially designed setup was used to assemble the hiPSCs. Three different devices were used for the experiments. The ultrasonic chambers themselves consisted of milled polymethyl methacrylate (PMMA) blocks. A chamber of 30 × 30 mm^2^ (device 1), 33 × 33 mm^2^ (device 2), and 60 × 60 mm^2^ (device 3) was milled. The wall thickness was 0.5 mm for devices 1 and 2, while structure 3 had a wall twice as thick at 1 mm, limited by the geometry of the milling head. The piezoelectric ceramics were then glued to one wall each, one ceramic per wall, that is, a total of four for one setup. For the experiments, piezoelectric ceramics of the sizes 30 × 10 × 2 mm^3^ (plate c144 × 30 y10 t1 wAg, PI Ceramic, Lederhose, Germany) (device 1), 33 × 10 × 1.97 mm^3^ (plate c255 × 33 y10 t1, 97 wCuNi, PI Ceramic) (device 2) and 60 × 45 × 1 mm^3^ (plate c255 × 60 y45 t1 eAg, PI Ceramic) (device 3) with a base frequency of 2 MHz and 1 MHz were used. Each ceramic was individually driven by a channel of a radio frequency (RF) signal generator (2‐channel arbitrary signal generator, P 4115, PeakTech, Ahrensburg, Germany). The acoustic chamber was placed on an aluminum plate under which a temperature unit was located. This consisted of a Peltier element (QC‐127‐1.4‐8.5MS, Quick‐Ohm, Wuppertal, Germany) to which cooling fins were glued. A fan (CFR1B5X‐S‐P, OLC Inc, Pleasanton, United States) was attached to the cooling fins to dissipate the heat. The temperature was recorded by an infrared sensor (Contact‐less Infrared Sensor MLX90614, Melexis, Ypres, Belgium), which was mounted above the acoustic chamber using a second custom‐made PMMA unit. Both the infrared sensor and the peltier element were connected to an arduino, which applied a current to the peltier for cooling as soon as the measured temperature rose above 36.5 °C.

### Temperature Measurement

The determination of the temperature inside the acoustic chamber was performed with fully assembled devices 1 and 2. Each device was operated at 10 Vpp voltage at its resonance frequency. For every combination, tests were performed with and without the operating cooling unit. Temperature was measured with an infrared sensor. Each parameter combination was tested in triplicate.

### Electrical Characterization of Acoustic Chambers

The piezoelectric ceramics implemented in the different devices were analyzed for their resonance frequency. Reflection and gain measurements were carried out using the Bode 100R2 vector network analyzer (OMICRON electronics, Klaus, Austria). For this purpose, a frequency sweep was generated ranging from 0.5 to 1.5 MHz for the 1 MHz ceramics and from 2 MHz to 2.5 MHz for the 2 MHz ceramics. The output level was set at 0 dBm with a nominal impedance of 50 Ω. Measurements were recorded at 1 kHz with reflection attenuated by 10 dB and gain attenuated by 20 dB. A total of 4096 data points were considered in each case. During the measurement, the acoustic device was set up as outlined in chapter 2.6. with a working cooling unit. In addition, the devices were placed in the incubator at 37 °C, 95% relative humidity, and 5% CO_2_. The operating frequencies of the respective devices were primarily determined by identifying the frequency with the highest gain. Since replicates of each device are subject to inaccuracies of the milling process, the measurements must be redone for each individual device.

### Measurement of Pressure Field

To determine the acoustic pressure in devices 1 and 2, a grid scan of 3 × 3 mm^2^ in the middle of the chamber was performed using a needle hydrophone (NH0500, Precision Acoustics, Dorchester, United Kingdom) amplified by a preamplifier (HP, Precision Acoustics). The hydrophone was connected to a 4‐channel oscilloscope (Picoscope 6424E, Picotechnology, Eaton Socon, United Kingdom) to visualize and record the voltage values of each step. Fields were measured at an applied voltage of 2.5 Vpp, 5 Vpp and 10 Vpp at their individual operating frequencies. The hydrophone was attached to a customized Prusa Printer (i3 MK3S+, Prague, Czech Republic), which was controlled via an arduino. A step width of 50 µm was selected for the measurements. After each step, a 5 Volt signal triggered the oscilloscope, which then started recording the voltage. Each scan took ≈30 min. The data was analyzed using Origin 2023 SR0 for Windows (Origin, OriginLab, Northhampton, United States).

### Acquisition of Cell Pattern

Images were captured under two different experimental setups, where acoustic devices were placed inside an incubator beneath a three‐axis linear stage. A camera (COE‐032‐M‐POE‐04‐IR‐C, Opto‐Engineering, Mantua, Italy) with a telecentric lens (TC23007, Opto‐Engineering) was mounted to record the images. Photographs were taken at intervals of 1, 10, 30, and 60 s after ultrasound excitation, along with a control image without ultrasound activation. Device 1 contained 3 mL of E8 medium with 6 million hiPSCs, while device 2 was filled with 4 mL of medium and 10 million hiPSCs. The cooling system was not used for this experiment.

### Ultrasound Field Simulation

A 2D finite element model was developed using the Acoustic‐Piezoelectric Interaction interface in COMSOL Multiphysics (COMSOL AB, Stockholm, Sweden) to simulate the system's acoustic response. The model included a PMMA chamber with attached lead zirconate titanate ceramics (PZT‐5H) and a water domain. The chamber's geometry and material properties were based on the experimental setup. The model's geometric parameters were based on device 1 and device 2 presented in 0. Four physics interfaces were defined for the model. The water domain was assigned the “Pressure Acoustics, Frequency Domain” (*acpr*) and “Particle Tracing for Fluid Flow” (*fpt*) interfaces. The solid components, including the PMMA wall and the PZT piezoelectric ceramics, utilized the “Solid Mechanics” (*solid*) interface, while only the ceramic domain made use of the “Electrostatics” (*es*) node. Additionally, the boundary between the PMMA and water domains was assigned the “Acoustic‐Structure Boundary” (*asb*) multiphysics interface, and the piezoelectric transducers also utilized the “Piezoelectricity” (*pze*) multiphysics node. The water domain was modeled as a viscous fluid, while the solid domains were treated as linear elastic materials. Furthermore, the PZT ceramic was assigned piezoelectric characteristics in the *solid* and the *pze* nodes. Finally, the corners of the PMMA chamber and the short ends of the PZT ceramic were modeled as free boundaries, since these surfaces are unrestrained in the physical setup. Further node‐specific physics definitions were made. In the case of solid materials, low‐reflecting boundaries were added to the free surface of each piezoelectric ceramic facing away from the chamber. These very same surfaces were assigned “Ground” characteristics in the electrostatics node, while the opposing surfaces in direct contact with the PMMA domains were assigned an electric potential of 5 V. The frequency domain solver was used to analyze the generated ultrasonic field and particle interactions with drag and acoustophoretic forces. The mesh was refined to ensure convergence, with mesh element sizes determined by the minimum wave speed in each material. Parameter sweeps were performed to ensure the independence of the results from the chosen mesh element size and to find an adequate frequency for the pattern generation. The final model was solved in the frequency domain, focusing on the system's response to an excitation with 1 MHz and 2 MHz frequencies. Finally, the particle tracing node utilized solid polystyrene (PS) beads of 10 µm in diameter, a random release of 100 000 particles at t = 0 s and a drag force following COMSOL's implementation of Stokes’ law and the Gor'kov ideal model for the acoustophoretic radiation force acting on the particles, deriving the used velocity and pressure fields from the pressure acoustics calculation.

### hiPSC Culture

The hiPSC line NP0040‐8, provided by Dr. Tomo Saric from the University of Cologne, Faculty of Medicine, Institute of Neurophysiology, was cultured in 6 cm petri dishes pre‐coated with (10 µg cm^−2^) Matrigel (353 003, Corning, New York, United States).

Every 3–4 days at 70–80% cell culture confluence, NP0040‐8 cells were passaged to ensure maintenance of the culture at >95% pluripotency.^[^ ^54^ ^]^ To make single cells suspension, NP0040‐8 culture was washed with DBPS (−/−) (5 mL), followed by adding ReLeSR (1 mL) (5872, Stemcell Technologies, Cologne, Germany). After 5 min of incubation at room temperature, ReLeSR was discarded, and the plate was incubated for an additional 5 min at 37 °C and 5% CO_2_. Next, the plate was knocked carefully against a solid surface to loosen cells from the surface and later pipetted up to 10 times up and down with 1000 µL pipette and E8 medium (3 mL) generating a single cell dissociated suspension. Dissociated NP0040‐8 cells were mixed with E8 medium (5 mL) supplemented with Rho Kinase (ROCK) inhibitor (10 µm) (Y27632, A11001‐5, Adooq, Irvine, United States). After 24 h the medium was completely refreshed using E8 medium without ROCK inhibitor. Medium was changed every second day and immediately before and after weekends.

### EB Formation

For EB formation, the devices were prepared as follows: An agarose stamp was pressed into the chambers to create a gap. Then, agarose (2%) (Agarose, low gelling temperature, A9414, Sigma‐Aldrich Darmstadt, Germany) was pipetted into the gap and incubated for 10 min at room temperature to allow the agarose to crosslink. Next, the acoustic device was assembled and placed into the incubator. Each of the four ultrasonic transducers was connected to one channel of the frequency generators. hiPSCs were harvested from the petri dish as follows: They were first washed with DPBS (−/−). Subsequently, TrypLE (2 mL) (TrypLE Express Enzyme (1×), without phenol red, 12 604 013, ThermoFisher Scientific) was added and incubated for 1 min at 37 °C. The TrypLE was then aspirated, and the cells were incubated again for 3 min at 37 °C. The petri dish was then knocked 10 times against a pipette box to detach the cells from the substrate. The cells were collected with E8 medium (3 mL). The suspension was then transferred to a collection tube and resuspended in E8 medium (10 mL). Cell suspension (10 µL) was mixed with Trypan blue (10 µL) (T10282, ThermoFisher Scientific) and the cell density was measured by the cell counter (Countess II FL, ThermoFisher Scientific). Meanwhile, the cell suspension was centrifuged at 300 g for 3 min. The cell pellet was then resuspended with an appropriate volume of medium and supplemented with ROCK inhibitor (10 µm). For device 1, E8 medium (3 mL) was prepared with 10 million, 6 million, 3 million, and 1 million. Device 2 was used with 4 mL and 10 million cells. A working volume of 10 mL medium with 10 million cells was prepared for Device 3. Additionally, three different ultrasound exposure durations of 1 hour, 6, and 24 h were used for the cell number of 10 million. All the other experiments were conducted with 24 h of active ultrasound. Voltages of 10 Vpp were used for all experiments. The frequency was determined and selected specifically for each setup. After the cultivation period, the acoustic setup was placed back under the sterile bench. The medium in the chamber was resuspended, and the medium‐EB mixture was then pipetted out of the chamber. Afterward EBs were cultured in 100 mm petri dish on custom made linear shaker at 60 rpm.

### Determination of EB Yield and EB Cell Count

After 24 h of acoustic clustering, EBs were collected and counted. Clustering was performed in three independent runs for each seeding density: 10 million, 6 million, and 1 million cells in device 1, and 10 million cells in device 3. For each condition, three samples of collected EBs were taken, counted, and averaged. To determine the average cell number per EB, selected EBs were collected, counted, enzymatically dissociated into single‐cell suspensions using trypsin, and counted again. This procedure was repeated three times for each experimental setup.

### Geometrical Characterization of Acoustic Clustered EBs

After successful acoustic clustering, all EBs were collected from both the center and the edges of the acoustic chamber, and their geometric properties were measured on different days. Transmitted light microscope images were taken for this purpose. The Cellpose 2.0 plugin from Fiji (Image J, v1.53f51, National Institutes of Health, USA) was used to create mask outlines. Cellpose 2.0 is a general‐purpose cell segmentation software that can be trained on personalized datasets using the human‐in‐the‐loop pipeline. The trained base model included in the setup reliably recognized spheroids in the images. EBs located at the edge of an image, and thus not fully captured, were excluded from the calculations using a script (Supplementary Data). In addition, incorrect outlines have been removed from the results. Only cell clusters, not individual cells, were included in the analysis. The tool measured the area and perimeter of the EBs. To determine the diameter, the EBs were assumed to have a circular structure.

### Characterization of hiPSCs‐EBs by Flow Cytometry

To analyze the degree of EB pluripotency, cell clusters from day 3 and day 5 were handled separately. For each time point, the aggregates were transferred into individual 15 mL collection tubes and centrifuged for 30 s at 300 g. Subsequently, the supernatant was aspirated and the EBs were washed with DPBS (−/−) (5 mL). In the next step, trypsin + EDTA (5 mL, 0.05%) (25 300 054, ThermoFisher Scientific) was added to the collection tube for 30 min. After that, to inactivate trypsin, FBS‐DMEM (2.5 mL, 10%) (FBS, F9665, Sigma‐Aldrich,) was added and centrifuged for 5 min at 300 g. The cell suspension was then filtered using a 40 µm cell strainer (83.3945.040, Sarstedt, Nümbrecht, Germany). Dilutions of the antibodies were also prepared using the DPBS (−/−). In the next step, the cells were incubated with the conjugated antibodies SSEA‐4 PE (1:50) (130‐098‐369, Miltenyi Biotec, Bergisch Gladbach, Germany) and SSEA‐5 APC (1:50) (130‐166‐661, Miltenyi Biotec) for 10 min at 4 °C. Following a washing step with DPBS (−/−) (5 mL). Next, they were resuspended with 500 µL DPBS (−/−) and transferred to a tube (352 054, Corning) for FC. The measurement was performed by the BD FACS Canto (BD Bioscience, San Jose, United States). For the first measurement, 4′,6‐diamidino‐2‐phenylindole (DAPI) (ThermoFisher Scientific) was added at a dilution of 1:5000 to distinguish between dead and living cells. Data was evaluated using Flowjo (BD, United States).

### Live/Dead Imaging

EBs were harvested on days 1, 3, and 5 in a collection tube and centrifuged at 300 g for 30 s. The supernatant was then removed. The live/dead staining was performed using fluorescein diacetate (FDA), propidium iodide (PI) assay (F7378‐10G/P4170‐10116, Sigma‐Aldrich) and Hoechst (Hoechst 33 342 Triydrochloride, ThermoFisher Scientific). Stock solutions were prepared as follows of FDA (0.05 g) was diluted in acetone (10 mL) and of PI (0.05 g) was diluted in ringer solution (10 mL) (University Hospital, Aachen, Germany). The staining mixture was prepared in a 1.5 mL centrifuge tube using PBS (300 µL), PI (10 µL) and FDA (10 µL) dyes and Hoechst (1 µL) solution. EBs were covered by staining mixture (100 µL) and incubated at 37 °C for 10 min. Followed by one washing step with DPBS (−/−) (5 mL). Live/dead ratio was calculated by detecting the green, fluorescent area relative to the total area using ImageJ software (Image J, Version 1.53f51, public domain, Wayne Rasband, National Institutes of Health, Bethesda, USA).

### Differentiation of Ultrasound Generated EBs

On day 0, EBs were counted and 100 EBs per mL were resuspended in RPMI 1640 (61870‐010, ThermoFisher Scientific) with 1× B27 supplement without insulin (175 044, ThermoFisher Scientific) and supplemented with CHIR99021 (8 µm) (C‐6556, LC Laboratories, Woburn, United States) for 24 h. On day 1, the medium was completely refreshed with RPMI 1640 containing 1× B27 supplement without insulin. On day 3, the medium was replaced was refreshed by replacing half of the old medium with an equal volume of fresh RPMI 1640 containing 1× B27 supplement without insulin and supplemented with IWP2 (5 µm) (3533, Tocris, Bristol, United Kingdom) and XAV939 (5 µm) (X3004, Sigma‐Aldrich). The cells were maintained without further medium change on a rocking table at 60 rpm for 48 h. On day 5, the medium was completely changed to RPMI 1640 with 1× B27 supplement without insulin for another 48 h. Starting on day 7 onward, the medium was changed every 3 days with RPMI 1640 containing 1× B27 without insulin and supplemented with ascorbate (100 µg mL^−1^) (013‐12061, Wako Chemicals, Osaka, Japan). Ascorbate (50 µg mL^−1^) was continuously added to the media from day 0 until the end of the experiment. CMs were characterized on days 10 and 20. For characterization, CMs were dissociated using Trypsin‐EDTA (0.05%) (25300‐054, ThermoFisher Scientific), and the cells were pipetted up to 10 times using a 1000 µL pipette after 20–30 min of enzymatic digestion. The cell suspension was passed through a 40 µm cell strainer (542 040, Greiner Bio‐One, Kremsmünster, Austria), and count by automatic counter.

### Characterization of Mesoderm State and Differentiated Cardiomyocytes by Flow Cytometry

For FC characterization, both mesoderm and CM populations were analyzed using specific staining protocols. 3D cell clusters were dissociated into single cells for analysis. For both cell types, 0.5 × 10^6^ single cells were resuspended in bovine serum albumin (BSA) (0.5 mL, 0.5%) and EDTA (0.1 mm) in Dulbecco's phosphate‐buffered saline DPBS (−/−) in 1.5 mL Eppendorf tubes. Cells were centrifuged at 300 g for 2 min at room temperature (RT), and the supernatant was removed. The cells were fixed with paraformaldehyde (PFA) (4%) for 15 min at RT and then permeabilized with Triton X‐100 (0.5%) in DPBS (−/−) for 15 min. After permeabilization, the cells were centrifuged again at 300 g for 2 min at RT, and the supernatant was removed. The cell pellet was resuspended in BSA (100 µL, 0.5%) and EDTA (0.1 mm) in DPBS (−/−) containing 1:400 of Brachyury Rabbit mAb antibody (81 694, Cell signaling) and incubated for 1 h at RT. Post‐incubation, (0.5 mL, 0.5% BSA) and EDTA (0.1 mm) in DPBS (−/−) was added, followed by centrifugation and removal of the supernatant. The cells were washed with BSA (0.5%) and EDTA (0.1 mm) in DPBS (−/−) and resuspended in BSA (200 µL, 0.5%) and EDTA (0.1 mm) in DPBS (−/−) containing 1:1000 Alexa Fluor 488 goat anti‐rabbit IgG1 (A‐11008, Life Technologies, Carlsbad, United States). The cells were incubated for 30 min at RT in the dark. The final pellet was resuspended in BSA (250 µL, 0.5%) and EDTA (0.1 mm) in DPBS (−/−) and stored at 4 °C in the dark until analysis. For CM staining, the cell pellet was resuspended in BSA (50 µL, 0.5%) and EDTA (0.1 mm) in DPBS (−/−) containing 1:50 of Cardiac Troponin T‐FITC (130‐119‐575, Miltenyi Biotec) for 1 h at RT in the dark. Following incubation, BSA (0.5 mL, 0.5%) and EDTA (0.1 mm) in DPBS (−/−) were added, centrifuged, and the supernatant was removed. The pellet was resuspended in BSA (250 µL, 0.5%) and EDTA (0.1 mm) in DPBS (−/−) and stored at 4 °C in the dark until analysis with FC (BD LSRFortessa, United Stated).

### Immunofluorescence

EBs were collected on days 3 and 5 in a 15 mL collection tube and stained with a pluripotent stem cell 4‐marker immunocytochemistry kit (Pluripotent Stem Cell 4‐Marker Immunocytochemistry Kit, A2488, ThermoFisher Scientific). Therefore, at first EBs were centrifuged for 30 s at 300 g. Followed by the removing of the supernatant from the EBs. Subsequently, the EBs were mixed with agarose (2%) solution and pipetted in 96‐well plate. After 10 min the agarose was crosslinked, and the fixative solution was added. Samples were incubated at 4 °C overnight. After removal of the fixative, the samples were washed with the wash buffer (diluted to 1X with DBPS). Subsequently, the permeabilization solution was added and incubated for 4 h. In the next step, the permeabilization solution was removed and blocked three times for 10 min. For each day two co‐stainings were performed. One was anti OCT4 in combination with anti SSEA4 and anti SOX2 together with anti TRA‐1‐60. Anti SSEA4, anti SOX2 together with anti TRA‐1‐60 was diluted with the blocking solution to 1:100, while anti OCT4 was diluted 1:200. Primary antibodies were added to the sample and incubated at 4 °C overnight. Next the solution was removed, and samples were washed three times for 10 min. Followed by adding the secondary antibodies Alexa Flour 555 and Alexa Flour 488 and incubation at 4 °C overnight. Secondary antibodies were diluted 1:250. Afterward the washing procedure was repeated. Finally, 2 drops mL^−1^ of NucBlue Fixed Cell Stain (DAPI) was given into the last wash step and incubated for 5 min. Pictures were taken with a Zeiss LSM 710 confocal Laser‐scanning microscope (Carl Zeiss Microscopy Deutschland, Oberkochen, Germany) and Zeiss Efficient Navigation software (Carl Zeiss Microscopy Deutschland).

For whole‐mount immunostaining, EBs were transferred into a well of a 48‐well plate using cut tips of a 1000 µL pipette and the medium was discarded from the well. EBs were fixed with PFA (4%) for 20 min at 4 °C and permeabilized with BSA (0.5%) and Triton X‐100 (0.5%) for 30 min at RT. Blocking was performed with BSA (3%) in DPBS (−/−) for 60 min at RT. After blocking, the solution was removed and BSA (200 µL, 3%) containing a 1:200 dilution of α‐actinin (ACTN2) mouse monoclonal IgG1 (A7811, Sigma‐Aldrich) was added. The samples were incubated overnight on a shaker at 60 rpm at 37 °C. The next day, the wells were washed twice with BSA (3%) in DPBS (−/−), and BSA (200 µL, 3%) containing 1:1000 Alexa Fluor 647 goat anti‐mouse IgG1 (A21422, Life Technologies) and Hoechst 33 342 was added. The samples were incubated for 30 min on a shaker at 60 rpm at 37 °C in the dark. Following staining, the samples were washed three times with 3% BSA in DPBS (−/−) and imaged using a confocal fluorescence microscope (SP8, Leica, Wetzlar, Deutschland).

### Statistical Analysis

The results were presented as the mean ± SD. All analyses were conducted based on at least three independent samples. Sample size (n), *p*‐values, data normalization methods, and the specific statistical tests used for each experiment are detailed in figure legends. Statistical analyses were performed using Origin 2023 SR0 (Origin, OriginLab). For group comparisons, one‐way ANOVA with Tukey's post hoc test was applied. In cases involving only two groups, a t‐test was used. Significance levels of *p* < 0.05 (*), *p* < 0.01 (**), and *p* < 0.001 (***) were defined and all tests were two‐sided.

## Conflict of Interest

The authors declare no conflict of interest.

## Author Contributions

J.H. and E.A. contributed equally to this work. J.H. contributed to conceptualization, investigation, formal analysis, methodology, and the writing‐original draft. E.A. contributed to investigation, formal analysis, methodology, writing the original draft. S.H. contributed to formal analysis, and writing‐review and editing. C.K. contributed to the investigation, and writing‐review and editing. A.M.G. contributed to the investigation, formal analysis, and writing‐review and editing. M.R. contributed to investigation, validation, and writing‐review and editing, K.P. contributed to formal analysis, supervision, resources, and writing‐review and editing. H.F. contributed to conceptualization, formal analysis, methodology, supervision, resources, and writing‐review and editing. All authors have given approval to the final version of the manuscript.

## Supporting information



Supporting Information

Supplemental Video 1

Supplemental Video 2

Supplemental Video 3

Supplemental Video 4

Supplemental Video 5

Supporting Information

## Data Availability

The data that support the findings of this study are available from the corresponding author upon reasonable request.
