# Supplementary material for: Mass Production of Uniform Embryoid Bodies by Acoustic Standing Waves
Source: Small Methods. 2025 Aug 11;9(9):e01283. doi: 10.1002/smtd.202501283 (PMC12464654; doi:10.1002/smtd.202501283)
Supplement: Supplementary file 1 — Supporting Information [file SMTD-9-e01283-s003.docx]

Supporting Information

**Mass Production of Uniform Embryoid Bodies by Acoustic Standing Waves**

*Johannes Hahn^1^, Ebru Aksoy^1^, Sarkawt Hamad, Christoph Kuckelkorn, Alejandro Montoya Gomez, Mira Ritter, Kurt Pfannkuche, Horst Fischer**

| 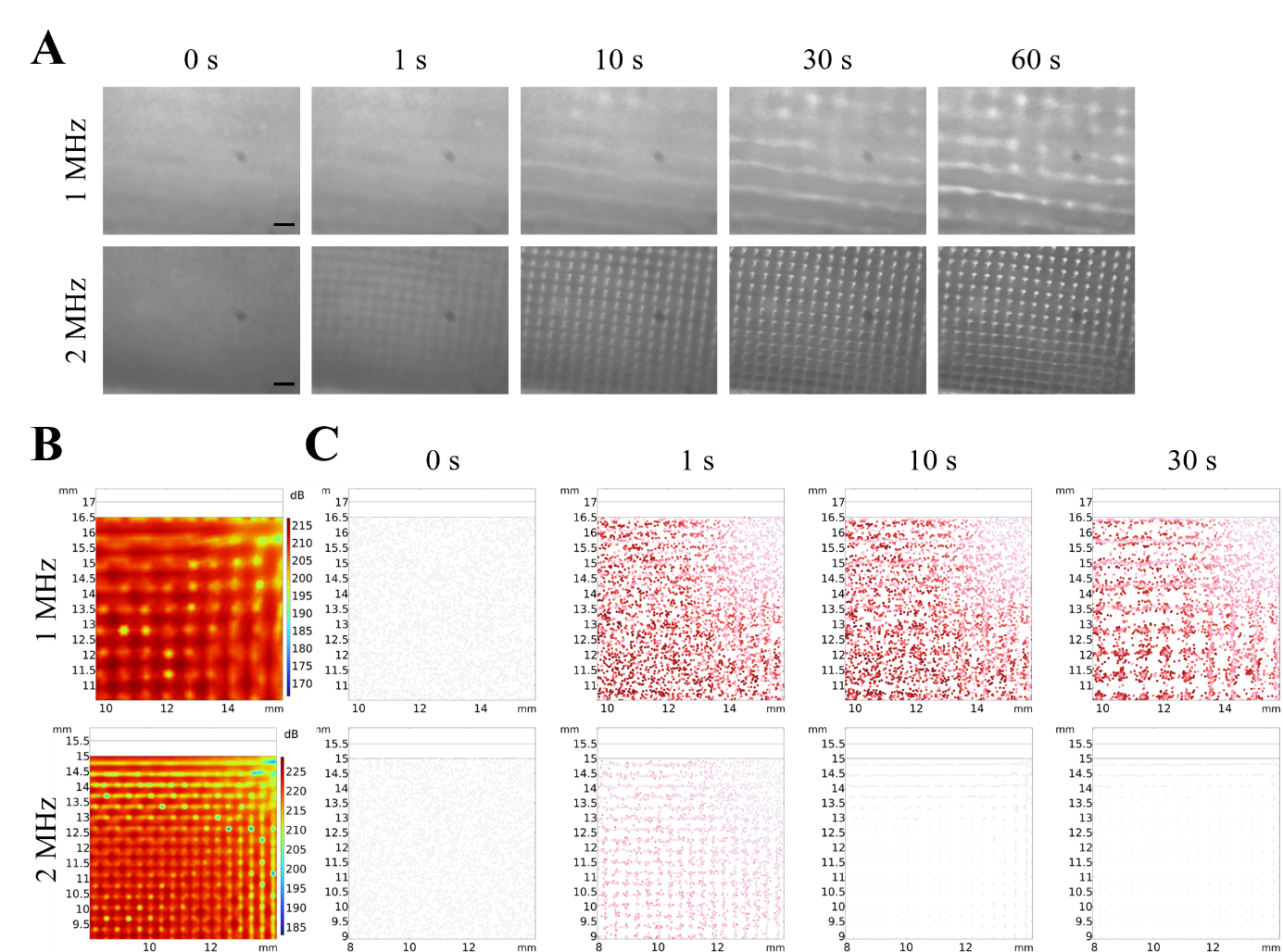 |
| --- |
| **Supplementary Figure 1.** A) Live images showing pattern formation adjacent to the walls of different sound chambers at various time points. Scale bars = 500 µm. B) Sound pressure distribution near the edges of the sound chambers. C) Particle trajectories near the boundaries of the sound chambers. |

| 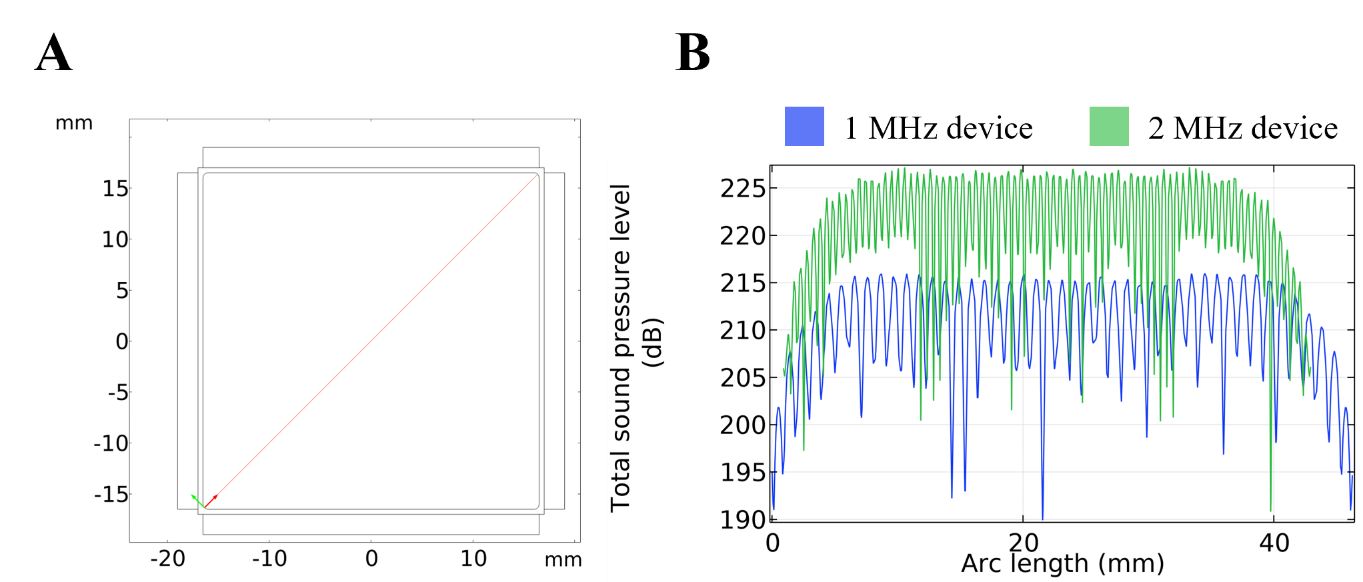 |
| --- |
| **Supplementary Figure 2.** A) Diagonal line indicating the location where simulated sound pressure is measured. B) Measured sound pressure profiles for the different devices. |

| 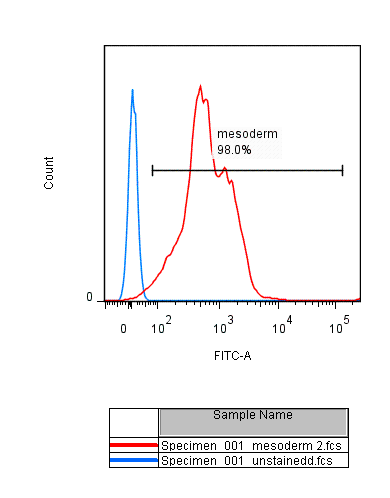 |
| --- |
| **Supplementary Figure 3.** Flow cytometry analysis of mesodermal state following differentiation induction. |

**Supplementary Table 1.** Number of measured samples for EB diameters after 24 hours of ultrasound exposure on days 1, 2, 3, and 5.

|  |
| --- |

Supplementary Data – Fiji Script for EB Analysis

// Ask the user to choose a file to open

fileToOpen = File.openDialog("Choose an image file");

// Open the selected file

open(fileToOpen);

run("8-bit");

run("Cellpose Advanced", "diameter=0 cellproba_threshold=0.0 flow_threshold=0.4 anisotropy=1.0 diam_threshold=12.0 model=cyto2 nuclei_channel=0 cyto_channel=1 dimensionmode=2D stitch_threshold=0.0 omni=false cluster=false additional_flags=4");

getStatistics(area, mean, min, max, std, histogram);

imageWidth = getWidth();

imageHeight = getHeight();

// Set a margin to exclude selections touching the edge (adjust as needed)

margin = 50;

// Clear any existing selections in the ROI Manager

roiManager("reset");

for (i = 1; i <= max; i++) {

// Set the threshold for the current intensity level

setThreshold(i, i);

// Create a selection based on the threshold

run("Create Selection");

// Check if the current selection is within the image bounds with a margin

getSelectionBounds(x, y, width, height);

if (width > 0 && height > 0 && x > margin && x + width < imageWidth - margin && y > margin && y + height < imageHeight - margin) {

// Add the selection to the ROI Manager if it's not touching the edge

roiManager("add");

// Measure the circularity of the selected region

run("Measure", "area perimeter circularity");

} else {

// Clear the selection if it's touching the edge or empty

run("Clear");

}

}

roiManager("Show All");

roiManager("Show All with labels");
