# Supplementary material for: Mass Production of Uniform Embryoid Bodies by Acoustic Standing Waves
Source: Small Methods. 2025 Aug 11;9(9):e01283. doi: 10.1002/smtd.202501283 (PMC12464654; doi:10.1002/smtd.202501283)
Supplement: Supplementary file 7 — Supporting Information [file SMTD-9-e01283-s005.docx]

[
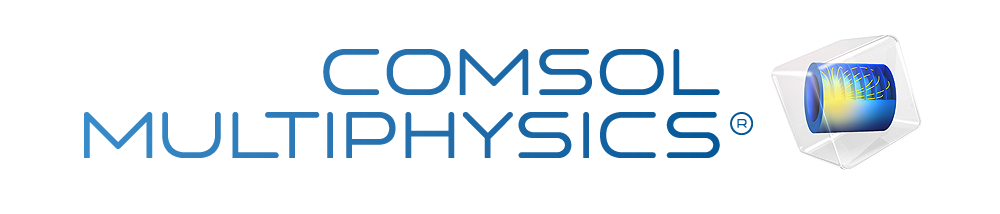
](https://www.comsol.com/)

20240715 Particle Tracing

| Report date | Aug 21, 2024, 1:57:14 PM |
| --- | --- |

Contents

[1. Global Definitions](#cs2842913)

[1.1. Parameters](#cs9715988)

[1.2. Shared Properties](#cs2632878)

[2. Component 1](#cs5325504)

[2.1. Definitions](#cs8168975)

[2.2. Geometry 1](#cs1760827)

[2.3. Materials](#cs5041181)

[2.4. Pressure Acoustics, Frequency Domain](#cs5767787)

[2.5. Solid Mechanics](#cs6883027)

[2.6. Electrostatics](#cs8385327)

[2.7. Particle Tracing for Fluid Flow](#cs1186420)

[2.8. Multiphysics](#cs7263018)

[2.9. Meshes](#cs3078767)

[3. Study 1 - Wave speed definition](#cs3842284)

[3.1. Frequency Domain](#cs1831521)

[3.2. Solver Configurations](#cs1645909)

[4. Study 2 - Mesh convergence study](#cs6353769)

[4.1. Parametric Sweep](#cs5979521)

[4.2. Frequency Domain](#cs1043356)

[4.3. Solver Configurations](#cs4014337)

[5. Study 3 - First sweep, lower frequency](#cs9124539)

[5.1. Parametric Sweep](#cs3169626)

[5.2. Frequency Domain](#cs8355355)

[5.3. Solver Configurations](#cs3616516)

[6. Study 4 - Second sweep, higher frequency](#cs4176182)

[6.1. Parametric Sweep](#cs2340788)

[6.2. Frequency Domain](#cs4379791)

[6.3. Solver Configurations](#cs1054958)

[7. Study 5 - Lower frequency particle tracing](#cs6131902)

[7.1. Parametric Sweep](#cs3435835)

[7.2. Time Dependent](#cs4877198)

[7.3. Solver Configurations](#cs7690633)

[8. Study 6 - Higher frequency particle tracing](#cs4761213)

[8.1. Parametric Sweep](#cs5921680)

[8.2. Time Dependent](#cs2815026)

[8.3. Solver Configurations](#cs7617499)

[9. Study 7 - Run all other studies](#cs8013044)

[9.1. Study 1 - Wave speed definition](#cs1979746)

[9.2. Study 2 - Mesh convergence study](#cs1046839)

[9.3. Study 3 - First sweep, lower frequency](#cs5119221)

[9.4. Study 4 - Second sweep, higher frequency](#cs8750507)

[9.5. Study 5 - Lower frequency particle tracing](#cs2197072)

[9.6. Study 6 - Higher frequency particle tracing](#cs1230313)

[9.7. Solver Configurations](#cs8468992)

[10. Results](#cs6370800)

[10.1. Datasets](#cs1291414)

[10.2. Wave speed definition plots](#cs7304738)

[10.3. Mesh convergence study](#cs2108608)

[10.4. First sweep: lower frequency](#cs2405251)

[10.5. Second sweep: higher frequency](#cs6248189)

[10.6. Plot Groups](#cs8067804)

[10.7. Evaluation Groups](#cs6970213)

# Global Definitions

| Date | Jul 15, 2024, 7:15:09 PM |
| --- | --- |

Global settings

| Name | 20240715 Particle Tracing.mph |
| --- | --- |
| Path | V:\ZWBF\Persönliche Ordner (aktuelle Mitarbeiter)\Alejandro\21 - Kooperationen\01 - Johannes\COMSOL\01 - Paper\20240715_Particle_Tracing.mph |
| Version | COMSOL Multiphysics 6.2 (Build: 415) |
| Unit system | SI |

Used products

| Acoustics Module |
| --- |
| COMSOL Multiphysics |
| Particle Tracing Module |
| CAD Import Module |
| Design Module |

Computer information

| CPU | 12th Gen Intel(R) Core(TM) i9-12900, 12 cores, 63.24 GB RAM |
| --- | --- |
| Operating system | Windows 10 |

## Parameters

Parameters 1

| **Name** | **Expression** | **Value** | **Description** |
| --- | --- | --- | --- |
| w_tank | 30 [mm] | 0.03 m | Water tank width |
| h_tank | 30 [mm] | 0.03 m | Water tank height |
| w_wall | 0.5 [mm] | 5E−4 m | Wall width |
| w_piezo | 2 [mm] | 0.002 m | Piezo width |
| r_fillet | 0.5 [mm] | 5E−4 m | Fillet radius |
| f1 | 1 [MHz] | 1E6 Hz | FIrst tested frequency |
| f2 | 2 [MHz] | 2E6 Hz | Second tested frequency |
| cw1 | 1481 [m/s] | 1481 m/s | c in water @20 deg C |
| N | 5 | 5 | Mesh Elements per wavelength |
| cPMMA1 | 1714.38 [m/s] | 1714.4 m/s | Relevant wave speed for meshing - PMMA |
| cPZT1 | 948.87 [m/s] | 948.87 m/s | Relevant wave speed for meshing - PZT |
| alpha | pi/2 | 1.5708 | Phase shift 1 |
| beta | 0 | 0 | Phase shift 2 |
| d_p | 10 [um] | 1E−5 m | Particle diameter |
| V0 | 5 [V] | 5 V | Input voltage (p2p) |
| pc | 100000 | 1E5 | Particle count |

## Shared Properties

### Default Model Inputs

| Tag | cminpt |
| --- | --- |

# Component 1

| Date | Jul 15, 2024, 8:07:57 AM |
| --- | --- |

Settings

| **Description** | **Value** |
| --- | --- |
| Unit system | Same as global system (SI) |
| Geometry shape function | Automatic |

Spatial frame coordinates

| **First** | **Second** | **Third** |
| --- | --- | --- |
| x | y | z |

Material frame coordinates

| **First** | **Second** | **Third** |
| --- | --- | --- |
| X | Y | Z |

Geometry frame coordinates

| **First** | **Second** | **Third** |
| --- | --- | --- |
| Xg | Yg | Zg |

Mesh frame coordinates

| **First** | **Second** | **Third** |
| --- | --- | --- |
| Xm | Ym | Zm |

## Definitions

### Selections

#### Water domains

| **Selection type** |
| --- |
| Explicit |

| **Selection** |
| --- |
| Domain 4 |

Color

| **Description** | **Value** |
| --- | --- |
| Color | Custom |
| Custom color | {0.39608, 0.71765, 0.85882} |


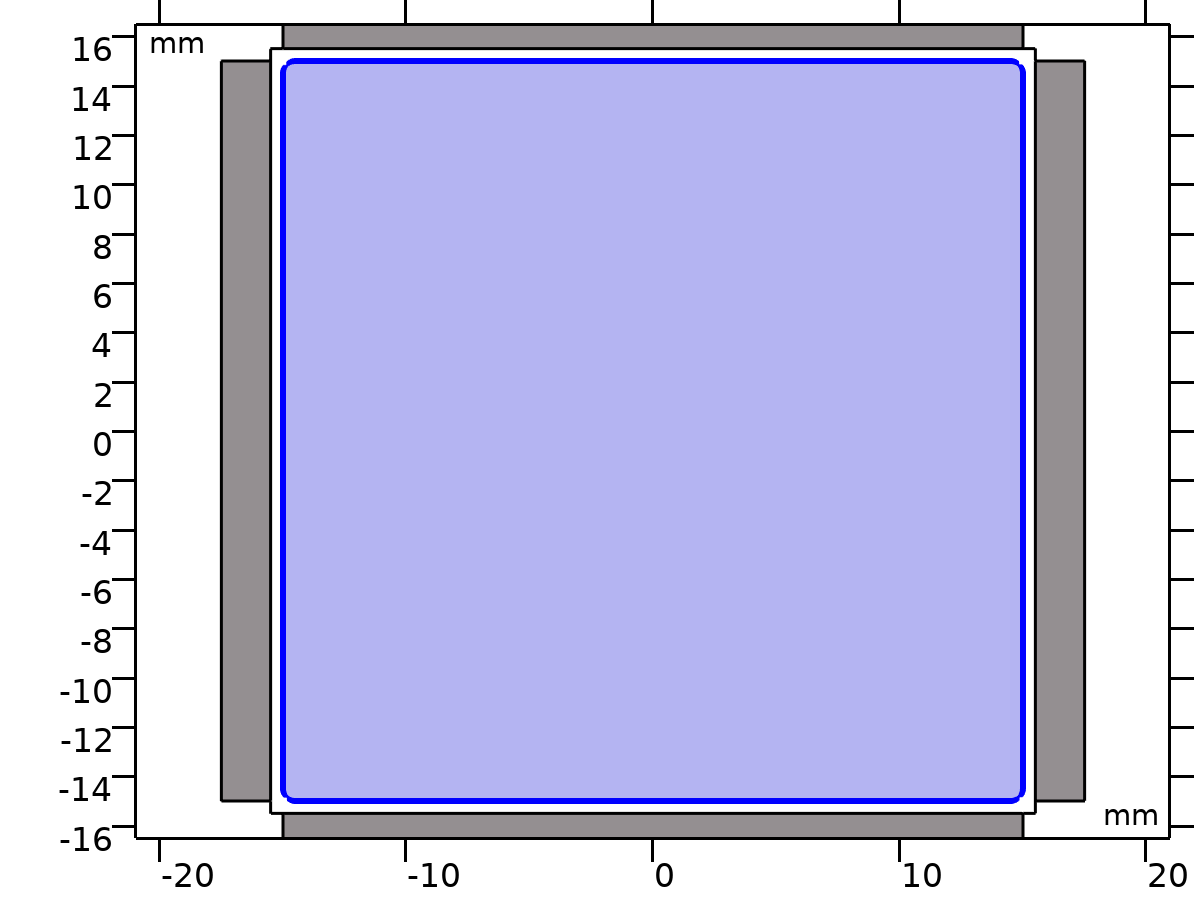


Water domains

#### PMMA domains

| **Selection type** |
| --- |
| Explicit |

| **Selection** |
| --- |
| Domain 2 |

Color

| **Description** | **Value** |
| --- | --- |
| Color | Custom |
| Custom color | {1, 1, 1} |


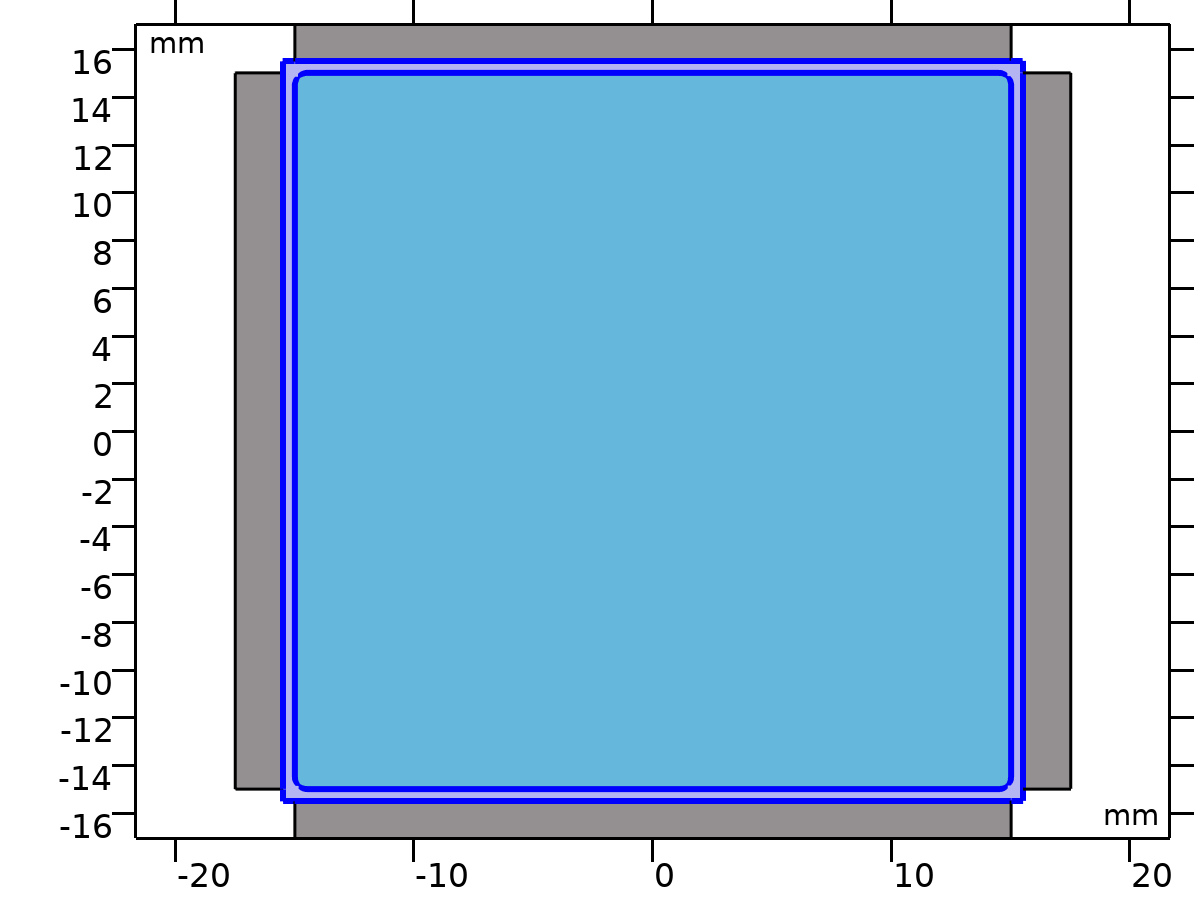


PMMA domains

#### PZT domains

| **Selection type** |
| --- |
| Explicit |

| **Selection** |
| --- |
| Domains 1, 3, 5–6 |

Color

| **Description** | **Value** |
| --- | --- |
| Color | Custom |
| Custom color | {0.58039, 0.56078, 0.56863} |


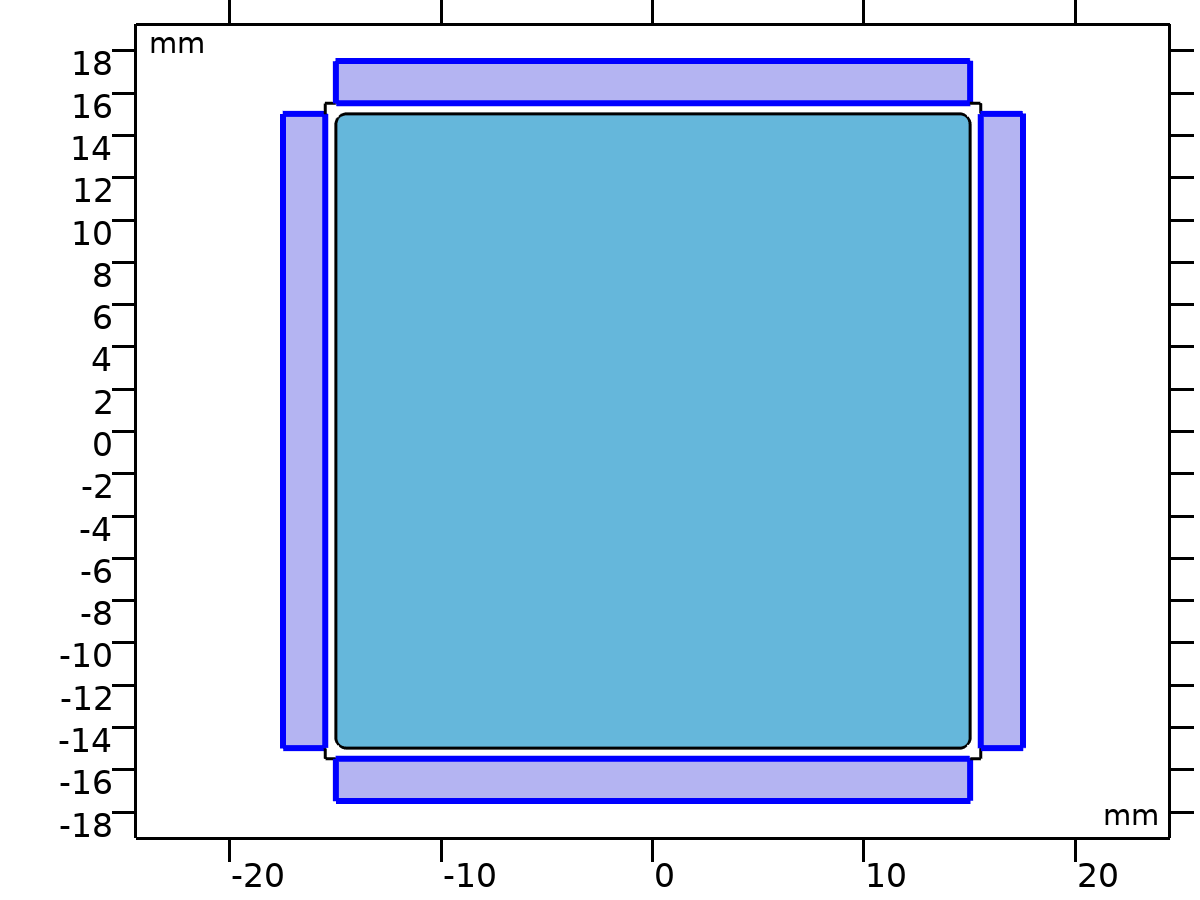


PZT domains

#### Union domains

| **Selection type** |
| --- |
| Explicit |

| **Selection** |
| --- |
| Domains 2, 4 |


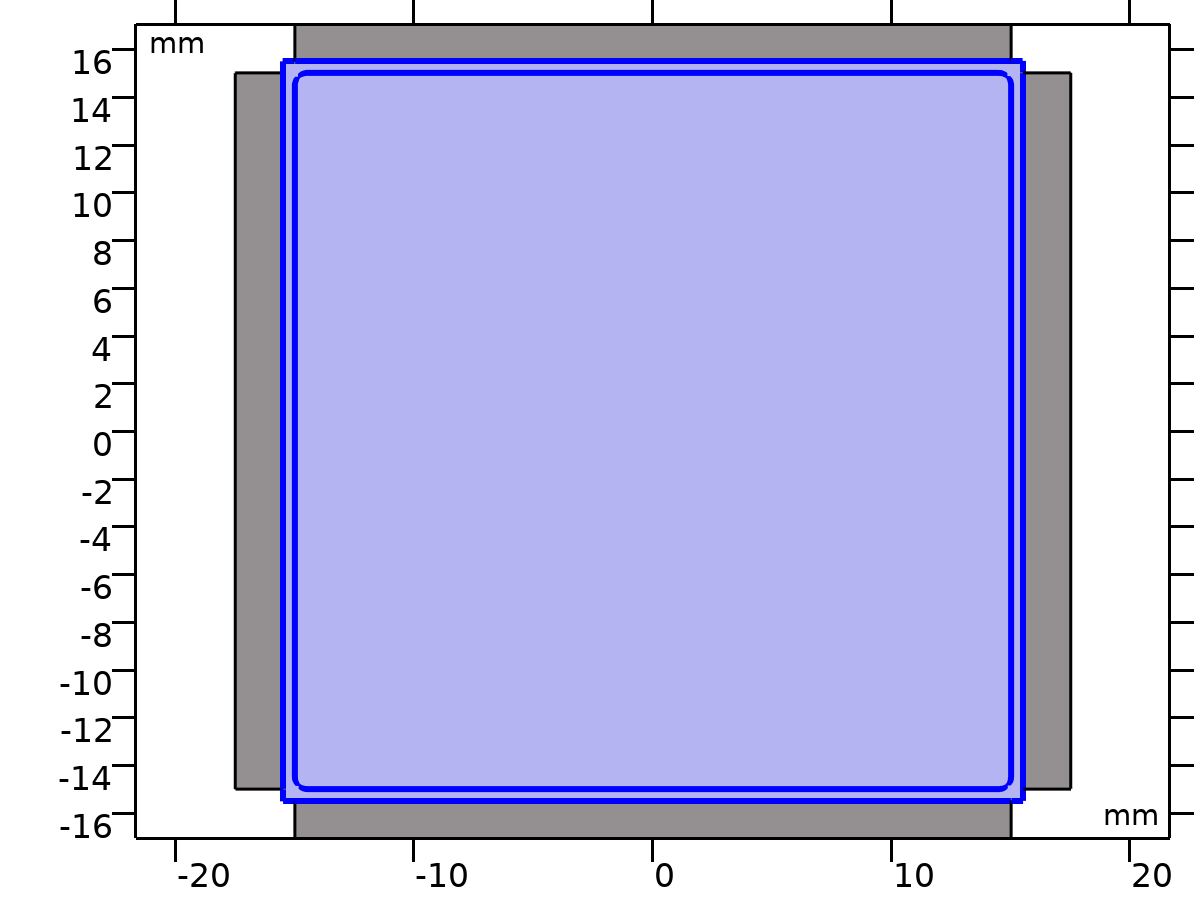


Union domains

#### Solid domains

| **Selection type** |
| --- |
| Explicit |

| **Selection** |
| --- |
| Domains 1–3, 5–6 |


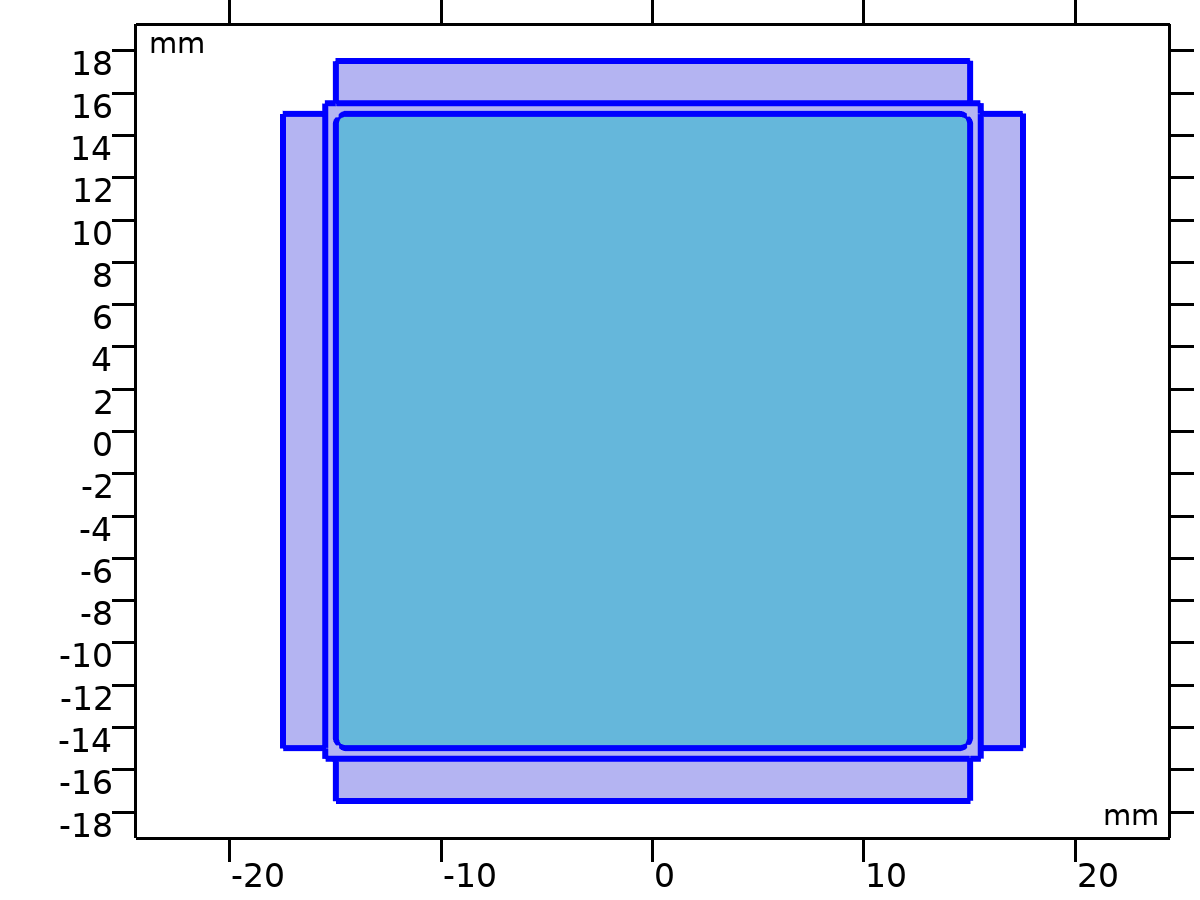


Solid domains

### Coordinate Systems

#### Boundary System 1

| Coordinate system type | Boundary system |
| --- | --- |
| Tag | sys1 |

Coordinate names

| **First** | **Second** | **Third** |
| --- | --- | --- |
| t1 | n | to |

#### Coordinate system for left piezo

| Coordinate system type | Base vector system |
| --- | --- |
| Tag | sys2 |

Coordinate names

| **First** | **Second** | **Third** |
| --- | --- | --- |
| x1 | x2 | x3 |

Base vectors

| **Description** | **Value** |
| --- | --- |
| Input method | All base vectors |
| Assume orthonormal | On |

Base vectors

|  | **x** | **y** | **z** |
| --- | --- | --- | --- |
| x1 | 0 | 1 | 0 |
| x2 | 0 | 0 | 1 |
| x3 | -1 | 0 | 0 |

Origin

| **x (m)** | **y (m)** |
| --- | --- |
| 0 | 0 |

#### Coordinate system for bottom piezo

| Coordinate system type | Base vector system |
| --- | --- |
| Tag | sys3 |

Coordinate names

| **First** | **Second** | **Third** |
| --- | --- | --- |
| x1 | x2 | x3 |

Base vectors

| **Description** | **Value** |
| --- | --- |
| Input method | All base vectors |
| Assume orthonormal | On |

Base vectors

|  | **x** | **y** | **z** |
| --- | --- | --- | --- |
| x1 | -1 | 0 | 0 |
| x2 | 0 | 0 | -1 |
| x3 | 0 | -1 | 0 |

Origin

| **x (m)** | **y (m)** |
| --- | --- |
| 0 | 0 |

#### Coordinate system for right piezo

| Coordinate system type | Base vector system |
| --- | --- |
| Tag | sys4 |

Coordinate names

| **First** | **Second** | **Third** |
| --- | --- | --- |
| x1 | x2 | x3 |

Base vectors

| **Description** | **Value** |
| --- | --- |
| Input method | All base vectors |
| Assume orthonormal | On |

Base vectors

|  | **x** | **y** | **z** |
| --- | --- | --- | --- |
| x1 | 0 | -1 | 0 |
| x2 | 0 | 0 | -1 |
| x3 | 1 | 0 | 0 |

Origin

| **x (m)** | **y (m)** |
| --- | --- |
| 0 | 0 |

#### Material XY-plane System

| Coordinate system type | Base vector system |
| --- | --- |
| Tag | comp1_xy_sys |

Coordinate names

| **First** | **Second** | **Third** |
| --- | --- | --- |
| x1 | x2 | x3 |

Base vectors

|  | **x** | **y** |
| --- | --- | --- |
| x1 | 1 | 0 |
| x2 | 0 | 1 |

Base vectors

| **Description** | **Value** |
| --- | --- |
| Assume orthonormal | On |

Origin

| **x (m)** | **y (m)** |
| --- | --- |
| 0 | 0 |

#### Material YZ-plane System

| Coordinate system type | Base vector system |
| --- | --- |
| Tag | comp1_yz_sys |

Coordinate names

| **First** | **Second** | **Third** |
| --- | --- | --- |
| x1 | x2 | x3 |

Base vectors

| **Description** | **Value** |
| --- | --- |
| Out-of-plane index | 1 |
| Assume orthonormal | On |

Base vectors

|  | **x** | **y** |
| --- | --- | --- |
| x2 | 1 | 0 |
| x3 | 0 | 1 |

Origin

| **x (m)** | **y (m)** |
| --- | --- |
| 0 | 0 |

#### Material ZX-plane System

| Coordinate system type | Base vector system |
| --- | --- |
| Tag | comp1_zx_sys |

Coordinate names

| **First** | **Second** | **Third** |
| --- | --- | --- |
| x1 | x2 | x3 |

Base vectors

| **Description** | **Value** |
| --- | --- |
| Out-of-plane index | 2 |
| Assume orthonormal | On |

Base vectors

|  | **x** | **y** |
| --- | --- | --- |
| x1 | 0 | 1 |
| x3 | 1 | 0 |

Origin

| **x (m)** | **y (m)** |
| --- | --- |
| 0 | 0 |

#### Material YX-plane System

| Coordinate system type | Base vector system |
| --- | --- |
| Tag | comp1_yx_sys |

Coordinate names

| **First** | **Second** | **Third** |
| --- | --- | --- |
| x1 | x2 | x3 |

Base vectors

|  | **x** | **y** |
| --- | --- | --- |
| x1 | 0 | 1 |
| x2 | 1 | 0 |

Base vectors

| **Description** | **Value** |
| --- | --- |
| Assume orthonormal | On |

Origin

| **x (m)** | **y (m)** |
| --- | --- |
| 0 | 0 |

#### Material XZ-plane System

| Coordinate system type | Base vector system |
| --- | --- |
| Tag | comp1_xz_sys |

Coordinate names

| **First** | **Second** | **Third** |
| --- | --- | --- |
| x1 | x2 | x3 |

Base vectors

| **Description** | **Value** |
| --- | --- |
| Out-of-plane index | 2 |
| Assume orthonormal | On |

Base vectors

|  | **x** | **y** |
| --- | --- | --- |
| x1 | 1 | 0 |
| x3 | 0 | 1 |

Origin

| **x (m)** | **y (m)** |
| --- | --- |
| 0 | 0 |

#### Material ZY-plane System

| Coordinate system type | Base vector system |
| --- | --- |
| Tag | comp1_zy_sys |

Coordinate names

| **First** | **Second** | **Third** |
| --- | --- | --- |
| x1 | x2 | x3 |

Base vectors

| **Description** | **Value** |
| --- | --- |
| Out-of-plane index | 1 |
| Assume orthonormal | On |

Base vectors

|  | **x** | **y** |
| --- | --- | --- |
| x2 | 0 | 1 |
| x3 | 1 | 0 |

Origin

| **x (m)** | **y (m)** |
| --- | --- |
| 0 | 0 |

## Geometry 1


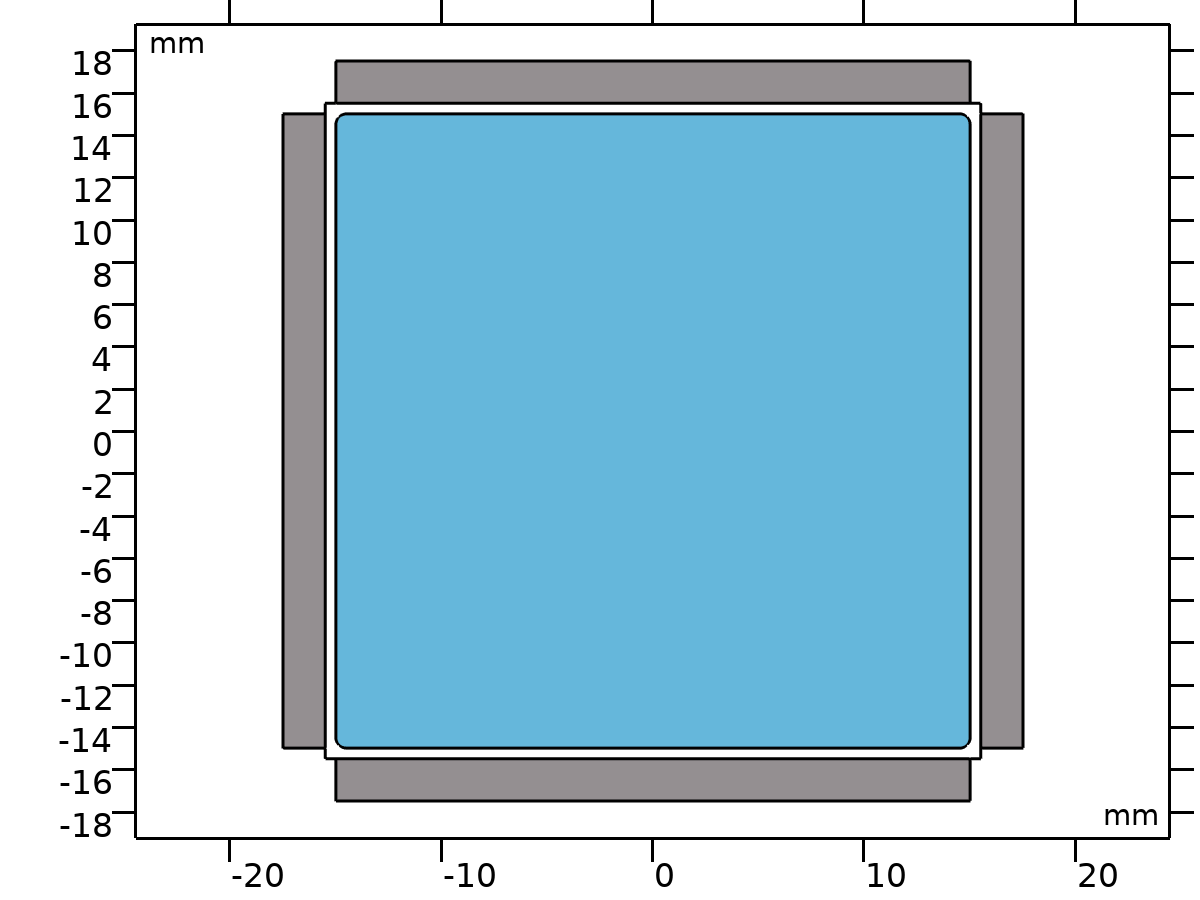


Geometry 1

Units

| Length unit | mm |
| --- | --- |
| Angular unit | deg |

Geometry statistics

| **Description** | **Value** |
| --- | --- |
| Space dimension | 2 |
| Number of domains | 6 |
| Number of boundaries | 32 |
| Number of vertices | 28 |

### Rectangle 1 (r1)

Position

| **Description** | **Value** |
| --- | --- |
| Position | {0, 0} |
| Base | Center |

Size

| **Description** | **Value** |
| --- | --- |
| Width | w_tank |
| Height | h_tank |

Information

| **Description** | **Value** |
| --- | --- |
| Last build time | < 1 second |
| Built with | COMSOL 6.2.0.415 (win64), Aug 21, 2024, 11:43:23 AM |

### Rectangle 2 (r2)

Position

| **Description** | **Value** |
| --- | --- |
| Position | {0, (h_tank + w_wall)/2} |
| Base | Center |

Size

| **Description** | **Value** |
| --- | --- |
| Width | w_tank + 2*w_wall |
| Height | w_wall |

Information

| **Description** | **Value** |
| --- | --- |
| Last build time | < 1 second |
| Built with | COMSOL 6.2.0.415 (win64), Aug 21, 2024, 11:43:23 AM |

### Rectangle 3 (r3)

Position

| **Description** | **Value** |
| --- | --- |
| Position | {(w_tank + w_wall)/2, 0} |
| Base | Center |

Size

| **Description** | **Value** |
| --- | --- |
| Width | w_wall |
| Height | h_tank + w_wall*2 |

Information

| **Description** | **Value** |
| --- | --- |
| Last build time | < 1 second |
| Built with | COMSOL 6.2.0.415 (win64), Aug 21, 2024, 11:43:23 AM |

### Rectangle 4 (r4)

Position

| **Description** | **Value** |
| --- | --- |
| Position | {-(w_tank + w_wall)/2, 0} |
| Base | Center |

Size

| **Description** | **Value** |
| --- | --- |
| Width | w_wall |
| Height | h_tank + w_wall*2 |

Information

| **Description** | **Value** |
| --- | --- |
| Last build time | < 1 second |
| Built with | COMSOL 6.2.0.415 (win64), Aug 21, 2024, 11:43:23 AM |

### Rectangle 5 (r5)

Position

| **Description** | **Value** |
| --- | --- |
| Position | {0, -(h_tank + w_wall)/2} |
| Base | Center |

Size

| **Description** | **Value** |
| --- | --- |
| Width | w_tank + 2*w_wall |
| Height | w_wall |

Information

| **Description** | **Value** |
| --- | --- |
| Last build time | < 1 second |
| Built with | COMSOL 6.2.0.415 (win64), Aug 21, 2024, 11:43:23 AM |

### Union 2 (uni1)

Compose

| **Description** | **Value** |
| --- | --- |
| Keep interior boundaries | Off |

Information

| **Description** | **Value** |
| --- | --- |
| Last build time | < 1 second |
| Built with | COMSOL 6.2.0.415 (win64), Aug 21, 2024, 11:43:23 AM |

### Fillet 1 (fil1)

Settings

| **Description** | **Value** |
| --- | --- |
| Select in sketch | On |
| Radius | r_fillet |

Information

| **Description** | **Value** |
| --- | --- |
| Last build time | < 1 second |
| Built with | COMSOL 6.2.0.415 (win64), Aug 21, 2024, 11:43:23 AM |

### Fillet 2 (fil2)

Settings

| **Description** | **Value** |
| --- | --- |
| Select in sketch | On |
| Radius | r_fillet |

Information

| **Description** | **Value** |
| --- | --- |
| Last build time | < 1 second |
| Built with | COMSOL 6.2.0.415 (win64), Aug 21, 2024, 11:43:23 AM |

### Rectangle 10 (r6)

Position

| **Description** | **Value** |
| --- | --- |
| Position | {-w_tank/2, -(h_tank/2 + w_piezo + w_wall)} |

Size

| **Description** | **Value** |
| --- | --- |
| Width | w_tank |
| Height | w_piezo |

Information

| **Description** | **Value** |
| --- | --- |
| Last build time | < 1 second |
| Built with | COMSOL 6.2.0.415 (win64), Aug 21, 2024, 11:43:23 AM |

### Rectangle 11 (r7)

Position

| **Description** | **Value** |
| --- | --- |
| Position | {-(w_tank/2 + w_piezo + w_wall), -h_tank/2} |

Size

| **Description** | **Value** |
| --- | --- |
| Width | w_piezo |
| Height | h_tank |

Information

| **Description** | **Value** |
| --- | --- |
| Last build time | < 1 second |
| Built with | COMSOL 6.2.0.415 (win64), Aug 21, 2024, 11:43:23 AM |

### Rectangle 12 (r8)

Position

| **Description** | **Value** |
| --- | --- |
| Position | {-w_tank/2, (h_tank/2 + w_wall)} |

Size

| **Description** | **Value** |
| --- | --- |
| Width | w_tank |
| Height | w_piezo |

Information

| **Description** | **Value** |
| --- | --- |
| Last build time | < 1 second |
| Built with | COMSOL 6.2.0.415 (win64), Aug 21, 2024, 11:43:23 AM |

### Rectangle 13 (r9)

Position

| **Description** | **Value** |
| --- | --- |
| Position | {(w_tank/2 + w_wall), -h_tank/2} |

Size

| **Description** | **Value** |
| --- | --- |
| Width | w_piezo |
| Height | h_tank |

Information

| **Description** | **Value** |
| --- | --- |
| Last build time | < 1 second |
| Built with | COMSOL 6.2.0.415 (win64), Aug 21, 2024, 11:43:23 AM |

### Union 1 (uni2)

Information

| **Description** | **Value** |
| --- | --- |
| Last build time | < 1 second |
| Built with | COMSOL 6.2.0.415 (win64), Aug 21, 2024, 11:43:23 AM |

### Form Union (fin)

Information

| **Description** | **Value** |
| --- | --- |
| Details | {Formed union of 5 solid objects., Union has 6 domains, 32 boundaries, and 28 vertices.} |
| Last build time | < 1 second |
| Built with | COMSOL 6.2.0.415 (win64), Aug 21, 2024, 11:43:23 AM |

## Materials

### PMMA - Polymethyl methacrylate


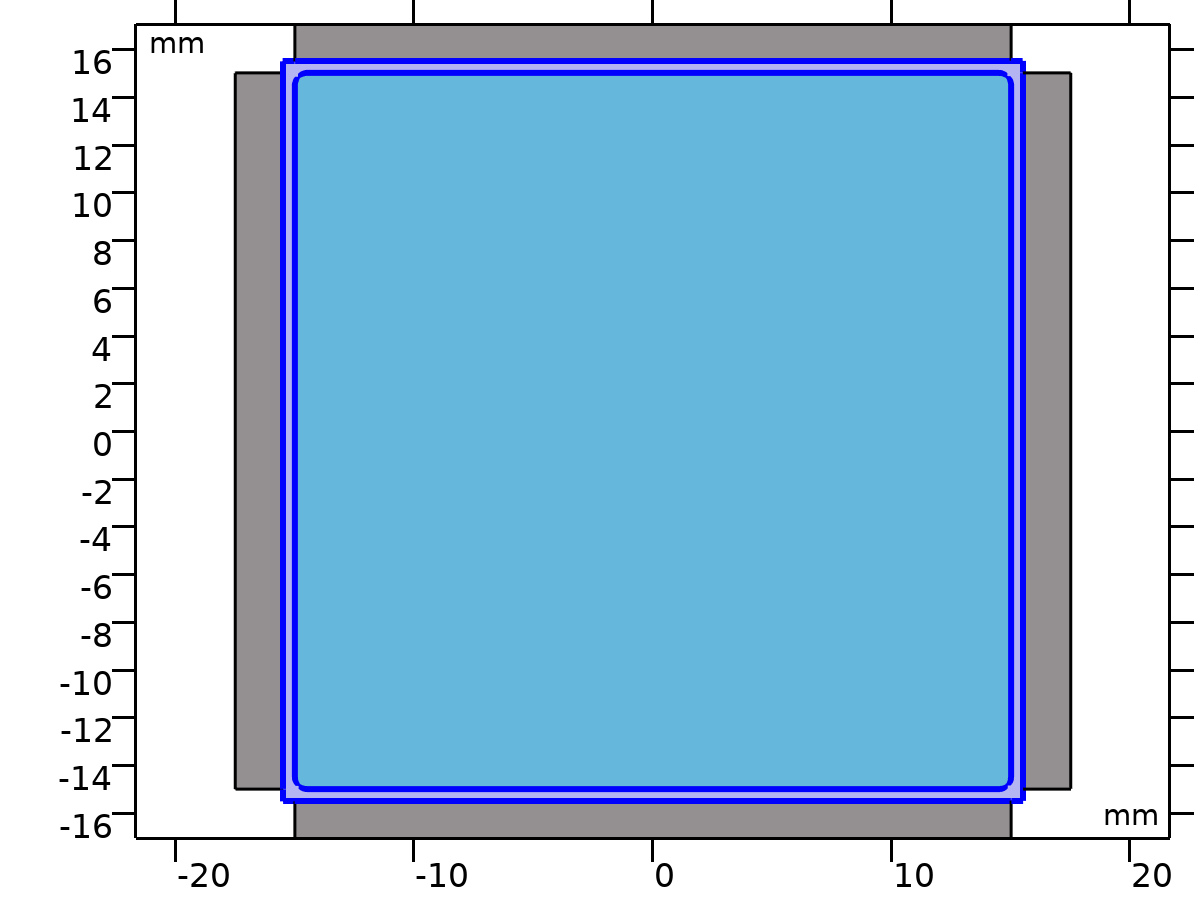


PMMA - Polymethyl methacrylate

Selection

| Geometric entity level | Domain |
| --- | --- |
| Name | PMMA domains |
| Selection | Named sel2: Geometry geom1: Dimension 2: Domain 2 |

Material parameters

| **Name** | **Value** | **Unit** | **Property group** |
| --- | --- | --- | --- |
| Density | 1190 | kg/m³ | Basic |
| Young's modulus | 3E9 | Pa | Young's modulus and Poisson's ratio |
| Poisson's ratio | 0.4 | 1 | Young's modulus and Poisson's ratio |

Basic

| **Description** | **Value** | **Unit** |
| --- | --- | --- |
| Density | 1190 | kg/m³ |

Young's modulus and Poisson's ratio

| **Description** | **Value** | **Unit** |
| --- | --- | --- |
| Young's modulus | 3E9 | Pa |
| Poisson's ratio | 0.4 | 1 |

### Lead Zirconate Titanate (PZT-5H)


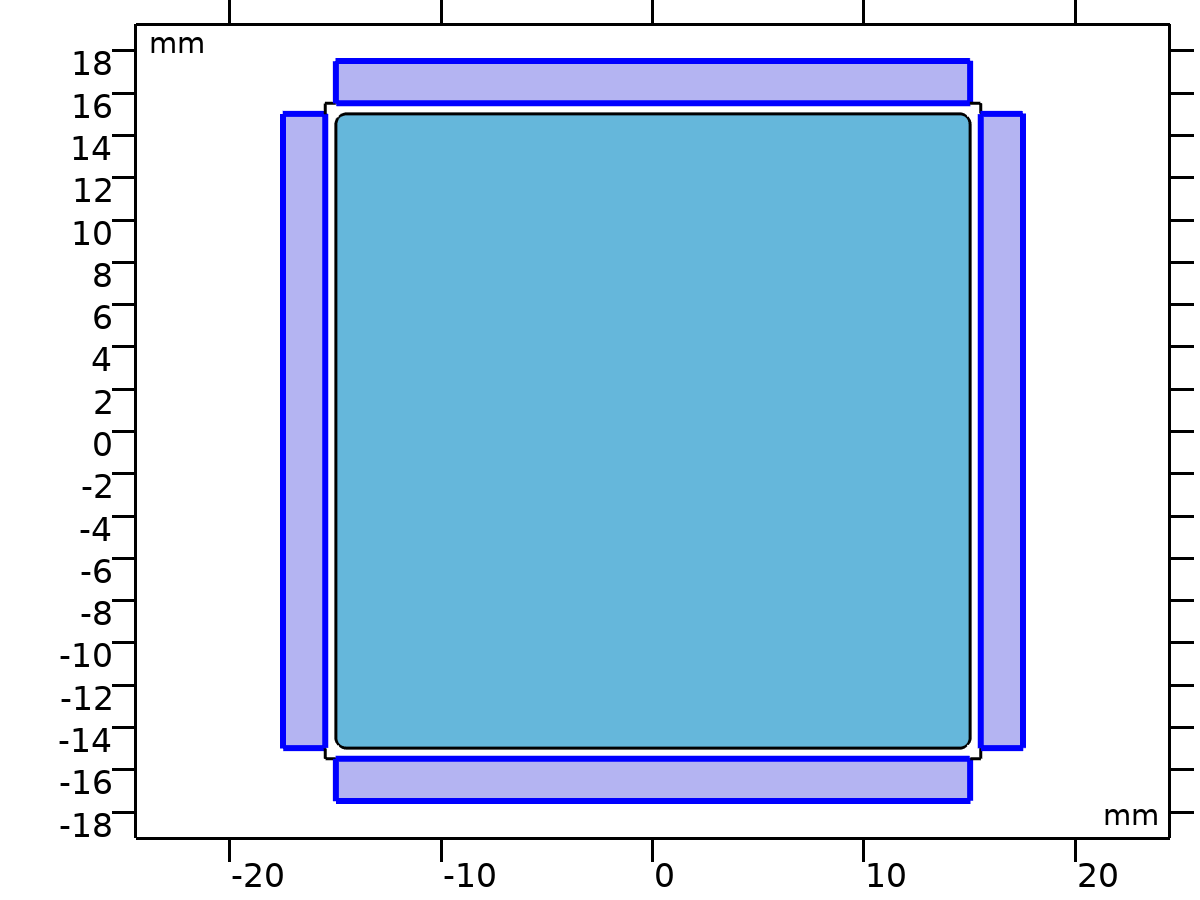


Lead Zirconate Titanate (PZT-5H)

Selection

| Geometric entity level | Domain |
| --- | --- |
| Name | PZT domains |
| Selection | Named sel3: Geometry geom1: Dimension 2: Domains 1, 3, 5–6 |

Material parameters

| **Name** | **Value** | **Unit** | **Property group** |
| --- | --- | --- | --- |
| Density | 7500 | kg/m³ | Basic |
| Elasticity matrix, Voigt notation | {1.2721E11, 8.0212E10, 1.2721E11, 8.467E10, 8.467E10, 1.1744E11, 0, 0, 0, 2.2989E10, 0, 0, 0, 0, 2.2989E10, 0, 0, 0, 0, 0, 2.3474E10} | Pa | Stress-charge form |
| Coupling matrix, Voigt notation | {0, 0, -6.6228, 0, 0, -6.6228, 0, 0, 23.24, 0, 17.035, 0, 17.035, 0, 0, 0, 0, 0} | C/m² | Stress-charge form |
| Relative permittivity | {1704.4, 1704.4, 1433.6} | 1 | Stress-charge form |

Basic

| **Description** | **Value** | **Unit** |
| --- | --- | --- |
| Density | 7500 | kg/m³ |

Stress-charge form

| **Description** | **Value** | **Unit** |
| --- | --- | --- |
| Elasticity matrix, Voigt notation | {1.2721E11, 8.0212E10, 8.467E10, 0, 0, 0, 8.0212E10, 1.2721E11, 8.467E10, 0, 0, 0, 8.467E10, 8.467E10, 1.1744E11, 0, 0, 0, 0, 0, 0, 2.2989E10, 0, 0, 0, 0, 0, 0, 2.2989E10, 0, 0, 0, 0, 0, 0, 2.3474E10} | Pa |
| Coupling matrix, Voigt notation | {0, 0, -6.6228, 0, 0, -6.6228, 0, 0, 23.24, 0, 17.035, 0, 17.035, 0, 0, 0, 0, 0} | C/m² |
| Relative permittivity | {1704.4, 1704.4, 1433.6} | 1 |

### Water, liquid


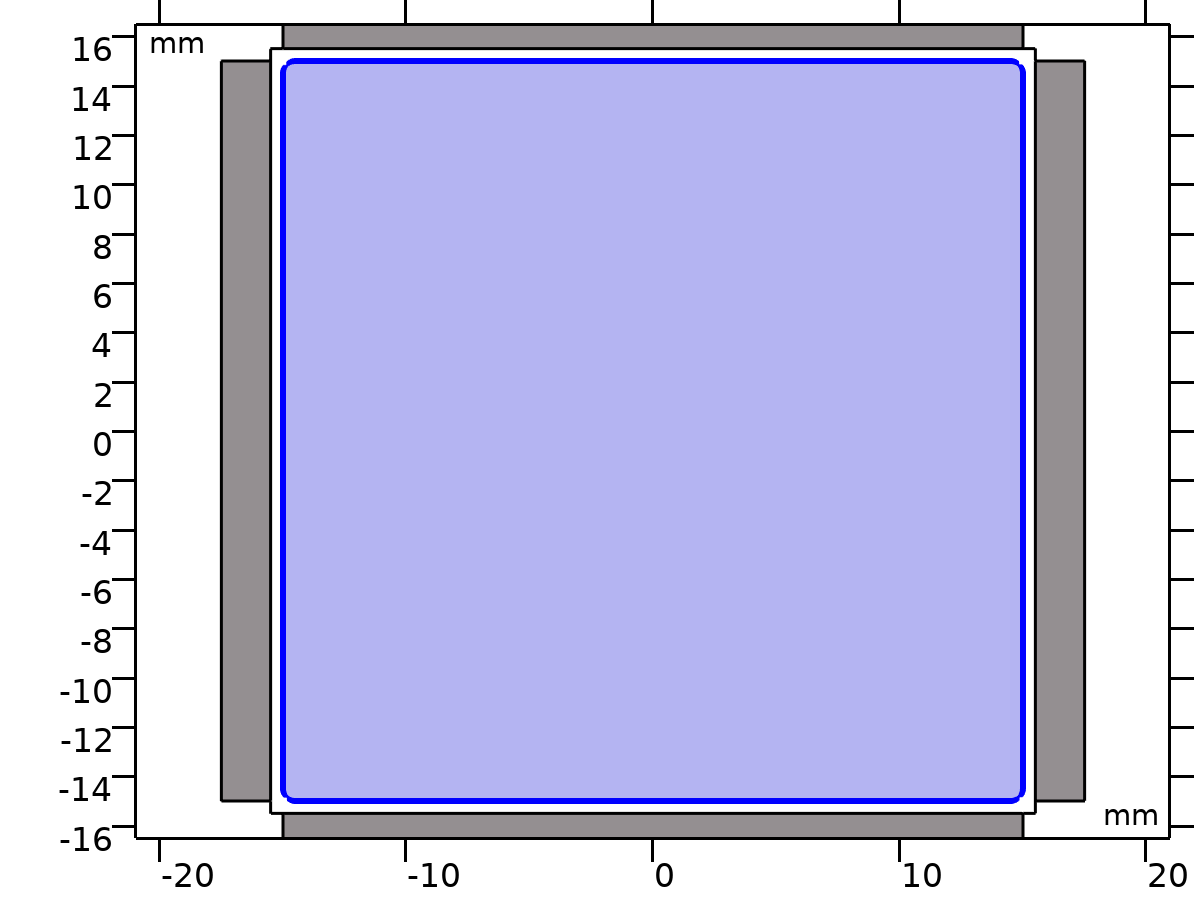


Water, liquid

Selection

| Geometric entity level | Domain |
| --- | --- |
| Name | Water domains |
| Selection | Named sel1: Geometry geom1: Dimension 2: Domain 4 |

Material parameters

| **Name** | **Value** | **Unit** | **Property group** |
| --- | --- | --- | --- |
| Bulk viscosity | muB(T) | Pa·s | Basic |
| Dynamic viscosity | eta(T) | Pa·s | Basic |
| Density | rho(T) | kg/m³ | Basic |
| Speed of sound | cs(T) | m/s | Basic |

Basic

| **Description** | **Value** | **Unit** |
| --- | --- | --- |
| Bulk viscosity | muB(T) | Pa·s |
| Dynamic viscosity | eta(T) | Pa·s |
| Density | rho(T) | kg/m³ |
| Speed of sound | cs(T) | m/s |

Functions

| **Function name** | **Type** |
| --- | --- |
| eta | Piecewise |
| Cp | Piecewise |
| rho | Piecewise |
| k | Piecewise |
| cs | Interpolation |
| alpha_p | Analytic |
| gamma_w | Analytic |
| muB | Analytic |

#### Piecewise


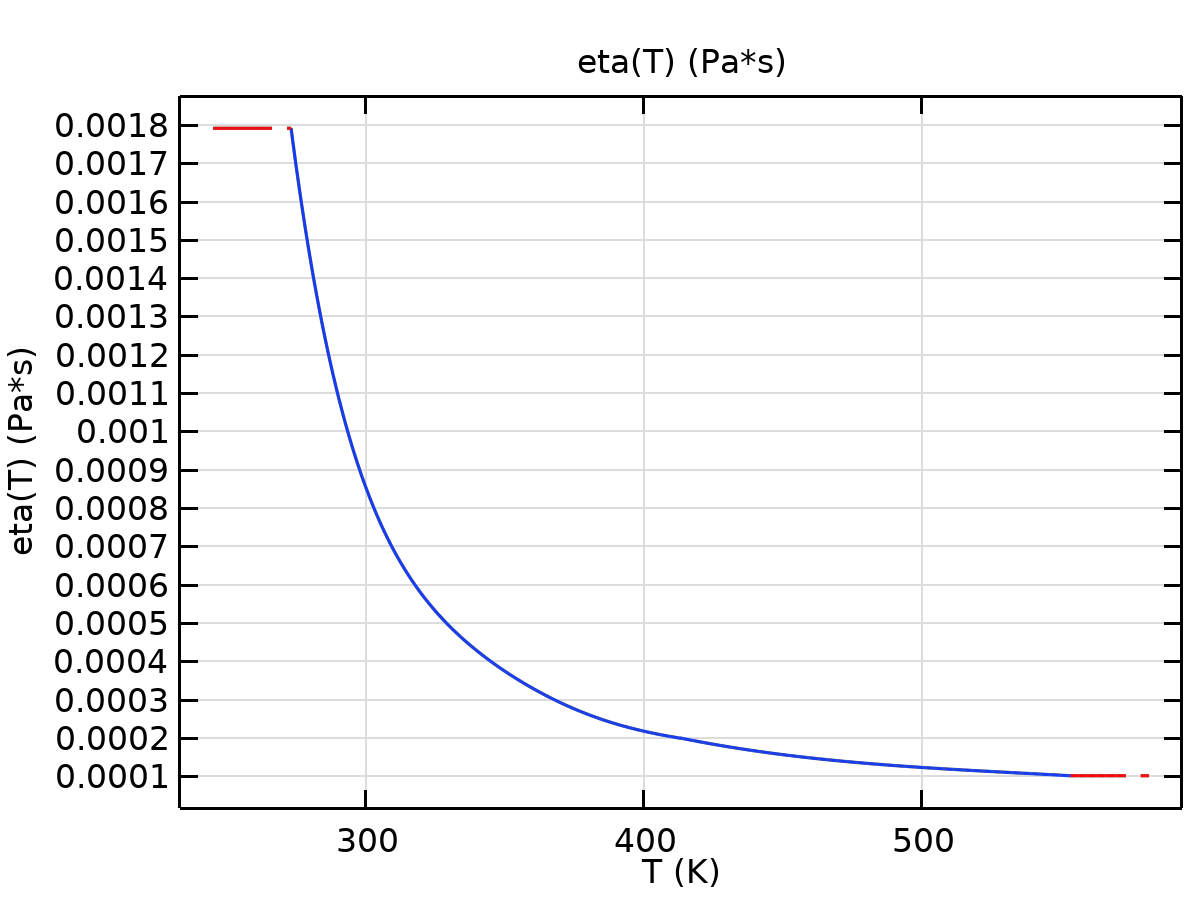


eta

#### Piecewise 2


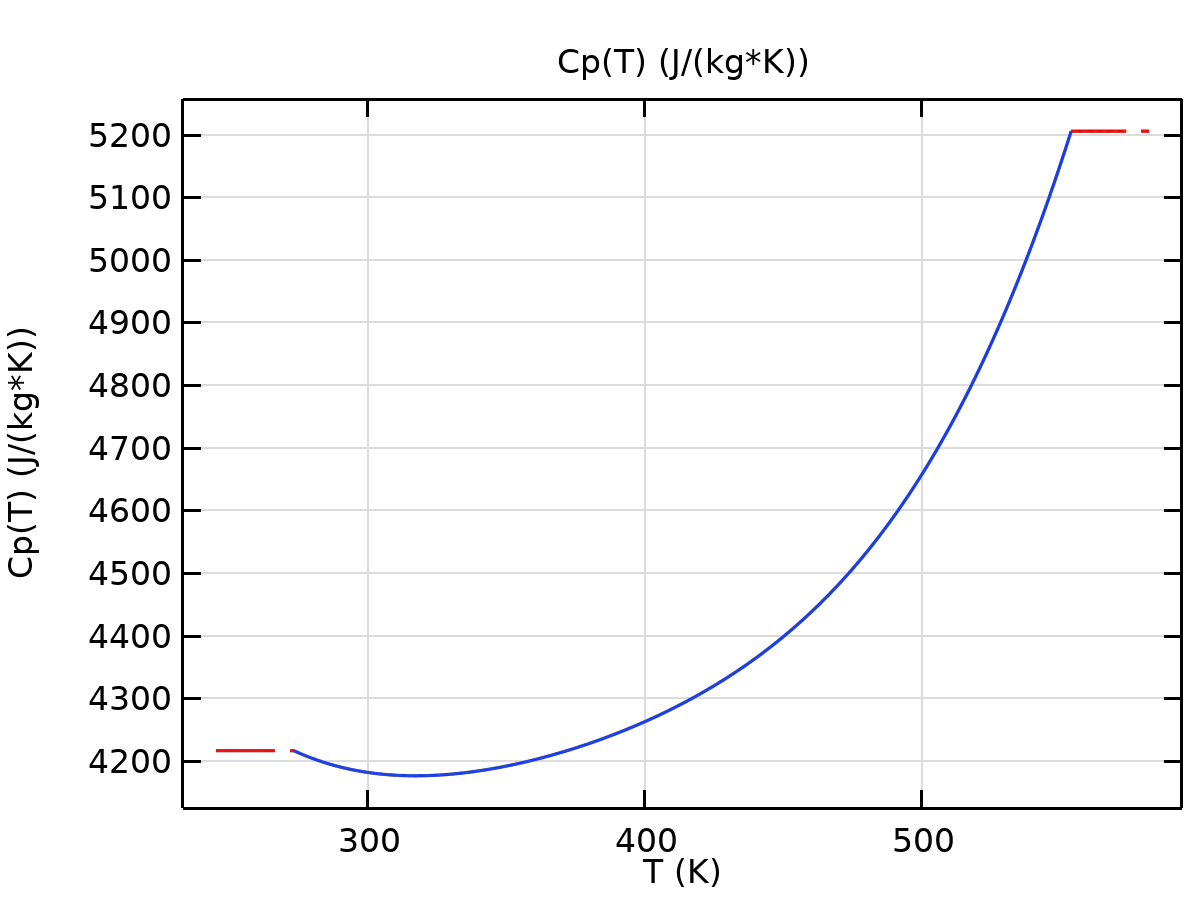


Cp

#### Piecewise 3


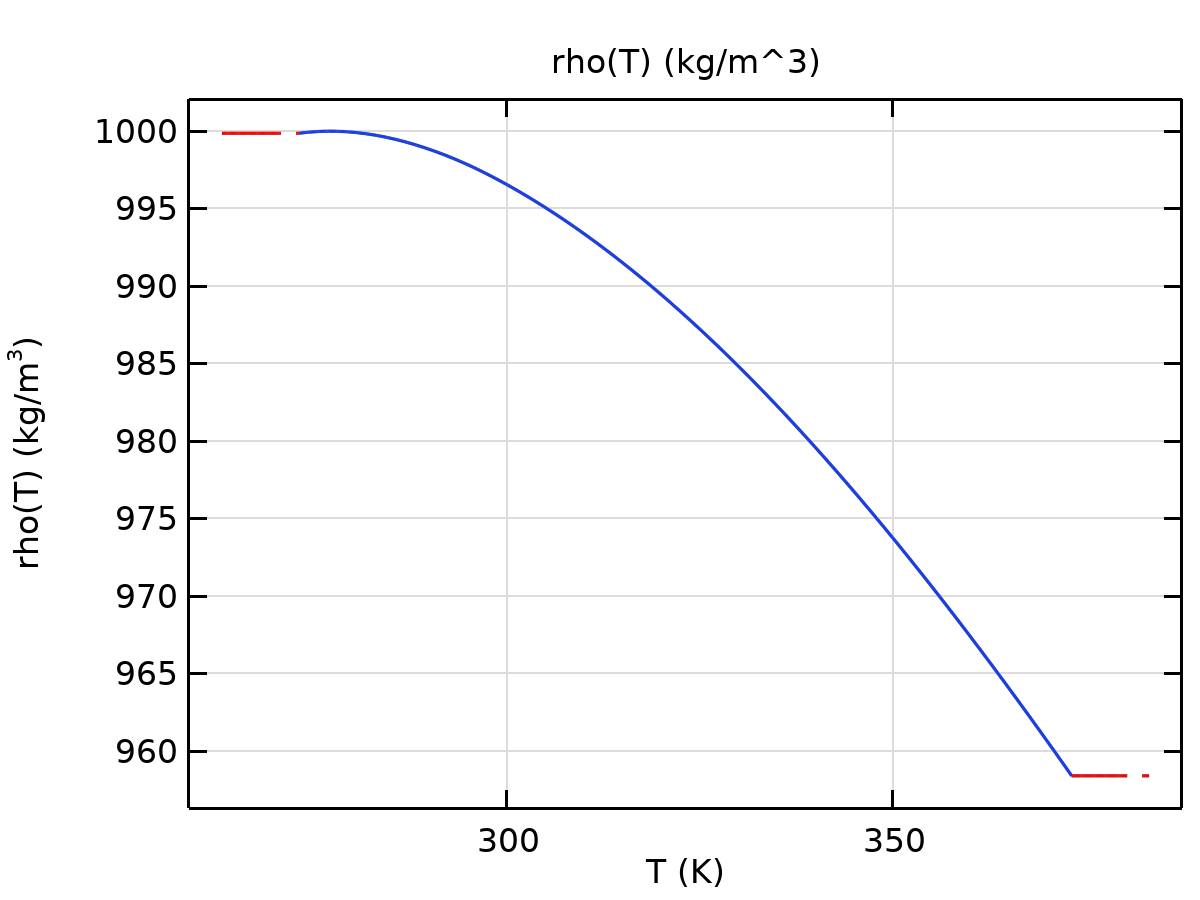


rho

#### Piecewise 4


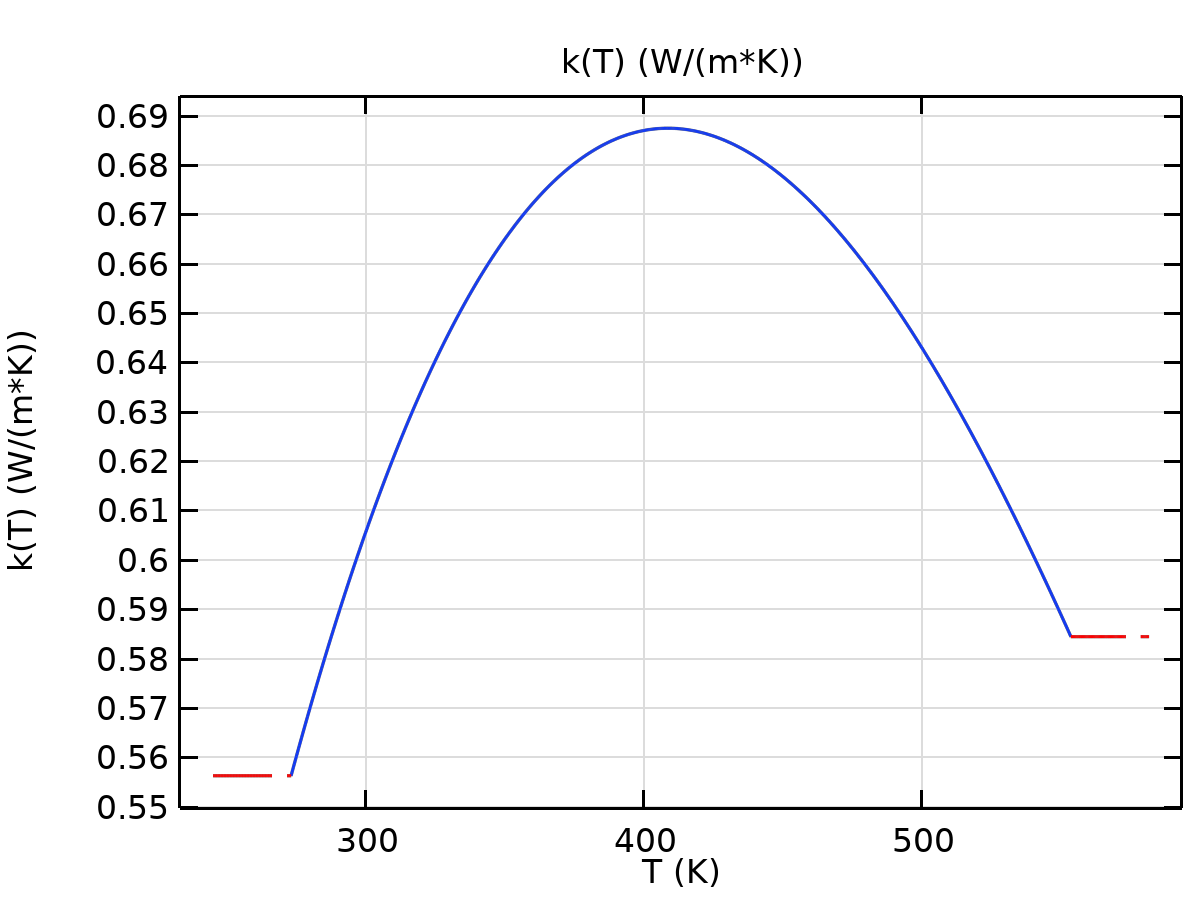


k

#### Interpolation


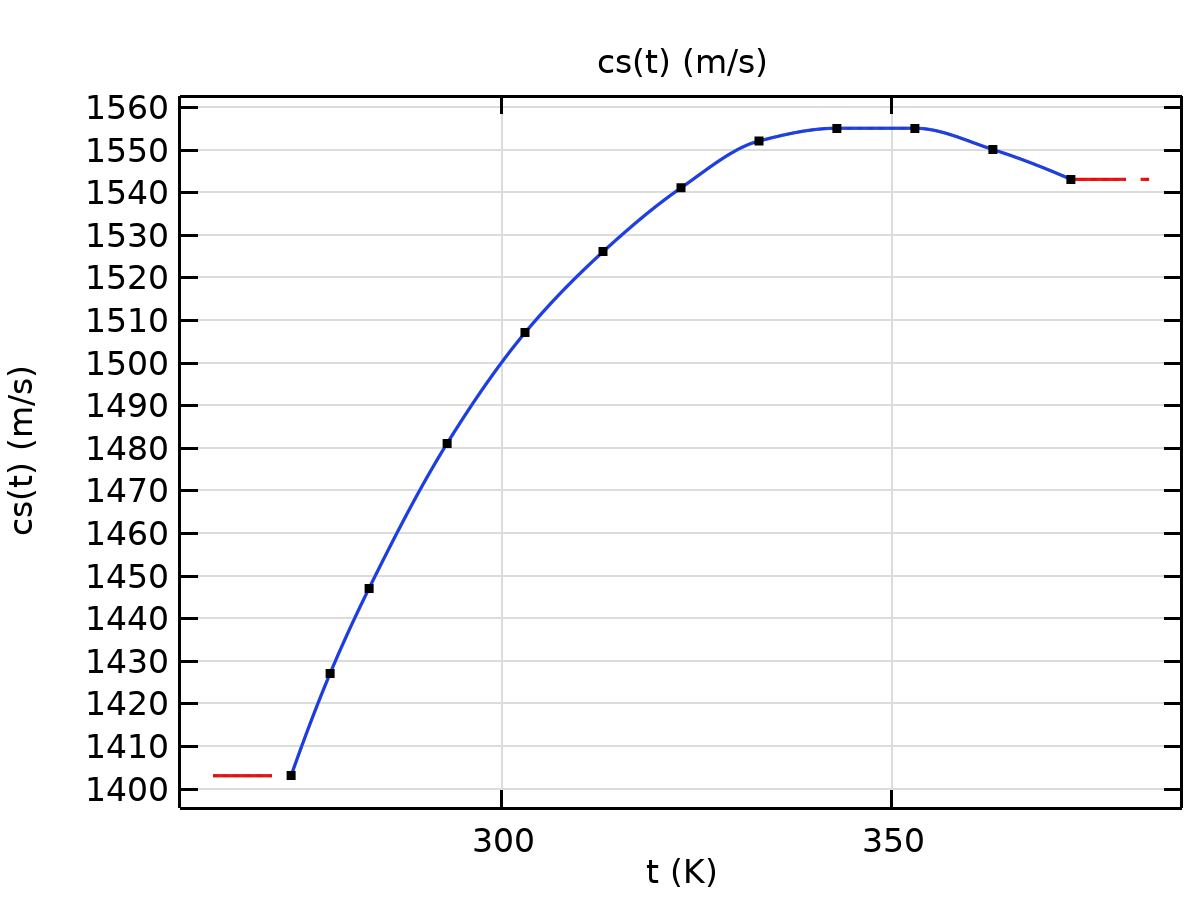


cs

#### Analytic 1


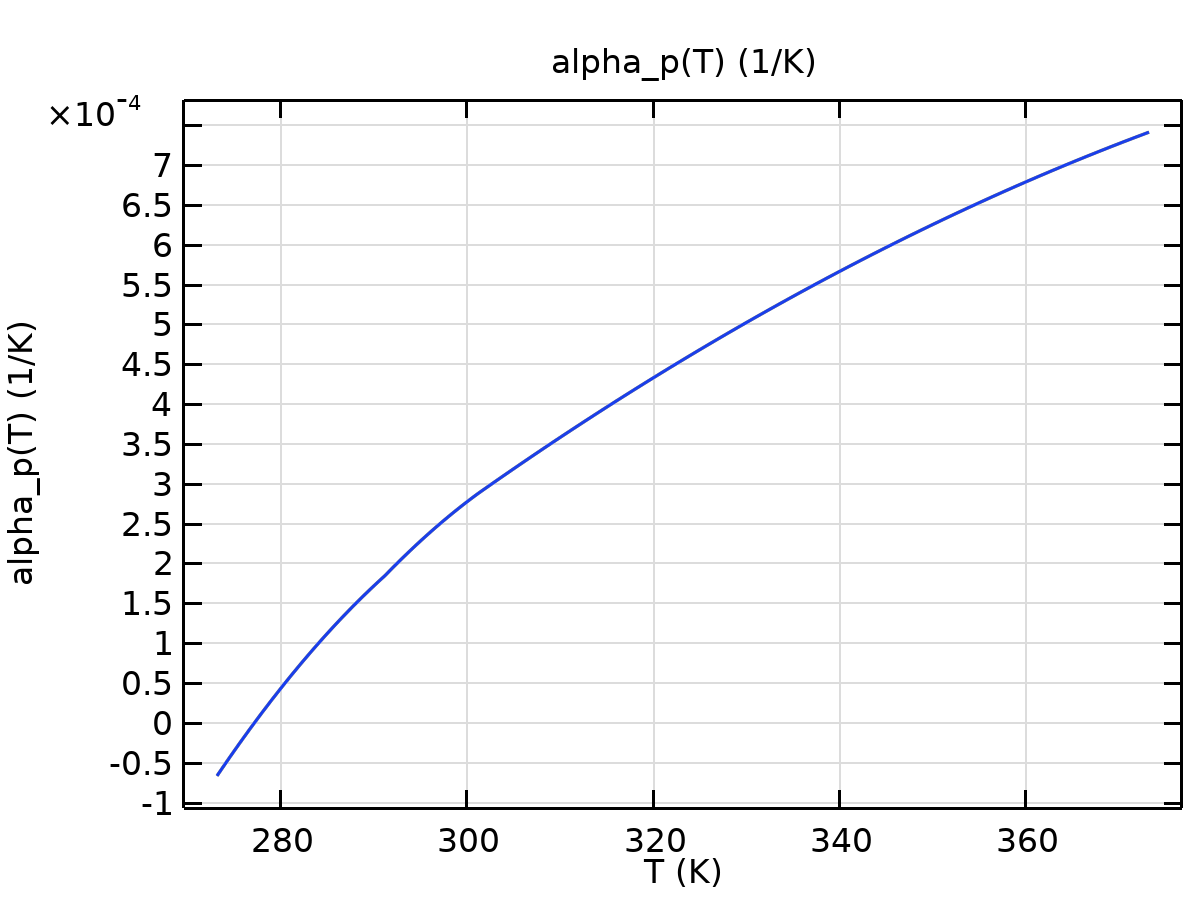


alpha_p

#### Analytic 2


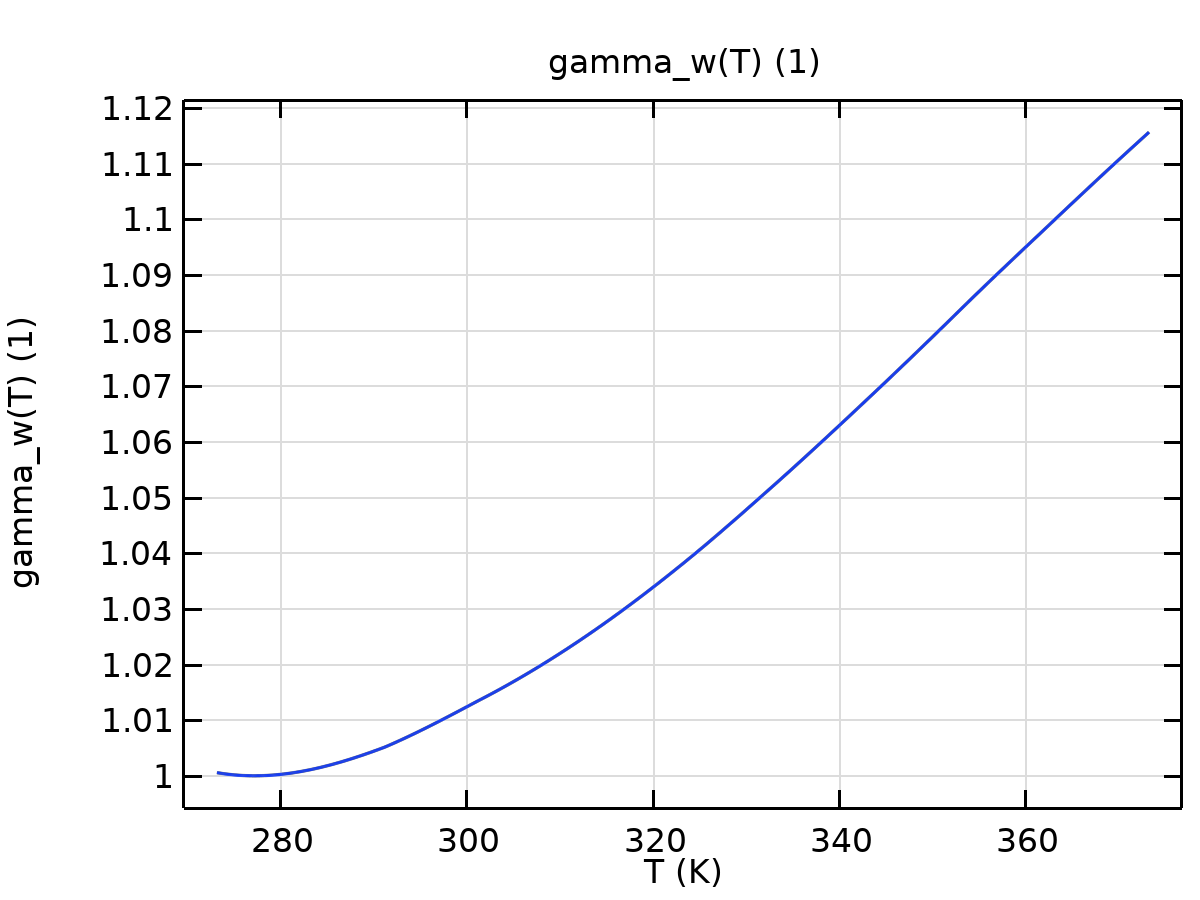


gamma_w

#### Analytic 3


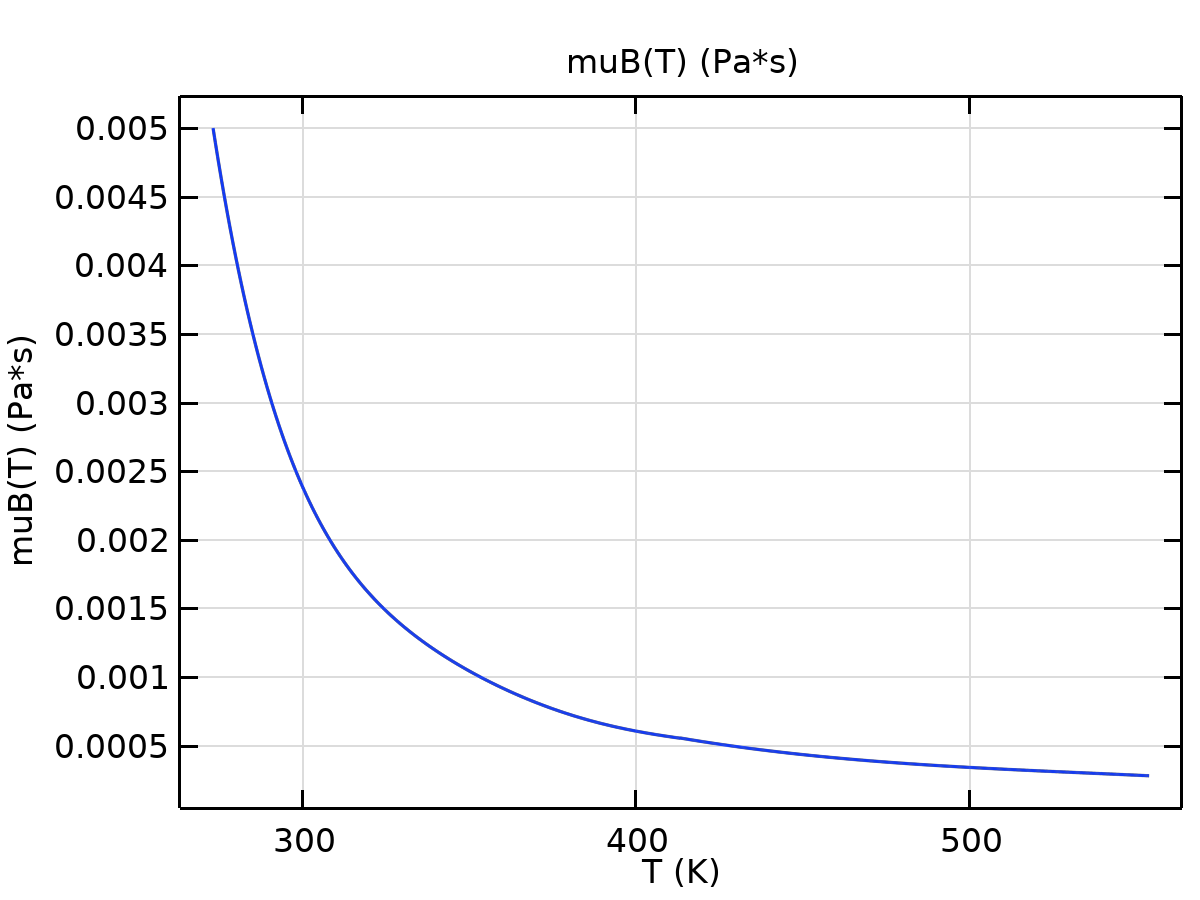


muB

### Polystyrene (PS) [solid]


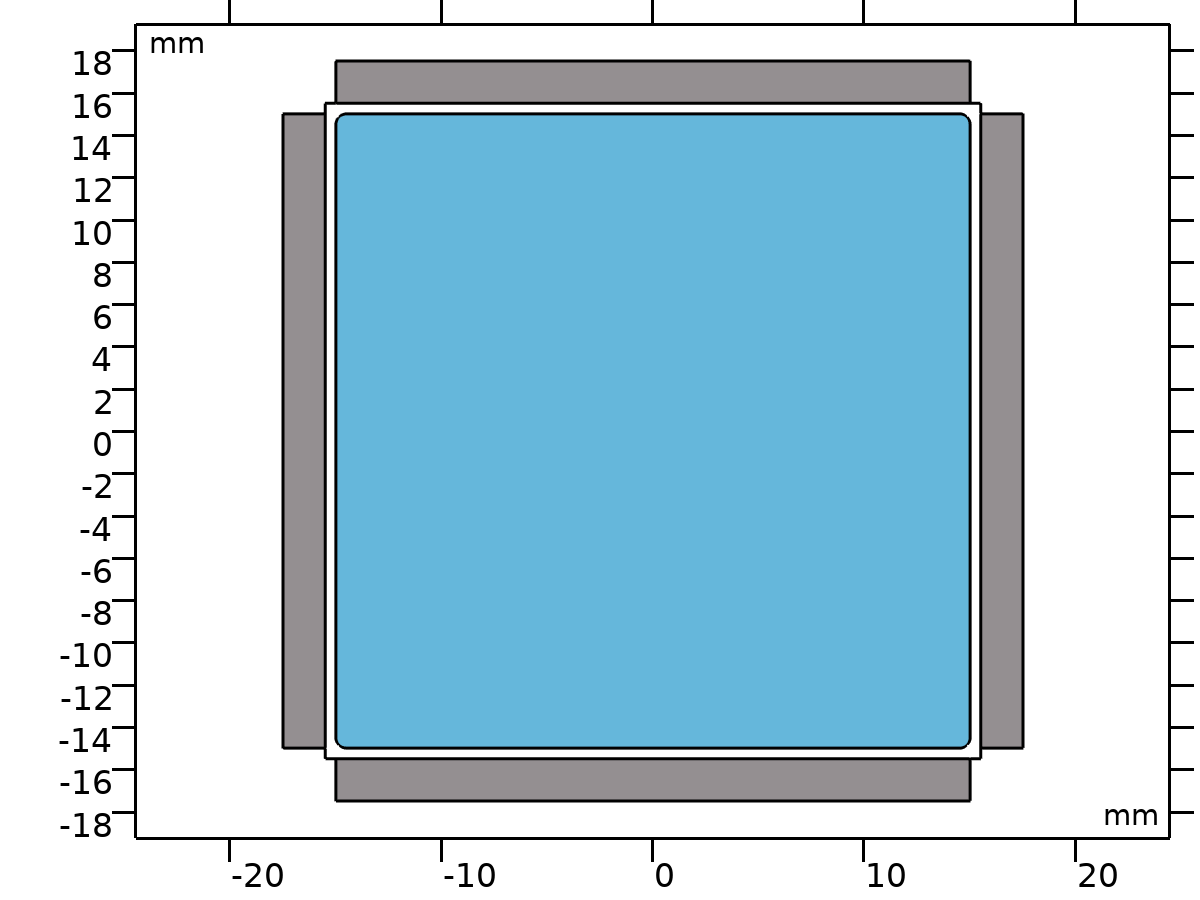


Polystyrene (PS) [solid]

Selection

| Geometric entity level | Domain |
| --- | --- |
| Selection | Geometry geom1: Dimension 2: No domains |

Material parameters

| **Name** | **Value** | **Unit** | **Property group** |
| --- | --- | --- | --- |
| Density | 1000 | kg/m³ | Basic |

Basic

| **Description** | **Value** | **Unit** |
| --- | --- | --- |
| Density | 1000 | kg/m³ |

Functions

| **Function name** | **Type** |
| --- | --- |
| TD | Piecewise |

#### Piecewise


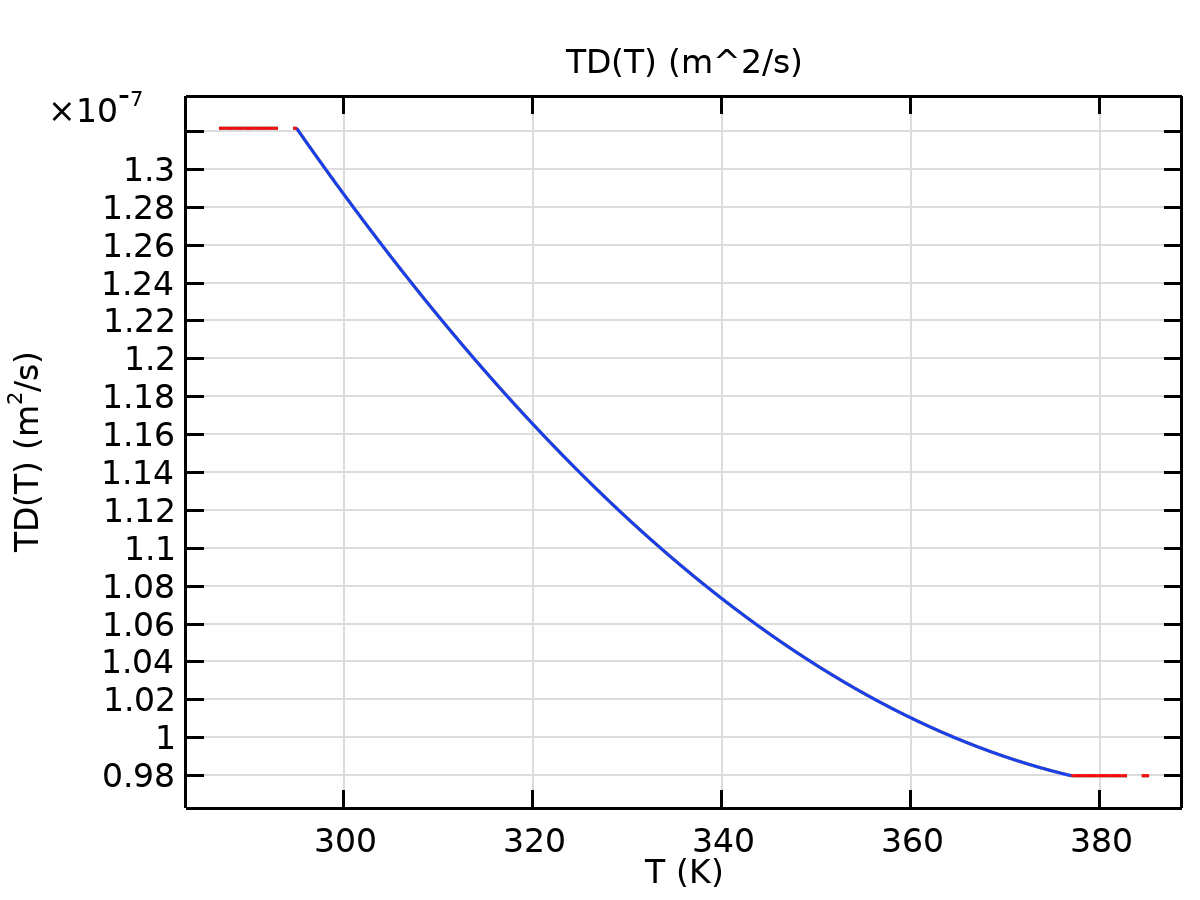


TD

Functions

| **Function name** | **Type** |
| --- | --- |
| E | Piecewise |
| nu | Piecewise |

#### Piecewise


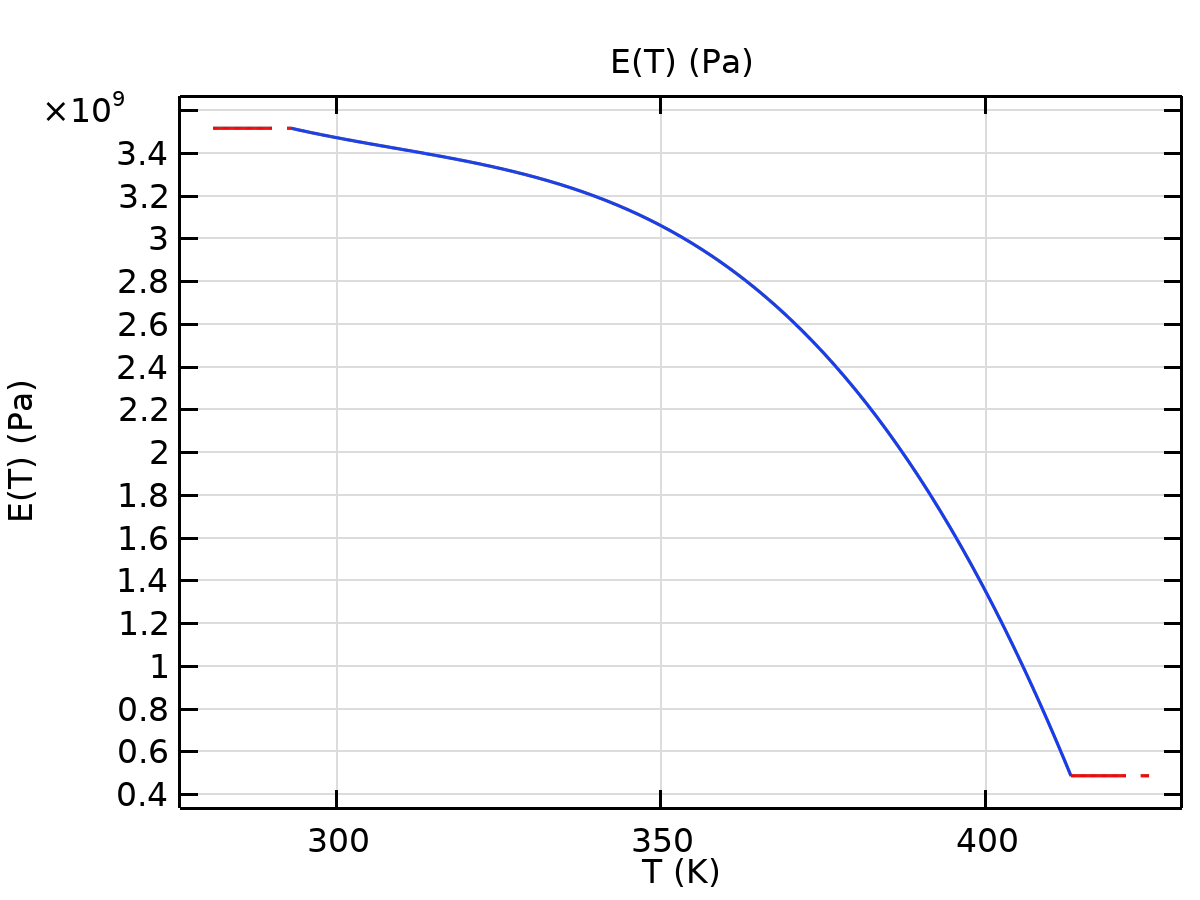


E

#### Piecewise 1


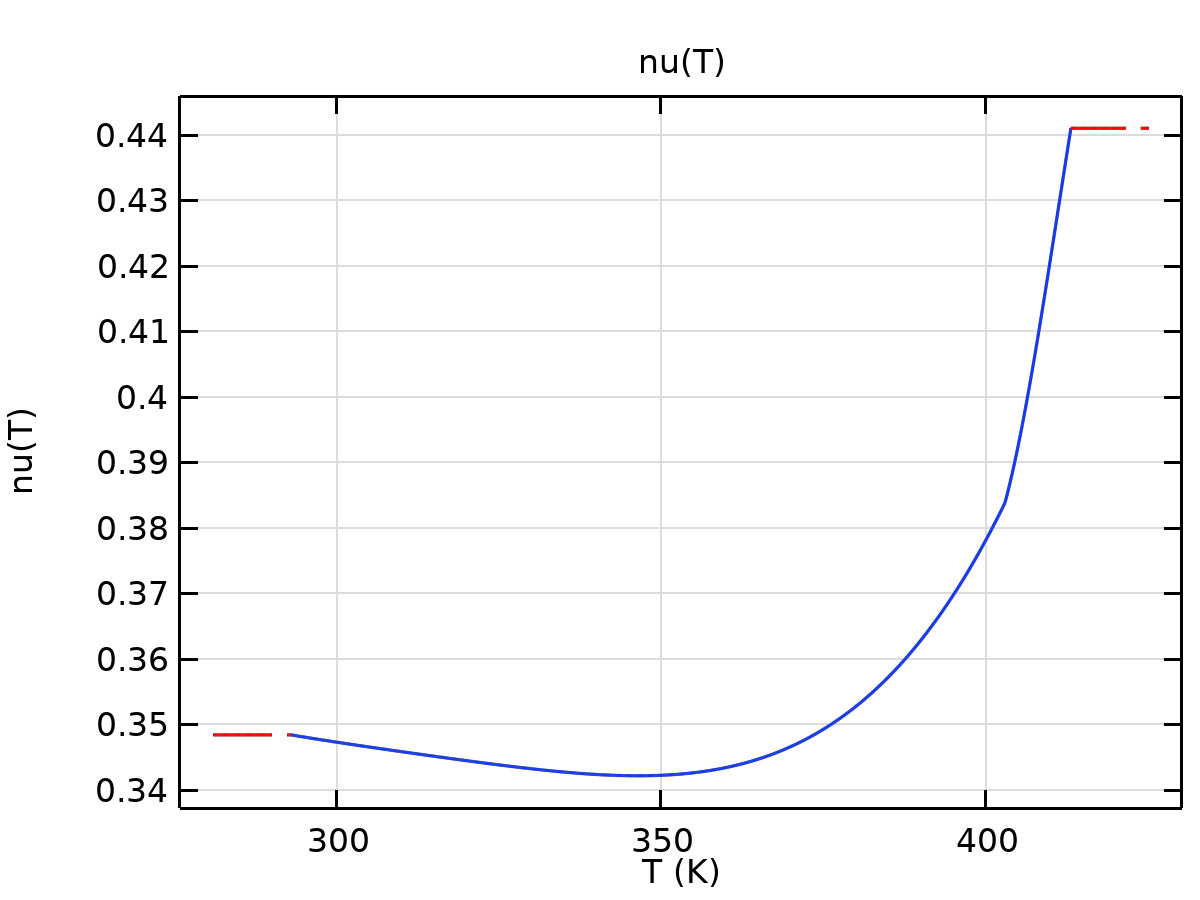


nu

Functions

| **Function name** | **Type** |
| --- | --- |
| mu | Piecewise |
| kappa | Piecewise |

#### Piecewise


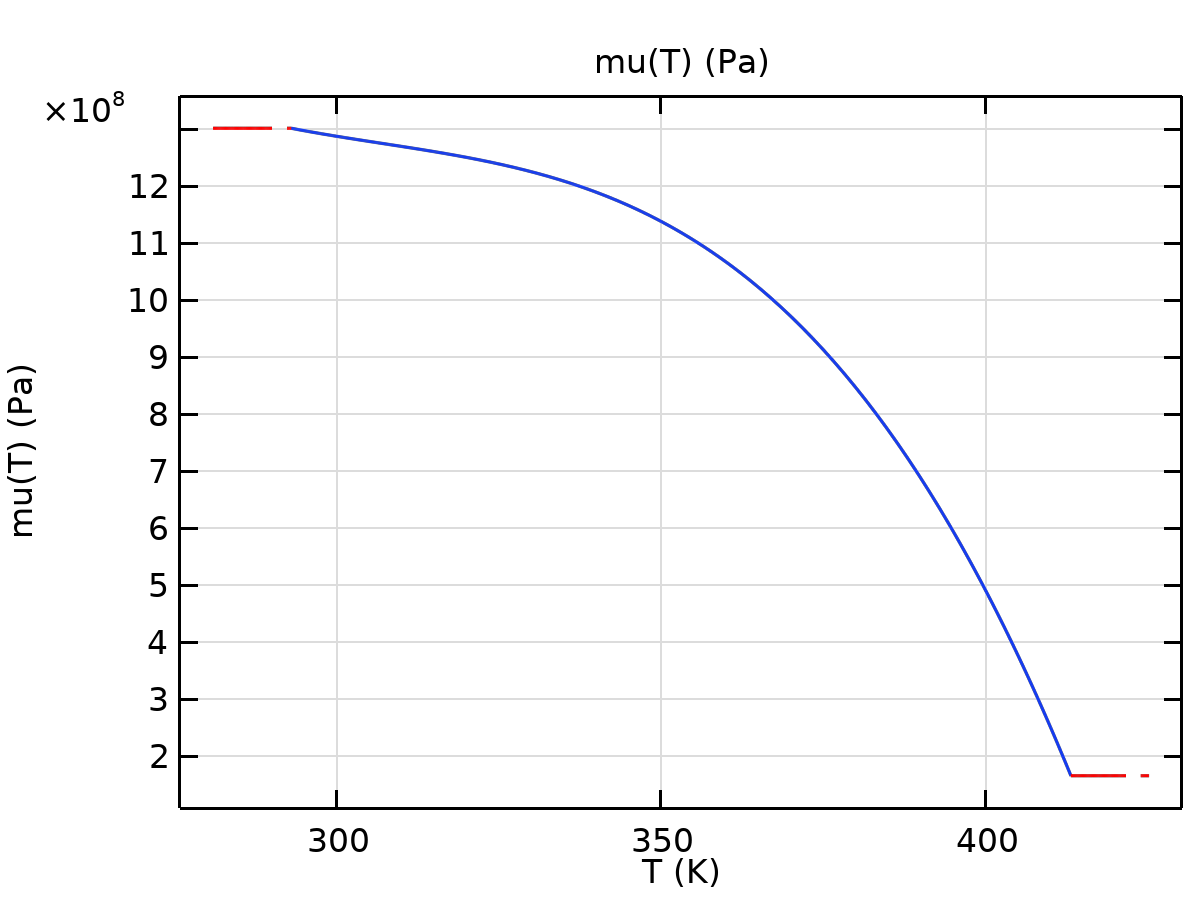


mu

#### Piecewise 1


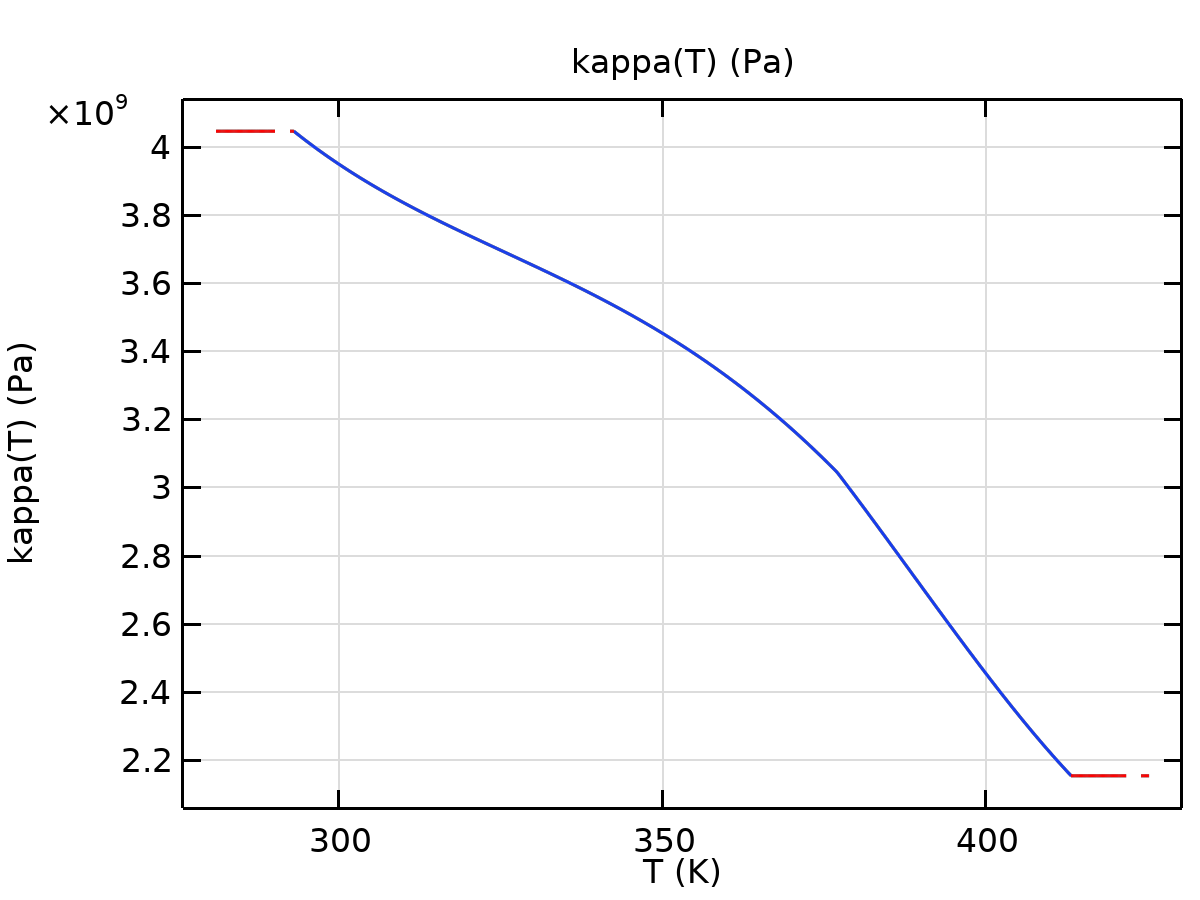


kappa

## Pressure Acoustics, Frequency Domain

Used products

| Acoustics Module |
| --- |
| COMSOL Multiphysics |


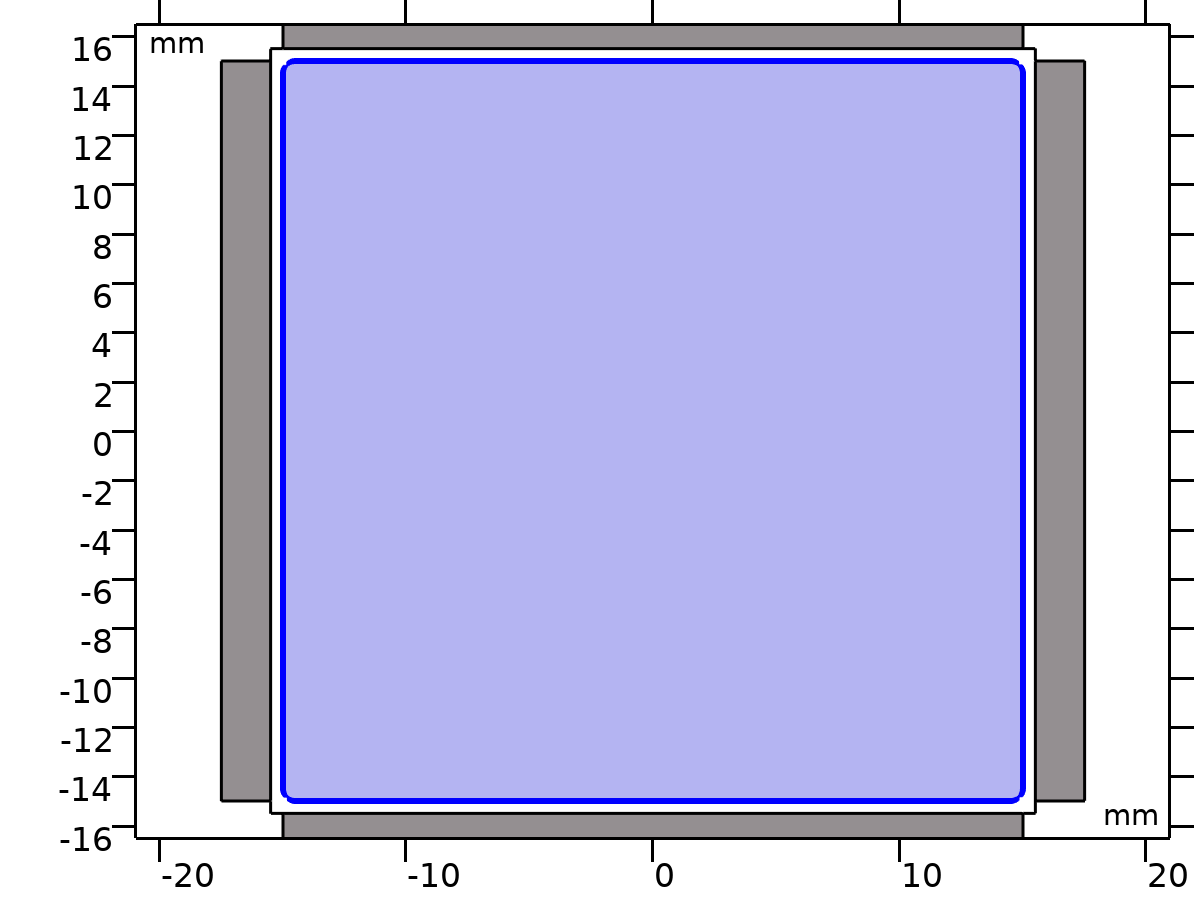


Pressure Acoustics, Frequency Domain

Selection

| Geometric entity level | Domain |
| --- | --- |
| Name | Water domains |
| Selection | Named sel1: Geometry geom1: Dimension 2: Domain 4 |

Equations


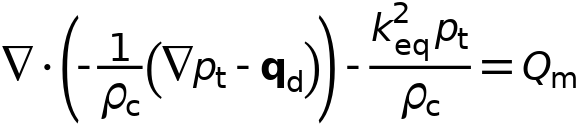


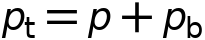


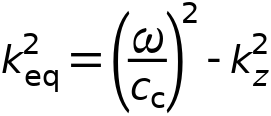


### Interface Settings

#### Physics Symbols

Settings

| **Description** | **Value** |
| --- | --- |
| Enable physics symbols | On |

#### Discretization

Settings

| **Description** | **Value** |
| --- | --- |
| Element order | Quadratic Lagrange |
| Compute boundary fluxes | Off |
| Value type when using splitting of complex variables | Complex |

#### Physics-Controlled Mesh

Settings

| **Description** | **Value** |
| --- | --- |
| Maximum mesh element size control parameter | From study |
| Number of mesh elements per wavelength | Automatic |

#### Pressure Acoustics Equation Settings

Settings

| **Description** | **Value** | **Unit** |
| --- | --- | --- |
| Out-of-plane wave number | 0 | rad/m |

#### Global Port Settings

Settings

| **Description** | **Value** |
| --- | --- |
| Port sweep settings | No port sweep |
| Mode shape normalization | Amplitude normalization |

#### Sound Pressure Level Settings

Settings

| **Description** | **Value** |
| --- | --- |
| Reference pressure for the sound pressure level | Use reference pressure for water |

#### Typical Wave Speed for Perfectly Matched Layers

Settings

| **Description** | **Value** | **Unit** |
| --- | --- | --- |
| Typical wave speed for perfectly matched layers | real(acpr.c_c) | m/s |

### Variables

| **Name** | **Expression** | **Unit** | **Description** | **Selection** |
| --- | --- | --- | --- | --- |
| acpr.freq | freq | Hz | Frequency | Global |
| acpr.omega | 2*pi*acpr.freq | rad/s | Angular frequency | Global |
| acpr.kz | 0 | rad/m | Out-of-plane wave number | Global |
| acpr.ikz | acpr.kz*i | rad/m | Phase-shifted out-of-plane wave number | Global |
| acpr.delta | 1/acpr.omega^2 | s² | Scaling factor | Domain 4 |
| acpr.pref_SPL | 1.0E-6[Pa] | Pa | Reference pressure | Global |
| acpr.Iref_SIL | 1.0E-12[W/m^2] | W/m² | Reference intensity | Global |
| acpr.Pref_SWL | 1.0E-12[W] | W | Reference power | Global |
| acpr.cref | real(acpr.c_c) | m/s | Typical wave speed for perfectly matched layers | Domain 4 |
| acpr.nx | dnx | 1 | Normal vector, x-component | Boundaries 12, 16–17, 20, 29–32 |
| acpr.ny | dny | 1 | Normal vector, y-component | Boundaries 12, 16–17, 20, 29–32 |
| acpr.nz | 0 | 1 | Normal vector, z-component | Boundaries 12, 16–17, 20, 29–32 |
| acpr.nxmesh | dnxmesh | 1 | Normal vector (mesh), x-component | Boundaries 12, 16–17, 20, 29–32 |
| acpr.nymesh | dnymesh | 1 | Normal vector (mesh), y-component | Boundaries 12, 16–17, 20, 29–32 |
| acpr.nzmesh | 0 | 1 | Normal vector (mesh), z-component | Boundaries 12, 16–17, 20, 29–32 |
| acpr.iomega | acpr.omega*i | rad/s | Complex angular frequency | Global |

### Pressure Acoustics 1


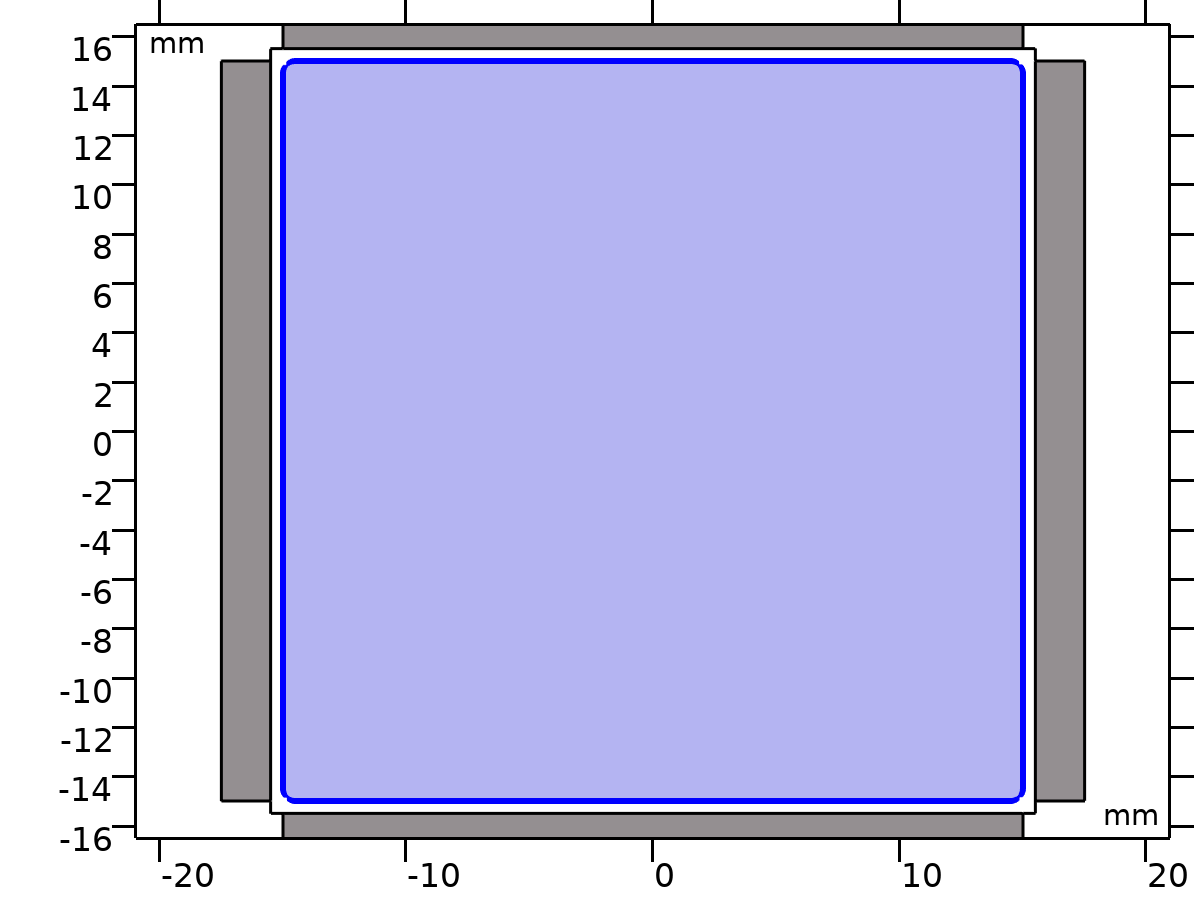


Pressure Acoustics 1

Selection

| Geometric entity level | Domain |
| --- | --- |
| Selection | Geometry geom1: Dimension 2: All domains |

Equations


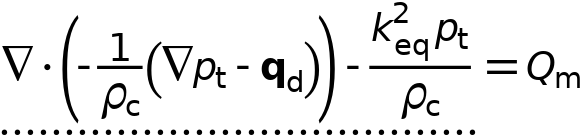


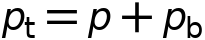


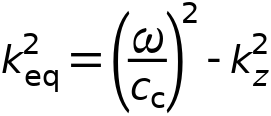


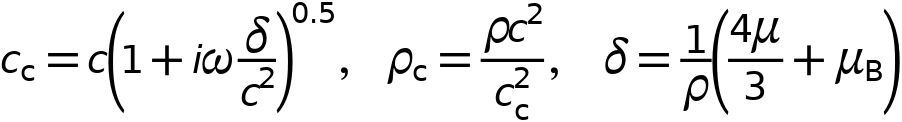


#### Pressure Acoustics Model

Settings

| **Description** | **Value** |
| --- | --- |
| Fluid model | Viscous |
| Speed of sound | From material |
| Density | From material |
| Dynamic viscosity | From material |
| Bulk viscosity | From material |

#### Model Input

Settings

| **Description** | **Value** | **Unit** |
| --- | --- | --- |
| Temperature | Common model input |  |
| Absolute pressure | User defined |  |
| Absolute pressure | 1.0133E5 | Pa |

Properties from material

| **Property** | **Material** | **Property group** |
| --- | --- | --- |
| Density | Water, liquid | Basic |
| Speed of sound | Water, liquid | Basic |
| Dynamic viscosity | Water, liquid | Basic |
| Bulk viscosity | Water, liquid | Basic |
| Density | Polystyrene (PS) [solid] | Basic |

#### Variables

| **Name** | **Expression** | **Unit** | **Description** | **Selection** | **Details** |
| --- | --- | --- | --- | --- | --- |
| acpr.mu | material.mu | Pa·s | Dynamic viscosity | Domain 4 | Meta |
| acpr.q_totx | acpr.qx |  | Total dipole source, x-component | Domain 4 | + operation |
| acpr.q_toty | acpr.qy |  | Total dipole source, y-component | Domain 4 | + operation |
| acpr.q_totz | acpr.qz |  | Total dipole source, z-component | Domain 4 | + operation |
| acpr.qx | 0 |  | Dipole domain source, x-component | Domain 4 | + operation |
| acpr.qy | 0 |  | Dipole domain source, y-component | Domain 4 | + operation |
| acpr.qz | 0 |  | Dipole domain source, z-component | Domain 4 | + operation |
| acpr.p_s | acpr.p_t-acpr.p_b | Pa | Scattered acoustic pressure | Domain 4 |  |
| acpr.p_b | 0 | Pa | Background acoustic pressure | Domain 4 | + operation |
| acpr.Lp_s | 20*log10(acpr.p_s_rms/acpr.pref_SPL) | dB | Scattered sound pressure level | Domain 4 |  |
| acpr.Lp_b | 20*log10(acpr.p_b_rms/acpr.pref_SPL) | dB | Background sound pressure level | Domain 4 |  |
| acpr.Ix | 0.5*realdot(acpr.p_t,acpr.vx) | W/m² | Intensity, x-component | Domain 4 |  |
| acpr.Iy | 0.5*realdot(acpr.p_t,acpr.vy) | W/m² | Intensity, y-component | Domain 4 |  |
| acpr.Iz | 0.5*realdot(acpr.p_t,acpr.vz) | W/m² | Intensity, z-component | Domain 4 |  |
| acpr.ax | -(acpr.gradpx-acpr.q_totx)/acpr.rho_c | m/s² | Total acoustic acceleration, x-component | Domain 4 |  |
| acpr.ay | -(acpr.gradpy-acpr.q_toty)/acpr.rho_c | m/s² | Total acoustic acceleration, y-component | Domain 4 |  |
| acpr.az | -(acpr.gradpz-acpr.q_totz)/acpr.rho_c | m/s² | Total acoustic acceleration, z-component | Domain 4 |  |
| acpr.a_tx | acpr.ax | m/s² | Total acoustic acceleration, x-component | Domain 4 |  |
| acpr.a_ty | acpr.ay | m/s² | Total acoustic acceleration, y-component | Domain 4 |  |
| acpr.a_tz | acpr.az | m/s² | Total acoustic acceleration, z-component | Domain 4 |  |
| acpr.vx | -(acpr.gradpx-acpr.q_totx)/(acpr.rho_c*acpr.iomega) | m/s | Total acoustic velocity, x-component | Domain 4 |  |
| acpr.vy | -(acpr.gradpy-acpr.q_toty)/(acpr.rho_c*acpr.iomega) | m/s | Total acoustic velocity, y-component | Domain 4 |  |
| acpr.vz | -(acpr.gradpz-acpr.q_totz)/(acpr.rho_c*acpr.iomega) | m/s | Total acoustic velocity, z-component | Domain 4 |  |
| acpr.v_tx | acpr.vx | m/s | Total acoustic velocity, x-component | Domain 4 |  |
| acpr.v_ty | acpr.vy | m/s | Total acoustic velocity, y-component | Domain 4 |  |
| acpr.v_tz | acpr.vz | m/s | Total acoustic velocity, z-component | Domain 4 |  |
| acpr.gradpx | d(acpr.p_t,x) | N/m³ | Gradient of the total pressure, x-component | Domain 4 | + operation |
| acpr.gradpy | d(acpr.p_t,y) | N/m³ | Gradient of the total pressure, y-component | Domain 4 | + operation |
| acpr.gradpz | -acpr.ikz*acpr.p_t | N/m³ | Gradient of the total pressure, z-component | Domain 4 | + operation |
| acpr.gradtestpx | test(px) | N/m³ | Help variable for equations, x-component | Domain 4 | + operation |
| acpr.gradtestpy | test(py) | N/m³ | Help variable for equations, y-component | Domain 4 | + operation |
| acpr.gradtestpz | acpr.ikz*test(p) | N/m³ | Help variable for equations, z-component | Domain 4 | + operation |
| acpr.hessianpxx | d(d(acpr.p_t,x),x) | N/(m·m³) | Hessian of the total pressure, xx-component | Domain 4 | + operation |
| acpr.hessianpyx | d(d(acpr.p_t,y),x) | N/(m·m³) | Hessian of the total pressure, yx-component | Domain 4 | + operation |
| acpr.hessianpzx | -acpr.ikz*d(acpr.p_t,x) | N/(m·m³) | Hessian of the total pressure, zx-component | Domain 4 | + operation |
| acpr.hessianpxy | d(d(acpr.p_t,x),y) | N/(m·m³) | Hessian of the total pressure, xy-component | Domain 4 | + operation |
| acpr.hessianpyy | d(d(acpr.p_t,y),y) | N/(m·m³) | Hessian of the total pressure, yy-component | Domain 4 | + operation |
| acpr.hessianpzy | -acpr.ikz*d(acpr.p_t,y) | N/(m·m³) | Hessian of the total pressure, zy-component | Domain 4 | + operation |
| acpr.hessianpxz | -d(acpr.p_t,x)*acpr.ikz | N/(m·m³) | Hessian of the total pressure, xz-component | Domain 4 | + operation |
| acpr.hessianpyz | -d(acpr.p_t,y)*acpr.ikz | N/(m·m³) | Hessian of the total pressure, yz-component | Domain 4 | + operation |
| acpr.hessianpzz | acpr.ikz^2*acpr.p_t | N/(m·m³) | Hessian of the total pressure, zz-component | Domain 4 | + operation |
| acpr.hessiantestpxx | test(pxx) | N/(m·m³) | Help variable for equations, xx-component | Domain 4 | + operation |
| acpr.hessiantestpyx | test(pyx) | N/(m·m³) | Help variable for equations, yx-component | Domain 4 | + operation |
| acpr.hessiantestpzx | acpr.ikz*test(px) | N/(m·m³) | Help variable for equations, zx-component | Domain 4 | + operation |
| acpr.hessiantestpxy | test(pxy) | N/(m·m³) | Help variable for equations, xy-component | Domain 4 | + operation |
| acpr.hessiantestpyy | test(pyy) | N/(m·m³) | Help variable for equations, yy-component | Domain 4 | + operation |
| acpr.hessiantestpzy | acpr.ikz*test(py) | N/(m·m³) | Help variable for equations, zy-component | Domain 4 | + operation |
| acpr.hessiantestpxz | test(px)*acpr.ikz | N/(m·m³) | Help variable for equations, xz-component | Domain 4 | + operation |
| acpr.hessiantestpyz | test(py)*acpr.ikz | N/(m·m³) | Help variable for equations, yz-component | Domain 4 | + operation |
| acpr.hessiantestpzz | acpr.ikz^2*test(p) | N/(m·m³) | Help variable for equations, zz-component | Domain 4 | + operation |
| acpr.rho | material.rho | kg/m³ | Density | Domain 4 | Meta |
| acpr.c | material.c | m/s | Speed of sound | Domain 4 | Meta |
| acpr.muB | material.muB | Pa·s | Bulk viscosity | Domain 4 | Meta |
| acpr.nacc | 0 | m/s² | Inward acceleration (source) | Boundaries 12, 16–17, 20, 29–32 | + operation |
| acpr.Q | 0 | 1/s² | Total monopole domain source | Domain 4 | + operation |
| acpr.FAcoPerAreax | acpr.p_t*acpr.nx | N/m² | Acoustic load per unit area, x-component | Boundaries 12, 16–17, 20, 29–32 |  |
| acpr.FAcoPerAreay | acpr.p_t*acpr.ny | N/m² | Acoustic load per unit area, y-component | Boundaries 12, 16–17, 20, 29–32 |  |
| acpr.FAcoPerAreaz | acpr.p_t*acpr.nz | N/m² | Acoustic load per unit area, z-component | Boundaries 12, 16–17, 20, 29–32 |  |
| acpr.p_t | p+acpr.p_b | Pa | Total acoustic pressure | Domain 4 |  |
| acpr.c_c | acpr.c*(1+acpr.iomega*acpr.delta_diff/acpr.c^2)^0.5 | m/s | Complex speed of sound | Domain 4 |  |
| acpr.rho_c | acpr.rho*acpr.c^2/acpr.c_c^2 | kg/m³ | Complex density | Domain 4 |  |
| acpr.Z | acpr.rho_c*acpr.c_c | Pa·s/m | Specific characteristic acoustic impedance | Domain 4 |  |
| acpr.k | acpr.iomega/(i*acpr.c_c) | rad/m | Wave number | Domain 4 |  |
| acpr.ik | acpr.iomega/acpr.c_c | rad/m | Phase-shifted wave number | Domain 4 |  |
| acpr.K_eff | acpr.rho_c*acpr.c_c^2 | Pa | Effective bulk modulus | Domain 4 |  |
| acpr.delta_diff | acpr.fpam1.b/acpr.rho | m²/s | Sound diffusivity | Domain 4 |  |
| acpr.fpam1.b | acpr.fpam1.b_visc | kg/(m·s) | Intermediate variable | Domain 4 | + operation |
| acpr.fpam1.b_visc | 4*acpr.mu/3+acpr.muB | Pa·s | Intermediate variable | Domain 4 |  |
| acpr.dmudp | d(acpr.mu,acpr.fpam1.minput_pressure) | s | Pressure dependency of the viscosity | Domain 4 |  |
| acpr.dmudT | d(acpr.mu,acpr.fpam1.minput_temperature) | kg/(m·s·K) | Temperature dependency of the viscosity | Domain 4 |  |
| acpr.keq_sq | -acpr.ik^2+acpr.ikz^2 | 1/m² | Squared wave number for equations | Domain 4 | + operation |
| acpr.keq | sqrt(acpr.keq_sq) | rad/m | Wave number for equations | Domain 4 |  |
| acpr.kip | sqrt(acpr.keq_sq) | rad/m | In-plane wave number | Domain 4 |  |
| acpr.vipx | acpr.v_tx | m/s | In-plane total acoustic velocity, x-component | Domain 4 |  |
| acpr.vipy | acpr.v_ty | m/s | In-plane total acoustic velocity, y-component | Domain 4 |  |
| acpr.vipz | 0 | m/s | In-plane total acoustic velocity, z-component | Domain 4 |  |
| acpr.vopx | 0 | m/s | Out-of-plane total acoustic velocity, x-component | Domain 4 |  |
| acpr.vopy | 0 | m/s | Out-of-plane total acoustic velocity, y-component | Domain 4 |  |
| acpr.vopz | acpr.v_tz | m/s | Out-of-plane total acoustic velocity, z-component | Domain 4 |  |
| acpr.aipx | acpr.a_tx | m/s² | In-plane total acoustic acceleration, x-component | Domain 4 |  |
| acpr.aipy | acpr.a_ty | m/s² | In-plane total acoustic acceleration, y-component | Domain 4 |  |
| acpr.aipz | 0 | m/s² | In-plane total acoustic acceleration, z-component | Domain 4 |  |
| acpr.aopx | 0 | m/s² | Out-of-plane total acoustic acceleration, x-component | Domain 4 |  |
| acpr.aopy | 0 | m/s² | Out-of-plane total acoustic acceleration, y-component | Domain 4 |  |
| acpr.aopz | acpr.a_tz | m/s² | Out-of-plane total acoustic acceleration, z-component | Domain 4 |  |
| acpr.Iipx | acpr.Ix | W/m² | In-plane intensity, x-component | Domain 4 |  |
| acpr.Iipy | acpr.Iy | W/m² | In-plane intensity, y-component | Domain 4 |  |
| acpr.Iipz | 0 | W/m² | In-plane intensity, z-component | Domain 4 |  |
| acpr.Iopx | 0 | W/m² | Out-of-plane intensity, x-component | Domain 4 |  |
| acpr.Iopy | 0 | W/m² | Out-of-plane intensity, y-component | Domain 4 |  |
| acpr.Iopz | acpr.Iz | W/m² | Out-of-plane intensity, z-component | Domain 4 |  |
| acpr.Iip_mag | sqrt(acpr.Iipx^2+acpr.Iipy^2+acpr.Iipz^2) | W/m² | In-plane intensity magnitude | Domain 4 |  |
| acpr.Iop_mag | sqrt(acpr.Iopx^2+acpr.Iopy^2+acpr.Iopz^2) | W/m² | Out-of-plane intensity magnitude | Domain 4 |  |
| acpr.vip_inst | sqrt(real(acpr.vipx)^2+real(acpr.vipy)^2+real(acpr.vipz)^2) | m/s | Instantaneous in-plane total acoustic velocity | Domain 4 |  |
| acpr.vip_rms | sqrt(0.5*(realdot(acpr.vipx,acpr.vipx)+realdot(acpr.vipy,acpr.vipy)+realdot(acpr.vipz,acpr.vipz))) | m/s | In-plane total acoustic velocity, RMS | Domain 4 |  |
| acpr.vop_inst | sqrt(real(acpr.vopx)^2+real(acpr.vopy)^2+real(acpr.vopz)^2) | m/s | Instantaneous out-of-plane total acoustic velocity | Domain 4 |  |
| acpr.vop_rms | sqrt(0.5*(realdot(acpr.vopx,acpr.vopx)+realdot(acpr.vopy,acpr.vopy)+realdot(acpr.vopz,acpr.vopz))) | m/s | Out-of-plane total acoustic velocity, RMS | Domain 4 |  |
| acpr.aip_inst | sqrt(real(acpr.aipx)^2+real(acpr.aipy)^2+real(acpr.aipz)^2) | m/s² | Instantaneous in-plane total acoustic acceleration | Domain 4 |  |
| acpr.aip_rms | sqrt(0.5*(realdot(acpr.aipx,acpr.aipx)+realdot(acpr.aipy,acpr.aipy)+realdot(acpr.aipz,acpr.aipz))) | m/s² | In-plane total acoustic acceleration, RMS | Domain 4 |  |
| acpr.aop_inst | sqrt(real(acpr.aopx)^2+real(acpr.aopy)^2+real(acpr.aopz)^2) | m/s² | Instantaneous out-of-plane total acoustic acceleration | Domain 4 |  |
| acpr.aop_rms | sqrt(0.5*(realdot(acpr.aopx,acpr.aopx)+realdot(acpr.aopy,acpr.aopy)+realdot(acpr.aopz,acpr.aopz))) | m/s² | Out-of-plane total acoustic acceleration, RMS | Domain 4 |  |
| acpr.a_inst | sqrt(real(acpr.ax)^2+real(acpr.ay)^2+real(acpr.az)^2) | m/s² | Instantaneous total acoustic acceleration | Domain 4 |  |
| acpr.a_rms | sqrt(0.5*(realdot(acpr.ax,acpr.ax)+realdot(acpr.ay,acpr.ay)+realdot(acpr.az,acpr.az))) | m/s² | Total acoustic acceleration, RMS | Domain 4 |  |
| acpr.v_inst | sqrt(real(acpr.vx)^2+real(acpr.vy)^2+real(acpr.vz)^2) | m/s | Instantaneous total acoustic velocity | Domain 4 |  |
| acpr.v_rms | sqrt(0.5*(realdot(acpr.vx,acpr.vx)+realdot(acpr.vy,acpr.vy)+realdot(acpr.vz,acpr.vz))) | m/s | Total acoustic velocity, RMS | Domain 4 |  |
| acpr.I_mag | sqrt(acpr.Ix^2+acpr.Iy^2+acpr.Iz^2) | W/m² | Intensity magnitude | Domain 4 |  |
| acpr.absp_t | sqrt(realdot(acpr.p_t,acpr.p_t)) | Pa | Absolute total acoustic pressure | Domain 4 |  |
| acpr.absp_s | sqrt(realdot(acpr.p_s,acpr.p_s)) | Pa | Absolute scattered acoustic pressure | Domain 4 |  |
| acpr.absp_b | sqrt(realdot(acpr.p_b,acpr.p_b)) | Pa | Absolute background acoustic pressure | Domain 4 |  |
| acpr.Lp_t | 20*log10(acpr.p_t_rms/acpr.pref_SPL) | dB | Total sound pressure level | Domain 4 |  |
| acpr.p_t_rms | sqrt(0.5*realdot(acpr.p_t,acpr.p_t)) | Pa | Total acoustic pressure, RMS | Domain 4 |  |
| acpr.p_s_rms | sqrt(0.5*realdot(acpr.p_s,acpr.p_s)) | Pa | Scattered acoustic pressure, RMS | Domain 4 |  |
| acpr.p_b_rms | sqrt(0.5*realdot(acpr.p_b,acpr.p_b)) | Pa | Background acoustic pressure, RMS | Domain 4 |  |
| acpr.diss_visc | 0.5*acpr.omega^2*acpr.p_t*conj(acpr.p_t)*acpr.fpam1.b_visc/(acpr.rho*acpr.c^2)^2 | W/m³ | Viscous power dissipation density | Domain 4 |  |
| acpr.diss_therm | 0 | W/m³ | Thermal power dissipation density | Domain 4 |  |
| acpr.diss_tot | acpr.diss_visc+acpr.diss_therm | W/m³ | Total thermoviscous power dissipation density | Domain 4 |  |
| acpr.Q_pw | -2*imag(acpr.k)*acpr.I_mag | W/m³ | Plane wave total power dissipation density | Domain 4 |  |
| acpr.rho_eff_invxx | 1/acpr.rho_c | m³/kg | Inverse of the effective density, xx-component | Domain 4 |  |
| acpr.rho_eff_invyx | 0 | m³/kg | Inverse of the effective density, yx-component | Domain 4 |  |
| acpr.rho_eff_invzx | 0 | m³/kg | Inverse of the effective density, zx-component | Domain 4 |  |
| acpr.rho_eff_invxy | 0 | m³/kg | Inverse of the effective density, xy-component | Domain 4 |  |
| acpr.rho_eff_invyy | 1/acpr.rho_c | m³/kg | Inverse of the effective density, yy-component | Domain 4 |  |
| acpr.rho_eff_invzy | 0 | m³/kg | Inverse of the effective density, zy-component | Domain 4 |  |
| acpr.rho_eff_invxz | 0 | m³/kg | Inverse of the effective density, xz-component | Domain 4 |  |
| acpr.rho_eff_invyz | 0 | m³/kg | Inverse of the effective density, yz-component | Domain 4 |  |
| acpr.rho_eff_invzz | 1/acpr.rho_c | m³/kg | Inverse of the effective density, zz-component | Domain 4 |  |
| acpr.rho_effxx | acpr.rho_c | kg/m³ | Effective density, xx-component | Domain 4 |  |
| acpr.rho_effyx | 0 | kg/m³ | Effective density, yx-component | Domain 4 |  |
| acpr.rho_effzx | 0 | kg/m³ | Effective density, zx-component | Domain 4 |  |
| acpr.rho_effxy | 0 | kg/m³ | Effective density, xy-component | Domain 4 |  |
| acpr.rho_effyy | acpr.rho_c | kg/m³ | Effective density, yy-component | Domain 4 |  |
| acpr.rho_effzy | 0 | kg/m³ | Effective density, zy-component | Domain 4 |  |
| acpr.rho_effxz | 0 | kg/m³ | Effective density, xz-component | Domain 4 |  |
| acpr.rho_effyz | 0 | kg/m³ | Effective density, yz-component | Domain 4 |  |
| acpr.rho_effzz | acpr.rho_c | kg/m³ | Effective density, zz-component | Domain 4 |  |
| pxt | d(px,TIME)-pxx*d(x,TIME)-pxy*d(y,TIME) | W/m⁴ | Gradient of p, x component, first time derivative | Domain 4 |  |
| pyt | d(py,TIME)-pyx*d(x,TIME)-pyy*d(y,TIME) | W/m⁴ | Gradient of p, y component, first time derivative | Domain 4 |  |
| pxtt | d(d(px,TIME)-pxx*d(x,TIME)-pxy*d(y,TIME),TIME)-d(d(px,TIME)-pxx*d(x,TIME)-pxy*d(y,TIME),x)*d(x,TIME)-d(d(px,TIME)-pxx*d(x,TIME)-pxy*d(y,TIME),y)*d(y,TIME) | kg/(m²·s⁴) | Gradient of p, x component, second time derivative | Domain 4 |  |
| pytt | d(d(py,TIME)-pyx*d(x,TIME)-pyy*d(y,TIME),TIME)-d(d(py,TIME)-pyx*d(x,TIME)-pyy*d(y,TIME),x)*d(x,TIME)-d(d(py,TIME)-pyx*d(x,TIME)-pyy*d(y,TIME),y)*d(y,TIME) | kg/(m²·s⁴) | Gradient of p, y component, second time derivative | Domain 4 |  |
| pt | d(p,TIME)-px*d(x,TIME)-py*d(y,TIME) | Pa/s | Acoustic pressure, first time derivative | Domain 4 |  |
| ptt | d(d(p,TIME)-px*d(x,TIME)-py*d(y,TIME),TIME)-d(d(p,TIME)-px*d(x,TIME)-py*d(y,TIME),x)*d(x,TIME)-d(d(p,TIME)-px*d(x,TIME)-py*d(y,TIME),y)*d(y,TIME) | Pa/s² | Acoustic pressure, second time derivative | Domain 4 |  |

#### Shape functions

| **Name** | **Shape function** | **Unit** | **Description** | **Shape frame** | **Selection** |
| --- | --- | --- | --- | --- | --- |
| p | Lagrange (Quadratic) | Pa | Acoustic pressure | Spatial | Domain 4 |

#### Weak Expressions

| **Weak expression** | **Integration order** | **Integration frame** | **Selection** |
| --- | --- | --- | --- |
| (-acpr.gradpx*acpr.gradtestpx-acpr.gradpy*acpr.gradtestpy-acpr.gradpz*acpr.gradtestpz-acpr.p_t*test(p)*acpr.ik^2)*acpr.delta/acpr.rho_c | 4 | Spatial | Domain 4 |
| acpr.delta*acpr.Q*test(p) | 4 | Spatial | Domain 4 |
| acpr.delta*(acpr.q_totx*acpr.gradtestpx+acpr.q_toty*acpr.gradtestpy+acpr.q_totz*acpr.gradtestpz)/acpr.rho_c | 4 | Spatial | Domain 4 |

### Sound Hard Boundary (Wall) 1


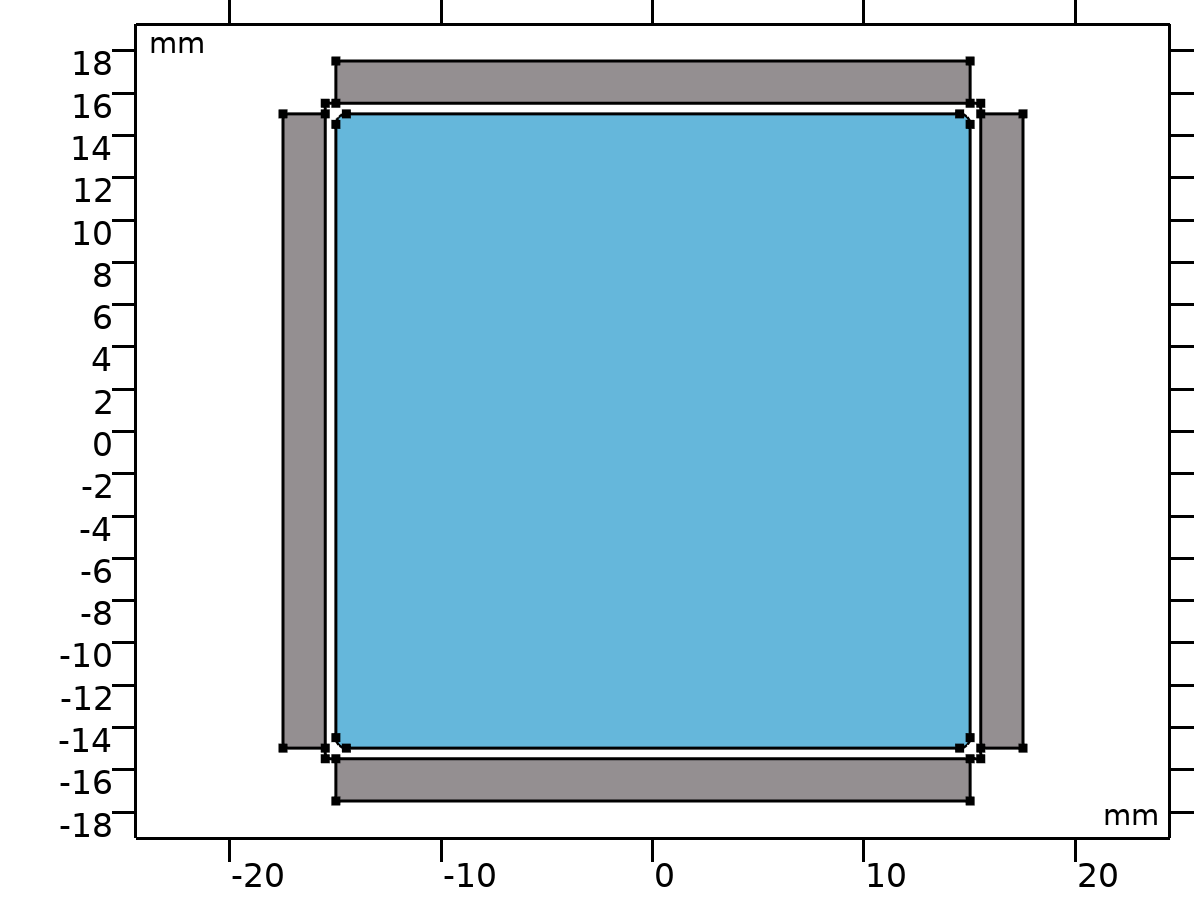


Sound Hard Boundary (Wall) 1

Selection

| Geometric entity level | Boundary |
| --- | --- |
| Selection | Geometry geom1: Dimension 1: All boundaries |

Equations


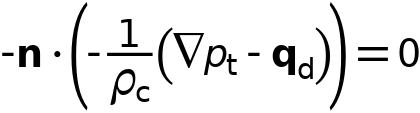


Used products

| COMSOL Multiphysics |
| --- |

Properties from material

| **Property** | **Material** | **Property group** |
| --- | --- | --- |
| Density | Polystyrene (PS) [solid] | Basic |

### Initial Values 1


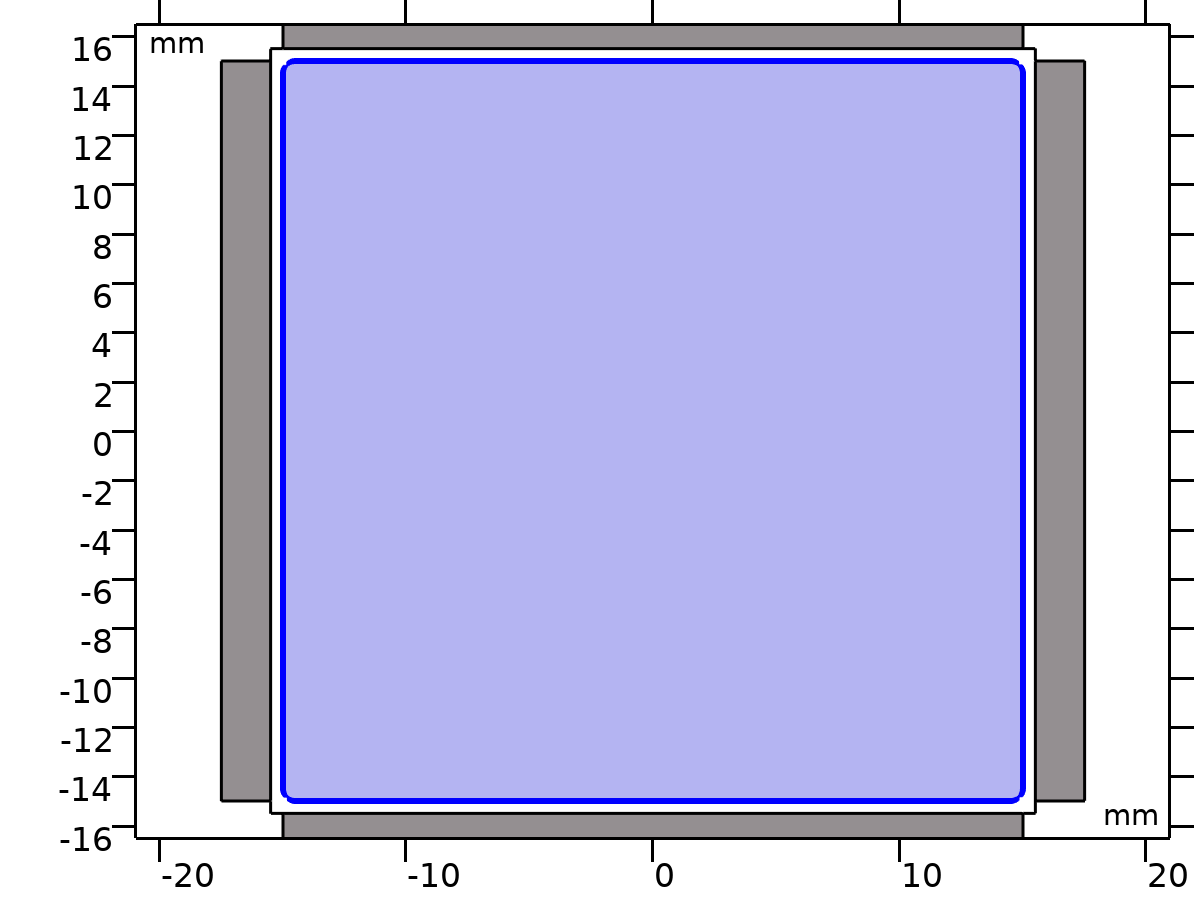


Initial Values 1

Selection

| Geometric entity level | Domain |
| --- | --- |
| Selection | Geometry geom1: Dimension 2: All domains |

Settings

| **Description** | **Value** | **Unit** |
| --- | --- | --- |
| Acoustic pressure | 0 | Pa |

Used products

| COMSOL Multiphysics |
| --- |

Properties from material

| **Property** | **Material** | **Property group** |
| --- | --- | --- |
| Density | Polystyrene (PS) [solid] | Basic |

## Solid Mechanics

Used products

| Acoustics Module |
| --- |
| COMSOL Multiphysics |


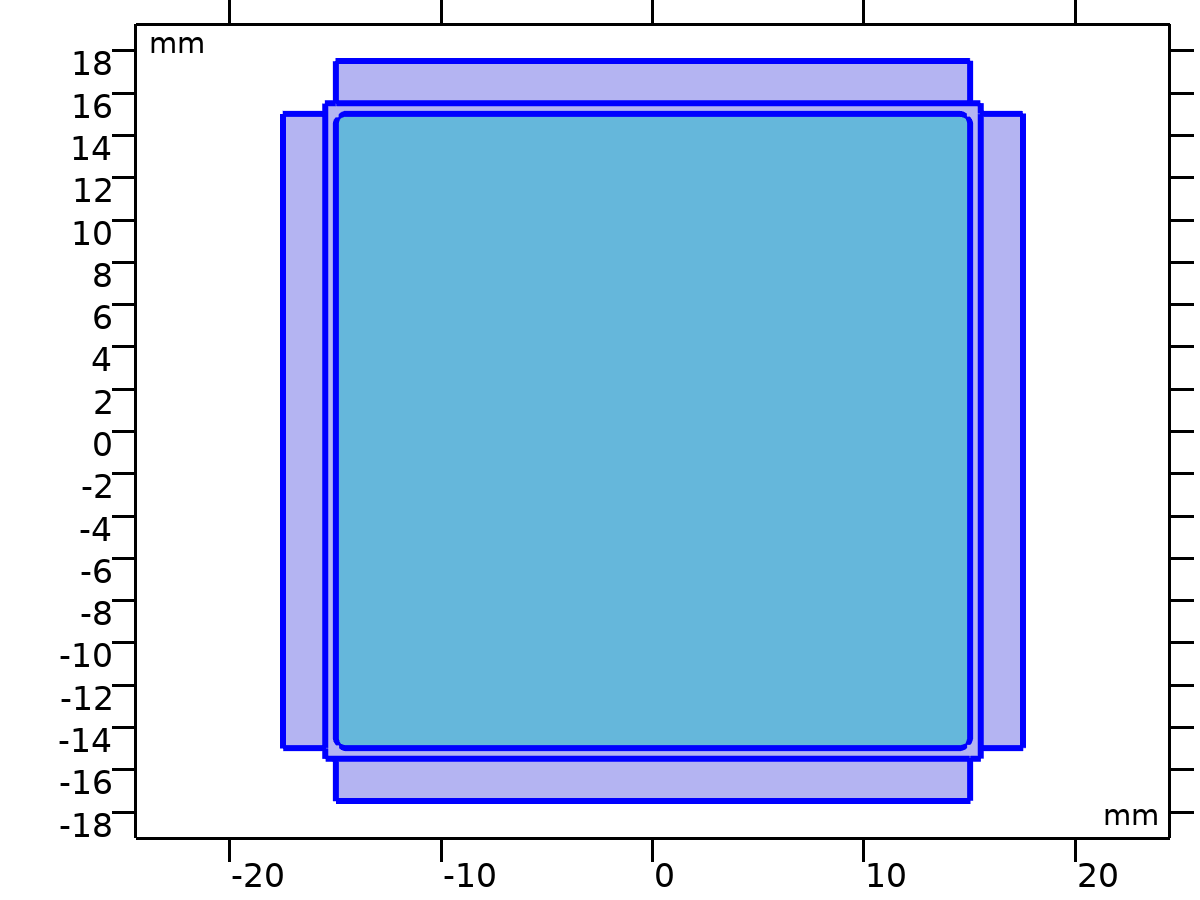


Solid Mechanics

Selection

| Geometric entity level | Domain |
| --- | --- |
| Name | Solid domains |
| Selection | Named sel5: Geometry geom1: Dimension 2: Domains 1–3, 5–6 |

Equations


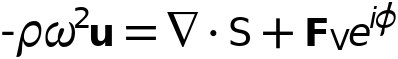


### Interface Settings

#### Physics Symbols

Settings

| **Description** | **Value** |
| --- | --- |
| Enable physics symbols | Off |

#### Discretization

Settings

| **Description** | **Value** |
| --- | --- |
| Displacement field | Quadratic serendipity |

#### 2D Approximation

Settings

| **Description** | **Value** |
| --- | --- |
| 2D approximation | Plane strain |
| Out-of-plane mode extension (time-harmonic) | Off |

Settings

| **Description** | **Value** | **Unit** |
| --- | --- | --- |
| Thickness | 0.01 | m |

#### Structural Transient Behavior

Settings

| **Description** | **Value** |
| --- | --- |
| Structural transient behavior | Include inertial terms |

#### Transient Solver Settings

Settings

| **Description** | **Value** |
| --- | --- |
| Description | Changes made to these settings only take effect when the default solver is generated. |
| Maximum frequency to resolve | Off |

#### Typical Wave Speed for Perfectly Matched Layers

Settings

| **Description** | **Value** | **Unit** |
| --- | --- | --- |
| Typical wave speed for perfectly matched layers | solid.cp | m/s |

#### Advanced Settings

Settings

| **Description** | **Value** |
| --- | --- |
| Rigid materials | On |
| Rigid connectors | On |
| Attachments | On |

### Variables

| **Name** | **Expression** | **Unit** | **Description** | **Selection** | **Details** |
| --- | --- | --- | --- | --- | --- |
| solid.X | X | m | Material coordinates, X-component | Domains 1–3, 5–6 |  |
| solid.Y | Y | m | Material coordinates, Y-component | Domains 1–3, 5–6 |  |
| solid.Z | 0 | m | Material coordinates, Z-component | Domains 1–3, 5–6 |  |
| solid.X | X | m | Material coordinates, X-component | Boundaries 1–32 |  |
| solid.Y | Y | m | Material coordinates, Y-component | Boundaries 1–32 |  |
| solid.Z | 0 | m | Material coordinates, Z-component | Boundaries 1–32 |  |
| solid.x | x | m | Spatial coordinates, x-component | Domains 1–3, 5–6 |  |
| solid.y | y | m | Spatial coordinates, y-component | Domains 1–3, 5–6 |  |
| solid.z | 0 | m | Spatial coordinates, z-component | Domains 1–3, 5–6 |  |
| solid.x | x | m | Spatial coordinates, x-component | Boundaries 1–32 |  |
| solid.y | y | m | Spatial coordinates, y-component | Boundaries 1–32 |  |
| solid.z | 0 | m | Spatial coordinates, z-component | Boundaries 1–32 |  |
| solid.uf | u | m | Displacement field, X-component | Domains 1–3, 5–6 |  |
| solid.vf | v | m | Displacement field, Y-component | Domains 1–3, 5–6 |  |
| solid.wf | 0 | m | Displacement field, Z-component | Domains 1–3, 5–6 |  |
| solid.uf | u | m | Displacement field, X-component | Boundaries 1–32 |  |
| solid.vf | v | m | Displacement field, Y-component | Boundaries 1–32 |  |
| solid.wf | 0 | m | Displacement field, Z-component | Boundaries 1–32 |  |
| solid.nX | nX | 1 | Normal vector, X-component | Boundaries 6, 11, 14, 24 |  |
| solid.nY | nY | 1 | Normal vector, Y-component | Boundaries 6, 11, 14, 24 |  |
| solid.nZ | 0 | 1 | Normal vector, Z-component | Boundaries 6, 11, 14, 24 |  |
| solid.nX | unX | 1 | Normal vector, X-component | Boundaries 12, 16–17, 20, 29–32 |  |
| solid.nY | unY | 1 | Normal vector, Y-component | Boundaries 12, 16–17, 20, 29–32 |  |
| solid.nZ | 0 | 1 | Normal vector, Z-component | Boundaries 12, 16–17, 20, 29–32 |  |
| solid.nX | dnX | 1 | Normal vector, X-component | Boundaries 1–5, 7–10, 13, 15, 18–19, 21–23, 25–28 |  |
| solid.nY | dnY | 1 | Normal vector, Y-component | Boundaries 1–5, 7–10, 13, 15, 18–19, 21–23, 25–28 |  |
| solid.nZ | 0 | 1 | Normal vector, Z-component | Boundaries 1–5, 7–10, 13, 15, 18–19, 21–23, 25–28 |  |
| solid.nx | nx | 1 | Normal vector, x-component | Boundaries 6, 11, 14, 24 |  |
| solid.ny | ny | 1 | Normal vector, y-component | Boundaries 6, 11, 14, 24 |  |
| solid.nz | 0 | 1 | Normal vector, z-component | Boundaries 6, 11, 14, 24 |  |
| solid.nx | unx | 1 | Normal vector, x-component | Boundaries 12, 16–17, 20, 29–32 |  |
| solid.ny | uny | 1 | Normal vector, y-component | Boundaries 12, 16–17, 20, 29–32 |  |
| solid.nz | 0 | 1 | Normal vector, z-component | Boundaries 12, 16–17, 20, 29–32 |  |
| solid.nx | dnx | 1 | Normal vector, x-component | Boundaries 1–5, 7–10, 13, 15, 18–19, 21–23, 25–28 |  |
| solid.ny | dny | 1 | Normal vector, y-component | Boundaries 1–5, 7–10, 13, 15, 18–19, 21–23, 25–28 |  |
| solid.nz | 0 | 1 | Normal vector, z-component | Boundaries 1–5, 7–10, 13, 15, 18–19, 21–23, 25–28 |  |
| solid.nXmesh | nXmesh | 1 | Normal vector (mesh), X-component | Boundaries 6, 11, 14, 24 |  |
| solid.nYmesh | nYmesh | 1 | Normal vector (mesh), Y-component | Boundaries 6, 11, 14, 24 |  |
| solid.nZmesh | 0 | 1 | Normal vector (mesh), Z-component | Boundaries 6, 11, 14, 24 |  |
| solid.nXmesh | unXmesh | 1 | Normal vector (mesh), X-component | Boundaries 12, 16–17, 20, 29–32 |  |
| solid.nYmesh | unYmesh | 1 | Normal vector (mesh), Y-component | Boundaries 12, 16–17, 20, 29–32 |  |
| solid.nZmesh | 0 | 1 | Normal vector (mesh), Z-component | Boundaries 12, 16–17, 20, 29–32 |  |
| solid.nXmesh | dnXmesh | 1 | Normal vector (mesh), X-component | Boundaries 1–5, 7–10, 13, 15, 18–19, 21–23, 25–28 |  |
| solid.nYmesh | dnYmesh | 1 | Normal vector (mesh), Y-component | Boundaries 1–5, 7–10, 13, 15, 18–19, 21–23, 25–28 |  |
| solid.nZmesh | 0 | 1 | Normal vector (mesh), Z-component | Boundaries 1–5, 7–10, 13, 15, 18–19, 21–23, 25–28 |  |
| solid.nxmesh | nxmesh | 1 | Normal vector (mesh), x-component | Boundaries 6, 11, 14, 24 |  |
| solid.nymesh | nymesh | 1 | Normal vector (mesh), y-component | Boundaries 6, 11, 14, 24 |  |
| solid.nzmesh | 0 | 1 | Normal vector (mesh), z-component | Boundaries 6, 11, 14, 24 |  |
| solid.nxmesh | unxmesh | 1 | Normal vector (mesh), x-component | Boundaries 12, 16–17, 20, 29–32 |  |
| solid.nymesh | unymesh | 1 | Normal vector (mesh), y-component | Boundaries 12, 16–17, 20, 29–32 |  |
| solid.nzmesh | 0 | 1 | Normal vector (mesh), z-component | Boundaries 12, 16–17, 20, 29–32 |  |
| solid.nxmesh | dnxmesh | 1 | Normal vector (mesh), x-component | Boundaries 1–5, 7–10, 13, 15, 18–19, 21–23, 25–28 |  |
| solid.nymesh | dnymesh | 1 | Normal vector (mesh), y-component | Boundaries 1–5, 7–10, 13, 15, 18–19, 21–23, 25–28 |  |
| solid.nzmesh | 0 | 1 | Normal vector (mesh), z-component | Boundaries 1–5, 7–10, 13, 15, 18–19, 21–23, 25–28 |  |
| solid.Q_freq | 2*solid.omega*solid.Wh_tot/if(solid.Qh_tot==0,eps,solid.Qh_tot) | 1 | Quality factor for frequency | Global |  |
| solid.d | 10[mm] | m | Thickness | Domains 1–3, 5–6 |  |
| solid.cref | solid.cp | m/s | Typical wave speed for perfectly matched layers | Domains 1–3, 5–6 |  |
| solid.isGeomNon | 0 | 1 | Geometric nonlinearity variable | Global |  |
| solid.Ws_tot | 0 | J | Total elastic strain energy | Global | + operation |
| solid.Wl_tot | 0 | J | Total potential energy of applied loads | Global | + operation |
| solid.W_tot | solid.Ws_tot+solid.Wl_tot | J | Total potential energy | Global | + operation |
| solid.freq | freq | Hz | Frequency | Global |  |
| solid.omega | 2*pi*solid.freq | rad/s | Angular frequency | Global |  |
| solid.activation_multiplier | 1 | 1 | Activation multiplier | Domains 1–3, 5–6 |  |
| solid.geomsize | 0.04949747468305833 | m | Bounding box | Global |  |
| solid.timestep | 0 | s | Time step | Domains 1–3, 5–6 |  |
| solid.RFtotalx | solid.sumreaction(solid.RFx)+solid.RFfsx+solid.RFfdx | N | Total reaction force, x-component | Global | + operation |
| solid.RFtotaly | solid.sumreaction(solid.RFy)+solid.RFfsy+solid.RFfdy | N | Total reaction force, y-component | Global | + operation |
| solid.RFtotalz | solid.sumreaction(solid.RFz)+solid.RFfsz+solid.RFfdz | N | Total reaction force, z-component | Global | + operation |
| solid.RMtotalx | solid.sumreaction(solid.RMx)+solid.RMmsx+solid.RMmdx | N·m | Total reaction moment, x-component | Global | + operation |
| solid.RMtotaly | solid.sumreaction(solid.RMy)+solid.RMmsy+solid.RMmdy | N·m | Total reaction moment, y-component | Global | + operation |
| solid.RMtotalz | solid.sumreaction(solid.RMz)+solid.RMmsz+solid.RMmdz | N·m | Total reaction moment, z-component | Global | + operation |
| solid.beta_dK | 0 | s | Stiffness damping parameter | Domains 1–3, 5–6 | + operation |
| xt | d(x,TIME) | m/s | Mesh velocity, x-component | Global |  |
| yt | d(y,TIME) | m/s | Mesh velocity, y-component | Global |  |
| zt | 0 | m/s | Mesh velocity, z-component | Global |  |
| solid.iomega | solid.omega*i | rad/s | Complex angular frequency | Global |  |

### Linear Elastic Material 1


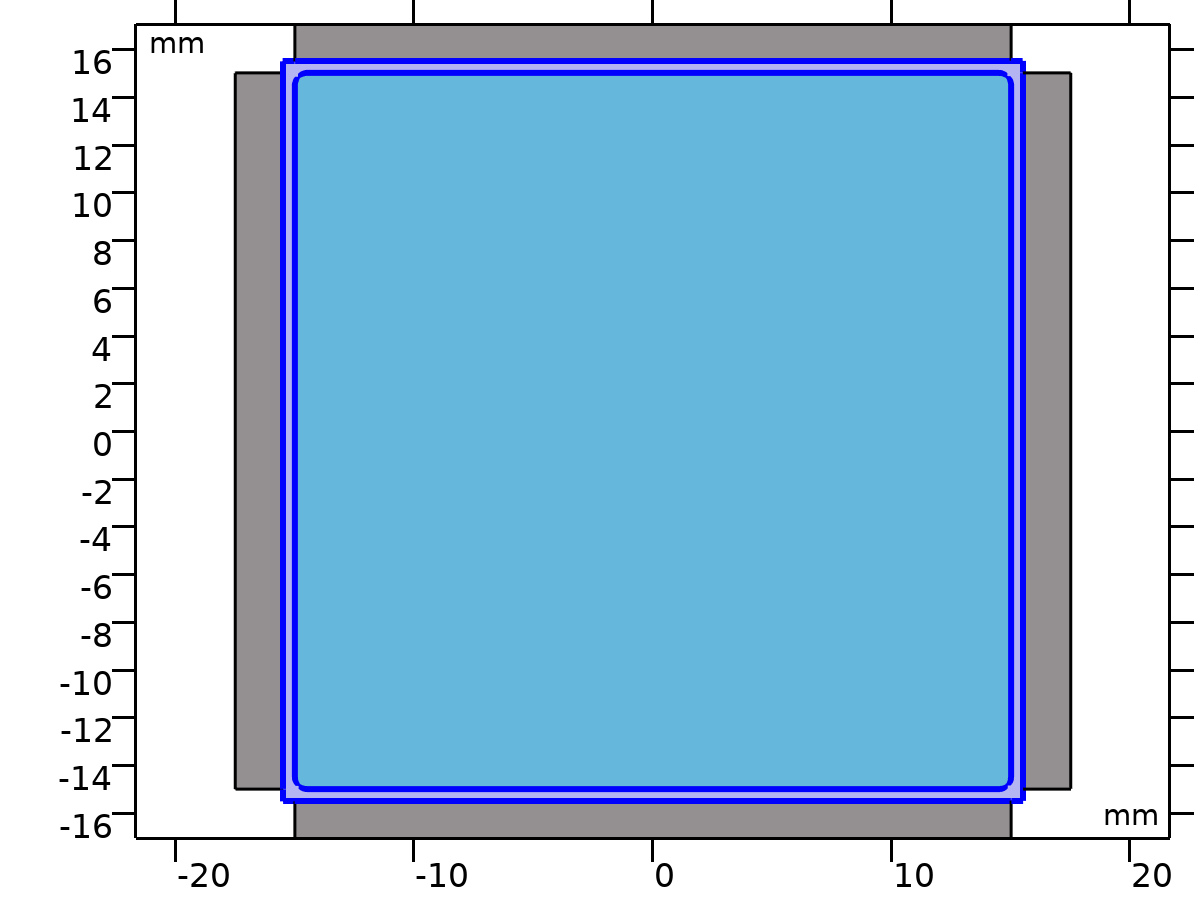


Linear Elastic Material 1

Selection

| Geometric entity level | Domain |
| --- | --- |
| Selection | Geometry geom1: Dimension 2: All domains |

Equations


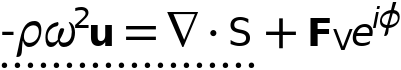


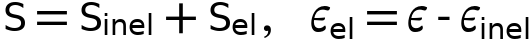


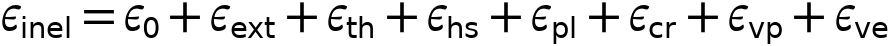


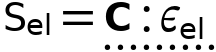


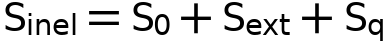


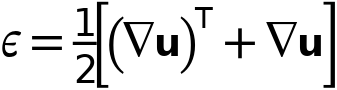


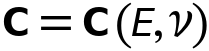


#### Linear Elastic Material

Settings

| **Description** | **Value** |
| --- | --- |
| Material symmetry | Isotropic |
| Specify | Young's modulus and Poisson's ratio |
| Young's modulus | From material |
| Poisson's ratio | From material |
| Density | From material |
| Use mixed formulation | None |

#### Geometric Nonlinearity

Settings

| **Description** | **Value** |
| --- | --- |
| Formulation | From study step |
| Strain decomposition | Automatic |

#### Energy Dissipation

Settings

| **Description** | **Value** |
| --- | --- |
| Calculate dissipated energy | Off |

#### Quadrature Settings

Settings

| **Description** | **Value** |
| --- | --- |
| Reduced integration | Off |

#### Coordinate System Selection

Settings

| **Description** | **Value** |
| --- | --- |
| Coordinate system | Global coordinate system |

#### Model Input

Settings

| **Description** | **Value** |
| --- | --- |
| Volume reference temperature | Common model input |
| Temperature | Common model input |

Used products

| COMSOL Multiphysics |
| --- |

Properties from material

| **Property** | **Material** | **Property group** |
| --- | --- | --- |
| Young's modulus | PMMA - Polymethyl methacrylate | Young's modulus and Poisson's ratio |
| Poisson's ratio | PMMA - Polymethyl methacrylate | Young's modulus and Poisson's ratio |
| Density | PMMA - Polymethyl methacrylate | Basic |
| Density | Polystyrene (PS) [solid] | Basic |

#### Variables

| **Name** | **Expression** | **Unit** | **Description** | **Selection** | **Details** |
| --- | --- | --- | --- | --- | --- |
| solid.disp | sqrteps(real(u)^2+real(v)^2) | m | Displacement magnitude | Domain 2 |  |
| solid.disp_rms | sqrt(0.5*(realdot(u,u)+realdot(v,v))) | m | Displacement, RMS | Domain 2 |  |
| solid.curlUX | 0 | 1 | Curl of displacement, X-component | Domain 2 |  |
| solid.curlUY | 0 | 1 | Curl of displacement, Y-component | Domain 2 |  |
| solid.curlUZ | solid.gradUyX-solid.gradUxY | 1 | Curl of displacement, Z-component | Domain 2 |  |
| solid.gradUxX | uX | 1 | Displacement gradient, xX-component | Domain 2 |  |
| solid.gradUyX | vX | 1 | Displacement gradient, yX-component | Domain 2 |  |
| solid.gradUzX | 0 | 1 | Displacement gradient, zX-component | Domain 2 |  |
| solid.gradUxY | uY | 1 | Displacement gradient, xY-component | Domain 2 |  |
| solid.gradUyY | vY | 1 | Displacement gradient, yY-component | Domain 2 |  |
| solid.gradUzY | 0 | 1 | Displacement gradient, zY-component | Domain 2 |  |
| solid.gradUxZ | 0 | 1 | Displacement gradient, xZ-component | Domain 2 |  |
| solid.gradUyZ | 0 | 1 | Displacement gradient, yZ-component | Domain 2 |  |
| solid.gradUzZ | 0 | 1 | Displacement gradient, zZ-component | Domain 2 |  |
| solid.FdxX | 1+solid.gradUxX | 1 | Deformation gradient, xX-component | Domain 2 |  |
| solid.FdyX | solid.gradUyX | 1 | Deformation gradient, yX-component | Domain 2 |  |
| solid.FdzX | solid.gradUzX | 1 | Deformation gradient, zX-component | Domain 2 |  |
| solid.FdxY | solid.gradUxY | 1 | Deformation gradient, xY-component | Domain 2 |  |
| solid.FdyY | 1+solid.gradUyY | 1 | Deformation gradient, yY-component | Domain 2 |  |
| solid.FdzY | solid.gradUzY | 1 | Deformation gradient, zY-component | Domain 2 |  |
| solid.FdxZ | solid.gradUxZ | 1 | Deformation gradient, xZ-component | Domain 2 |  |
| solid.FdyZ | solid.gradUyZ | 1 | Deformation gradient, yZ-component | Domain 2 |  |
| solid.FdzZ | 1+solid.gradUzZ | 1 | Deformation gradient, zZ-component | Domain 2 |  |
| solid.FdiXx | (solid.FdyY*solid.FdzZ-solid.FdyZ*solid.FdzY)/solid.J | 1 | Deformation gradient inverse, Xx-component | Domain 2 |  |
| solid.FdiYx | (solid.FdyZ*solid.FdzX-solid.FdyX*solid.FdzZ)/solid.J | 1 | Deformation gradient inverse, Yx-component | Domain 2 |  |
| solid.FdiZx | (solid.FdyX*solid.FdzY-solid.FdyY*solid.FdzX)/solid.J | 1 | Deformation gradient inverse, Zx-component | Domain 2 |  |
| solid.FdiXy | (solid.FdxZ*solid.FdzY-solid.FdxY*solid.FdzZ)/solid.J | 1 | Deformation gradient inverse, Xy-component | Domain 2 |  |
| solid.FdiYy | (solid.FdxX*solid.FdzZ-solid.FdxZ*solid.FdzX)/solid.J | 1 | Deformation gradient inverse, Yy-component | Domain 2 |  |
| solid.FdiZy | (solid.FdxY*solid.FdzX-solid.FdxX*solid.FdzY)/solid.J | 1 | Deformation gradient inverse, Zy-component | Domain 2 |  |
| solid.FdiXz | (solid.FdxY*solid.FdyZ-solid.FdxZ*solid.FdyY)/solid.J | 1 | Deformation gradient inverse, Xz-component | Domain 2 |  |
| solid.FdiYz | (solid.FdxZ*solid.FdyX-solid.FdxX*solid.FdyZ)/solid.J | 1 | Deformation gradient inverse, Yz-component | Domain 2 |  |
| solid.FdiZz | (solid.FdxX*solid.FdyY-solid.FdxY*solid.FdyX)/solid.J | 1 | Deformation gradient inverse, Zz-component | Domain 2 |  |
| solid.J | solid.FdxX*solid.FdyY*solid.FdzZ+solid.FdxY*solid.FdyZ*solid.FdzX+solid.FdxZ*solid.FdyX*solid.FdzY-solid.FdxX*solid.FdyZ*solid.FdzY-solid.FdxY*solid.FdyX*solid.FdzZ-solid.FdxZ*solid.FdyY*solid.FdzX | 1 | Volume ratio | Domain 2 |  |
| solid.Fdlx1 | solid.FdxX | 1 | Deformation gradient, local, x1-component | Domain 2 |  |
| solid.Fdly1 | solid.FdyX | 1 | Deformation gradient, local, y1-component | Domain 2 |  |
| solid.Fdlz1 | solid.FdzX | 1 | Deformation gradient, local, z1-component | Domain 2 |  |
| solid.Fdlx2 | solid.FdxY | 1 | Deformation gradient, local, x2-component | Domain 2 |  |
| solid.Fdly2 | solid.FdyY | 1 | Deformation gradient, local, y2-component | Domain 2 |  |
| solid.Fdlz2 | solid.FdzY | 1 | Deformation gradient, local, z2-component | Domain 2 |  |
| solid.Fdlx3 | solid.FdxZ | 1 | Deformation gradient, local, x3-component | Domain 2 |  |
| solid.Fdly3 | solid.FdyZ | 1 | Deformation gradient, local, y3-component | Domain 2 |  |
| solid.Fdlz3 | solid.FdzZ | 1 | Deformation gradient, local, z3-component | Domain 2 |  |
| solid.Fdil1x | (solid.Fdly2*solid.Fdlz3-solid.Fdly3*solid.Fdlz2)/solid.J | 1 | Deformation gradient inverse, local coordinate system, 1x-component | Domain 2 |  |
| solid.Fdil2x | (solid.Fdly3*solid.Fdlz1-solid.Fdly1*solid.Fdlz3)/solid.J | 1 | Deformation gradient inverse, local coordinate system, 2x-component | Domain 2 |  |
| solid.Fdil3x | (solid.Fdly1*solid.Fdlz2-solid.Fdly2*solid.Fdlz1)/solid.J | 1 | Deformation gradient inverse, local coordinate system, 3x-component | Domain 2 |  |
| solid.Fdil1y | (solid.Fdlx3*solid.Fdlz2-solid.Fdlx2*solid.Fdlz3)/solid.J | 1 | Deformation gradient inverse, local coordinate system, 1y-component | Domain 2 |  |
| solid.Fdil2y | (solid.Fdlx1*solid.Fdlz3-solid.Fdlx3*solid.Fdlz1)/solid.J | 1 | Deformation gradient inverse, local coordinate system, 2y-component | Domain 2 |  |
| solid.Fdil3y | (solid.Fdlx2*solid.Fdlz1-solid.Fdlx1*solid.Fdlz2)/solid.J | 1 | Deformation gradient inverse, local coordinate system, 3y-component | Domain 2 |  |
| solid.Fdil1z | (solid.Fdlx2*solid.Fdly3-solid.Fdlx3*solid.Fdly2)/solid.J | 1 | Deformation gradient inverse, local coordinate system, 1z-component | Domain 2 |  |
| solid.Fdil2z | (solid.Fdlx3*solid.Fdly1-solid.Fdlx1*solid.Fdly3)/solid.J | 1 | Deformation gradient inverse, local coordinate system, 2z-component | Domain 2 |  |
| solid.Fdil3z | (solid.Fdlx1*solid.Fdly2-solid.Fdlx2*solid.Fdly1)/solid.J | 1 | Deformation gradient inverse, local coordinate system, 3z-component | Domain 2 |  |
| solid.Ji | 1/(solid.Fiil11*solid.Fiil22*solid.Fiil33+solid.Fiil12*solid.Fiil23*solid.Fiil31+solid.Fiil13*solid.Fiil21*solid.Fiil32-solid.Fiil11*solid.Fiil23*solid.Fiil32-solid.Fiil12*solid.Fiil21*solid.Fiil33-solid.Fiil13*solid.Fiil22*solid.Fiil31) | 1 | Inelastic volume ratio | Domain 2 |  |
| solid.Fil11 | (solid.Fiil22*solid.Fiil33-solid.Fiil23*solid.Fiil32)*solid.Ji | 1 | Inelastic deformation gradient, local coordinate system, 11-component | Domain 2 | Matrix multiplication |
| solid.Fil21 | (solid.Fiil23*solid.Fiil31-solid.Fiil21*solid.Fiil33)*solid.Ji | 1 | Inelastic deformation gradient, local coordinate system, 21-component | Domain 2 | Matrix multiplication |
| solid.Fil31 | (solid.Fiil21*solid.Fiil32-solid.Fiil22*solid.Fiil31)*solid.Ji | 1 | Inelastic deformation gradient, local coordinate system, 31-component | Domain 2 | Matrix multiplication |
| solid.Fil12 | (solid.Fiil13*solid.Fiil32-solid.Fiil12*solid.Fiil33)*solid.Ji | 1 | Inelastic deformation gradient, local coordinate system, 12-component | Domain 2 | Matrix multiplication |
| solid.Fil22 | (solid.Fiil11*solid.Fiil33-solid.Fiil13*solid.Fiil31)*solid.Ji | 1 | Inelastic deformation gradient, local coordinate system, 22-component | Domain 2 | Matrix multiplication |
| solid.Fil32 | (solid.Fiil12*solid.Fiil31-solid.Fiil11*solid.Fiil32)*solid.Ji | 1 | Inelastic deformation gradient, local coordinate system, 32-component | Domain 2 | Matrix multiplication |
| solid.Fil13 | (solid.Fiil12*solid.Fiil23-solid.Fiil13*solid.Fiil22)*solid.Ji | 1 | Inelastic deformation gradient, local coordinate system, 13-component | Domain 2 | Matrix multiplication |
| solid.Fil23 | (solid.Fiil13*solid.Fiil21-solid.Fiil11*solid.Fiil23)*solid.Ji | 1 | Inelastic deformation gradient, local coordinate system, 23-component | Domain 2 | Matrix multiplication |
| solid.Fil33 | (solid.Fiil11*solid.Fiil22-solid.Fiil12*solid.Fiil21)*solid.Ji | 1 | Inelastic deformation gradient, local coordinate system, 33-component | Domain 2 | Matrix multiplication |
| solid.Fiil11 | 1 | 1 | Inelastic deformation gradient inverse, local coordinate system, 11-component | Domain 2 | Matrix multiplication |
| solid.Fiil21 | 0 | 1 | Inelastic deformation gradient inverse, local coordinate system, 21-component | Domain 2 | Matrix multiplication |
| solid.Fiil31 | 0 | 1 | Inelastic deformation gradient inverse, local coordinate system, 31-component | Domain 2 | Matrix multiplication |
| solid.Fiil12 | 0 | 1 | Inelastic deformation gradient inverse, local coordinate system, 12-component | Domain 2 | Matrix multiplication |
| solid.Fiil22 | 1 | 1 | Inelastic deformation gradient inverse, local coordinate system, 22-component | Domain 2 | Matrix multiplication |
| solid.Fiil32 | 0 | 1 | Inelastic deformation gradient inverse, local coordinate system, 32-component | Domain 2 | Matrix multiplication |
| solid.Fiil13 | 0 | 1 | Inelastic deformation gradient inverse, local coordinate system, 13-component | Domain 2 | Matrix multiplication |
| solid.Fiil23 | 0 | 1 | Inelastic deformation gradient inverse, local coordinate system, 23-component | Domain 2 | Matrix multiplication |
| solid.Fiil33 | 1 | 1 | Inelastic deformation gradient inverse, local coordinate system, 33-component | Domain 2 | Matrix multiplication |
| solid.Jel | sqrt(solid.Cel11*solid.Cel22*solid.Cel33+2*solid.Cel12*solid.Cel23*solid.Cel13-solid.Cel11*solid.Cel23^2-solid.Cel12^2*solid.Cel33-solid.Cel22*solid.Cel13^2) | 1 | Elastic volume ratio | Domain 2 |  |
| solid.CXX | solid.FdxX^2+solid.FdyX^2+solid.FdzX^2 | 1 | Cauchy–Green tensor, XX-component | Domain 2 |  |
| solid.CXY | solid.FdxX*solid.FdxY+solid.FdyX*solid.FdyY+solid.FdzX*solid.FdzY | 1 | Cauchy–Green tensor, XY-component | Domain 2 |  |
| solid.CXZ | solid.FdxX*solid.FdxZ+solid.FdyX*solid.FdyZ+solid.FdzX*solid.FdzZ | 1 | Cauchy–Green tensor, XZ-component | Domain 2 |  |
| solid.CYY | solid.FdxY^2+solid.FdyY^2+solid.FdzY^2 | 1 | Cauchy–Green tensor, YY-component | Domain 2 |  |
| solid.CYZ | solid.FdxY*solid.FdxZ+solid.FdyY*solid.FdyZ+solid.FdzY*solid.FdzZ | 1 | Cauchy–Green tensor, YZ-component | Domain 2 |  |
| solid.CZZ | solid.FdxZ^2+solid.FdyZ^2+solid.FdzZ^2 | 1 | Cauchy–Green tensor, ZZ-component | Domain 2 |  |
| solid.Cl11 | 1+2*solid.el11 | 1 | Cauchy–Green tensor, local coordinate system, 11-component | Domain 2 |  |
| solid.Cl12 | 2*solid.el12 | 1 | Cauchy–Green tensor, local coordinate system, 12-component | Domain 2 |  |
| solid.Cl13 | 2*solid.el13 | 1 | Cauchy–Green tensor, local coordinate system, 13-component | Domain 2 |  |
| solid.Cl22 | 1+2*solid.el22 | 1 | Cauchy–Green tensor, local coordinate system, 22-component | Domain 2 |  |
| solid.Cl23 | 2*solid.el23 | 1 | Cauchy–Green tensor, local coordinate system, 23-component | Domain 2 |  |
| solid.Cl33 | 1+2*solid.el33 | 1 | Cauchy–Green tensor, local coordinate system, 33-component | Domain 2 |  |
| solid.Cel11 | solid.FiilCl11*solid.Fiil11+solid.FiilCl12*solid.Fiil21+solid.FiilCl13*solid.Fiil31 | 1 | Elastic Cauchy–Green tensor, local coordinate system, 11-component | Domain 2 |  |
| solid.Cel12 | solid.FiilCl11*solid.Fiil12+solid.FiilCl12*solid.Fiil22+solid.FiilCl13*solid.Fiil32 | 1 | Elastic Cauchy–Green tensor, local coordinate system, 12-component | Domain 2 |  |
| solid.Cel13 | solid.FiilCl11*solid.Fiil13+solid.FiilCl12*solid.Fiil23+solid.FiilCl13*solid.Fiil33 | 1 | Elastic Cauchy–Green tensor, local coordinate system, 13-component | Domain 2 |  |
| solid.Cel22 | solid.FiilCl21*solid.Fiil12+solid.FiilCl22*solid.Fiil22+solid.FiilCl23*solid.Fiil32 | 1 | Elastic Cauchy–Green tensor, local coordinate system, 22-component | Domain 2 |  |
| solid.Cel23 | solid.FiilCl21*solid.Fiil13+solid.FiilCl22*solid.Fiil23+solid.FiilCl23*solid.Fiil33 | 1 | Elastic Cauchy–Green tensor, local coordinate system, 23-component | Domain 2 |  |
| solid.Cel33 | solid.FiilCl31*solid.Fiil13+solid.FiilCl32*solid.Fiil23+solid.FiilCl33*solid.Fiil33 | 1 | Elastic Cauchy–Green tensor, local coordinate system, 33-component | Domain 2 |  |
| solid.Ceil11 | (solid.Cel22*solid.Cel33-solid.Cel23^2)/(solid.Cel11*solid.Cel22*solid.Cel33+2*solid.Cel12*solid.Cel23*solid.Cel13-solid.Cel11*solid.Cel23^2-solid.Cel12^2*solid.Cel33-solid.Cel22*solid.Cel13^2) | 1 | Elastic Cauchy–Green tensor inverse, local coordinate system, 11-component | Domain 2 |  |
| solid.Ceil12 | (solid.Cel23*solid.Cel13-solid.Cel12*solid.Cel33)/(solid.Cel11*solid.Cel22*solid.Cel33+2*solid.Cel12*solid.Cel23*solid.Cel13-solid.Cel11*solid.Cel23^2-solid.Cel12^2*solid.Cel33-solid.Cel22*solid.Cel13^2) | 1 | Elastic Cauchy–Green tensor inverse, local coordinate system, 12-component | Domain 2 |  |
| solid.Ceil13 | (solid.Cel12*solid.Cel23-solid.Cel22*solid.Cel13)/(solid.Cel11*solid.Cel22*solid.Cel33+2*solid.Cel12*solid.Cel23*solid.Cel13-solid.Cel11*solid.Cel23^2-solid.Cel12^2*solid.Cel33-solid.Cel22*solid.Cel13^2) | 1 | Elastic Cauchy–Green tensor inverse, local coordinate system, 13-component | Domain 2 |  |
| solid.Ceil22 | (solid.Cel11*solid.Cel33-solid.Cel13^2)/(solid.Cel11*solid.Cel22*solid.Cel33+2*solid.Cel12*solid.Cel23*solid.Cel13-solid.Cel11*solid.Cel23^2-solid.Cel12^2*solid.Cel33-solid.Cel22*solid.Cel13^2) | 1 | Elastic Cauchy–Green tensor inverse, local coordinate system, 22-component | Domain 2 |  |
| solid.Ceil23 | (solid.Cel12*solid.Cel13-solid.Cel11*solid.Cel23)/(solid.Cel11*solid.Cel22*solid.Cel33+2*solid.Cel12*solid.Cel23*solid.Cel13-solid.Cel11*solid.Cel23^2-solid.Cel12^2*solid.Cel33-solid.Cel22*solid.Cel13^2) | 1 | Elastic Cauchy–Green tensor inverse, local coordinate system, 23-component | Domain 2 |  |
| solid.Ceil33 | (solid.Cel11*solid.Cel22-solid.Cel12^2)/(solid.Cel11*solid.Cel22*solid.Cel33+2*solid.Cel12*solid.Cel23*solid.Cel13-solid.Cel11*solid.Cel23^2-solid.Cel12^2*solid.Cel33-solid.Cel22*solid.Cel13^2) | 1 | Elastic Cauchy–Green tensor inverse, local coordinate system, 33-component | Domain 2 |  |
| solid.eXX | solid.gradUxX | 1 | Strain tensor, XX-component | Domain 2 | + operation |
| solid.eXY | 0.5*(solid.gradUxY+solid.gradUyX) | 1 | Strain tensor, XY-component | Domain 2 | + operation |
| solid.eXZ | 0.5*(solid.gradUxZ+solid.gradUzX) | 1 | Strain tensor, XZ-component | Domain 2 | + operation |
| solid.eYY | solid.gradUyY | 1 | Strain tensor, YY-component | Domain 2 | + operation |
| solid.eYZ | 0.5*(solid.gradUyZ+solid.gradUzY) | 1 | Strain tensor, YZ-component | Domain 2 | + operation |
| solid.eZZ | solid.gradUzZ | 1 | Strain tensor, ZZ-component | Domain 2 | + operation |
| solid.el11 | solid.eXX | 1 | Strain tensor, local coordinate system, 11-component | Domain 2 | + operation |
| solid.el12 | solid.eXY | 1 | Strain tensor, local coordinate system, 12-component | Domain 2 | + operation |
| solid.el13 | solid.eXZ | 1 | Strain tensor, local coordinate system, 13-component | Domain 2 | + operation |
| solid.el22 | solid.eYY | 1 | Strain tensor, local coordinate system, 22-component | Domain 2 | + operation |
| solid.el23 | solid.eYZ | 1 | Strain tensor, local coordinate system, 23-component | Domain 2 | + operation |
| solid.el33 | solid.eZZ | 1 | Strain tensor, local coordinate system, 33-component | Domain 2 | + operation |
| solid.evol | solid.eXX+solid.eYY+solid.eZZ | 1 | Volumetric strain | Domain 2 |  |
| solid.eldev11 | solid.el11-solid.evol/3 | 1 | Deviatoric strain tensor, local coordinate system, 11-component | Domain 2 |  |
| solid.eldev12 | solid.el12 | 1 | Deviatoric strain tensor, local coordinate system, 12-component | Domain 2 |  |
| solid.eldev13 | solid.el13 | 1 | Deviatoric strain tensor, local coordinate system, 13-component | Domain 2 |  |
| solid.eldev22 | solid.el22-solid.evol/3 | 1 | Deviatoric strain tensor, local coordinate system, 22-component | Domain 2 |  |
| solid.eldev23 | solid.el23 | 1 | Deviatoric strain tensor, local coordinate system, 23-component | Domain 2 |  |
| solid.eldev33 | solid.el33-solid.evol/3 | 1 | Deviatoric strain tensor, local coordinate system, 33-component | Domain 2 |  |
| solid.edeve | sqrt(2*(solid.eldev11^2+2*solid.eldev12^2+2*solid.eldev13^2+solid.eldev22^2+2*solid.eldev23^2+solid.eldev33^2)/3+eps^2) | 1 | Equivalent deviatoric strain | Domain 2 |  |
| solid.eeXX | solid.eel11 | 1 | Elastic strain tensor, XX-component | Domain 2 |  |
| solid.eeXY | solid.eel12 | 1 | Elastic strain tensor, XY-component | Domain 2 |  |
| solid.eeXZ | solid.eel13 | 1 | Elastic strain tensor, XZ-component | Domain 2 |  |
| solid.eeYY | solid.eel22 | 1 | Elastic strain tensor, YY-component | Domain 2 |  |
| solid.eeYZ | solid.eel23 | 1 | Elastic strain tensor, YZ-component | Domain 2 |  |
| solid.eeZZ | solid.eel33 | 1 | Elastic strain tensor, ZZ-component | Domain 2 |  |
| solid.eel11 | solid.el11-solid.eiel11 | 1 | Elastic strain tensor, local coordinate system, 11-component | Domain 2 |  |
| solid.eel12 | solid.el12-solid.eiel12 | 1 | Elastic strain tensor, local coordinate system, 12-component | Domain 2 |  |
| solid.eel13 | solid.el13-solid.eiel13 | 1 | Elastic strain tensor, local coordinate system, 13-component | Domain 2 |  |
| solid.eel22 | solid.el22-solid.eiel22 | 1 | Elastic strain tensor, local coordinate system, 22-component | Domain 2 |  |
| solid.eel23 | solid.el23-solid.eiel23 | 1 | Elastic strain tensor, local coordinate system, 23-component | Domain 2 |  |
| solid.eel33 | solid.el33-solid.eiel33 | 1 | Elastic strain tensor, local coordinate system, 33-component | Domain 2 |  |
| solid.eeldev11 | solid.eel11-(solid.eel11+solid.eel22+solid.eel33)/3 | 1 | Deviatoric elastic strain tensor, local coordinate system, 11-component | Domain 2 |  |
| solid.eeldev12 | solid.eel12 | 1 | Deviatoric elastic strain tensor, local coordinate system, 12-component | Domain 2 |  |
| solid.eeldev13 | solid.eel13 | 1 | Deviatoric elastic strain tensor, local coordinate system, 13-component | Domain 2 |  |
| solid.eeldev22 | solid.eel22-(solid.eel11+solid.eel22+solid.eel33)/3 | 1 | Deviatoric elastic strain tensor, local coordinate system, 22-component | Domain 2 |  |
| solid.eeldev23 | solid.eel23 | 1 | Deviatoric elastic strain tensor, local coordinate system, 23-component | Domain 2 |  |
| solid.eeldev33 | solid.eel33-(solid.eel11+solid.eel22+solid.eel33)/3 | 1 | Deviatoric elastic strain tensor, local coordinate system, 33-component | Domain 2 |  |
| solid.eelvol | solid.eel11+solid.eel22+solid.eel33 | 1 | Volumetric elastic strain | Domain 2 |  |
| solid.eil11 | 0 | 1 | Initial strain tensor, local coordinate system, 11-component | Domain 2 | + operation |
| solid.eil12 | 0 | 1 | Initial strain tensor, local coordinate system, 12-component | Domain 2 | + operation |
| solid.eil13 | 0 | 1 | Initial strain tensor, local coordinate system, 13-component | Domain 2 | + operation |
| solid.eil22 | 0 | 1 | Initial strain tensor, local coordinate system, 22-component | Domain 2 | + operation |
| solid.eil23 | 0 | 1 | Initial strain tensor, local coordinate system, 23-component | Domain 2 | + operation |
| solid.eil33 | 0 | 1 | Initial strain tensor, local coordinate system, 33-component | Domain 2 | + operation |
| solid.eiel11 | 0 | 1 | Inelastic strain tensor, local coordinate system, 11-component | Domain 2 | + operation |
| solid.eiel12 | 0 | 1 | Inelastic strain tensor, local coordinate system, 12-component | Domain 2 | + operation |
| solid.eiel13 | 0 | 1 | Inelastic strain tensor, local coordinate system, 13-component | Domain 2 | + operation |
| solid.eiel22 | 0 | 1 | Inelastic strain tensor, local coordinate system, 22-component | Domain 2 | + operation |
| solid.eiel23 | 0 | 1 | Inelastic strain tensor, local coordinate system, 23-component | Domain 2 | + operation |
| solid.eiel33 | 0 | 1 | Inelastic strain tensor, local coordinate system, 33-component | Domain 2 | + operation |
| solid.II2eel | 0.5*(solid.eeldev11^2+2*solid.eeldev12^2+2*solid.eeldev13^2+solid.eeldev22^2+2*solid.eeldev23^2+solid.eeldev33^2) | 1 | Second invariant of deviatoric elastic strain tensor | Domain 2 |  |
| solid.eeGpXX | solid.gpeval(solid.eeXX) | 1 | Elastic strain tensor, XX-component | Domain 2 |  |
| solid.eeGpXY | solid.gpeval(solid.eeXY) | 1 | Elastic strain tensor, XY-component | Domain 2 |  |
| solid.eeGpXZ | solid.gpeval(solid.eeXZ) | 1 | Elastic strain tensor, XZ-component | Domain 2 |  |
| solid.eeGpYY | solid.gpeval(solid.eeYY) | 1 | Elastic strain tensor, YY-component | Domain 2 |  |
| solid.eeGpYZ | solid.gpeval(solid.eeYZ) | 1 | Elastic strain tensor, YZ-component | Domain 2 |  |
| solid.eeGpZZ | solid.gpeval(solid.eeZZ) | 1 | Elastic strain tensor, ZZ-component | Domain 2 |  |
| solid.eelGp11 | solid.gpeval(solid.eel11) | 1 | Elastic strain tensor, local coordinate system, 11-component | Domain 2 |  |
| solid.eelGp12 | solid.gpeval(solid.eel12) | 1 | Elastic strain tensor, local coordinate system, 12-component | Domain 2 |  |
| solid.eelGp13 | solid.gpeval(solid.eel13) | 1 | Elastic strain tensor, local coordinate system, 13-component | Domain 2 |  |
| solid.eelGp22 | solid.gpeval(solid.eel22) | 1 | Elastic strain tensor, local coordinate system, 22-component | Domain 2 |  |
| solid.eelGp23 | solid.gpeval(solid.eel23) | 1 | Elastic strain tensor, local coordinate system, 23-component | Domain 2 |  |
| solid.eelGp33 | solid.gpeval(solid.eel33) | 1 | Elastic strain tensor, local coordinate system, 33-component | Domain 2 |  |
| solid.eeldevGp11 | solid.gpeval(solid.eeldev11) | 1 | Deviatoric elastic strain tensor, local coordinate system, 11-component | Domain 2 |  |
| solid.eeldevGp12 | solid.gpeval(solid.eeldev12) | 1 | Deviatoric elastic strain tensor, local coordinate system, 12-component | Domain 2 |  |
| solid.eeldevGp13 | solid.gpeval(solid.eeldev13) | 1 | Deviatoric elastic strain tensor, local coordinate system, 13-component | Domain 2 |  |
| solid.eeldevGp22 | solid.gpeval(solid.eeldev22) | 1 | Deviatoric elastic strain tensor, local coordinate system, 22-component | Domain 2 |  |
| solid.eeldevGp23 | solid.gpeval(solid.eeldev23) | 1 | Deviatoric elastic strain tensor, local coordinate system, 23-component | Domain 2 |  |
| solid.eeldevGp33 | solid.gpeval(solid.eeldev33) | 1 | Deviatoric elastic strain tensor, local coordinate system, 33-component | Domain 2 |  |
| solid.eelvolGp | solid.gpeval(solid.eelvol) | 1 | Volumetric elastic strain | Domain 2 |  |
| solid.eielGp11 | solid.gpeval(solid.eiel11) | 1 | Inelastic strain tensor, local coordinate system, 11-component | Domain 2 | + operation |
| solid.eielGp12 | solid.gpeval(solid.eiel12) | 1 | Inelastic strain tensor, local coordinate system, 12-component | Domain 2 | + operation |
| solid.eielGp13 | solid.gpeval(solid.eiel13) | 1 | Inelastic strain tensor, local coordinate system, 13-component | Domain 2 | + operation |
| solid.eielGp22 | solid.gpeval(solid.eiel22) | 1 | Inelastic strain tensor, local coordinate system, 22-component | Domain 2 | + operation |
| solid.eielGp23 | solid.gpeval(solid.eiel23) | 1 | Inelastic strain tensor, local coordinate system, 23-component | Domain 2 | + operation |
| solid.eielGp33 | solid.gpeval(solid.eiel33) | 1 | Inelastic strain tensor, local coordinate system, 33-component | Domain 2 | + operation |
| solid.II2eelGp | max(0,solid.gpeval(solid.II2eel)) | 1 | Second invariant of deviatoric elastic strain tensor | Domain 2 |  |
| solid.sxx | solid.SXX | N/m² | Stress tensor, xx-component | Domain 2 |  |
| solid.sxy | solid.SXY | N/m² | Stress tensor, xy-component | Domain 2 |  |
| solid.sxz | solid.SXZ | N/m² | Stress tensor, xz-component | Domain 2 |  |
| solid.syy | solid.SYY | N/m² | Stress tensor, yy-component | Domain 2 |  |
| solid.syz | solid.SYZ | N/m² | Stress tensor, yz-component | Domain 2 |  |
| solid.szz | solid.SZZ | N/m² | Stress tensor, zz-component | Domain 2 |  |
| solid.sl11 | solid.Sl11 | N/m² | Stress tensor, local coordinate system, 11-component | Domain 2 |  |
| solid.sl12 | solid.Sl12 | N/m² | Stress tensor, local coordinate system, 12-component | Domain 2 |  |
| solid.sl13 | solid.Sl13 | N/m² | Stress tensor, local coordinate system, 13-component | Domain 2 |  |
| solid.sl22 | solid.Sl22 | N/m² | Stress tensor, local coordinate system, 22-component | Domain 2 |  |
| solid.sl23 | solid.Sl23 | N/m² | Stress tensor, local coordinate system, 23-component | Domain 2 |  |
| solid.sl33 | solid.Sl33 | N/m² | Stress tensor, local coordinate system, 33-component | Domain 2 |  |
| solid.sdevxx | solid.sxx-(solid.sxx+solid.syy+solid.szz)/3 | N/m² | Deviatoric stress tensor, xx-component | Domain 2 |  |
| solid.sdevxy | solid.sxy | N/m² | Deviatoric stress tensor, xy-component | Domain 2 |  |
| solid.sdevxz | solid.sxz | N/m² | Deviatoric stress tensor, xz-component | Domain 2 |  |
| solid.sdevyy | solid.syy-(solid.sxx+solid.syy+solid.szz)/3 | N/m² | Deviatoric stress tensor, yy-component | Domain 2 |  |
| solid.sdevyz | solid.syz | N/m² | Deviatoric stress tensor, yz-component | Domain 2 |  |
| solid.sdevzz | solid.szz-(solid.sxx+solid.syy+solid.szz)/3 | N/m² | Deviatoric stress tensor, zz-component | Domain 2 |  |
| solid.sldev11 | solid.sl11-(solid.sl11+solid.sl22+solid.sl33)/3 | N/m² | Deviatoric stress tensor, local coordinate system, 11-component | Domain 2 |  |
| solid.sldev12 | solid.sl12 | N/m² | Deviatoric stress tensor, local coordinate system, 12-component | Domain 2 |  |
| solid.sldev13 | solid.sl13 | N/m² | Deviatoric stress tensor, local coordinate system, 13-component | Domain 2 |  |
| solid.sldev22 | solid.sl22-(solid.sl11+solid.sl22+solid.sl33)/3 | N/m² | Deviatoric stress tensor, local coordinate system, 22-component | Domain 2 |  |
| solid.sldev23 | solid.sl23 | N/m² | Deviatoric stress tensor, local coordinate system, 23-component | Domain 2 |  |
| solid.sldev33 | solid.sl33-(solid.sl11+solid.sl22+solid.sl33)/3 | N/m² | Deviatoric stress tensor, local coordinate system, 33-component | Domain 2 |  |
| solid.I1s | solid.sl11+solid.sl22+solid.sl33 | N/m² | First principal invariant of stress | Domain 2 |  |
| solid.I2s | 0.5*(solid.I1s^2-solid.sl11^2-2*solid.sl12^2-2*solid.sl13^2-solid.sl22^2-2*solid.sl23^2-solid.sl33^2) | kg²/(m²·s⁴) | Second principal invariant of stress | Domain 2 |  |
| solid.I3s | solid.sl11*solid.sl22*solid.sl33+2*solid.sl12*solid.sl23*solid.sl13-solid.sl11*solid.sl23^2-solid.sl12^2*solid.sl33-solid.sl22*solid.sl13^2 | kg³/(m³·s⁶) | Third principal invariant of stress | Domain 2 |  |
| solid.II2s | 0.5*(real(solid.sldev11)^2+2*real(solid.sldev12)^2+2*real(solid.sldev13)^2+real(solid.sldev22)^2+2*real(solid.sldev23)^2+real(solid.sldev33)^2) | kg²/(m²·s⁴) | Second invariant of stress deviator | Domain 2 |  |
| solid.II3s | solid.sldev11*solid.sldev22*solid.sldev33+2*solid.sldev12*solid.sldev23*solid.sldev13-solid.sldev11*solid.sldev23^2-solid.sldev12^2*solid.sldev33-solid.sldev22*solid.sldev13^2 | kg³/(m³·s⁶) | Third invariant of stress deviator | Domain 2 |  |
| solid.svol | solid.I1s/3 | N/m² | Hydrostatic stress | Domain 2 |  |
| solid.rdev | sqrteps(2*solid.II2s) | N/m² | Norm of stress deviator | Domain 2 |  |
| solid.thetaL | atan2(sqrt(max(0.14814814814814814*solid.II2s^3-solid.II3s^2,eps)),solid.II3s)/3 | rad | Lode angle | Domain 2 |  |
| solid.Sel11 | solid.Seli11 | N/m² | Elastic second Piola–Kirchhoff stress, local coordinate system, 11-component | Domain 2 |  |
| solid.Sel12 | solid.Seli12 | N/m² | Elastic second Piola–Kirchhoff stress, local coordinate system, 12-component | Domain 2 |  |
| solid.Sel13 | solid.Seli13 | N/m² | Elastic second Piola–Kirchhoff stress, local coordinate system, 13-component | Domain 2 |  |
| solid.Sel22 | solid.Seli22 | N/m² | Elastic second Piola–Kirchhoff stress, local coordinate system, 22-component | Domain 2 |  |
| solid.Sel23 | solid.Seli23 | N/m² | Elastic second Piola–Kirchhoff stress, local coordinate system, 23-component | Domain 2 |  |
| solid.Sel33 | solid.Seli33 | N/m² | Elastic second Piola–Kirchhoff stress, local coordinate system, 33-component | Domain 2 |  |
| solid.Siel11 | 0 | N/m² | Inelastic second Piola–Kirchhoff stress tensor, local coordinate system, 11-component | Domain 2 | + operation |
| solid.Siel12 | 0 | N/m² | Inelastic second Piola–Kirchhoff stress tensor, local coordinate system, 12-component | Domain 2 | + operation |
| solid.Siel13 | 0 | N/m² | Inelastic second Piola–Kirchhoff stress tensor, local coordinate system, 13-component | Domain 2 | + operation |
| solid.Siel22 | 0 | N/m² | Inelastic second Piola–Kirchhoff stress tensor, local coordinate system, 22-component | Domain 2 | + operation |
| solid.Siel23 | 0 | N/m² | Inelastic second Piola–Kirchhoff stress tensor, local coordinate system, 23-component | Domain 2 | + operation |
| solid.Siel33 | 0 | N/m² | Inelastic second Piola–Kirchhoff stress tensor, local coordinate system, 33-component | Domain 2 | + operation |
| solid.SXX | solid.Sl11 | N/m² | Second Piola–Kirchhoff stress, XX-component | Domain 2 |  |
| solid.SXY | solid.Sl12 | N/m² | Second Piola–Kirchhoff stress, XY-component | Domain 2 |  |
| solid.SXZ | solid.Sl13 | N/m² | Second Piola–Kirchhoff stress, XZ-component | Domain 2 |  |
| solid.SYY | solid.Sl22 | N/m² | Second Piola–Kirchhoff stress, YY-component | Domain 2 |  |
| solid.SYZ | solid.Sl23 | N/m² | Second Piola–Kirchhoff stress, YZ-component | Domain 2 |  |
| solid.SZZ | solid.Sl33 | N/m² | Second Piola–Kirchhoff stress, ZZ-component | Domain 2 |  |
| solid.Sl11 | solid.Siel11+solid.Sel11 | N/m² | Second Piola–Kirchhoff stress, local coordinate system, 11-component | Domain 2 | + operation |
| solid.Sl12 | solid.Siel12+solid.Sel12 | N/m² | Second Piola–Kirchhoff stress, local coordinate system, 12-component | Domain 2 | + operation |
| solid.Sl13 | solid.Siel13+solid.Sel13 | N/m² | Second Piola–Kirchhoff stress, local coordinate system, 13-component | Domain 2 | + operation |
| solid.Sl22 | solid.Siel22+solid.Sel22 | N/m² | Second Piola–Kirchhoff stress, local coordinate system, 22-component | Domain 2 | + operation |
| solid.Sl23 | solid.Siel23+solid.Sel23 | N/m² | Second Piola–Kirchhoff stress, local coordinate system, 23-component | Domain 2 | + operation |
| solid.Sl33 | solid.Siel33+solid.Sel33 | N/m² | Second Piola–Kirchhoff stress, local coordinate system, 33-component | Domain 2 | + operation |
| solid.SdevXX | solid.SXX-(solid.SXX+solid.SYY+solid.SZZ)/3 | N/m² | Deviatoric second Piola–Kirchhoff stress, XX-component | Domain 2 |  |
| solid.SdevXY | solid.SXY | N/m² | Deviatoric second Piola–Kirchhoff stress, XY-component | Domain 2 |  |
| solid.SdevXZ | solid.SXZ | N/m² | Deviatoric second Piola–Kirchhoff stress, XZ-component | Domain 2 |  |
| solid.SdevYY | solid.SYY-(solid.SXX+solid.SYY+solid.SZZ)/3 | N/m² | Deviatoric second Piola–Kirchhoff stress, YY-component | Domain 2 |  |
| solid.SdevYZ | solid.SYZ | N/m² | Deviatoric second Piola–Kirchhoff stress, YZ-component | Domain 2 |  |
| solid.SdevZZ | solid.SZZ-(solid.SXX+solid.SYY+solid.SZZ)/3 | N/m² | Deviatoric second Piola–Kirchhoff stress, ZZ-component | Domain 2 |  |
| solid.Sldev11 | solid.Sl11-(solid.Sl11+solid.Sl22+solid.Sl33)/3 | N/m² | Deviatoric second Piola–Kirchhoff stress, local coordinate system, 11-component | Domain 2 |  |
| solid.Sldev12 | solid.Sl12 | N/m² | Deviatoric second Piola–Kirchhoff stress, local coordinate system, 12-component | Domain 2 |  |
| solid.Sldev13 | solid.Sl13 | N/m² | Deviatoric second Piola–Kirchhoff stress, local coordinate system, 13-component | Domain 2 |  |
| solid.Sldev22 | solid.Sl22-(solid.Sl11+solid.Sl22+solid.Sl33)/3 | N/m² | Deviatoric second Piola–Kirchhoff stress, local coordinate system, 22-component | Domain 2 |  |
| solid.Sldev23 | solid.Sl23 | N/m² | Deviatoric second Piola–Kirchhoff stress, local coordinate system, 23-component | Domain 2 |  |
| solid.Sldev33 | solid.Sl33-(solid.Sl11+solid.Sl22+solid.Sl33)/3 | N/m² | Deviatoric second Piola–Kirchhoff stress, local coordinate system, 33-component | Domain 2 |  |
| solid.PxX | solid.SXX | N/m² | First Piola–Kirchhoff stress, xX-component | Domain 2 |  |
| solid.PyX | solid.SXY | N/m² | First Piola–Kirchhoff stress, yX-component | Domain 2 |  |
| solid.PzX | solid.SXZ | N/m² | First Piola–Kirchhoff stress, zX-component | Domain 2 |  |
| solid.PxY | solid.SXY | N/m² | First Piola–Kirchhoff stress, xY-component | Domain 2 |  |
| solid.PyY | solid.SYY | N/m² | First Piola–Kirchhoff stress, yY-component | Domain 2 |  |
| solid.PzY | solid.SYZ | N/m² | First Piola–Kirchhoff stress, zY-component | Domain 2 |  |
| solid.PxZ | solid.SXZ | N/m² | First Piola–Kirchhoff stress, xZ-component | Domain 2 |  |
| solid.PyZ | solid.SYZ | N/m² | First Piola–Kirchhoff stress, yZ-component | Domain 2 |  |
| solid.PzZ | solid.SZZ | N/m² | First Piola–Kirchhoff stress, zZ-component | Domain 2 |  |
| solid.Sil11 | 0 | N/m² | Initial stress tensor, local coordinate system, 11-component | Domain 2 | + operation |
| solid.Sil12 | 0 | N/m² | Initial stress tensor, local coordinate system, 12-component | Domain 2 | + operation |
| solid.Sil13 | 0 | N/m² | Initial stress tensor, local coordinate system, 13-component | Domain 2 | + operation |
| solid.Sil22 | 0 | N/m² | Initial stress tensor, local coordinate system, 22-component | Domain 2 | + operation |
| solid.Sil23 | 0 | N/m² | Initial stress tensor, local coordinate system, 23-component | Domain 2 | + operation |
| solid.Sil33 | 0 | N/m² | Initial stress tensor, local coordinate system, 33-component | Domain 2 | + operation |
| solid.mises_peak | sqrteps(solid.lemm1.cvector1+solid.lemm1.cvector2+solid.lemm1.cvector3+solid.lemm1.cvector4+solid.lemm1.cvector5+solid.lemm1.cvector6+solid.lemm1.cvector7+solid.lemm1.cvector8+solid.lemm1.cvector9+sqrteps(solid.lemm1.avector1^2+solid.lemm1.avector1*solid.lemm1.avector2*cos(solid.lemm1.bvector1-solid.lemm1.bvector2)+solid.lemm1.avector1*solid.lemm1.avector3*cos(solid.lemm1.bvector1-solid.lemm1.bvector3)+solid.lemm1.avector1*solid.lemm1.avector4*cos(solid.lemm1.bvector1-solid.lemm1.bvector4)+solid.lemm1.avector1*solid.lemm1.avector5*cos(solid.lemm1.bvector1-solid.lemm1.bvector5)+solid.lemm1.avector1*solid.lemm1.avector6*cos(solid.lemm1.bvector1-solid.lemm1.bvector6)+solid.lemm1.avector1*solid.lemm1.avector7*cos(solid.lemm1.bvector1-solid.lemm1.bvector7)+solid.lemm1.avector1*solid.lemm1.avector8*cos(solid.lemm1.bvector1-solid.lemm1.bvector8)+solid.lemm1.avector1*solid.lemm1.avector9*cos(solid.lemm1.bvector1-solid.lemm1.bvector9)+solid.lemm1.avector2*solid.lemm1.avector1*cos(solid.lemm1.bvector2-solid.lemm1.bvector1)+solid.lemm1.avector2^2+solid.lemm1.avector2*solid.lemm1.avector3*cos(solid.lemm1.bvector2-solid.lemm1.bvector3)+solid.lemm1.avector2*solid.lemm1.avector4*cos(solid.lemm1.bvector2-solid.lemm1.bvector4)+solid.lemm1.avector2*solid.lemm1.avector5*cos(solid.lemm1.bvector2-solid.lemm1.bvector5)+solid.lemm1.avector2*solid.lemm1.avector6*cos(solid.lemm1.bvector2-solid.lemm1.bvector6)+solid.lemm1.avector2*solid.lemm1.avector7*cos(solid.lemm1.bvector2-solid.lemm1.bvector7)+solid.lemm1.avector2*solid.lemm1.avector8*cos(solid.lemm1.bvector2-solid.lemm1.bvector8)+solid.lemm1.avector2*solid.lemm1.avector9*cos(solid.lemm1.bvector2-solid.lemm1.bvector9)+solid.lemm1.avector3*solid.lemm1.avector1*cos(solid.lemm1.bvector3-solid.lemm1.bvector1)+solid.lemm1.avector3*solid.lemm1.avector2*cos(solid.lemm1.bvector3-solid.lemm1.bvector2)+solid.lemm1.avector3^2+solid.lemm1.avector3*solid.lemm1.avector4*cos(solid.lemm1.bvector3-solid.lemm1.bvector4)+solid.lemm1.avector3*solid.lemm1.avector5*cos(solid.lemm1.bvector3-solid.lemm1.bvector5)+solid.lemm1.avector3*solid.lemm1.avector6*cos(solid.lemm1.bvector3-solid.lemm1.bvector6)+solid.lemm1.avector3*solid.lemm1.avector7*cos(solid.lemm1.bvector3-solid.lemm1.bvector7)+solid.lemm1.avector3*solid.lemm1.avector8*cos(solid.lemm1.bvector3-solid.lemm1.bvector8)+solid.lemm1.avector3*solid.lemm1.avector9*cos(solid.lemm1.bvector3-solid.lemm1.bvector9)+solid.lemm1.avector4*solid.lemm1.avector1*cos(solid.lemm1.bvector4-solid.lemm1.bvector1)+solid.lemm1.avector4*solid.lemm1.avector2*cos(solid.lemm1.bvector4-solid.lemm1.bvector2)+solid.lemm1.avector4*solid.lemm1.avector3*cos(solid.lemm1.bvector4-solid.lemm1.bvector3)+solid.lemm1.avector4^2+solid.lemm1.avector4*solid.lemm1.avector5*cos(solid.lemm1.bvector4-solid.lemm1.bvector5)+solid.lemm1.avector4*solid.lemm1.avector6*cos(solid.lemm1.bvector4-solid.lemm1.bvector6)+solid.lemm1.avector4*solid.lemm1.avector7*cos(solid.lemm1.bvector4-solid.lemm1.bvector7)+solid.lemm1.avector4*solid.lemm1.avector8*cos(solid.lemm1.bvector4-solid.lemm1.bvector8)+solid.lemm1.avector4*solid.lemm1.avector9*cos(solid.lemm1.bvector4-solid.lemm1.bvector9)+solid.lemm1.avector5*solid.lemm1.avector1*cos(solid.lemm1.bvector5-solid.lemm1.bvector1)+solid.lemm1.avector5*solid.lemm1.avector2*cos(solid.lemm1.bvector5-solid.lemm1.bvector2)+solid.lemm1.avector5*solid.lemm1.avector3*cos(solid.lemm1.bvector5-solid.lemm1.bvector3)+solid.lemm1.avector5*solid.lemm1.avector4*cos(solid.lemm1.bvector5-solid.lemm1.bvector4)+solid.lemm1.avector5^2+solid.lemm1.avector5*solid.lemm1.avector6*cos(solid.lemm1.bvector5-solid.lemm1.bvector6)+solid.lemm1.avector5*solid.lemm1.avector7*cos(solid.lemm1.bvector5-solid.lemm1.bvector7)+solid.lemm1.avector5*solid.lemm1.avector8*cos(solid.lemm1.bvector5-solid.lemm1.bvector8)+solid.lemm1.avector5*solid.lemm1.avector9*cos(solid.lemm1.bvector5-solid.lemm1.bvector9)+solid.lemm1.avector6*solid.lemm1.avector1*cos(solid.lemm1.bvector6-solid.lemm1.bvector1)+solid.lemm1.avector6*solid.lemm1.avector2*cos(solid.lemm1.bvector6-solid.lemm1.bvector2)+solid.lemm1.avector6*solid.lemm1.avector3*cos(solid.lemm1.bvector6-solid.lemm1.bvector3)+solid.lemm1.avector6*solid.lemm1.avector4*cos(solid.lemm1.bvector6-solid.lemm1.bvector4)+solid.lemm1.avector6*solid.lemm1.avector5*cos(solid.lemm1.bvector6-solid.lemm1.bvector5)+solid.lemm1.avector6^2+solid.lemm1.avector6*solid.lemm1.avector7*cos(solid.lemm1.bvector6-solid.lemm1.bvector7)+solid.lemm1.avector6*solid.lemm1.avector8*cos(solid.lemm1.bvector6-solid.lemm1.bvector8)+solid.lemm1.avector6*solid.lemm1.avector9*cos(solid.lemm1.bvector6-solid.lemm1.bvector9)+solid.lemm1.avector7*solid.lemm1.avector1*cos(solid.lemm1.bvector7-solid.lemm1.bvector1)+solid.lemm1.avector7*solid.lemm1.avector2*cos(solid.lemm1.bvector7-solid.lemm1.bvector2)+solid.lemm1.avector7*solid.lemm1.avector3*cos(solid.lemm1.bvector7-solid.lemm1.bvector3)+solid.lemm1.avector7*solid.lemm1.avector4*cos(solid.lemm1.bvector7-solid.lemm1.bvector4)+solid.lemm1.avector7*solid.lemm1.avector5*cos(solid.lemm1.bvector7-solid.lemm1.bvector5)+solid.lemm1.avector7*solid.lemm1.avector6*cos(solid.lemm1.bvector7-solid.lemm1.bvector6)+solid.lemm1.avector7^2+solid.lemm1.avector7*solid.lemm1.avector8*cos(solid.lemm1.bvector7-solid.lemm1.bvector8)+solid.lemm1.avector7*solid.lemm1.avector9*cos(solid.lemm1.bvector7-solid.lemm1.bvector9)+solid.lemm1.avector8*solid.lemm1.avector1*cos(solid.lemm1.bvector8-solid.lemm1.bvector1)+solid.lemm1.avector8*solid.lemm1.avector2*cos(solid.lemm1.bvector8-solid.lemm1.bvector2)+solid.lemm1.avector8*solid.lemm1.avector3*cos(solid.lemm1.bvector8-solid.lemm1.bvector3)+solid.lemm1.avector8*solid.lemm1.avector4*cos(solid.lemm1.bvector8-solid.lemm1.bvector4)+solid.lemm1.avector8*solid.lemm1.avector5*cos(solid.lemm1.bvector8-solid.lemm1.bvector5)+solid.lemm1.avector8*solid.lemm1.avector6*cos(solid.lemm1.bvector8-solid.lemm1.bvector6)+solid.lemm1.avector8*solid.lemm1.avector7*cos(solid.lemm1.bvector8-solid.lemm1.bvector7)+solid.lemm1.avector8^2+solid.lemm1.avector8*solid.lemm1.avector9*cos(solid.lemm1.bvector8-solid.lemm1.bvector9)+solid.lemm1.avector9*solid.lemm1.avector1*cos(solid.lemm1.bvector9-solid.lemm1.bvector1)+solid.lemm1.avector9*solid.lemm1.avector2*cos(solid.lemm1.bvector9-solid.lemm1.bvector2)+solid.lemm1.avector9*solid.lemm1.avector3*cos(solid.lemm1.bvector9-solid.lemm1.bvector3)+solid.lemm1.avector9*solid.lemm1.avector4*cos(solid.lemm1.bvector9-solid.lemm1.bvector4)+solid.lemm1.avector9*solid.lemm1.avector5*cos(solid.lemm1.bvector9-solid.lemm1.bvector5)+solid.lemm1.avector9*solid.lemm1.avector6*cos(solid.lemm1.bvector9-solid.lemm1.bvector6)+solid.lemm1.avector9*solid.lemm1.avector7*cos(solid.lemm1.bvector9-solid.lemm1.bvector7)+solid.lemm1.avector9*solid.lemm1.avector8*cos(solid.lemm1.bvector9-solid.lemm1.bvector8)+solid.lemm1.avector9^2)) | N/m² | Von Mises stress, peak | Domain 2 |  |
| solid.tresca | max(max(abs(solid.sp1-solid.sp2),abs(solid.sp1-solid.sp3)),abs(solid.sp2-solid.sp3)) | N/m² | Tresca stress | Domain 2 |  |
| solid.mises | sqrteps(3*solid.II2s) | N/m² | von Mises stress | Domain 2 |  |
| solid.pm | -(solid.sl11+solid.sl22+solid.sl33)/3 | N/m² | Pressure | Domain 2 |  |
| solid.Seli11 | solid.D11*solid.eel11+solid.D12*solid.eel22+solid.D13*solid.eel33+2*solid.D14*solid.eel12+2*solid.D15*solid.eel23+2*solid.D16*solid.eel13 | N/m² | Elastic second Piola–Kirchhoff stress, intermediate configuration, 11-component | Domain 2 |  |
| solid.Seli12 | solid.D14*solid.eel11+solid.D24*solid.eel22+solid.D34*solid.eel33+2*solid.D44*solid.eel12+2*solid.D45*solid.eel23+2*solid.D46*solid.eel13 | N/m² | Elastic second Piola–Kirchhoff stress, intermediate configuration, 12-component | Domain 2 |  |
| solid.Seli13 | solid.D16*solid.eel11+solid.D26*solid.eel22+solid.D36*solid.eel33+2*solid.D46*solid.eel12+2*solid.D56*solid.eel23+2*solid.D66*solid.eel13 | N/m² | Elastic second Piola–Kirchhoff stress, intermediate configuration, 13-component | Domain 2 |  |
| solid.Seli22 | solid.D12*solid.eel11+solid.D22*solid.eel22+solid.D23*solid.eel33+2*solid.D24*solid.eel12+2*solid.D25*solid.eel23+2*solid.D26*solid.eel13 | N/m² | Elastic second Piola–Kirchhoff stress, intermediate configuration, 22-component | Domain 2 |  |
| solid.Seli23 | solid.D15*solid.eel11+solid.D25*solid.eel22+solid.D35*solid.eel33+2*solid.D45*solid.eel12+2*solid.D55*solid.eel23+2*solid.D56*solid.eel13 | N/m² | Elastic second Piola–Kirchhoff stress, intermediate configuration, 23-component | Domain 2 |  |
| solid.Seli33 | solid.D13*solid.eel11+solid.D23*solid.eel22+solid.D33*solid.eel33+2*solid.D34*solid.eel12+2*solid.D35*solid.eel23+2*solid.D36*solid.eel13 | N/m² | Elastic second Piola–Kirchhoff stress, intermediate configuration, 33-component | Domain 2 |  |
| solid.Sieli11 | solid.Siel11 | N/m² | Inelastic second Piola–Kirchhoff stress tensor, intermediate configuration, 11-component | Domain 2 |  |
| solid.Sieli12 | solid.Siel12 | N/m² | Inelastic second Piola–Kirchhoff stress tensor, intermediate configuration, 12-component | Domain 2 |  |
| solid.Sieli13 | solid.Siel13 | N/m² | Inelastic second Piola–Kirchhoff stress tensor, intermediate configuration, 13-component | Domain 2 |  |
| solid.Sieli22 | solid.Siel22 | N/m² | Inelastic second Piola–Kirchhoff stress tensor, intermediate configuration, 22-component | Domain 2 |  |
| solid.Sieli23 | solid.Siel23 | N/m² | Inelastic second Piola–Kirchhoff stress tensor, intermediate configuration, 23-component | Domain 2 |  |
| solid.Sieli33 | solid.Siel33 | N/m² | Inelastic second Piola–Kirchhoff stress tensor, intermediate configuration, 33-component | Domain 2 |  |
| solid.Mandel11 | solid.Sel11 | N/m² | Mandel stress, local coordinate system, 11-component | Domain 2 |  |
| solid.Mandel21 | solid.Sel12 | N/m² | Mandel stress, local coordinate system, 21-component | Domain 2 |  |
| solid.Mandel31 | solid.Sel13 | N/m² | Mandel stress, local coordinate system, 31-component | Domain 2 |  |
| solid.Mandel12 | solid.Sel12 | N/m² | Mandel stress, local coordinate system, 12-component | Domain 2 |  |
| solid.Mandel22 | solid.Sel22 | N/m² | Mandel stress, local coordinate system, 22-component | Domain 2 |  |
| solid.Mandel32 | solid.Sel23 | N/m² | Mandel stress, local coordinate system, 32-component | Domain 2 |  |
| solid.Mandel13 | solid.Sel13 | N/m² | Mandel stress, local coordinate system, 13-component | Domain 2 |  |
| solid.Mandel23 | solid.Sel23 | N/m² | Mandel stress, local coordinate system, 23-component | Domain 2 |  |
| solid.Mandel33 | solid.Sel33 | N/m² | Mandel stress, local coordinate system, 33-component | Domain 2 |  |
| solid.sGpxx | solid.gpeval(solid.sxx) | N/m² | Stress tensor, xx-component | Domain 2 |  |
| solid.sGpxy | solid.gpeval(solid.sxy) | N/m² | Stress tensor, xy-component | Domain 2 |  |
| solid.sGpxz | solid.gpeval(solid.sxz) | N/m² | Stress tensor, xz-component | Domain 2 |  |
| solid.sGpyy | solid.gpeval(solid.syy) | N/m² | Stress tensor, yy-component | Domain 2 |  |
| solid.sGpyz | solid.gpeval(solid.syz) | N/m² | Stress tensor, yz-component | Domain 2 |  |
| solid.sGpzz | solid.gpeval(solid.szz) | N/m² | Stress tensor, zz-component | Domain 2 |  |
| solid.slGp11 | solid.gpeval(solid.sl11) | N/m² | Stress tensor, local coordinate system, 11-component | Domain 2 |  |
| solid.slGp12 | solid.gpeval(solid.sl12) | N/m² | Stress tensor, local coordinate system, 12-component | Domain 2 |  |
| solid.slGp13 | solid.gpeval(solid.sl13) | N/m² | Stress tensor, local coordinate system, 13-component | Domain 2 |  |
| solid.slGp22 | solid.gpeval(solid.sl22) | N/m² | Stress tensor, local coordinate system, 22-component | Domain 2 |  |
| solid.slGp23 | solid.gpeval(solid.sl23) | N/m² | Stress tensor, local coordinate system, 23-component | Domain 2 |  |
| solid.slGp33 | solid.gpeval(solid.sl33) | N/m² | Stress tensor, local coordinate system, 33-component | Domain 2 |  |
| solid.sdevGpxx | solid.gpeval(solid.sdevxx) | N/m² | Deviatoric stress tensor, xx-component | Domain 2 |  |
| solid.sdevGpxy | solid.gpeval(solid.sdevxy) | N/m² | Deviatoric stress tensor, xy-component | Domain 2 |  |
| solid.sdevGpxz | solid.gpeval(solid.sdevxz) | N/m² | Deviatoric stress tensor, xz-component | Domain 2 |  |
| solid.sdevGpyy | solid.gpeval(solid.sdevyy) | N/m² | Deviatoric stress tensor, yy-component | Domain 2 |  |
| solid.sdevGpyz | solid.gpeval(solid.sdevyz) | N/m² | Deviatoric stress tensor, yz-component | Domain 2 |  |
| solid.sdevGpzz | solid.gpeval(solid.sdevzz) | N/m² | Deviatoric stress tensor, zz-component | Domain 2 |  |
| solid.sldevGp11 | solid.gpeval(solid.sldev11) | N/m² | Deviatoric stress tensor, 11-component | Domain 2 |  |
| solid.sldevGp12 | solid.gpeval(solid.sldev12) | N/m² | Deviatoric stress tensor, 12-component | Domain 2 |  |
| solid.sldevGp13 | solid.gpeval(solid.sldev13) | N/m² | Deviatoric stress tensor, 13-component | Domain 2 |  |
| solid.sldevGp22 | solid.gpeval(solid.sldev22) | N/m² | Deviatoric stress tensor, 22-component | Domain 2 |  |
| solid.sldevGp23 | solid.gpeval(solid.sldev23) | N/m² | Deviatoric stress tensor, 23-component | Domain 2 |  |
| solid.sldevGp33 | solid.gpeval(solid.sldev33) | N/m² | Deviatoric stress tensor, 33-component | Domain 2 |  |
| solid.I1sGp | solid.gpeval(solid.I1s) | N/m² | First principal invariant of stress | Domain 2 |  |
| solid.I2sGp | max(0,solid.gpeval(solid.I2s)) | kg²/(m²·s⁴) | Second principal invariant of stress | Domain 2 |  |
| solid.I3sGp | solid.gpeval(solid.I3s) | kg³/(m³·s⁶) | Third principal invariant of stress | Domain 2 |  |
| solid.II3sGp | solid.gpeval(solid.II3s) | kg³/(m³·s⁶) | Third invariant of stress deviator | Domain 2 |  |
| solid.svolGp | solid.gpeval(solid.svol) | N/m² | Hydrostatic stress | Domain 2 |  |
| solid.rdevGp | max(0,solid.gpeval(solid.rdev)) | N/m² | Norm of stress deviator | Domain 2 |  |
| solid.thetaLGp | solid.gpeval(solid.thetaL) | rad | Lode angle | Domain 2 |  |
| solid.SGpXX | solid.gpeval(solid.SXX) | N/m² | Second Piola–Kirchhoff stress, XX-component | Domain 2 |  |
| solid.SGpXY | solid.gpeval(solid.SXY) | N/m² | Second Piola–Kirchhoff stress, XY-component | Domain 2 |  |
| solid.SGpXZ | solid.gpeval(solid.SXZ) | N/m² | Second Piola–Kirchhoff stress, XZ-component | Domain 2 |  |
| solid.SGpYY | solid.gpeval(solid.SYY) | N/m² | Second Piola–Kirchhoff stress, YY-component | Domain 2 |  |
| solid.SGpYZ | solid.gpeval(solid.SYZ) | N/m² | Second Piola–Kirchhoff stress, YZ-component | Domain 2 |  |
| solid.SGpZZ | solid.gpeval(solid.SZZ) | N/m² | Second Piola–Kirchhoff stress, ZZ-component | Domain 2 |  |
| solid.SlGp11 | solid.gpeval(solid.Sl11) | N/m² | Second Piola–Kirchhoff stress, local coordinate system, 11-component | Domain 2 |  |
| solid.SlGp12 | solid.gpeval(solid.Sl12) | N/m² | Second Piola–Kirchhoff stress, local coordinate system, 12-component | Domain 2 |  |
| solid.SlGp13 | solid.gpeval(solid.Sl13) | N/m² | Second Piola–Kirchhoff stress, local coordinate system, 13-component | Domain 2 |  |
| solid.SlGp22 | solid.gpeval(solid.Sl22) | N/m² | Second Piola–Kirchhoff stress, local coordinate system, 22-component | Domain 2 |  |
| solid.SlGp23 | solid.gpeval(solid.Sl23) | N/m² | Second Piola–Kirchhoff stress, local coordinate system, 23-component | Domain 2 |  |
| solid.SlGp33 | solid.gpeval(solid.Sl33) | N/m² | Second Piola–Kirchhoff stress, local coordinate system, 33-component | Domain 2 |  |
| solid.SdevGpXX | solid.gpeval(solid.SdevXX) | N/m² | Deviatoric second Piola–Kirchhoff stress, XX-component | Domain 2 |  |
| solid.SdevGpXY | solid.gpeval(solid.SdevXY) | N/m² | Deviatoric second Piola–Kirchhoff stress, XY-component | Domain 2 |  |
| solid.SdevGpXZ | solid.gpeval(solid.SdevXZ) | N/m² | Deviatoric second Piola–Kirchhoff stress, XZ-component | Domain 2 |  |
| solid.SdevGpYY | solid.gpeval(solid.SdevYY) | N/m² | Deviatoric second Piola–Kirchhoff stress, YY-component | Domain 2 |  |
| solid.SdevGpYZ | solid.gpeval(solid.SdevYZ) | N/m² | Deviatoric second Piola–Kirchhoff stress, YZ-component | Domain 2 |  |
| solid.SdevGpZZ | solid.gpeval(solid.SdevZZ) | N/m² | Deviatoric second Piola–Kirchhoff stress, ZZ-component | Domain 2 |  |
| solid.SldevGp11 | solid.gpeval(solid.Sldev11) | N/m² | Deviatoric second Piola–Kirchhoff stress, local coordinate system, 11-component | Domain 2 |  |
| solid.SldevGp12 | solid.gpeval(solid.Sldev12) | N/m² | Deviatoric second Piola–Kirchhoff stress, local coordinate system, 12-component | Domain 2 |  |
| solid.SldevGp13 | solid.gpeval(solid.Sldev13) | N/m² | Deviatoric second Piola–Kirchhoff stress, local coordinate system, 13-component | Domain 2 |  |
| solid.SldevGp22 | solid.gpeval(solid.Sldev22) | N/m² | Deviatoric second Piola–Kirchhoff stress, local coordinate system, 22-component | Domain 2 |  |
| solid.SldevGp23 | solid.gpeval(solid.Sldev23) | N/m² | Deviatoric second Piola–Kirchhoff stress, local coordinate system, 23-component | Domain 2 |  |
| solid.SldevGp33 | solid.gpeval(solid.Sldev33) | N/m² | Deviatoric second Piola–Kirchhoff stress, local coordinate system, 33-component | Domain 2 |  |
| solid.PGpxX | solid.gpeval(solid.PxX) | N/m² | First Piola–Kirchhoff stress, xX-component | Domain 2 |  |
| solid.PGpyX | solid.gpeval(solid.PyX) | N/m² | First Piola–Kirchhoff stress, yX-component | Domain 2 |  |
| solid.PGpzX | solid.gpeval(solid.PzX) | N/m² | First Piola–Kirchhoff stress, zX-component | Domain 2 |  |
| solid.PGpxY | solid.gpeval(solid.PxY) | N/m² | First Piola–Kirchhoff stress, xY-component | Domain 2 |  |
| solid.PGpyY | solid.gpeval(solid.PyY) | N/m² | First Piola–Kirchhoff stress, yY-component | Domain 2 |  |
| solid.PGpzY | solid.gpeval(solid.PzY) | N/m² | First Piola–Kirchhoff stress, zY-component | Domain 2 |  |
| solid.PGpxZ | solid.gpeval(solid.PxZ) | N/m² | First Piola–Kirchhoff stress, xZ-component | Domain 2 |  |
| solid.PGpyZ | solid.gpeval(solid.PyZ) | N/m² | First Piola–Kirchhoff stress, yZ-component | Domain 2 |  |
| solid.PGpzZ | solid.gpeval(solid.PzZ) | N/m² | First Piola–Kirchhoff stress, zZ-component | Domain 2 |  |
| solid.PlGpx1 | solid.gpeval(solid.Plx1) | N/m² | First Piola–Kirchhoff stress, local coordinate system, x1-component | Domain 2 |  |
| solid.PlGpy1 | solid.gpeval(solid.Ply1) | N/m² | First Piola–Kirchhoff stress, local coordinate system, y1-component | Domain 2 |  |
| solid.PlGpz1 | solid.gpeval(solid.Plz1) | N/m² | First Piola–Kirchhoff stress, local coordinate system, z1-component | Domain 2 |  |
| solid.PlGpx2 | solid.gpeval(solid.Plx2) | N/m² | First Piola–Kirchhoff stress, local coordinate system, x2-component | Domain 2 |  |
| solid.PlGpy2 | solid.gpeval(solid.Ply2) | N/m² | First Piola–Kirchhoff stress, local coordinate system, y2-component | Domain 2 |  |
| solid.PlGpz2 | solid.gpeval(solid.Plz2) | N/m² | First Piola–Kirchhoff stress, local coordinate system, z2-component | Domain 2 |  |
| solid.PlGpx3 | solid.gpeval(solid.Plx3) | N/m² | First Piola–Kirchhoff stress, local coordinate system, x3-component | Domain 2 |  |
| solid.PlGpy3 | solid.gpeval(solid.Ply3) | N/m² | First Piola–Kirchhoff stress, local coordinate system, y3-component | Domain 2 |  |
| solid.PlGpz3 | solid.gpeval(solid.Plz3) | N/m² | First Piola–Kirchhoff stress, local coordinate system, z3-component | Domain 2 |  |
| solid.misesGp | max(0,solid.gpeval(solid.mises)) | N/m² | von Mises stress | Domain 2 |  |
| solid.misesGp_peak | max(0,solid.gpeval(solid.mises_peak)) | N/m² | Von Mises stress, peak | Domain 2 |  |
| solid.trescaGp | max(0,solid.gpeval(solid.tresca)) | N/m² | Tresca stress | Domain 2 |  |
| solid.pmGp | solid.gpeval(solid.pm) | N/m² | Pressure | Domain 2 |  |
| solid.Ws | 0.5*solid.Ji*(solid.Sel11*solid.eel11+2*solid.Sel12*solid.eel12+2*solid.Sel13*solid.eel13+solid.Sel22*solid.eel22+2*solid.Sel23*solid.eel23+solid.Sel33*solid.eel33) | J/m³ | Elastic strain energy density | Domain 2 | + operation |
| solid.WsGp | max(0,solid.gpeval(solid.Ws)) | J/m³ | Elastic strain energy density | Domain 2 |  |
| solid.Ws_tot | solid.lemm1.intDom(solid.Ws*solid.lemm1.integrand*solid.lemm1.gsv) | J | Total elastic strain energy | Global | + operation |
| solid.Ws_current | 0.5*solid.Ji*(real(solid.Sel11)*real(solid.eel11)+2*real(solid.Sel12)*real(solid.eel12)+2*real(solid.Sel13)*real(solid.eel13)+real(solid.Sel22)*real(solid.eel22)+2*real(solid.Sel23)*real(solid.eel23)+real(solid.Sel33)*real(solid.eel33)) | J/m³ | Elastic strain energy density, current phase | Domain 2 | + operation |
| solid.Wh | 0.25*solid.Ji*real(solid.eel11*conj(solid.Sel11)+2*solid.eel12*conj(solid.Sel12)+2*solid.eel13*conj(solid.Sel13)+solid.eel22*conj(solid.Sel22)+2*solid.eel23*conj(solid.Sel23)+solid.eel33*conj(solid.Sel33))*!isPML | J/m³ | Stored energy density | Domain 2 | + operation |
| solid.WhGp | max(0,solid.gpeval(solid.Wh)) | J/m³ | Stored energy density | Domain 2 |  |
| solid.Wh_tot | solid.lemm1.intDom(solid.Wh*solid.lemm1.integrand*solid.lemm1.gsv) | J | Total stored energy | Global | + operation |
| solid.Wk | 0.25*solid.rho*(realdot(u*solid.iomega,u*solid.iomega)+realdot(v*solid.iomega,v*solid.iomega))*!isPML | J/m³ | Kinetic energy density | Domain 2 | + operation |
| solid.Wk_tot | solid.lemm1.intDomFull(solid.Wk*solid.lemm1.integrand*solid.lemm1.gsv) | J | Total kinetic energy | Global | + operation |
| solid.Eequ | solid.E | Pa | Equivalent Young's modulus | Domain 2 |  |
| solid.nuequ | solid.nu | 1 | Equivalent Poisson's ratio | Domain 2 |  |
| solid.Gequ | solid.G | N/m² | Equivalent shear modulus | Domain 2 |  |
| solid.Kequ | solid.Eequ*solid.Gequ/(3*(3*solid.Gequ-solid.Eequ)) | N/m² | Equivalent bulk modulus | Domain 2 |  |
| solid.Eequtot | solid.Eequ | Pa | Total equivalent Young's modulus | Domain 2 | + operation |
| solid.Gequtot | solid.Gequ | N/m² | Total equivalent shear modulus | Domain 2 | + operation |
| solid.Kequtot | solid.Kequ | N/m² | Total equivalent bulk modulus | Domain 2 | + operation |
| solid.K | solid.E/(3*(1-2*solid.nu)) | N/m² | Bulk modulus | Domain 2 | * operation |
| solid.D11 | solid.E*(1-solid.nu)/((1+solid.nu)*(1-2*solid.nu)) | Pa | Elasticity matrix, 11-component | Domain 2 | * operation |
| solid.D12 | solid.E*solid.nu/((1+solid.nu)*(1-2*solid.nu)) | Pa | Elasticity matrix, 12-component | Domain 2 | * operation |
| solid.D13 | solid.E*solid.nu/((1+solid.nu)*(1-2*solid.nu)) | Pa | Elasticity matrix, 13-component | Domain 2 | * operation |
| solid.D14 | 0 | Pa | Elasticity matrix, 14-component | Domain 2 | * operation |
| solid.D15 | 0 | Pa | Elasticity matrix, 15-component | Domain 2 | * operation |
| solid.D16 | 0 | Pa | Elasticity matrix, 16-component | Domain 2 | * operation |
| solid.D22 | solid.E*(1-solid.nu)/((1+solid.nu)*(1-2*solid.nu)) | Pa | Elasticity matrix, 22-component | Domain 2 | * operation |
| solid.D23 | solid.E*solid.nu/((1+solid.nu)*(1-2*solid.nu)) | Pa | Elasticity matrix, 23-component | Domain 2 | * operation |
| solid.D24 | 0 | Pa | Elasticity matrix, 24-component | Domain 2 | * operation |
| solid.D25 | 0 | Pa | Elasticity matrix, 25-component | Domain 2 | * operation |
| solid.D26 | 0 | Pa | Elasticity matrix, 26-component | Domain 2 | * operation |
| solid.D33 | solid.E*(1-solid.nu)/((1+solid.nu)*(1-2*solid.nu)) | Pa | Elasticity matrix, 33-component | Domain 2 | * operation |
| solid.D34 | 0 | Pa | Elasticity matrix, 34-component | Domain 2 | * operation |
| solid.D35 | 0 | Pa | Elasticity matrix, 35-component | Domain 2 | * operation |
| solid.D36 | 0 | Pa | Elasticity matrix, 36-component | Domain 2 | * operation |
| solid.D44 | 0.5*solid.E/(1+solid.nu) | Pa | Elasticity matrix, 44-component | Domain 2 | * operation |
| solid.D45 | 0 | Pa | Elasticity matrix, 45-component | Domain 2 | * operation |
| solid.D46 | 0 | Pa | Elasticity matrix, 46-component | Domain 2 | * operation |
| solid.D55 | 0.5*solid.E/(1+solid.nu) | Pa | Elasticity matrix, 55-component | Domain 2 | * operation |
| solid.D56 | 0 | Pa | Elasticity matrix, 56-component | Domain 2 | * operation |
| solid.D66 | 0.5*solid.E/(1+solid.nu) | Pa | Elasticity matrix, 66-component | Domain 2 | * operation |
| solid.DVo11 | solid.D11 | Pa | Elasticity matrix, Voigt notation, 11-component | Domain 2 |  |
| solid.DVo12 | solid.D12 | Pa | Elasticity matrix, Voigt notation, 12-component | Domain 2 |  |
| solid.DVo13 | solid.D13 | Pa | Elasticity matrix, Voigt notation, 13-component | Domain 2 |  |
| solid.DVo14 | solid.D15 | Pa | Elasticity matrix, Voigt notation, 14-component | Domain 2 |  |
| solid.DVo15 | solid.D16 | Pa | Elasticity matrix, Voigt notation, 15-component | Domain 2 |  |
| solid.DVo16 | solid.D14 | Pa | Elasticity matrix, Voigt notation, 16-component | Domain 2 |  |
| solid.DVo22 | solid.D22 | Pa | Elasticity matrix, Voigt notation, 22-component | Domain 2 |  |
| solid.DVo23 | solid.D23 | Pa | Elasticity matrix, Voigt notation, 23-component | Domain 2 |  |
| solid.DVo24 | solid.D25 | Pa | Elasticity matrix, Voigt notation, 24-component | Domain 2 |  |
| solid.DVo25 | solid.D26 | Pa | Elasticity matrix, Voigt notation, 25-component | Domain 2 |  |
| solid.DVo26 | solid.D24 | Pa | Elasticity matrix, Voigt notation, 26-component | Domain 2 |  |
| solid.DVo33 | solid.D33 | Pa | Elasticity matrix, Voigt notation, 33-component | Domain 2 |  |
| solid.DVo34 | solid.D35 | Pa | Elasticity matrix, Voigt notation, 34-component | Domain 2 |  |
| solid.DVo35 | solid.D36 | Pa | Elasticity matrix, Voigt notation, 35-component | Domain 2 |  |
| solid.DVo36 | solid.D34 | Pa | Elasticity matrix, Voigt notation, 36-component | Domain 2 |  |
| solid.DVo44 | solid.D55 | Pa | Elasticity matrix, Voigt notation, 44-component | Domain 2 |  |
| solid.DVo45 | solid.D56 | Pa | Elasticity matrix, Voigt notation, 45-component | Domain 2 |  |
| solid.DVo46 | solid.D45 | Pa | Elasticity matrix, Voigt notation, 46-component | Domain 2 |  |
| solid.DVo55 | solid.D66 | Pa | Elasticity matrix, Voigt notation, 55-component | Domain 2 |  |
| solid.DVo56 | solid.D46 | Pa | Elasticity matrix, Voigt notation, 56-component | Domain 2 |  |
| solid.DVo66 | solid.D44 | Pa | Elasticity matrix, Voigt notation, 66-component | Domain 2 |  |
| solid.Dg11 | solid.D11 | Pa | Elasticity matrix (global), 11-component | Domain 2 |  |
| solid.Dg12 | solid.D12 | Pa | Elasticity matrix (global), 12-component | Domain 2 |  |
| solid.Dg13 | solid.D13 | Pa | Elasticity matrix (global), 13-component | Domain 2 |  |
| solid.Dg14 | solid.D14 | Pa | Elasticity matrix (global), 14-component | Domain 2 |  |
| solid.Dg15 | solid.D15 | Pa | Elasticity matrix (global), 15-component | Domain 2 |  |
| solid.Dg16 | solid.D16 | Pa | Elasticity matrix (global), 16-component | Domain 2 |  |
| solid.Dg22 | solid.D22 | Pa | Elasticity matrix (global), 22-component | Domain 2 |  |
| solid.Dg23 | solid.D23 | Pa | Elasticity matrix (global), 23-component | Domain 2 |  |
| solid.Dg24 | solid.D24 | Pa | Elasticity matrix (global), 24-component | Domain 2 |  |
| solid.Dg25 | solid.D25 | Pa | Elasticity matrix (global), 25-component | Domain 2 |  |
| solid.Dg26 | solid.D26 | Pa | Elasticity matrix (global), 26-component | Domain 2 |  |
| solid.Dg33 | solid.D33 | Pa | Elasticity matrix (global), 33-component | Domain 2 |  |
| solid.Dg34 | solid.D34 | Pa | Elasticity matrix (global), 34-component | Domain 2 |  |
| solid.Dg35 | solid.D35 | Pa | Elasticity matrix (global), 35-component | Domain 2 |  |
| solid.Dg36 | solid.D36 | Pa | Elasticity matrix (global), 36-component | Domain 2 |  |
| solid.Dg44 | solid.D44 | Pa | Elasticity matrix (global), 44-component | Domain 2 |  |
| solid.Dg45 | solid.D45 | Pa | Elasticity matrix (global), 45-component | Domain 2 |  |
| solid.Dg46 | solid.D46 | Pa | Elasticity matrix (global), 46-component | Domain 2 |  |
| solid.Dg55 | solid.D55 | Pa | Elasticity matrix (global), 55-component | Domain 2 |  |
| solid.Dg56 | solid.D56 | Pa | Elasticity matrix (global), 56-component | Domain 2 |  |
| solid.Dg66 | solid.D66 | Pa | Elasticity matrix (global), 66-component | Domain 2 |  |
| solid.DVog11 | solid.DVo11 | Pa | Elasticity matrix, Voigt notation (global), 11-component | Domain 2 |  |
| solid.DVog12 | solid.DVo12 | Pa | Elasticity matrix, Voigt notation (global), 12-component | Domain 2 |  |
| solid.DVog13 | solid.DVo13 | Pa | Elasticity matrix, Voigt notation (global), 13-component | Domain 2 |  |
| solid.DVog14 | solid.DVo14 | Pa | Elasticity matrix, Voigt notation (global), 14-component | Domain 2 |  |
| solid.DVog15 | solid.DVo15 | Pa | Elasticity matrix, Voigt notation (global), 15-component | Domain 2 |  |
| solid.DVog16 | solid.DVo16 | Pa | Elasticity matrix, Voigt notation (global), 16-component | Domain 2 |  |
| solid.DVog22 | solid.DVo22 | Pa | Elasticity matrix, Voigt notation (global), 22-component | Domain 2 |  |
| solid.DVog23 | solid.DVo23 | Pa | Elasticity matrix, Voigt notation (global), 23-component | Domain 2 |  |
| solid.DVog24 | solid.DVo24 | Pa | Elasticity matrix, Voigt notation (global), 24-component | Domain 2 |  |
| solid.DVog25 | solid.DVo25 | Pa | Elasticity matrix, Voigt notation (global), 25-component | Domain 2 |  |
| solid.DVog26 | solid.DVo26 | Pa | Elasticity matrix, Voigt notation (global), 26-component | Domain 2 |  |
| solid.DVog33 | solid.DVo33 | Pa | Elasticity matrix, Voigt notation (global), 33-component | Domain 2 |  |
| solid.DVog34 | solid.DVo34 | Pa | Elasticity matrix, Voigt notation (global), 34-component | Domain 2 |  |
| solid.DVog35 | solid.DVo35 | Pa | Elasticity matrix, Voigt notation (global), 35-component | Domain 2 |  |
| solid.DVog36 | solid.DVo36 | Pa | Elasticity matrix, Voigt notation (global), 36-component | Domain 2 |  |
| solid.DVog44 | solid.DVo44 | Pa | Elasticity matrix, Voigt notation (global), 44-component | Domain 2 |  |
| solid.DVog45 | solid.DVo45 | Pa | Elasticity matrix, Voigt notation (global), 45-component | Domain 2 |  |
| solid.DVog46 | solid.DVo46 | Pa | Elasticity matrix, Voigt notation (global), 46-component | Domain 2 |  |
| solid.DVog55 | solid.DVo55 | Pa | Elasticity matrix, Voigt notation (global), 55-component | Domain 2 |  |
| solid.DVog56 | solid.DVo56 | Pa | Elasticity matrix, Voigt notation (global), 56-component | Domain 2 |  |
| solid.DVog66 | solid.DVo66 | Pa | Elasticity matrix, Voigt notation (global), 66-component | Domain 2 |  |
| solid.Kpar0 | 0 |  | Instantaneous bulk modulus | Domain 2 | + operation |
| solid.Qser0 | 0 | 1/Pa | Instantaneous bulk compliance | Domain 2 | + operation |
| solid.Jser0 | 0 | 1/Pa | Instantaneous shear compliance | Domain 2 | + operation |
| solid.Gpar0 | 0 |  | Instantaneous shear modulus | Domain 2 | + operation |
| solid.K0 | 1/(1/(solid.Kequ+solid.Kpar0)+solid.Qser0) | N/m² | Instantaneous bulk modulus | Domain 2 |  |
| solid.G0 | 1/(1/(solid.Gequ+solid.Gpar0)+solid.Jser0) | N/m² | Instantaneous shear modulus | Domain 2 |  |
| solid.Qh | 0.5*solid.omega*real((imag(solid.D11)*solid.eel11+imag(solid.D12)*solid.eel22+imag(solid.D13)*solid.eel33+2*imag(solid.D14)*solid.eel12+2*imag(solid.D15)*solid.eel23+2*imag(solid.D16)*solid.eel13)*conj(solid.eel11)+2*(imag(solid.D14)*solid.eel11+imag(solid.D24)*solid.eel22+imag(solid.D34)*solid.eel33+2*imag(solid.D44)*solid.eel12+2*imag(solid.D45)*solid.eel23+2*imag(solid.D46)*solid.eel13)*conj(solid.eel12)+2*(imag(solid.D16)*solid.eel11+imag(solid.D26)*solid.eel22+imag(solid.D36)*solid.eel33+2*imag(solid.D46)*solid.eel12+2*imag(solid.D56)*solid.eel23+2*imag(solid.D66)*solid.eel13)*conj(solid.eel13)+(imag(solid.D12)*solid.eel11+imag(solid.D22)*solid.eel22+imag(solid.D23)*solid.eel33+2*imag(solid.D24)*solid.eel12+2*imag(solid.D25)*solid.eel23+2*imag(solid.D26)*solid.eel13)*conj(solid.eel22)+2*(imag(solid.D15)*solid.eel11+imag(solid.D25)*solid.eel22+imag(solid.D35)*solid.eel33+2*imag(solid.D45)*solid.eel12+2*imag(solid.D55)*solid.eel23+2*imag(solid.D56)*solid.eel13)*conj(solid.eel23)+(imag(solid.D13)*solid.eel11+imag(solid.D23)*solid.eel22+imag(solid.D33)*solid.eel33+2*imag(solid.D34)*solid.eel12+2*imag(solid.D35)*solid.eel23+2*imag(solid.D36)*solid.eel13)*conj(solid.eel33))*!isPML | W/m³ | Total power dissipation density | Domain 2 | + operation |
| solid.rho | material.rho | kg/m³ | Density | Domain 2 | Meta, * operation |
| solid.cp | sqrt((solid.K+4*solid.G/3)/solid.rho) | m/s | Equivalent speed of pressure wave | Domain 2 |  |
| solid.cs | sqrt(solid.G/solid.rho) | m/s | Equivalent speed of shear wave | Domain 2 |  |
| solid.Cgl11 | solid.D11+solid.D12+solid.D13 | Pa | C:g tensor, local coordinate system, 11-component | Domain 2 |  |
| solid.Cgl12 | solid.D14+solid.D24+solid.D34 | Pa | C:g tensor, local coordinate system, 12-component | Domain 2 |  |
| solid.Cgl13 | solid.D16+solid.D26+solid.D36 | Pa | C:g tensor, local coordinate system, 13-component | Domain 2 |  |
| solid.Cgl22 | solid.D12+solid.D22+solid.D23 | Pa | C:g tensor, local coordinate system, 22-component | Domain 2 |  |
| solid.Cgl23 | solid.D15+solid.D25+solid.D35 | Pa | C:g tensor, local coordinate system, 23-component | Domain 2 |  |
| solid.Cgl33 | solid.D13+solid.D23+solid.D33 | Pa | C:g tensor, local coordinate system, 33-component | Domain 2 |  |
| solid.elogxx | log(solid.stchp1)*solid.LW11^2+log(solid.stchp2)*solid.LW12^2+log(solid.stchp3)*solid.LW13^2 | 1 | Logarithmic strain tensor, xx-component | Domain 2 |  |
| solid.elogxy | log(solid.stchp1)*solid.LW11*solid.LW21+log(solid.stchp2)*solid.LW12*solid.LW22+log(solid.stchp3)*solid.LW13*solid.LW23 | 1 | Logarithmic strain tensor, xy-component | Domain 2 |  |
| solid.elogxz | log(solid.stchp1)*solid.LW11*solid.LW31+log(solid.stchp2)*solid.LW12*solid.LW32+log(solid.stchp3)*solid.LW13*solid.LW33 | 1 | Logarithmic strain tensor, xz-component | Domain 2 |  |
| solid.elogyy | log(solid.stchp1)*solid.LW21^2+log(solid.stchp2)*solid.LW22^2+log(solid.stchp3)*solid.LW23^2 | 1 | Logarithmic strain tensor, yy-component | Domain 2 |  |
| solid.elogyz | log(solid.stchp1)*solid.LW21*solid.LW31+log(solid.stchp2)*solid.LW22*solid.LW32+log(solid.stchp3)*solid.LW23*solid.LW33 | 1 | Logarithmic strain tensor, yz-component | Domain 2 |  |
| solid.elogzz | log(solid.stchp1)*solid.LW31^2+log(solid.stchp2)*solid.LW32^2+log(solid.stchp3)*solid.LW33^2 | 1 | Logarithmic strain tensor, zz-component | Domain 2 |  |
| solid.ellog11 | (solid.sysT11*solid.elogxx+solid.sysT12*solid.elogxy+solid.sysT13*solid.elogxz)*solid.sysT11+(solid.sysT11*solid.elogxy+solid.sysT12*solid.elogyy+solid.sysT13*solid.elogyz)*solid.sysT12+(solid.sysT11*solid.elogxz+solid.sysT12*solid.elogyz+solid.sysT13*solid.elogzz)*solid.sysT13 | 1 | Logarithmic strain tensor, local coordinate system, 11-component | Domain 2 |  |
| solid.ellog12 | (solid.sysT11*solid.elogxx+solid.sysT12*solid.elogxy+solid.sysT13*solid.elogxz)*solid.sysT21+(solid.sysT11*solid.elogxy+solid.sysT12*solid.elogyy+solid.sysT13*solid.elogyz)*solid.sysT22+(solid.sysT11*solid.elogxz+solid.sysT12*solid.elogyz+solid.sysT13*solid.elogzz)*solid.sysT23 | 1 | Logarithmic strain tensor, local coordinate system, 12-component | Domain 2 |  |
| solid.ellog13 | (solid.sysT11*solid.elogxx+solid.sysT12*solid.elogxy+solid.sysT13*solid.elogxz)*solid.sysT31+(solid.sysT11*solid.elogxy+solid.sysT12*solid.elogyy+solid.sysT13*solid.elogyz)*solid.sysT32+(solid.sysT11*solid.elogxz+solid.sysT12*solid.elogyz+solid.sysT13*solid.elogzz)*solid.sysT33 | 1 | Logarithmic strain tensor, local coordinate system, 13-component | Domain 2 |  |
| solid.ellog22 | (solid.sysT21*solid.elogxx+solid.sysT22*solid.elogxy+solid.sysT23*solid.elogxz)*solid.sysT21+(solid.sysT21*solid.elogxy+solid.sysT22*solid.elogyy+solid.sysT23*solid.elogyz)*solid.sysT22+(solid.sysT21*solid.elogxz+solid.sysT22*solid.elogyz+solid.sysT23*solid.elogzz)*solid.sysT23 | 1 | Logarithmic strain tensor, local coordinate system, 22-component | Domain 2 |  |
| solid.ellog23 | (solid.sysT21*solid.elogxx+solid.sysT22*solid.elogxy+solid.sysT23*solid.elogxz)*solid.sysT31+(solid.sysT21*solid.elogxy+solid.sysT22*solid.elogyy+solid.sysT23*solid.elogyz)*solid.sysT32+(solid.sysT21*solid.elogxz+solid.sysT22*solid.elogyz+solid.sysT23*solid.elogzz)*solid.sysT33 | 1 | Logarithmic strain tensor, local coordinate system, 23-component | Domain 2 |  |
| solid.ellog33 | (solid.sysT31*solid.elogxx+solid.sysT32*solid.elogxy+solid.sysT33*solid.elogxz)*solid.sysT31+(solid.sysT31*solid.elogxy+solid.sysT32*solid.elogyy+solid.sysT33*solid.elogyz)*solid.sysT32+(solid.sysT31*solid.elogxz+solid.sysT32*solid.elogyz+solid.sysT33*solid.elogzz)*solid.sysT33 | 1 | Logarithmic strain tensor, local coordinate system, 33-component | Domain 2 |  |
| solid.disp_rv | sqrt(u^2+v^2+eps) | m | Displacement magnitude | Domain 2 |  |
| solid.vel_rv | 2*solid.disp_rv*pi*solid.freq | m/s | Velocity magnitude | Domain 2 |  |
| solid.utt_rv | solid.disp_rv*(2*pi*solid.freq)^2 | m/s² | Acceleration magnitude | Domain 2 |  |
| solid.mises_rv | sqrt(3*solid.II2s+eps) | N/m² | von Mises stress | Domain 2 |  |
| solid.u_tt_rvX | -(2*pi*solid.freq)^2*u | m/s² | Acceleration, X-component | Domain 2 |  |
| solid.u_tt_rvY | -(2*pi*solid.freq)^2*v | m/s² | Acceleration, Y-component | Domain 2 |  |
| solid.u_tt_rvZ | 0 | m/s² | Acceleration, Z-component | Domain 2 |  |
| solid.u_ttX | material.dt(material.dt(u)) | m/s² | Acceleration, X-component | Domain 2 |  |
| solid.u_ttY | material.dt(material.dt(v)) | m/s² | Acceleration, Y-component | Domain 2 |  |
| solid.u_ttZ | material.dt(material.dt(0)) | m/s² | Acceleration, Z-component | Domain 2 |  |
| solid.u_tX | material.dt(u) | m/s | Velocity, X-component | Domain 2 |  |
| solid.u_tY | material.dt(v) | m/s | Velocity, Y-component | Domain 2 |  |
| solid.u_tZ | material.dt(0) | m/s | Velocity, Z-component | Domain 2 |  |
| solid.vel_rms | sqrt(0.5*(realdot(solid.u_tX,solid.u_tX)+realdot(solid.u_tY,solid.u_tY)+realdot(solid.u_tZ,solid.u_tZ))) | m/s | Velocity magnitude, RMS | Domain 2 |  |
| solid.acc_rms | sqrt(0.5*(realdot(solid.u_ttX,solid.u_ttX)+realdot(solid.u_ttY,solid.u_ttY)+realdot(solid.u_ttZ,solid.u_ttZ))) | m/s² | Acceleration magnitude, RMS | Domain 2 |  |
| solid.uAmpX | abs(u) | m | Displacement amplitude, X-component | Domain 2 |  |
| solid.uAmpY | abs(v) | m | Displacement amplitude, Y-component | Domain 2 |  |
| solid.uAmpZ | 0 | m | Displacement amplitude, Z-component | Domain 2 |  |
| solid.uAmp_tX | solid.uAmpX*abs(solid.omega) | m/s | Velocity amplitude, X-component | Domain 2 |  |
| solid.uAmp_tY | solid.uAmpY*abs(solid.omega) | m/s | Velocity amplitude, Y-component | Domain 2 |  |
| solid.uAmp_tZ | solid.uAmpZ*abs(solid.omega) | m/s | Velocity amplitude, Z-component | Domain 2 |  |
| solid.uAmp_ttX | solid.uAmpX*solid.omega^2 | m/s² | Acceleration amplitude, X-component | Domain 2 |  |
| solid.uAmp_ttY | solid.uAmpY*solid.omega^2 | m/s² | Acceleration amplitude, Y-component | Domain 2 |  |
| solid.uAmp_ttZ | solid.uAmpZ*solid.omega^2 | m/s² | Acceleration amplitude, Z-component | Domain 2 |  |
| solid.uPhaseX | mod(arg(u),2*pi) | rad | Displacement phase, X-component | Domain 2 |  |
| solid.uPhaseY | mod(arg(v),2*pi) | rad | Displacement phase, Y-component | Domain 2 |  |
| solid.uPhaseZ | mod(arg(0),2*pi) | rad | Displacement phase, Z-component | Domain 2 |  |
| solid.uPhase_tX | mod(0.5*pi+solid.uPhaseX,2*pi) | rad | Velocity phase, X-component | Domain 2 |  |
| solid.uPhase_tY | mod(0.5*pi+solid.uPhaseY,2*pi) | rad | Velocity phase, Y-component | Domain 2 |  |
| solid.uPhase_tZ | mod(0.5*pi+solid.uPhaseZ,2*pi) | rad | Velocity phase, Z-component | Domain 2 |  |
| solid.uPhase_ttX | mod(pi+solid.uPhaseX,2*pi) | rad | Acceleration phase, X-component | Domain 2 |  |
| solid.uPhase_ttY | mod(pi+solid.uPhaseY,2*pi) | rad | Acceleration phase, Y-component | Domain 2 |  |
| solid.uPhase_ttZ | mod(pi+solid.uPhaseZ,2*pi) | rad | Acceleration phase, Z-component | Domain 2 |  |
| solid.disp_peak | sqrteps(0.5*(solid.uAmpX^2+solid.uAmpY^2+solid.uAmpZ^2+sqrteps(solid.uAmpX^2*(solid.uAmpX^2+cos(2*(solid.uPhaseX-solid.uPhaseY))*solid.uAmpY^2+cos(2*(solid.uPhaseX-solid.uPhaseZ))*solid.uAmpZ^2)+solid.uAmpY^2*(cos(2*(solid.uPhaseX-solid.uPhaseY))*solid.uAmpX^2+solid.uAmpY^2+cos(2*(solid.uPhaseY-solid.uPhaseZ))*solid.uAmpZ^2)+solid.uAmpZ^2*(cos(2*(solid.uPhaseX-solid.uPhaseZ))*solid.uAmpX^2+cos(2*(solid.uPhaseY-solid.uPhaseZ))*solid.uAmpY^2+solid.uAmpZ^2)))) | m | Displacement magnitude, peak | Domain 2 |  |
| solid.vel_peak | sqrteps(0.5*(solid.uAmp_tX^2+solid.uAmp_tY^2+solid.uAmp_tZ^2+sqrteps(solid.uAmp_tX^2*(solid.uAmp_tX^2+cos(2*(solid.uPhase_tX-solid.uPhase_tY))*solid.uAmp_tY^2+cos(2*(solid.uPhase_tX-solid.uPhase_tZ))*solid.uAmp_tZ^2)+solid.uAmp_tY^2*(cos(2*(solid.uPhase_tX-solid.uPhase_tY))*solid.uAmp_tX^2+solid.uAmp_tY^2+cos(2*(solid.uPhase_tY-solid.uPhase_tZ))*solid.uAmp_tZ^2)+solid.uAmp_tZ^2*(cos(2*(solid.uPhase_tX-solid.uPhase_tZ))*solid.uAmp_tX^2+cos(2*(solid.uPhase_tY-solid.uPhase_tZ))*solid.uAmp_tY^2+solid.uAmp_tZ^2)))) | m/s | Velocity magnitude, peak | Domain 2 |  |
| solid.u_tt_peak | sqrteps(0.5*(solid.uAmp_ttX^2+solid.uAmp_ttY^2+solid.uAmp_ttZ^2+sqrteps(solid.uAmp_ttX^2*(solid.uAmp_ttX^2+cos(2*(solid.uPhase_ttX-solid.uPhase_ttY))*solid.uAmp_ttY^2+cos(2*(solid.uPhase_ttX-solid.uPhase_ttZ))*solid.uAmp_ttZ^2)+solid.uAmp_ttY^2*(cos(2*(solid.uPhase_ttX-solid.uPhase_ttY))*solid.uAmp_ttX^2+solid.uAmp_ttY^2+cos(2*(solid.uPhase_ttY-solid.uPhase_ttZ))*solid.uAmp_ttZ^2)+solid.uAmp_ttZ^2*(cos(2*(solid.uPhase_ttX-solid.uPhase_ttZ))*solid.uAmp_ttX^2+cos(2*(solid.uPhase_ttY-solid.uPhase_ttZ))*solid.uAmp_ttY^2+solid.uAmp_ttZ^2)))) | m/s² | Acceleration magnitude, peak | Domain 2 |  |
| solid.afX | 0 | m/s² | Frame acceleration, X-component | Domain 2 | + operation |
| solid.afY | 0 | m/s² | Frame acceleration, Y-component | Domain 2 | + operation |
| solid.afZ | 0 | m/s² | Frame acceleration, Z-component | Domain 2 | + operation |
| solid.accX | solid.u_ttX | m/s² | Effective acceleration, X-component | Domain 2 | + operation |
| solid.accY | solid.u_ttY | m/s² | Effective acceleration, Y-component | Domain 2 | + operation |
| solid.accZ | solid.u_ttZ | m/s² | Effective acceleration, Z-component | Domain 2 | + operation |
| solid.vel | sqrteps(real(solid.u_tX)^2+real(solid.u_tY)^2+real(solid.u_tZ)^2) | m/s | Velocity magnitude | Domain 2 |  |
| solid.acc | sqrteps(real(solid.accX)^2+real(solid.accY)^2+real(solid.accZ)^2) | m/s² | Effective acceleration magnitude | Domain 2 |  |
| solid.u_tt | sqrteps(real(solid.u_ttX)^2+real(solid.u_ttY)^2+real(solid.u_ttZ)^2) | m/s² | Acceleration magnitude | Domain 2 |  |
| solid.Tax | mean(solid.sxx)*solid.nx+mean(solid.sxy)*solid.ny+mean(solid.sxz)*solid.nz | N/m² | Traction (force/area), x-component | Boundaries 4–8, 11–12, 14, 16–17, 19–20, 22–24, 26, 29–32 |  |
| solid.Tay | mean(solid.sxy)*solid.nx+mean(solid.syy)*solid.ny+mean(solid.syz)*solid.nz | N/m² | Traction (force/area), y-component | Boundaries 4–8, 11–12, 14, 16–17, 19–20, 22–24, 26, 29–32 |  |
| solid.Taz | mean(solid.sxz)*solid.nx+mean(solid.syz)*solid.ny+mean(solid.szz)*solid.nz | N/m² | Traction (force/area), z-component | Boundaries 4–8, 11–12, 14, 16–17, 19–20, 22–24, 26, 29–32 |  |
| solid.stn | solid.Tax*solid.nx+solid.Tay*solid.ny+solid.Taz*solid.nz | N/m² | Normal stress | Boundaries 4–8, 11–12, 14, 16–17, 19–20, 22–24, 26, 29–32 |  |
| solid.Tanx | solid.stn*solid.nx | N/m² | Normal component of traction, x-component | Boundaries 4–8, 11–12, 14, 16–17, 19–20, 22–24, 26, 29–32 |  |
| solid.Tany | solid.stn*solid.ny | N/m² | Normal component of traction, y-component | Boundaries 4–8, 11–12, 14, 16–17, 19–20, 22–24, 26, 29–32 |  |
| solid.Tanz | solid.stn*solid.nz | N/m² | Normal component of traction, z-component | Boundaries 4–8, 11–12, 14, 16–17, 19–20, 22–24, 26, 29–32 |  |
| solid.Tatx | solid.Tax-solid.Tanx | N/m² | Shear component of traction, x-component | Boundaries 4–8, 11–12, 14, 16–17, 19–20, 22–24, 26, 29–32 |  |
| solid.Taty | solid.Tay-solid.Tany | N/m² | Shear component of traction, y-component | Boundaries 4–8, 11–12, 14, 16–17, 19–20, 22–24, 26, 29–32 |  |
| solid.Tatz | solid.Taz-solid.Tanz | N/m² | Shear component of traction, z-component | Boundaries 4–8, 11–12, 14, 16–17, 19–20, 22–24, 26, 29–32 |  |
| solid.stt | sqrt(solid.Tatx^2+solid.Taty^2+solid.Tatz^2) | N/m² | Shear stress | Boundaries 4–8, 11–12, 14, 16–17, 19–20, 22–24, 26, 29–32 |  |
| solid.sysT11 | 1 | 1 | Transform to global system, 11-component | Domain 2 |  |
| solid.sysT21 | 0 | 1 | Transform to global system, 21-component | Domain 2 |  |
| solid.sysT31 | 0 | 1 | Transform to global system, 31-component | Domain 2 |  |
| solid.sysT12 | 0 | 1 | Transform to global system, 12-component | Domain 2 |  |
| solid.sysT22 | 1 | 1 | Transform to global system, 22-component | Domain 2 |  |
| solid.sysT32 | 0 | 1 | Transform to global system, 32-component | Domain 2 |  |
| solid.sysT13 | 0 | 1 | Transform to global system, 13-component | Domain 2 |  |
| solid.sysT23 | 0 | 1 | Transform to global system, 23-component | Domain 2 |  |
| solid.sysT33 | 1 | 1 | Transform to global system, 33-component | Domain 2 |  |
| uTXt | d(uTX,TIME) | 1/s | Tangential gradient of u, X component, first time derivative | Boundaries 4–8, 11–12, 14, 16–17, 19–20, 22–24, 26, 29–32 |  |
| uTYt | d(uTY,TIME) | 1/s | Tangential gradient of u, Y component, first time derivative | Boundaries 4–8, 11–12, 14, 16–17, 19–20, 22–24, 26, 29–32 |  |
| uTXtt | d(d(uTX,TIME),TIME) | 1/s² | Tangential gradient of u, X component, second time derivative | Boundaries 4–8, 11–12, 14, 16–17, 19–20, 22–24, 26, 29–32 |  |
| uTYtt | d(d(uTY,TIME),TIME) | 1/s² | Tangential gradient of u, Y component, second time derivative | Boundaries 4–8, 11–12, 14, 16–17, 19–20, 22–24, 26, 29–32 |  |
| uXt | d(uX,TIME) | 1/s | Gradient of u, X component, first time derivative | Domain 2 |  |
| uYt | d(uY,TIME) | 1/s | Gradient of u, Y component, first time derivative | Domain 2 |  |
| uXtt | d(d(uX,TIME),TIME) | 1/s² | Gradient of u, X component, second time derivative | Domain 2 |  |
| uYtt | d(d(uY,TIME),TIME) | 1/s² | Gradient of u, Y component, second time derivative | Domain 2 |  |
| vTXt | d(vTX,TIME) | 1/s | Tangential gradient of v, X component, first time derivative | Boundaries 4–8, 11–12, 14, 16–17, 19–20, 22–24, 26, 29–32 |  |
| vTYt | d(vTY,TIME) | 1/s | Tangential gradient of v, Y component, first time derivative | Boundaries 4–8, 11–12, 14, 16–17, 19–20, 22–24, 26, 29–32 |  |
| vTXtt | d(d(vTX,TIME),TIME) | 1/s² | Tangential gradient of v, X component, second time derivative | Boundaries 4–8, 11–12, 14, 16–17, 19–20, 22–24, 26, 29–32 |  |
| vTYtt | d(d(vTY,TIME),TIME) | 1/s² | Tangential gradient of v, Y component, second time derivative | Boundaries 4–8, 11–12, 14, 16–17, 19–20, 22–24, 26, 29–32 |  |
| vXt | d(vX,TIME) | 1/s | Gradient of v, X component, first time derivative | Domain 2 |  |
| vYt | d(vY,TIME) | 1/s | Gradient of v, Y component, first time derivative | Domain 2 |  |
| vXtt | d(d(vX,TIME),TIME) | 1/s² | Gradient of v, X component, second time derivative | Domain 2 |  |
| vYtt | d(d(vY,TIME),TIME) | 1/s² | Gradient of v, Y component, second time derivative | Domain 2 |  |
| ut | d(u,TIME) | m/s | Structural velocity field, X-component | Domain 2 |  |
| vt | d(v,TIME) | m/s | Structural velocity field, Y-component | Domain 2 |  |
| utt | d(d(u,TIME),TIME) | m/s² | Acceleration field, X-component | Domain 2 |  |
| vtt | d(d(v,TIME),TIME) | m/s² | Acceleration field, Y-component | Domain 2 |  |
| solid.Ldx | solid.gradUxX*solid.iomega | 1/s | Rate of strain tensor, x-component | Domain 2 |  |
| solid.Ldxy | 0.5*solid.iomega*(solid.gradUxY+solid.gradUyX) | 1/s | Rate of strain tensor, xy-component | Domain 2 |  |
| solid.Ldxz | 0.5*solid.iomega*(solid.gradUxZ+solid.gradUzX) | 1/s | Rate of strain tensor, xz-component | Domain 2 |  |
| solid.Ldy | solid.gradUyY*solid.iomega | 1/s | Rate of strain tensor, y-component | Domain 2 |  |
| solid.Ldyz | 0.5*solid.iomega*(solid.gradUyZ+solid.gradUzY) | 1/s | Rate of strain tensor, yz-component | Domain 2 |  |
| solid.Ldz | solid.gradUzZ*solid.iomega | 1/s | Rate of strain tensor, z-component | Domain 2 |  |
| solid.Lwx | 0 | 1/s | Spin tensor, x-component | Domain 2 |  |
| solid.Lwxy | 0.5*solid.iomega*(solid.gradUxY-solid.gradUyX) | 1/s | Spin tensor, xy-component | Domain 2 |  |
| solid.Lwxz | 0.5*solid.iomega*(solid.gradUxZ-solid.gradUzX) | 1/s | Spin tensor, xz-component | Domain 2 |  |
| solid.Lwy | 0 | 1/s | Spin tensor, y-component | Domain 2 |  |
| solid.Lwyz | 0.5*solid.iomega*(solid.gradUyZ-solid.gradUzY) | 1/s | Spin tensor, yz-component | Domain 2 |  |
| solid.Lwz | 0 | 1/s | Spin tensor, z-component | Domain 2 |  |
| solid.sp1Gp | solid.gpeval(solid.sp1) | N/m² | First principal stress | Domain 2 |  |
| solid.sp2Gp | solid.gpeval(solid.sp2) | N/m² | Second principal stress | Domain 2 |  |
| solid.sp3Gp | solid.gpeval(solid.sp3) | N/m² | Third principal stress | Domain 2 |  |
| solid.RFx | reacf(u) | N | Reaction force, x-component | Domain 2 |  |
| solid.RFy | reacf(v) | N | Reaction force, y-component | Domain 2 |  |
| solid.RFz | 0 | N | Reaction force, z-component | Domain 2 |  |
| solid.RMx | solid.RFz*(y-solid.refpnty)+solid.RFy*solid.refpntz | N·m | Reaction moment, x-component | Domain 2 |  |
| solid.RMy | -solid.RFz*(x-solid.refpntx)-solid.RFx*solid.refpntz | N·m | Reaction moment, y-component | Domain 2 |  |
| solid.RMz | solid.RFy*(x-solid.refpntx)-solid.RFx*(y-solid.refpnty) | N·m | Reaction moment, z-component | Domain 2 |  |
| solid.Qh_tot | solid.lemm1.int10(solid.Qh*solid.d) | W | Total power dissipation | Global | + operation |
| solid.IX | 0.5*real(-solid.SX*conj(solid.u_tX)-solid.SXY*conj(solid.u_tY)-solid.SXZ*conj(solid.u_tZ)) | W/m² | Mechanical energy flux, X-component | Domain 2 |  |
| solid.IY | 0.5*real(-solid.SXY*conj(solid.u_tX)-solid.SY*conj(solid.u_tY)-solid.SYZ*conj(solid.u_tZ)) | W/m² | Mechanical energy flux, Y-component | Domain 2 |  |
| solid.IZ | 0.5*real(-solid.SXZ*conj(solid.u_tX)-solid.SYZ*conj(solid.u_tY)-solid.SZ*conj(solid.u_tZ)) | W/m² | Mechanical energy flux, Z-component | Domain 2 |  |
| solid.IcomplexX | -solid.SX*conj(solid.u_tX)-solid.SXY*conj(solid.u_tY)-solid.SXZ*conj(solid.u_tZ) | W/m² | Complex mechanical energy flux, X-component | Domain 2 |  |
| solid.IcomplexY | -solid.SXY*conj(solid.u_tX)-solid.SY*conj(solid.u_tY)-solid.SYZ*conj(solid.u_tZ) | W/m² | Complex mechanical energy flux, Y-component | Domain 2 |  |
| solid.IcomplexZ | -solid.SXZ*conj(solid.u_tX)-solid.SYZ*conj(solid.u_tY)-solid.SZ*conj(solid.u_tZ) | W/m² | Complex mechanical energy flux, Z-component | Domain 2 |  |
| solid.nI | nX*solid.IX+nY*solid.IY | W/m² | Outward mechanical energy flux | Boundaries 4–5, 7–8, 12, 16–17, 19–20, 22–23, 26, 29–32 | Meta |
| solid.nIcomplex | nX*solid.IcomplexX+nY*solid.IcomplexY | W/m² | Outward complex mechanical energy flux | Boundaries 4–5, 7–8, 12, 16–17, 19–20, 22–23, 26, 29–32 | Meta |
| solid.lemm1.integrand | 1 |  | Intermediate variable | Domain 2 | Meta |
| solid.lemm1.gsv | solid.d | m | Geometry scale factor (volume) | Domain 2 |  |
| solid.E | material.E | Pa | Young's modulus | Domain 2 | Meta, * operation |
| solid.nu | material.nu | 1 | Poisson's ratio | Domain 2 | Meta |
| solid.G | 0.5*solid.E/(1+solid.nu) | N/m² | Shear modulus | Domain 2 | * operation |
| solid.lambLame | solid.E*solid.nu/((1+solid.nu)*(1-2*solid.nu)) | N/m² | Lamé parameter λ | Domain 2 | * operation |
| solid.muLame | 0.5*solid.E/(1+solid.nu) | N/m² | Lamé parameter μ | Domain 2 | * operation |
| solid.lemm1.nI | nX*solid.IX+nY*solid.IY | W/m² | Outward mechanical energy flux | Boundaries 4–8, 11–12, 14, 16–17, 19–20, 22–24, 26, 29–32 | Meta |
| solid.lemm1.nIcomplex | nX*solid.IcomplexX+nY*solid.IcomplexY | W/m² | Outward complex mechanical energy flux | Boundaries 4–8, 11–12, 14, 16–17, 19–20, 22–24, 26, 29–32 | Meta |

#### Shape functions

| **Name** | **Shape function** | **Unit** | **Description** | **Shape frame** | **Selection** |
| --- | --- | --- | --- | --- | --- |
| u | Nodal serendipity (Quadratic) | m | Displacement field, X-component | Material | Domain 2 |
| v | Nodal serendipity (Quadratic) | m | Displacement field, Y-component | Material | Domain 2 |

#### Weak Expressions

| **Weak expression** | **Integration order** | **Integration frame** | **Selection** |
| --- | --- | --- | --- |
| (-solid.Sl11*test(solid.el11)-2*solid.Sl12*test(solid.el12)-2*solid.Sl13*test(solid.el13)-solid.Sl22*test(solid.el22)-2*solid.Sl23*test(solid.el23)-solid.Sl33*test(solid.el33))*solid.d | 4 | Material | Domain 2 |
| -solid.rho*solid.iomega^2*(u*test(u)+v*test(v))*solid.d | 4 | Material | Domain 2 |

### Free 1


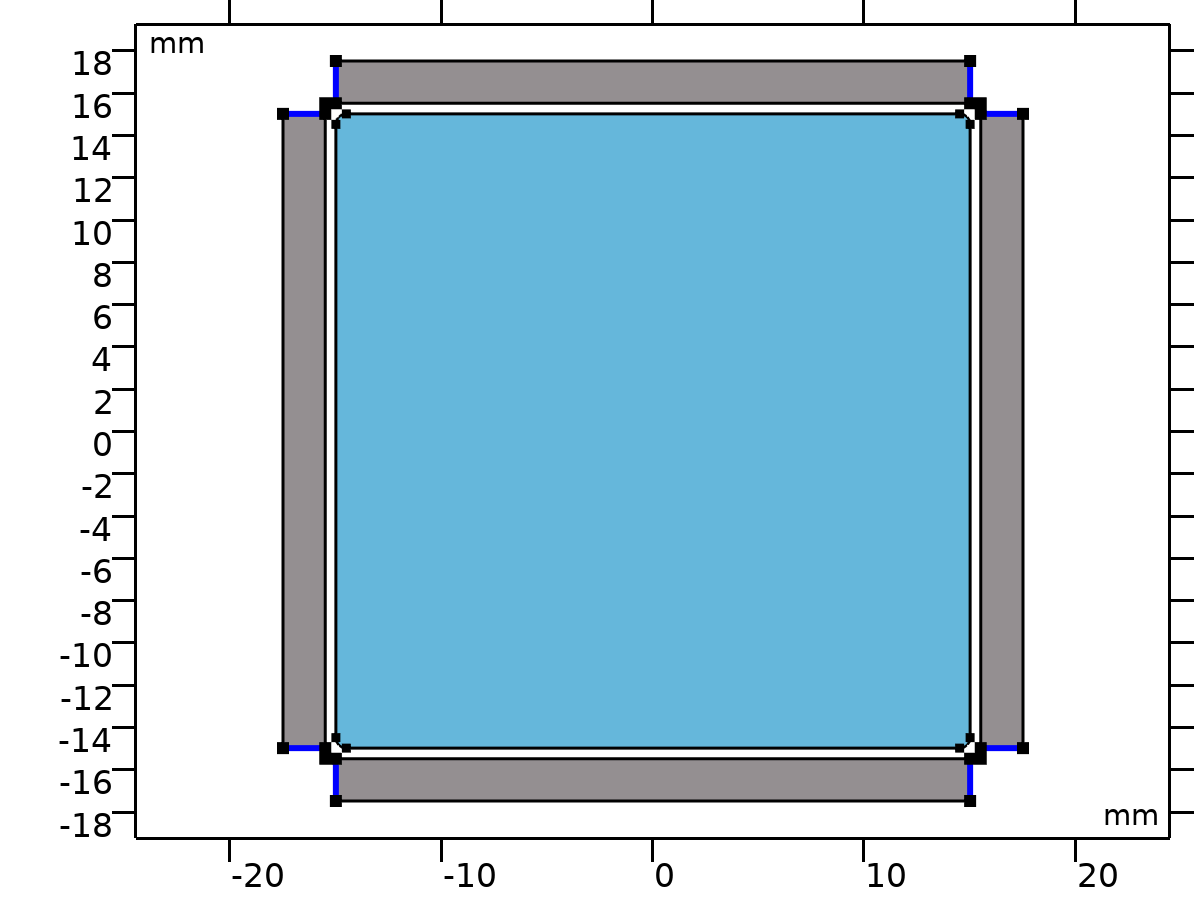


Free 1

Selection

| Geometric entity level | Boundary |
| --- | --- |
| Selection | Geometry geom1: Dimension 1: All boundaries |

Used products

| COMSOL Multiphysics |
| --- |

Properties from material

| **Property** | **Material** | **Property group** |
| --- | --- | --- |
| Density | Polystyrene (PS) [solid] | Basic |

### Initial Values 1


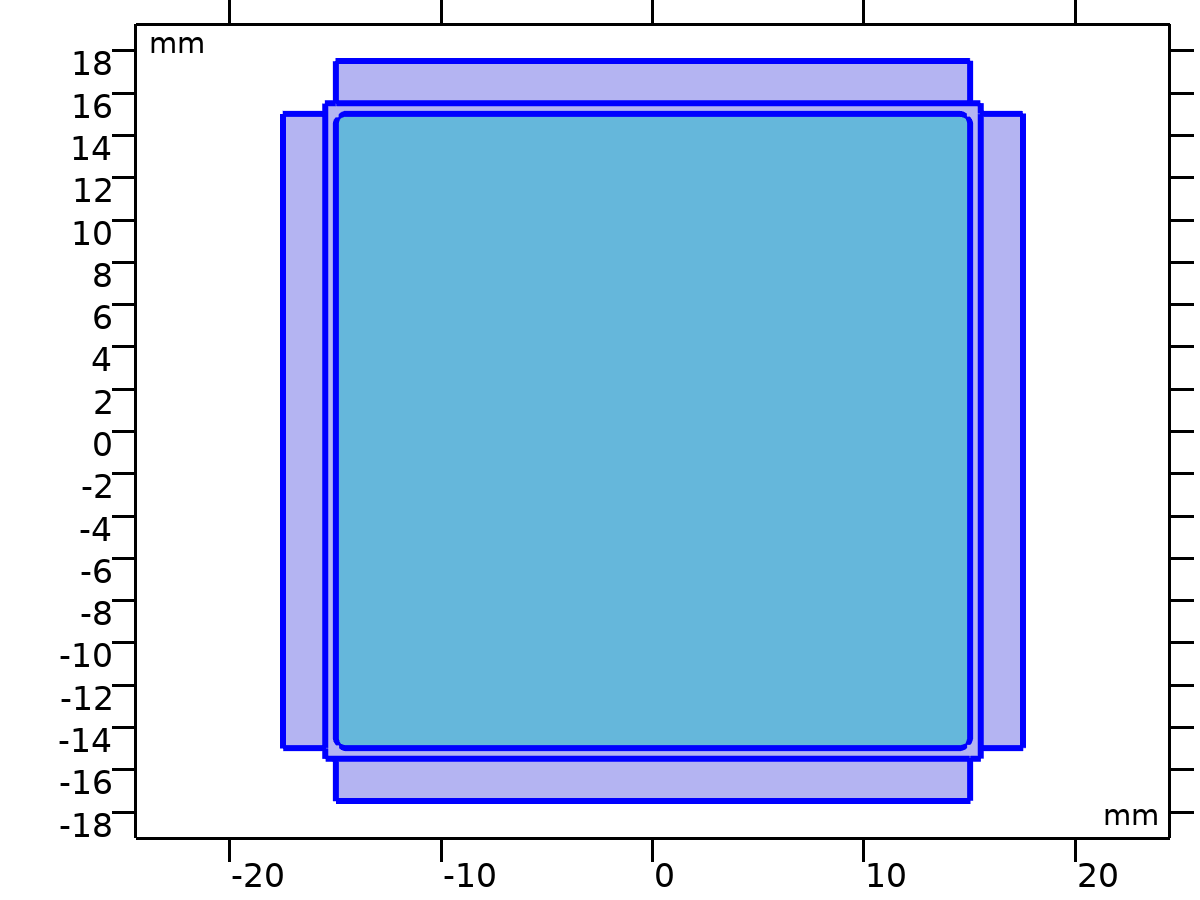


Initial Values 1

Selection

| Geometric entity level | Domain |
| --- | --- |
| Selection | Geometry geom1: Dimension 2: All domains |

#### Initial Values

Settings

| **Description** | **Value** | **Unit** |
| --- | --- | --- |
| Displacement field, X-component | 0 | m |
| Displacement field, Y-component | 0 | m |
| Displacement field, Z-component | 0 | m |
| Structural velocity field, X-component | 0 | m/s |
| Structural velocity field, Y-component | 0 | m/s |
| Structural velocity field, Z-component | 0 | m/s |

#### Coordinate System Selection

Settings

| **Description** | **Value** |
| --- | --- |
| Coordinate system | Global coordinate system |

Used products

| COMSOL Multiphysics |
| --- |

Properties from material

| **Property** | **Material** | **Property group** |
| --- | --- | --- |
| Density | Polystyrene (PS) [solid] | Basic |

#### Variables

| **Name** | **Expression** | **Unit** | **Description** | **Selection** |
| --- | --- | --- | --- | --- |
| solid.init1.u1 | 0 | m | Initial value of displacement, 1-component | Domains 1–3, 5–6 |
| solid.init1.u2 | 0 | m | Initial value of displacement, 2-component | Domains 1–3, 5–6 |
| solid.init1.u3 | 0 | m | Initial value of displacement, 3-component | Domains 1–3, 5–6 |
| solid.init1.ut1 | 0 | m/s | Initial value of structural velocity, 1-component | Domains 1–3, 5–6 |
| solid.init1.ut2 | 0 | m/s | Initial value of structural velocity, 2-component | Domains 1–3, 5–6 |
| solid.init1.ut3 | 0 | m/s | Initial value of structural velocity, 3-component | Domains 1–3, 5–6 |
| solid.uInitx | solid.init1.u1 | m | Initial value of displacement, x-component | Domains 1–3, 5–6 |
| solid.uInity | solid.init1.u2 | m | Initial value of displacement, y-component | Domains 1–3, 5–6 |
| solid.uInitz | solid.init1.u3 | m | Initial value of displacement, z-component | Domains 1–3, 5–6 |
| solid.utInitx | solid.init1.ut1 | m/s | Initial value of structural velocity, x-component | Domains 1–3, 5–6 |
| solid.utInity | solid.init1.ut2 | m/s | Initial value of structural velocity, y-component | Domains 1–3, 5–6 |
| solid.utInitz | solid.init1.ut3 | m/s | Initial value of structural velocity, z-component | Domains 1–3, 5–6 |

### Piezoelectric Material top


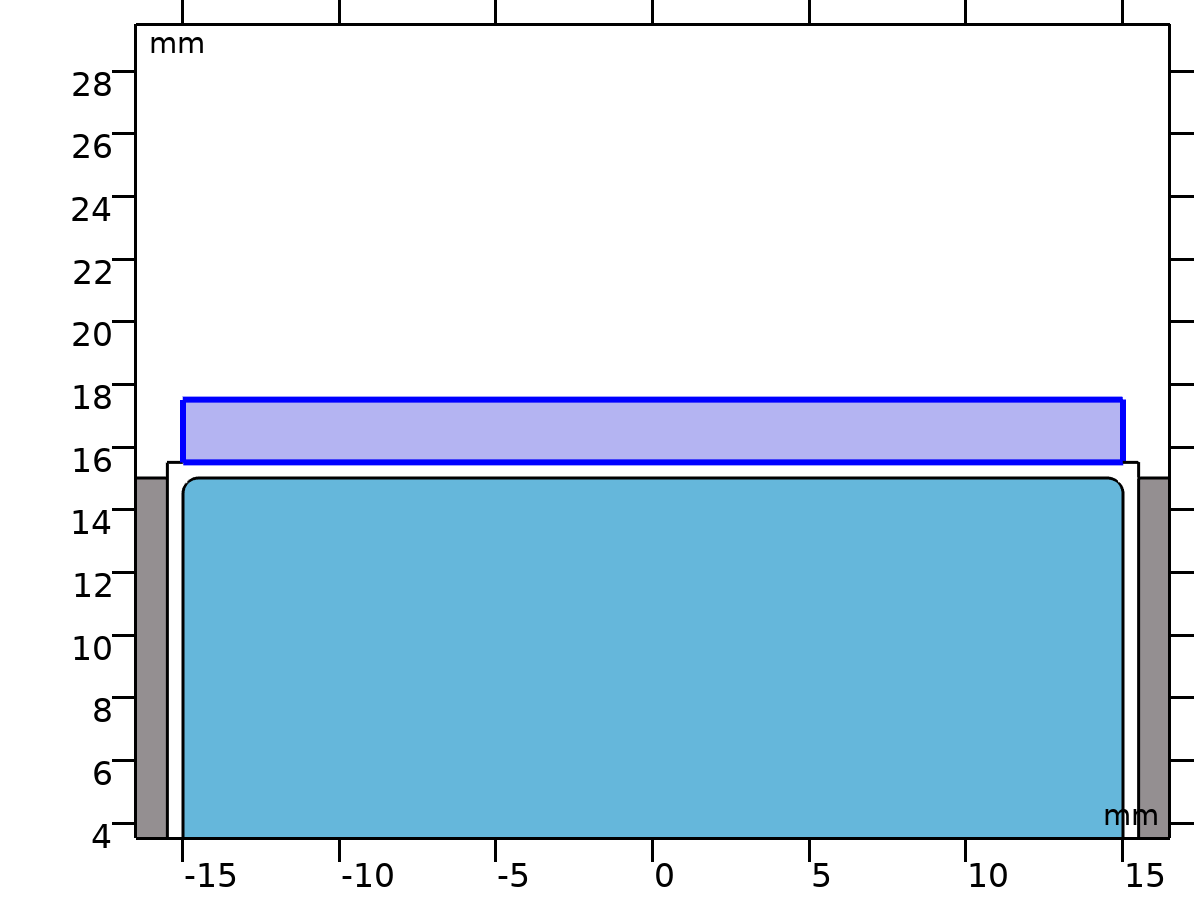


Piezoelectric Material top

Selection

| Geometric entity level | Domain |
| --- | --- |
| Selection | Geometry geom1: Dimension 2: Domain 5 |

Equations


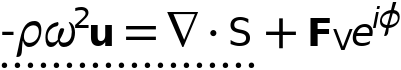


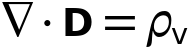


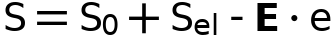


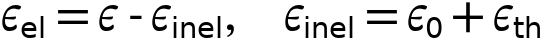


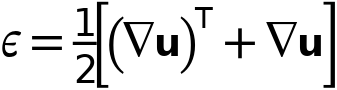


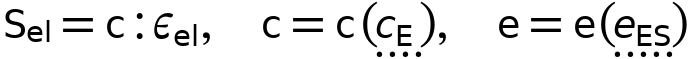


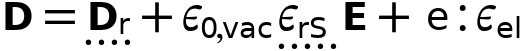


#### Piezoelectric Material Properties

Settings

| **Description** | **Value** | **Unit** |
| --- | --- | --- |
| Constitutive relation | Stress - charge form |  |
| Use multiplicative formulation | Off |  |
| Elasticity matrix, Voigt notation | From material |  |
| Coupling matrix, Voigt notation | From material |  |
| Relative permittivity | From material |  |
| Use mixed formulation | None |  |
| Remanent electric displacement, x1-component | 0 | C/m² |
| Remanent electric displacement, x2-component | 0 | C/m² |
| Remanent electric displacement, x3-component | 0 | C/m² |
| Density | From material |  |

#### Geometric Nonlinearity

Settings

| **Description** | **Value** |
| --- | --- |
| Formulation | From study step |
| Strain decomposition | Automatic |

#### Energy Dissipation

Settings

| **Description** | **Value** |
| --- | --- |
| Calculate dissipated energy | Off |

#### Quadrature Settings

Settings

| **Description** | **Value** |
| --- | --- |
| Reduced integration | Off |

#### Coordinate System Selection

Settings

| **Description** | **Value** |
| --- | --- |
| Coordinate system | Material XZ - plane System (comp1_xz_sys) |

#### Model Input

Settings

| **Description** | **Value** |
| --- | --- |
| Volume reference temperature | Common model input |

Properties from material

| **Property** | **Material** | **Property group** |
| --- | --- | --- |
| Elasticity matrix, Voigt notation | Lead Zirconate Titanate (PZT-5H) | Stress-charge form |
| Coupling matrix, Voigt notation | Lead Zirconate Titanate (PZT-5H) | Stress-charge form |
| Relative permittivity | Lead Zirconate Titanate (PZT-5H) | Stress-charge form |
| Density | Lead Zirconate Titanate (PZT-5H) | Basic |
| Density | Polystyrene (PS) [solid] | Basic |

#### Variables

| **Name** | **Expression** | **Unit** | **Description** | **Selection** | **Details** |
| --- | --- | --- | --- | --- | --- |
| solid.DrX | comp1_xz_sys.T11*solid.Dr1+comp1_xz_sys.T21*solid.Dr2+comp1_xz_sys.T31*solid.Dr3 | C/m² | Remanent electric displacement, X-component | Domain 5 |  |
| solid.DrY | comp1_xz_sys.T12*solid.Dr1+comp1_xz_sys.T22*solid.Dr2+comp1_xz_sys.T32*solid.Dr3 | C/m² | Remanent electric displacement, Y-component | Domain 5 |  |
| solid.DrZ | comp1_xz_sys.T13*solid.Dr1+comp1_xz_sys.T23*solid.Dr2+comp1_xz_sys.T33*solid.Dr3 | C/m² | Remanent electric displacement, Z-component | Domain 5 |  |
| solid.epsilonrXX | (comp1_xz_sys.T11*solid.epsilonrS11+comp1_xz_sys.T21*solid.epsilonrS12+comp1_xz_sys.T31*solid.epsilonrS13)*comp1_xz_sys.T11+(comp1_xz_sys.T11*solid.epsilonrS12+comp1_xz_sys.T21*solid.epsilonrS22+comp1_xz_sys.T31*solid.epsilonrS23)*comp1_xz_sys.T21+(comp1_xz_sys.T11*solid.epsilonrS13+comp1_xz_sys.T21*solid.epsilonrS23+comp1_xz_sys.T31*solid.epsilonrS33)*comp1_xz_sys.T31 | 1 | Relative permittivity, piezoelectric solid, XX-component | Domain 5 |  |
| solid.epsilonrYX | (comp1_xz_sys.T12*solid.epsilonrS11+comp1_xz_sys.T22*solid.epsilonrS12+comp1_xz_sys.T32*solid.epsilonrS13)*comp1_xz_sys.T11+(comp1_xz_sys.T12*solid.epsilonrS12+comp1_xz_sys.T22*solid.epsilonrS22+comp1_xz_sys.T32*solid.epsilonrS23)*comp1_xz_sys.T21+(comp1_xz_sys.T12*solid.epsilonrS13+comp1_xz_sys.T22*solid.epsilonrS23+comp1_xz_sys.T32*solid.epsilonrS33)*comp1_xz_sys.T31 | 1 | Relative permittivity, piezoelectric solid, YX-component | Domain 5 |  |
| solid.epsilonrZX | (comp1_xz_sys.T13*solid.epsilonrS11+comp1_xz_sys.T23*solid.epsilonrS12+comp1_xz_sys.T33*solid.epsilonrS13)*comp1_xz_sys.T11+(comp1_xz_sys.T13*solid.epsilonrS12+comp1_xz_sys.T23*solid.epsilonrS22+comp1_xz_sys.T33*solid.epsilonrS23)*comp1_xz_sys.T21+(comp1_xz_sys.T13*solid.epsilonrS13+comp1_xz_sys.T23*solid.epsilonrS23+comp1_xz_sys.T33*solid.epsilonrS33)*comp1_xz_sys.T31 | 1 | Relative permittivity, piezoelectric solid, ZX-component | Domain 5 |  |
| solid.epsilonrXY | (comp1_xz_sys.T11*solid.epsilonrS11+comp1_xz_sys.T21*solid.epsilonrS12+comp1_xz_sys.T31*solid.epsilonrS13)*comp1_xz_sys.T12+(comp1_xz_sys.T11*solid.epsilonrS12+comp1_xz_sys.T21*solid.epsilonrS22+comp1_xz_sys.T31*solid.epsilonrS23)*comp1_xz_sys.T22+(comp1_xz_sys.T11*solid.epsilonrS13+comp1_xz_sys.T21*solid.epsilonrS23+comp1_xz_sys.T31*solid.epsilonrS33)*comp1_xz_sys.T32 | 1 | Relative permittivity, piezoelectric solid, XY-component | Domain 5 |  |
| solid.epsilonrYY | (comp1_xz_sys.T12*solid.epsilonrS11+comp1_xz_sys.T22*solid.epsilonrS12+comp1_xz_sys.T32*solid.epsilonrS13)*comp1_xz_sys.T12+(comp1_xz_sys.T12*solid.epsilonrS12+comp1_xz_sys.T22*solid.epsilonrS22+comp1_xz_sys.T32*solid.epsilonrS23)*comp1_xz_sys.T22+(comp1_xz_sys.T12*solid.epsilonrS13+comp1_xz_sys.T22*solid.epsilonrS23+comp1_xz_sys.T32*solid.epsilonrS33)*comp1_xz_sys.T32 | 1 | Relative permittivity, piezoelectric solid, YY-component | Domain 5 |  |
| solid.epsilonrZY | (comp1_xz_sys.T13*solid.epsilonrS11+comp1_xz_sys.T23*solid.epsilonrS12+comp1_xz_sys.T33*solid.epsilonrS13)*comp1_xz_sys.T12+(comp1_xz_sys.T13*solid.epsilonrS12+comp1_xz_sys.T23*solid.epsilonrS22+comp1_xz_sys.T33*solid.epsilonrS23)*comp1_xz_sys.T22+(comp1_xz_sys.T13*solid.epsilonrS13+comp1_xz_sys.T23*solid.epsilonrS23+comp1_xz_sys.T33*solid.epsilonrS33)*comp1_xz_sys.T32 | 1 | Relative permittivity, piezoelectric solid, ZY-component | Domain 5 |  |
| solid.epsilonrXZ | (comp1_xz_sys.T11*solid.epsilonrS11+comp1_xz_sys.T21*solid.epsilonrS12+comp1_xz_sys.T31*solid.epsilonrS13)*comp1_xz_sys.T13+(comp1_xz_sys.T11*solid.epsilonrS12+comp1_xz_sys.T21*solid.epsilonrS22+comp1_xz_sys.T31*solid.epsilonrS23)*comp1_xz_sys.T23+(comp1_xz_sys.T11*solid.epsilonrS13+comp1_xz_sys.T21*solid.epsilonrS23+comp1_xz_sys.T31*solid.epsilonrS33)*comp1_xz_sys.T33 | 1 | Relative permittivity, piezoelectric solid, XZ-component | Domain 5 |  |
| solid.epsilonrYZ | (comp1_xz_sys.T12*solid.epsilonrS11+comp1_xz_sys.T22*solid.epsilonrS12+comp1_xz_sys.T32*solid.epsilonrS13)*comp1_xz_sys.T13+(comp1_xz_sys.T12*solid.epsilonrS12+comp1_xz_sys.T22*solid.epsilonrS22+comp1_xz_sys.T32*solid.epsilonrS23)*comp1_xz_sys.T23+(comp1_xz_sys.T12*solid.epsilonrS13+comp1_xz_sys.T22*solid.epsilonrS23+comp1_xz_sys.T32*solid.epsilonrS33)*comp1_xz_sys.T33 | 1 | Relative permittivity, piezoelectric solid, YZ-component | Domain 5 |  |
| solid.epsilonrZZ | (comp1_xz_sys.T13*solid.epsilonrS11+comp1_xz_sys.T23*solid.epsilonrS12+comp1_xz_sys.T33*solid.epsilonrS13)*comp1_xz_sys.T13+(comp1_xz_sys.T13*solid.epsilonrS12+comp1_xz_sys.T23*solid.epsilonrS22+comp1_xz_sys.T33*solid.epsilonrS23)*comp1_xz_sys.T23+(comp1_xz_sys.T13*solid.epsilonrS13+comp1_xz_sys.T23*solid.epsilonrS23+comp1_xz_sys.T33*solid.epsilonrS33)*comp1_xz_sys.T33 | 1 | Relative permittivity, piezoelectric solid, ZZ-component | Domain 5 |  |
| solid.PpzeX | comp1_xz_sys.T11*(solid.eES11*solid.eel11+solid.eES12*solid.eel22+solid.eES13*solid.eel33+2*solid.eES14*solid.eel23+2*solid.eES15*solid.eel13+2*solid.eES16*solid.eel12)+comp1_xz_sys.T21*(solid.eES21*solid.eel11+solid.eES22*solid.eel22+solid.eES23*solid.eel33+2*solid.eES24*solid.eel23+2*solid.eES25*solid.eel13+2*solid.eES26*solid.eel12)+comp1_xz_sys.T31*(solid.eES31*solid.eel11+solid.eES32*solid.eel22+solid.eES33*solid.eel33+2*solid.eES34*solid.eel23+2*solid.eES35*solid.eel13+2*solid.eES36*solid.eel12) | C/m² | Piezoelectric polarization, X-component | Domain 5 | + operation |
| solid.PpzeY | comp1_xz_sys.T12*(solid.eES11*solid.eel11+solid.eES12*solid.eel22+solid.eES13*solid.eel33+2*solid.eES14*solid.eel23+2*solid.eES15*solid.eel13+2*solid.eES16*solid.eel12)+comp1_xz_sys.T22*(solid.eES21*solid.eel11+solid.eES22*solid.eel22+solid.eES23*solid.eel33+2*solid.eES24*solid.eel23+2*solid.eES25*solid.eel13+2*solid.eES26*solid.eel12)+comp1_xz_sys.T32*(solid.eES31*solid.eel11+solid.eES32*solid.eel22+solid.eES33*solid.eel33+2*solid.eES34*solid.eel23+2*solid.eES35*solid.eel13+2*solid.eES36*solid.eel12) | C/m² | Piezoelectric polarization, Y-component | Domain 5 | + operation |
| solid.PpzeZ | comp1_xz_sys.T13*(solid.eES11*solid.eel11+solid.eES12*solid.eel22+solid.eES13*solid.eel33+2*solid.eES14*solid.eel23+2*solid.eES15*solid.eel13+2*solid.eES16*solid.eel12)+comp1_xz_sys.T23*(solid.eES21*solid.eel11+solid.eES22*solid.eel22+solid.eES23*solid.eel33+2*solid.eES24*solid.eel23+2*solid.eES25*solid.eel13+2*solid.eES26*solid.eel12)+comp1_xz_sys.T33*(solid.eES31*solid.eel11+solid.eES32*solid.eel22+solid.eES33*solid.eel33+2*solid.eES34*solid.eel23+2*solid.eES35*solid.eel13+2*solid.eES36*solid.eel12) | C/m² | Piezoelectric polarization, Z-component | Domain 5 | + operation |
| solid.DpzeX | 0 | C/m² | Electric displacement field, X-component | Domain 5 | + operation |
| solid.DpzeY | 0 | C/m² | Electric displacement field, Y-component | Domain 5 | + operation |
| solid.DpzeZ | 0 | C/m² | Electric displacement field, Z-component | Domain 5 | + operation |
| solid.EpzeX | 0 | V/m | Electric field, X-component | Domain 5 | + operation |
| solid.EpzeY | 0 | V/m | Electric field, Y-component | Domain 5 | + operation |
| solid.EpzeZ | 0 | V/m | Electric field, Z-component | Domain 5 | + operation |
| solid.disp | sqrteps(real(u)^2+real(v)^2) | m | Displacement magnitude | Domain 5 |  |
| solid.disp_rms | sqrt(0.5*(realdot(u,u)+realdot(v,v))) | m | Displacement, RMS | Domain 5 |  |
| solid.curlUX | 0 | 1 | Curl of displacement, X-component | Domain 5 |  |
| solid.curlUY | 0 | 1 | Curl of displacement, Y-component | Domain 5 |  |
| solid.curlUZ | solid.gradUyX-solid.gradUxY | 1 | Curl of displacement, Z-component | Domain 5 |  |
| solid.gradUxX | uX | 1 | Displacement gradient, xX-component | Domain 5 |  |
| solid.gradUyX | vX | 1 | Displacement gradient, yX-component | Domain 5 |  |
| solid.gradUzX | 0 | 1 | Displacement gradient, zX-component | Domain 5 |  |
| solid.gradUxY | uY | 1 | Displacement gradient, xY-component | Domain 5 |  |
| solid.gradUyY | vY | 1 | Displacement gradient, yY-component | Domain 5 |  |
| solid.gradUzY | 0 | 1 | Displacement gradient, zY-component | Domain 5 |  |
| solid.gradUxZ | 0 | 1 | Displacement gradient, xZ-component | Domain 5 |  |
| solid.gradUyZ | 0 | 1 | Displacement gradient, yZ-component | Domain 5 |  |
| solid.gradUzZ | 0 | 1 | Displacement gradient, zZ-component | Domain 5 |  |
| solid.FdxX | 1+solid.gradUxX | 1 | Deformation gradient, xX-component | Domain 5 |  |
| solid.FdyX | solid.gradUyX | 1 | Deformation gradient, yX-component | Domain 5 |  |
| solid.FdzX | solid.gradUzX | 1 | Deformation gradient, zX-component | Domain 5 |  |
| solid.FdxY | solid.gradUxY | 1 | Deformation gradient, xY-component | Domain 5 |  |
| solid.FdyY | 1+solid.gradUyY | 1 | Deformation gradient, yY-component | Domain 5 |  |
| solid.FdzY | solid.gradUzY | 1 | Deformation gradient, zY-component | Domain 5 |  |
| solid.FdxZ | solid.gradUxZ | 1 | Deformation gradient, xZ-component | Domain 5 |  |
| solid.FdyZ | solid.gradUyZ | 1 | Deformation gradient, yZ-component | Domain 5 |  |
| solid.FdzZ | 1+solid.gradUzZ | 1 | Deformation gradient, zZ-component | Domain 5 |  |
| solid.FdiXx | (solid.FdyY*solid.FdzZ-solid.FdyZ*solid.FdzY)/solid.J | 1 | Deformation gradient inverse, Xx-component | Domain 5 |  |
| solid.FdiYx | (solid.FdyZ*solid.FdzX-solid.FdyX*solid.FdzZ)/solid.J | 1 | Deformation gradient inverse, Yx-component | Domain 5 |  |
| solid.FdiZx | (solid.FdyX*solid.FdzY-solid.FdyY*solid.FdzX)/solid.J | 1 | Deformation gradient inverse, Zx-component | Domain 5 |  |
| solid.FdiXy | (solid.FdxZ*solid.FdzY-solid.FdxY*solid.FdzZ)/solid.J | 1 | Deformation gradient inverse, Xy-component | Domain 5 |  |
| solid.FdiYy | (solid.FdxX*solid.FdzZ-solid.FdxZ*solid.FdzX)/solid.J | 1 | Deformation gradient inverse, Yy-component | Domain 5 |  |
| solid.FdiZy | (solid.FdxY*solid.FdzX-solid.FdxX*solid.FdzY)/solid.J | 1 | Deformation gradient inverse, Zy-component | Domain 5 |  |
| solid.FdiXz | (solid.FdxY*solid.FdyZ-solid.FdxZ*solid.FdyY)/solid.J | 1 | Deformation gradient inverse, Xz-component | Domain 5 |  |
| solid.FdiYz | (solid.FdxZ*solid.FdyX-solid.FdxX*solid.FdyZ)/solid.J | 1 | Deformation gradient inverse, Yz-component | Domain 5 |  |
| solid.FdiZz | (solid.FdxX*solid.FdyY-solid.FdxY*solid.FdyX)/solid.J | 1 | Deformation gradient inverse, Zz-component | Domain 5 |  |
| solid.J | solid.FdxX*solid.FdyY*solid.FdzZ+solid.FdxY*solid.FdyZ*solid.FdzX+solid.FdxZ*solid.FdyX*solid.FdzY-solid.FdxX*solid.FdyZ*solid.FdzY-solid.FdxY*solid.FdyX*solid.FdzZ-solid.FdxZ*solid.FdyY*solid.FdzX | 1 | Volume ratio | Domain 5 |  |
| solid.Fdlx1 | solid.FdxX*solid.sysT11+solid.FdxY*solid.sysT12+solid.FdxZ*solid.sysT13 | 1 | Deformation gradient, local, x1-component | Domain 5 |  |
| solid.Fdly1 | solid.FdyX*solid.sysT11+solid.FdyY*solid.sysT12+solid.FdyZ*solid.sysT13 | 1 | Deformation gradient, local, y1-component | Domain 5 |  |
| solid.Fdlz1 | solid.FdzX*solid.sysT11+solid.FdzY*solid.sysT12+solid.FdzZ*solid.sysT13 | 1 | Deformation gradient, local, z1-component | Domain 5 |  |
| solid.Fdlx2 | solid.FdxX*solid.sysT21+solid.FdxY*solid.sysT22+solid.FdxZ*solid.sysT23 | 1 | Deformation gradient, local, x2-component | Domain 5 |  |
| solid.Fdly2 | solid.FdyX*solid.sysT21+solid.FdyY*solid.sysT22+solid.FdyZ*solid.sysT23 | 1 | Deformation gradient, local, y2-component | Domain 5 |  |
| solid.Fdlz2 | solid.FdzX*solid.sysT21+solid.FdzY*solid.sysT22+solid.FdzZ*solid.sysT23 | 1 | Deformation gradient, local, z2-component | Domain 5 |  |
| solid.Fdlx3 | solid.FdxX*solid.sysT31+solid.FdxY*solid.sysT32+solid.FdxZ*solid.sysT33 | 1 | Deformation gradient, local, x3-component | Domain 5 |  |
| solid.Fdly3 | solid.FdyX*solid.sysT31+solid.FdyY*solid.sysT32+solid.FdyZ*solid.sysT33 | 1 | Deformation gradient, local, y3-component | Domain 5 |  |
| solid.Fdlz3 | solid.FdzX*solid.sysT31+solid.FdzY*solid.sysT32+solid.FdzZ*solid.sysT33 | 1 | Deformation gradient, local, z3-component | Domain 5 |  |
| solid.Fdil1x | (solid.Fdly2*solid.Fdlz3-solid.Fdly3*solid.Fdlz2)/solid.J | 1 | Deformation gradient inverse, local coordinate system, 1x-component | Domain 5 |  |
| solid.Fdil2x | (solid.Fdly3*solid.Fdlz1-solid.Fdly1*solid.Fdlz3)/solid.J | 1 | Deformation gradient inverse, local coordinate system, 2x-component | Domain 5 |  |
| solid.Fdil3x | (solid.Fdly1*solid.Fdlz2-solid.Fdly2*solid.Fdlz1)/solid.J | 1 | Deformation gradient inverse, local coordinate system, 3x-component | Domain 5 |  |
| solid.Fdil1y | (solid.Fdlx3*solid.Fdlz2-solid.Fdlx2*solid.Fdlz3)/solid.J | 1 | Deformation gradient inverse, local coordinate system, 1y-component | Domain 5 |  |
| solid.Fdil2y | (solid.Fdlx1*solid.Fdlz3-solid.Fdlx3*solid.Fdlz1)/solid.J | 1 | Deformation gradient inverse, local coordinate system, 2y-component | Domain 5 |  |
| solid.Fdil3y | (solid.Fdlx2*solid.Fdlz1-solid.Fdlx1*solid.Fdlz2)/solid.J | 1 | Deformation gradient inverse, local coordinate system, 3y-component | Domain 5 |  |
| solid.Fdil1z | (solid.Fdlx2*solid.Fdly3-solid.Fdlx3*solid.Fdly2)/solid.J | 1 | Deformation gradient inverse, local coordinate system, 1z-component | Domain 5 |  |
| solid.Fdil2z | (solid.Fdlx3*solid.Fdly1-solid.Fdlx1*solid.Fdly3)/solid.J | 1 | Deformation gradient inverse, local coordinate system, 2z-component | Domain 5 |  |
| solid.Fdil3z | (solid.Fdlx1*solid.Fdly2-solid.Fdlx2*solid.Fdly1)/solid.J | 1 | Deformation gradient inverse, local coordinate system, 3z-component | Domain 5 |  |
| solid.Ji | 1/(solid.Fiil11*solid.Fiil22*solid.Fiil33+solid.Fiil12*solid.Fiil23*solid.Fiil31+solid.Fiil13*solid.Fiil21*solid.Fiil32-solid.Fiil11*solid.Fiil23*solid.Fiil32-solid.Fiil12*solid.Fiil21*solid.Fiil33-solid.Fiil13*solid.Fiil22*solid.Fiil31) | 1 | Inelastic volume ratio | Domain 5 |  |
| solid.Fil11 | (solid.Fiil22*solid.Fiil33-solid.Fiil23*solid.Fiil32)*solid.Ji | 1 | Inelastic deformation gradient, local coordinate system, 11-component | Domain 5 | Matrix multiplication |
| solid.Fil21 | (solid.Fiil23*solid.Fiil31-solid.Fiil21*solid.Fiil33)*solid.Ji | 1 | Inelastic deformation gradient, local coordinate system, 21-component | Domain 5 | Matrix multiplication |
| solid.Fil31 | (solid.Fiil21*solid.Fiil32-solid.Fiil22*solid.Fiil31)*solid.Ji | 1 | Inelastic deformation gradient, local coordinate system, 31-component | Domain 5 | Matrix multiplication |
| solid.Fil12 | (solid.Fiil13*solid.Fiil32-solid.Fiil12*solid.Fiil33)*solid.Ji | 1 | Inelastic deformation gradient, local coordinate system, 12-component | Domain 5 | Matrix multiplication |
| solid.Fil22 | (solid.Fiil11*solid.Fiil33-solid.Fiil13*solid.Fiil31)*solid.Ji | 1 | Inelastic deformation gradient, local coordinate system, 22-component | Domain 5 | Matrix multiplication |
| solid.Fil32 | (solid.Fiil12*solid.Fiil31-solid.Fiil11*solid.Fiil32)*solid.Ji | 1 | Inelastic deformation gradient, local coordinate system, 32-component | Domain 5 | Matrix multiplication |
| solid.Fil13 | (solid.Fiil12*solid.Fiil23-solid.Fiil13*solid.Fiil22)*solid.Ji | 1 | Inelastic deformation gradient, local coordinate system, 13-component | Domain 5 | Matrix multiplication |
| solid.Fil23 | (solid.Fiil13*solid.Fiil21-solid.Fiil11*solid.Fiil23)*solid.Ji | 1 | Inelastic deformation gradient, local coordinate system, 23-component | Domain 5 | Matrix multiplication |
| solid.Fil33 | (solid.Fiil11*solid.Fiil22-solid.Fiil12*solid.Fiil21)*solid.Ji | 1 | Inelastic deformation gradient, local coordinate system, 33-component | Domain 5 | Matrix multiplication |
| solid.Fiil11 | 1 | 1 | Inelastic deformation gradient inverse, local coordinate system, 11-component | Domain 5 | Matrix multiplication |
| solid.Fiil21 | 0 | 1 | Inelastic deformation gradient inverse, local coordinate system, 21-component | Domain 5 | Matrix multiplication |
| solid.Fiil31 | 0 | 1 | Inelastic deformation gradient inverse, local coordinate system, 31-component | Domain 5 | Matrix multiplication |
| solid.Fiil12 | 0 | 1 | Inelastic deformation gradient inverse, local coordinate system, 12-component | Domain 5 | Matrix multiplication |
| solid.Fiil22 | 1 | 1 | Inelastic deformation gradient inverse, local coordinate system, 22-component | Domain 5 | Matrix multiplication |
| solid.Fiil32 | 0 | 1 | Inelastic deformation gradient inverse, local coordinate system, 32-component | Domain 5 | Matrix multiplication |
| solid.Fiil13 | 0 | 1 | Inelastic deformation gradient inverse, local coordinate system, 13-component | Domain 5 | Matrix multiplication |
| solid.Fiil23 | 0 | 1 | Inelastic deformation gradient inverse, local coordinate system, 23-component | Domain 5 | Matrix multiplication |
| solid.Fiil33 | 1 | 1 | Inelastic deformation gradient inverse, local coordinate system, 33-component | Domain 5 | Matrix multiplication |
| solid.Jel | sqrt(solid.Cel11*solid.Cel22*solid.Cel33+2*solid.Cel12*solid.Cel23*solid.Cel13-solid.Cel11*solid.Cel23^2-solid.Cel12^2*solid.Cel33-solid.Cel22*solid.Cel13^2) | 1 | Elastic volume ratio | Domain 5 |  |
| solid.CXX | solid.FdxX^2+solid.FdyX^2+solid.FdzX^2 | 1 | Cauchy–Green tensor, XX-component | Domain 5 |  |
| solid.CXY | solid.FdxX*solid.FdxY+solid.FdyX*solid.FdyY+solid.FdzX*solid.FdzY | 1 | Cauchy–Green tensor, XY-component | Domain 5 |  |
| solid.CXZ | solid.FdxX*solid.FdxZ+solid.FdyX*solid.FdyZ+solid.FdzX*solid.FdzZ | 1 | Cauchy–Green tensor, XZ-component | Domain 5 |  |
| solid.CYY | solid.FdxY^2+solid.FdyY^2+solid.FdzY^2 | 1 | Cauchy–Green tensor, YY-component | Domain 5 |  |
| solid.CYZ | solid.FdxY*solid.FdxZ+solid.FdyY*solid.FdyZ+solid.FdzY*solid.FdzZ | 1 | Cauchy–Green tensor, YZ-component | Domain 5 |  |
| solid.CZZ | solid.FdxZ^2+solid.FdyZ^2+solid.FdzZ^2 | 1 | Cauchy–Green tensor, ZZ-component | Domain 5 |  |
| solid.Cl11 | 1+2*solid.el11 | 1 | Cauchy–Green tensor, local coordinate system, 11-component | Domain 5 |  |
| solid.Cl12 | 2*solid.el12 | 1 | Cauchy–Green tensor, local coordinate system, 12-component | Domain 5 |  |
| solid.Cl13 | 2*solid.el13 | 1 | Cauchy–Green tensor, local coordinate system, 13-component | Domain 5 |  |
| solid.Cl22 | 1+2*solid.el22 | 1 | Cauchy–Green tensor, local coordinate system, 22-component | Domain 5 |  |
| solid.Cl23 | 2*solid.el23 | 1 | Cauchy–Green tensor, local coordinate system, 23-component | Domain 5 |  |
| solid.Cl33 | 1+2*solid.el33 | 1 | Cauchy–Green tensor, local coordinate system, 33-component | Domain 5 |  |
| solid.Cel11 | solid.FiilCl11*solid.Fiil11+solid.FiilCl12*solid.Fiil21+solid.FiilCl13*solid.Fiil31 | 1 | Elastic Cauchy–Green tensor, local coordinate system, 11-component | Domain 5 |  |
| solid.Cel12 | solid.FiilCl11*solid.Fiil12+solid.FiilCl12*solid.Fiil22+solid.FiilCl13*solid.Fiil32 | 1 | Elastic Cauchy–Green tensor, local coordinate system, 12-component | Domain 5 |  |
| solid.Cel13 | solid.FiilCl11*solid.Fiil13+solid.FiilCl12*solid.Fiil23+solid.FiilCl13*solid.Fiil33 | 1 | Elastic Cauchy–Green tensor, local coordinate system, 13-component | Domain 5 |  |
| solid.Cel22 | solid.FiilCl21*solid.Fiil12+solid.FiilCl22*solid.Fiil22+solid.FiilCl23*solid.Fiil32 | 1 | Elastic Cauchy–Green tensor, local coordinate system, 22-component | Domain 5 |  |
| solid.Cel23 | solid.FiilCl21*solid.Fiil13+solid.FiilCl22*solid.Fiil23+solid.FiilCl23*solid.Fiil33 | 1 | Elastic Cauchy–Green tensor, local coordinate system, 23-component | Domain 5 |  |
| solid.Cel33 | solid.FiilCl31*solid.Fiil13+solid.FiilCl32*solid.Fiil23+solid.FiilCl33*solid.Fiil33 | 1 | Elastic Cauchy–Green tensor, local coordinate system, 33-component | Domain 5 |  |
| solid.Ceil11 | (solid.Cel22*solid.Cel33-solid.Cel23^2)/(solid.Cel11*solid.Cel22*solid.Cel33+2*solid.Cel12*solid.Cel23*solid.Cel13-solid.Cel11*solid.Cel23^2-solid.Cel12^2*solid.Cel33-solid.Cel22*solid.Cel13^2) | 1 | Elastic Cauchy–Green tensor inverse, local coordinate system, 11-component | Domain 5 |  |
| solid.Ceil12 | (solid.Cel23*solid.Cel13-solid.Cel12*solid.Cel33)/(solid.Cel11*solid.Cel22*solid.Cel33+2*solid.Cel12*solid.Cel23*solid.Cel13-solid.Cel11*solid.Cel23^2-solid.Cel12^2*solid.Cel33-solid.Cel22*solid.Cel13^2) | 1 | Elastic Cauchy–Green tensor inverse, local coordinate system, 12-component | Domain 5 |  |
| solid.Ceil13 | (solid.Cel12*solid.Cel23-solid.Cel22*solid.Cel13)/(solid.Cel11*solid.Cel22*solid.Cel33+2*solid.Cel12*solid.Cel23*solid.Cel13-solid.Cel11*solid.Cel23^2-solid.Cel12^2*solid.Cel33-solid.Cel22*solid.Cel13^2) | 1 | Elastic Cauchy–Green tensor inverse, local coordinate system, 13-component | Domain 5 |  |
| solid.Ceil22 | (solid.Cel11*solid.Cel33-solid.Cel13^2)/(solid.Cel11*solid.Cel22*solid.Cel33+2*solid.Cel12*solid.Cel23*solid.Cel13-solid.Cel11*solid.Cel23^2-solid.Cel12^2*solid.Cel33-solid.Cel22*solid.Cel13^2) | 1 | Elastic Cauchy–Green tensor inverse, local coordinate system, 22-component | Domain 5 |  |
| solid.Ceil23 | (solid.Cel12*solid.Cel13-solid.Cel11*solid.Cel23)/(solid.Cel11*solid.Cel22*solid.Cel33+2*solid.Cel12*solid.Cel23*solid.Cel13-solid.Cel11*solid.Cel23^2-solid.Cel12^2*solid.Cel33-solid.Cel22*solid.Cel13^2) | 1 | Elastic Cauchy–Green tensor inverse, local coordinate system, 23-component | Domain 5 |  |
| solid.Ceil33 | (solid.Cel11*solid.Cel22-solid.Cel12^2)/(solid.Cel11*solid.Cel22*solid.Cel33+2*solid.Cel12*solid.Cel23*solid.Cel13-solid.Cel11*solid.Cel23^2-solid.Cel12^2*solid.Cel33-solid.Cel22*solid.Cel13^2) | 1 | Elastic Cauchy–Green tensor inverse, local coordinate system, 33-component | Domain 5 |  |
| solid.eXX | solid.gradUxX | 1 | Strain tensor, XX-component | Domain 5 | + operation |
| solid.eXY | 0.5*(solid.gradUxY+solid.gradUyX) | 1 | Strain tensor, XY-component | Domain 5 | + operation |
| solid.eXZ | 0.5*(solid.gradUxZ+solid.gradUzX) | 1 | Strain tensor, XZ-component | Domain 5 | + operation |
| solid.eYY | solid.gradUyY | 1 | Strain tensor, YY-component | Domain 5 | + operation |
| solid.eYZ | 0.5*(solid.gradUyZ+solid.gradUzY) | 1 | Strain tensor, YZ-component | Domain 5 | + operation |
| solid.eZZ | solid.gradUzZ | 1 | Strain tensor, ZZ-component | Domain 5 | + operation |
| solid.el11 | (solid.sysT11*solid.eXX+solid.sysT12*solid.eXY+solid.sysT13*solid.eXZ)*solid.sysT11+(solid.sysT11*solid.eXY+solid.sysT12*solid.eYY+solid.sysT13*solid.eYZ)*solid.sysT12+(solid.sysT11*solid.eXZ+solid.sysT12*solid.eYZ+solid.sysT13*solid.eZZ)*solid.sysT13 | 1 | Strain tensor, local coordinate system, 11-component | Domain 5 | + operation |
| solid.el12 | (solid.sysT11*solid.eXX+solid.sysT12*solid.eXY+solid.sysT13*solid.eXZ)*solid.sysT21+(solid.sysT11*solid.eXY+solid.sysT12*solid.eYY+solid.sysT13*solid.eYZ)*solid.sysT22+(solid.sysT11*solid.eXZ+solid.sysT12*solid.eYZ+solid.sysT13*solid.eZZ)*solid.sysT23 | 1 | Strain tensor, local coordinate system, 12-component | Domain 5 | + operation |
| solid.el13 | (solid.sysT11*solid.eXX+solid.sysT12*solid.eXY+solid.sysT13*solid.eXZ)*solid.sysT31+(solid.sysT11*solid.eXY+solid.sysT12*solid.eYY+solid.sysT13*solid.eYZ)*solid.sysT32+(solid.sysT11*solid.eXZ+solid.sysT12*solid.eYZ+solid.sysT13*solid.eZZ)*solid.sysT33 | 1 | Strain tensor, local coordinate system, 13-component | Domain 5 | + operation |
| solid.el22 | (solid.sysT21*solid.eXX+solid.sysT22*solid.eXY+solid.sysT23*solid.eXZ)*solid.sysT21+(solid.sysT21*solid.eXY+solid.sysT22*solid.eYY+solid.sysT23*solid.eYZ)*solid.sysT22+(solid.sysT21*solid.eXZ+solid.sysT22*solid.eYZ+solid.sysT23*solid.eZZ)*solid.sysT23 | 1 | Strain tensor, local coordinate system, 22-component | Domain 5 | + operation |
| solid.el23 | (solid.sysT21*solid.eXX+solid.sysT22*solid.eXY+solid.sysT23*solid.eXZ)*solid.sysT31+(solid.sysT21*solid.eXY+solid.sysT22*solid.eYY+solid.sysT23*solid.eYZ)*solid.sysT32+(solid.sysT21*solid.eXZ+solid.sysT22*solid.eYZ+solid.sysT23*solid.eZZ)*solid.sysT33 | 1 | Strain tensor, local coordinate system, 23-component | Domain 5 | + operation |
| solid.el33 | (solid.sysT31*solid.eXX+solid.sysT32*solid.eXY+solid.sysT33*solid.eXZ)*solid.sysT31+(solid.sysT31*solid.eXY+solid.sysT32*solid.eYY+solid.sysT33*solid.eYZ)*solid.sysT32+(solid.sysT31*solid.eXZ+solid.sysT32*solid.eYZ+solid.sysT33*solid.eZZ)*solid.sysT33 | 1 | Strain tensor, local coordinate system, 33-component | Domain 5 | + operation |
| solid.evol | solid.eXX+solid.eYY+solid.eZZ | 1 | Volumetric strain | Domain 5 |  |
| solid.eldev11 | solid.el11-solid.evol/3 | 1 | Deviatoric strain tensor, local coordinate system, 11-component | Domain 5 |  |
| solid.eldev12 | solid.el12 | 1 | Deviatoric strain tensor, local coordinate system, 12-component | Domain 5 |  |
| solid.eldev13 | solid.el13 | 1 | Deviatoric strain tensor, local coordinate system, 13-component | Domain 5 |  |
| solid.eldev22 | solid.el22-solid.evol/3 | 1 | Deviatoric strain tensor, local coordinate system, 22-component | Domain 5 |  |
| solid.eldev23 | solid.el23 | 1 | Deviatoric strain tensor, local coordinate system, 23-component | Domain 5 |  |
| solid.eldev33 | solid.el33-solid.evol/3 | 1 | Deviatoric strain tensor, local coordinate system, 33-component | Domain 5 |  |
| solid.edeve | sqrt(2*(solid.eldev11^2+2*solid.eldev12^2+2*solid.eldev13^2+solid.eldev22^2+2*solid.eldev23^2+solid.eldev33^2)/3+eps^2) | 1 | Equivalent deviatoric strain | Domain 5 |  |
| solid.eeXX | (solid.sysT11*solid.eel11+solid.sysT21*solid.eel12+solid.sysT31*solid.eel13)*solid.sysT11+(solid.sysT11*solid.eel12+solid.sysT21*solid.eel22+solid.sysT31*solid.eel23)*solid.sysT21+(solid.sysT11*solid.eel13+solid.sysT21*solid.eel23+solid.sysT31*solid.eel33)*solid.sysT31 | 1 | Elastic strain tensor, XX-component | Domain 5 |  |
| solid.eeXY | (solid.sysT11*solid.eel11+solid.sysT21*solid.eel12+solid.sysT31*solid.eel13)*solid.sysT12+(solid.sysT11*solid.eel12+solid.sysT21*solid.eel22+solid.sysT31*solid.eel23)*solid.sysT22+(solid.sysT11*solid.eel13+solid.sysT21*solid.eel23+solid.sysT31*solid.eel33)*solid.sysT32 | 1 | Elastic strain tensor, XY-component | Domain 5 |  |
| solid.eeXZ | (solid.sysT11*solid.eel11+solid.sysT21*solid.eel12+solid.sysT31*solid.eel13)*solid.sysT13+(solid.sysT11*solid.eel12+solid.sysT21*solid.eel22+solid.sysT31*solid.eel23)*solid.sysT23+(solid.sysT11*solid.eel13+solid.sysT21*solid.eel23+solid.sysT31*solid.eel33)*solid.sysT33 | 1 | Elastic strain tensor, XZ-component | Domain 5 |  |
| solid.eeYY | (solid.sysT12*solid.eel11+solid.sysT22*solid.eel12+solid.sysT32*solid.eel13)*solid.sysT12+(solid.sysT12*solid.eel12+solid.sysT22*solid.eel22+solid.sysT32*solid.eel23)*solid.sysT22+(solid.sysT12*solid.eel13+solid.sysT22*solid.eel23+solid.sysT32*solid.eel33)*solid.sysT32 | 1 | Elastic strain tensor, YY-component | Domain 5 |  |
| solid.eeYZ | (solid.sysT12*solid.eel11+solid.sysT22*solid.eel12+solid.sysT32*solid.eel13)*solid.sysT13+(solid.sysT12*solid.eel12+solid.sysT22*solid.eel22+solid.sysT32*solid.eel23)*solid.sysT23+(solid.sysT12*solid.eel13+solid.sysT22*solid.eel23+solid.sysT32*solid.eel33)*solid.sysT33 | 1 | Elastic strain tensor, YZ-component | Domain 5 |  |
| solid.eeZZ | (solid.sysT13*solid.eel11+solid.sysT23*solid.eel12+solid.sysT33*solid.eel13)*solid.sysT13+(solid.sysT13*solid.eel12+solid.sysT23*solid.eel22+solid.sysT33*solid.eel23)*solid.sysT23+(solid.sysT13*solid.eel13+solid.sysT23*solid.eel23+solid.sysT33*solid.eel33)*solid.sysT33 | 1 | Elastic strain tensor, ZZ-component | Domain 5 |  |
| solid.eel11 | solid.el11-solid.eiel11 | 1 | Elastic strain tensor, local coordinate system, 11-component | Domain 5 |  |
| solid.eel12 | solid.el12-solid.eiel12 | 1 | Elastic strain tensor, local coordinate system, 12-component | Domain 5 |  |
| solid.eel13 | solid.el13-solid.eiel13 | 1 | Elastic strain tensor, local coordinate system, 13-component | Domain 5 |  |
| solid.eel22 | solid.el22-solid.eiel22 | 1 | Elastic strain tensor, local coordinate system, 22-component | Domain 5 |  |
| solid.eel23 | solid.el23-solid.eiel23 | 1 | Elastic strain tensor, local coordinate system, 23-component | Domain 5 |  |
| solid.eel33 | solid.el33-solid.eiel33 | 1 | Elastic strain tensor, local coordinate system, 33-component | Domain 5 |  |
| solid.eeldev11 | solid.eel11-(solid.eel11+solid.eel22+solid.eel33)/3 | 1 | Deviatoric elastic strain tensor, local coordinate system, 11-component | Domain 5 |  |
| solid.eeldev12 | solid.eel12 | 1 | Deviatoric elastic strain tensor, local coordinate system, 12-component | Domain 5 |  |
| solid.eeldev13 | solid.eel13 | 1 | Deviatoric elastic strain tensor, local coordinate system, 13-component | Domain 5 |  |
| solid.eeldev22 | solid.eel22-(solid.eel11+solid.eel22+solid.eel33)/3 | 1 | Deviatoric elastic strain tensor, local coordinate system, 22-component | Domain 5 |  |
| solid.eeldev23 | solid.eel23 | 1 | Deviatoric elastic strain tensor, local coordinate system, 23-component | Domain 5 |  |
| solid.eeldev33 | solid.eel33-(solid.eel11+solid.eel22+solid.eel33)/3 | 1 | Deviatoric elastic strain tensor, local coordinate system, 33-component | Domain 5 |  |
| solid.eelvol | solid.eel11+solid.eel22+solid.eel33 | 1 | Volumetric elastic strain | Domain 5 |  |
| solid.eil11 | 0 | 1 | Initial strain tensor, local coordinate system, 11-component | Domain 5 | + operation |
| solid.eil12 | 0 | 1 | Initial strain tensor, local coordinate system, 12-component | Domain 5 | + operation |
| solid.eil13 | 0 | 1 | Initial strain tensor, local coordinate system, 13-component | Domain 5 | + operation |
| solid.eil22 | 0 | 1 | Initial strain tensor, local coordinate system, 22-component | Domain 5 | + operation |
| solid.eil23 | 0 | 1 | Initial strain tensor, local coordinate system, 23-component | Domain 5 | + operation |
| solid.eil33 | 0 | 1 | Initial strain tensor, local coordinate system, 33-component | Domain 5 | + operation |
| solid.eiel11 | 0 | 1 | Inelastic strain tensor, local coordinate system, 11-component | Domain 5 | + operation |
| solid.eiel12 | 0 | 1 | Inelastic strain tensor, local coordinate system, 12-component | Domain 5 | + operation |
| solid.eiel13 | 0 | 1 | Inelastic strain tensor, local coordinate system, 13-component | Domain 5 | + operation |
| solid.eiel22 | 0 | 1 | Inelastic strain tensor, local coordinate system, 22-component | Domain 5 | + operation |
| solid.eiel23 | 0 | 1 | Inelastic strain tensor, local coordinate system, 23-component | Domain 5 | + operation |
| solid.eiel33 | 0 | 1 | Inelastic strain tensor, local coordinate system, 33-component | Domain 5 | + operation |
| solid.II2eel | 0.5*(solid.eeldev11^2+2*solid.eeldev12^2+2*solid.eeldev13^2+solid.eeldev22^2+2*solid.eeldev23^2+solid.eeldev33^2) | 1 | Second invariant of deviatoric elastic strain tensor | Domain 5 |  |
| solid.eeGpXX | solid.gpeval(solid.eeXX) | 1 | Elastic strain tensor, XX-component | Domain 5 |  |
| solid.eeGpXY | solid.gpeval(solid.eeXY) | 1 | Elastic strain tensor, XY-component | Domain 5 |  |
| solid.eeGpXZ | solid.gpeval(solid.eeXZ) | 1 | Elastic strain tensor, XZ-component | Domain 5 |  |
| solid.eeGpYY | solid.gpeval(solid.eeYY) | 1 | Elastic strain tensor, YY-component | Domain 5 |  |
| solid.eeGpYZ | solid.gpeval(solid.eeYZ) | 1 | Elastic strain tensor, YZ-component | Domain 5 |  |
| solid.eeGpZZ | solid.gpeval(solid.eeZZ) | 1 | Elastic strain tensor, ZZ-component | Domain 5 |  |
| solid.eelGp11 | solid.gpeval(solid.eel11) | 1 | Elastic strain tensor, local coordinate system, 11-component | Domain 5 |  |
| solid.eelGp12 | solid.gpeval(solid.eel12) | 1 | Elastic strain tensor, local coordinate system, 12-component | Domain 5 |  |
| solid.eelGp13 | solid.gpeval(solid.eel13) | 1 | Elastic strain tensor, local coordinate system, 13-component | Domain 5 |  |
| solid.eelGp22 | solid.gpeval(solid.eel22) | 1 | Elastic strain tensor, local coordinate system, 22-component | Domain 5 |  |
| solid.eelGp23 | solid.gpeval(solid.eel23) | 1 | Elastic strain tensor, local coordinate system, 23-component | Domain 5 |  |
| solid.eelGp33 | solid.gpeval(solid.eel33) | 1 | Elastic strain tensor, local coordinate system, 33-component | Domain 5 |  |
| solid.eeldevGp11 | solid.gpeval(solid.eeldev11) | 1 | Deviatoric elastic strain tensor, local coordinate system, 11-component | Domain 5 |  |
| solid.eeldevGp12 | solid.gpeval(solid.eeldev12) | 1 | Deviatoric elastic strain tensor, local coordinate system, 12-component | Domain 5 |  |
| solid.eeldevGp13 | solid.gpeval(solid.eeldev13) | 1 | Deviatoric elastic strain tensor, local coordinate system, 13-component | Domain 5 |  |
| solid.eeldevGp22 | solid.gpeval(solid.eeldev22) | 1 | Deviatoric elastic strain tensor, local coordinate system, 22-component | Domain 5 |  |
| solid.eeldevGp23 | solid.gpeval(solid.eeldev23) | 1 | Deviatoric elastic strain tensor, local coordinate system, 23-component | Domain 5 |  |
| solid.eeldevGp33 | solid.gpeval(solid.eeldev33) | 1 | Deviatoric elastic strain tensor, local coordinate system, 33-component | Domain 5 |  |
| solid.eelvolGp | solid.gpeval(solid.eelvol) | 1 | Volumetric elastic strain | Domain 5 |  |
| solid.eielGp11 | solid.gpeval(solid.eiel11) | 1 | Inelastic strain tensor, local coordinate system, 11-component | Domain 5 | + operation |
| solid.eielGp12 | solid.gpeval(solid.eiel12) | 1 | Inelastic strain tensor, local coordinate system, 12-component | Domain 5 | + operation |
| solid.eielGp13 | solid.gpeval(solid.eiel13) | 1 | Inelastic strain tensor, local coordinate system, 13-component | Domain 5 | + operation |
| solid.eielGp22 | solid.gpeval(solid.eiel22) | 1 | Inelastic strain tensor, local coordinate system, 22-component | Domain 5 | + operation |
| solid.eielGp23 | solid.gpeval(solid.eiel23) | 1 | Inelastic strain tensor, local coordinate system, 23-component | Domain 5 | + operation |
| solid.eielGp33 | solid.gpeval(solid.eiel33) | 1 | Inelastic strain tensor, local coordinate system, 33-component | Domain 5 | + operation |
| solid.II2eelGp | max(0,solid.gpeval(solid.II2eel)) | 1 | Second invariant of deviatoric elastic strain tensor | Domain 5 |  |
| solid.sxx | solid.SXX | N/m² | Stress tensor, xx-component | Domain 5 |  |
| solid.sxy | solid.SXY | N/m² | Stress tensor, xy-component | Domain 5 |  |
| solid.sxz | solid.SXZ | N/m² | Stress tensor, xz-component | Domain 5 |  |
| solid.syy | solid.SYY | N/m² | Stress tensor, yy-component | Domain 5 |  |
| solid.syz | solid.SYZ | N/m² | Stress tensor, yz-component | Domain 5 |  |
| solid.szz | solid.SZZ | N/m² | Stress tensor, zz-component | Domain 5 |  |
| solid.sl11 | solid.Sl11 | N/m² | Stress tensor, local coordinate system, 11-component | Domain 5 |  |
| solid.sl12 | solid.Sl12 | N/m² | Stress tensor, local coordinate system, 12-component | Domain 5 |  |
| solid.sl13 | solid.Sl13 | N/m² | Stress tensor, local coordinate system, 13-component | Domain 5 |  |
| solid.sl22 | solid.Sl22 | N/m² | Stress tensor, local coordinate system, 22-component | Domain 5 |  |
| solid.sl23 | solid.Sl23 | N/m² | Stress tensor, local coordinate system, 23-component | Domain 5 |  |
| solid.sl33 | solid.Sl33 | N/m² | Stress tensor, local coordinate system, 33-component | Domain 5 |  |
| solid.sdevxx | solid.sxx-(solid.sxx+solid.syy+solid.szz)/3 | N/m² | Deviatoric stress tensor, xx-component | Domain 5 |  |
| solid.sdevxy | solid.sxy | N/m² | Deviatoric stress tensor, xy-component | Domain 5 |  |
| solid.sdevxz | solid.sxz | N/m² | Deviatoric stress tensor, xz-component | Domain 5 |  |
| solid.sdevyy | solid.syy-(solid.sxx+solid.syy+solid.szz)/3 | N/m² | Deviatoric stress tensor, yy-component | Domain 5 |  |
| solid.sdevyz | solid.syz | N/m² | Deviatoric stress tensor, yz-component | Domain 5 |  |
| solid.sdevzz | solid.szz-(solid.sxx+solid.syy+solid.szz)/3 | N/m² | Deviatoric stress tensor, zz-component | Domain 5 |  |
| solid.sldev11 | solid.sl11-(solid.sl11+solid.sl22+solid.sl33)/3 | N/m² | Deviatoric stress tensor, local coordinate system, 11-component | Domain 5 |  |
| solid.sldev12 | solid.sl12 | N/m² | Deviatoric stress tensor, local coordinate system, 12-component | Domain 5 |  |
| solid.sldev13 | solid.sl13 | N/m² | Deviatoric stress tensor, local coordinate system, 13-component | Domain 5 |  |
| solid.sldev22 | solid.sl22-(solid.sl11+solid.sl22+solid.sl33)/3 | N/m² | Deviatoric stress tensor, local coordinate system, 22-component | Domain 5 |  |
| solid.sldev23 | solid.sl23 | N/m² | Deviatoric stress tensor, local coordinate system, 23-component | Domain 5 |  |
| solid.sldev33 | solid.sl33-(solid.sl11+solid.sl22+solid.sl33)/3 | N/m² | Deviatoric stress tensor, local coordinate system, 33-component | Domain 5 |  |
| solid.I1s | solid.sl11+solid.sl22+solid.sl33 | N/m² | First principal invariant of stress | Domain 5 |  |
| solid.I2s | 0.5*(solid.I1s^2-solid.sl11^2-2*solid.sl12^2-2*solid.sl13^2-solid.sl22^2-2*solid.sl23^2-solid.sl33^2) | kg²/(m²·s⁴) | Second principal invariant of stress | Domain 5 |  |
| solid.I3s | solid.sl11*solid.sl22*solid.sl33+2*solid.sl12*solid.sl23*solid.sl13-solid.sl11*solid.sl23^2-solid.sl12^2*solid.sl33-solid.sl22*solid.sl13^2 | kg³/(m³·s⁶) | Third principal invariant of stress | Domain 5 |  |
| solid.II2s | 0.5*(real(solid.sldev11)^2+2*real(solid.sldev12)^2+2*real(solid.sldev13)^2+real(solid.sldev22)^2+2*real(solid.sldev23)^2+real(solid.sldev33)^2) | kg²/(m²·s⁴) | Second invariant of stress deviator | Domain 5 |  |
| solid.II3s | solid.sldev11*solid.sldev22*solid.sldev33+2*solid.sldev12*solid.sldev23*solid.sldev13-solid.sldev11*solid.sldev23^2-solid.sldev12^2*solid.sldev33-solid.sldev22*solid.sldev13^2 | kg³/(m³·s⁶) | Third invariant of stress deviator | Domain 5 |  |
| solid.svol | solid.I1s/3 | N/m² | Hydrostatic stress | Domain 5 |  |
| solid.rdev | sqrteps(2*solid.II2s) | N/m² | Norm of stress deviator | Domain 5 |  |
| solid.thetaL | atan2(sqrt(max(0.14814814814814814*solid.II2s^3-solid.II3s^2,eps)),solid.II3s)/3 | rad | Lode angle | Domain 5 |  |
| solid.Sel11 | solid.Seli11 | N/m² | Elastic second Piola–Kirchhoff stress, local coordinate system, 11-component | Domain 5 |  |
| solid.Sel12 | solid.Seli12 | N/m² | Elastic second Piola–Kirchhoff stress, local coordinate system, 12-component | Domain 5 |  |
| solid.Sel13 | solid.Seli13 | N/m² | Elastic second Piola–Kirchhoff stress, local coordinate system, 13-component | Domain 5 |  |
| solid.Sel22 | solid.Seli22 | N/m² | Elastic second Piola–Kirchhoff stress, local coordinate system, 22-component | Domain 5 |  |
| solid.Sel23 | solid.Seli23 | N/m² | Elastic second Piola–Kirchhoff stress, local coordinate system, 23-component | Domain 5 |  |
| solid.Sel33 | solid.Seli33 | N/m² | Elastic second Piola–Kirchhoff stress, local coordinate system, 33-component | Domain 5 |  |
| solid.Siel11 | solid.Spze11 | N/m² | Inelastic second Piola–Kirchhoff stress tensor, local coordinate system, 11-component | Domain 5 | + operation |
| solid.Siel12 | solid.Spze12 | N/m² | Inelastic second Piola–Kirchhoff stress tensor, local coordinate system, 12-component | Domain 5 | + operation |
| solid.Siel13 | solid.Spze13 | N/m² | Inelastic second Piola–Kirchhoff stress tensor, local coordinate system, 13-component | Domain 5 | + operation |
| solid.Siel22 | solid.Spze22 | N/m² | Inelastic second Piola–Kirchhoff stress tensor, local coordinate system, 22-component | Domain 5 | + operation |
| solid.Siel23 | solid.Spze23 | N/m² | Inelastic second Piola–Kirchhoff stress tensor, local coordinate system, 23-component | Domain 5 | + operation |
| solid.Siel33 | solid.Spze33 | N/m² | Inelastic second Piola–Kirchhoff stress tensor, local coordinate system, 33-component | Domain 5 | + operation |
| solid.SXX | (solid.sysT11*solid.Sl11+solid.sysT21*solid.Sl12+solid.sysT31*solid.Sl13)*solid.sysT11+(solid.sysT11*solid.Sl12+solid.sysT21*solid.Sl22+solid.sysT31*solid.Sl23)*solid.sysT21+(solid.sysT11*solid.Sl13+solid.sysT21*solid.Sl23+solid.sysT31*solid.Sl33)*solid.sysT31 | N/m² | Second Piola–Kirchhoff stress, XX-component | Domain 5 |  |
| solid.SXY | (solid.sysT11*solid.Sl11+solid.sysT21*solid.Sl12+solid.sysT31*solid.Sl13)*solid.sysT12+(solid.sysT11*solid.Sl12+solid.sysT21*solid.Sl22+solid.sysT31*solid.Sl23)*solid.sysT22+(solid.sysT11*solid.Sl13+solid.sysT21*solid.Sl23+solid.sysT31*solid.Sl33)*solid.sysT32 | N/m² | Second Piola–Kirchhoff stress, XY-component | Domain 5 |  |
| solid.SXZ | (solid.sysT11*solid.Sl11+solid.sysT21*solid.Sl12+solid.sysT31*solid.Sl13)*solid.sysT13+(solid.sysT11*solid.Sl12+solid.sysT21*solid.Sl22+solid.sysT31*solid.Sl23)*solid.sysT23+(solid.sysT11*solid.Sl13+solid.sysT21*solid.Sl23+solid.sysT31*solid.Sl33)*solid.sysT33 | N/m² | Second Piola–Kirchhoff stress, XZ-component | Domain 5 |  |
| solid.SYY | (solid.sysT12*solid.Sl11+solid.sysT22*solid.Sl12+solid.sysT32*solid.Sl13)*solid.sysT12+(solid.sysT12*solid.Sl12+solid.sysT22*solid.Sl22+solid.sysT32*solid.Sl23)*solid.sysT22+(solid.sysT12*solid.Sl13+solid.sysT22*solid.Sl23+solid.sysT32*solid.Sl33)*solid.sysT32 | N/m² | Second Piola–Kirchhoff stress, YY-component | Domain 5 |  |
| solid.SYZ | (solid.sysT12*solid.Sl11+solid.sysT22*solid.Sl12+solid.sysT32*solid.Sl13)*solid.sysT13+(solid.sysT12*solid.Sl12+solid.sysT22*solid.Sl22+solid.sysT32*solid.Sl23)*solid.sysT23+(solid.sysT12*solid.Sl13+solid.sysT22*solid.Sl23+solid.sysT32*solid.Sl33)*solid.sysT33 | N/m² | Second Piola–Kirchhoff stress, YZ-component | Domain 5 |  |
| solid.SZZ | (solid.sysT13*solid.Sl11+solid.sysT23*solid.Sl12+solid.sysT33*solid.Sl13)*solid.sysT13+(solid.sysT13*solid.Sl12+solid.sysT23*solid.Sl22+solid.sysT33*solid.Sl23)*solid.sysT23+(solid.sysT13*solid.Sl13+solid.sysT23*solid.Sl23+solid.sysT33*solid.Sl33)*solid.sysT33 | N/m² | Second Piola–Kirchhoff stress, ZZ-component | Domain 5 |  |
| solid.Sl11 | solid.Siel11+solid.Sel11 | N/m² | Second Piola–Kirchhoff stress, local coordinate system, 11-component | Domain 5 | + operation |
| solid.Sl12 | solid.Siel12+solid.Sel12 | N/m² | Second Piola–Kirchhoff stress, local coordinate system, 12-component | Domain 5 | + operation |
| solid.Sl13 | solid.Siel13+solid.Sel13 | N/m² | Second Piola–Kirchhoff stress, local coordinate system, 13-component | Domain 5 | + operation |
| solid.Sl22 | solid.Siel22+solid.Sel22 | N/m² | Second Piola–Kirchhoff stress, local coordinate system, 22-component | Domain 5 | + operation |
| solid.Sl23 | solid.Siel23+solid.Sel23 | N/m² | Second Piola–Kirchhoff stress, local coordinate system, 23-component | Domain 5 | + operation |
| solid.Sl33 | solid.Siel33+solid.Sel33 | N/m² | Second Piola–Kirchhoff stress, local coordinate system, 33-component | Domain 5 | + operation |
| solid.SdevXX | solid.SXX-(solid.SXX+solid.SYY+solid.SZZ)/3 | N/m² | Deviatoric second Piola–Kirchhoff stress, XX-component | Domain 5 |  |
| solid.SdevXY | solid.SXY | N/m² | Deviatoric second Piola–Kirchhoff stress, XY-component | Domain 5 |  |
| solid.SdevXZ | solid.SXZ | N/m² | Deviatoric second Piola–Kirchhoff stress, XZ-component | Domain 5 |  |
| solid.SdevYY | solid.SYY-(solid.SXX+solid.SYY+solid.SZZ)/3 | N/m² | Deviatoric second Piola–Kirchhoff stress, YY-component | Domain 5 |  |
| solid.SdevYZ | solid.SYZ | N/m² | Deviatoric second Piola–Kirchhoff stress, YZ-component | Domain 5 |  |
| solid.SdevZZ | solid.SZZ-(solid.SXX+solid.SYY+solid.SZZ)/3 | N/m² | Deviatoric second Piola–Kirchhoff stress, ZZ-component | Domain 5 |  |
| solid.Sldev11 | solid.Sl11-(solid.Sl11+solid.Sl22+solid.Sl33)/3 | N/m² | Deviatoric second Piola–Kirchhoff stress, local coordinate system, 11-component | Domain 5 |  |
| solid.Sldev12 | solid.Sl12 | N/m² | Deviatoric second Piola–Kirchhoff stress, local coordinate system, 12-component | Domain 5 |  |
| solid.Sldev13 | solid.Sl13 | N/m² | Deviatoric second Piola–Kirchhoff stress, local coordinate system, 13-component | Domain 5 |  |
| solid.Sldev22 | solid.Sl22-(solid.Sl11+solid.Sl22+solid.Sl33)/3 | N/m² | Deviatoric second Piola–Kirchhoff stress, local coordinate system, 22-component | Domain 5 |  |
| solid.Sldev23 | solid.Sl23 | N/m² | Deviatoric second Piola–Kirchhoff stress, local coordinate system, 23-component | Domain 5 |  |
| solid.Sldev33 | solid.Sl33-(solid.Sl11+solid.Sl22+solid.Sl33)/3 | N/m² | Deviatoric second Piola–Kirchhoff stress, local coordinate system, 33-component | Domain 5 |  |
| solid.PxX | solid.SXX | N/m² | First Piola–Kirchhoff stress, xX-component | Domain 5 |  |
| solid.PyX | solid.SXY | N/m² | First Piola–Kirchhoff stress, yX-component | Domain 5 |  |
| solid.PzX | solid.SXZ | N/m² | First Piola–Kirchhoff stress, zX-component | Domain 5 |  |
| solid.PxY | solid.SXY | N/m² | First Piola–Kirchhoff stress, xY-component | Domain 5 |  |
| solid.PyY | solid.SYY | N/m² | First Piola–Kirchhoff stress, yY-component | Domain 5 |  |
| solid.PzY | solid.SYZ | N/m² | First Piola–Kirchhoff stress, zY-component | Domain 5 |  |
| solid.PxZ | solid.SXZ | N/m² | First Piola–Kirchhoff stress, xZ-component | Domain 5 |  |
| solid.PyZ | solid.SYZ | N/m² | First Piola–Kirchhoff stress, yZ-component | Domain 5 |  |
| solid.PzZ | solid.SZZ | N/m² | First Piola–Kirchhoff stress, zZ-component | Domain 5 |  |
| solid.Spze11 | -solid.eES11*(comp1_xz_sys.T11*solid.EpzeX+comp1_xz_sys.T12*solid.EpzeY+comp1_xz_sys.T13*solid.EpzeZ)-solid.eES21*(comp1_xz_sys.T21*solid.EpzeX+comp1_xz_sys.T22*solid.EpzeY+comp1_xz_sys.T23*solid.EpzeZ)-solid.eES31*(comp1_xz_sys.T31*solid.EpzeX+comp1_xz_sys.T32*solid.EpzeY+comp1_xz_sys.T33*solid.EpzeZ) | N/m² | Piezoelectric stress tensor, local coordinate system, 11-component | Domain 5 |  |
| solid.Spze12 | -solid.eES16*(comp1_xz_sys.T11*solid.EpzeX+comp1_xz_sys.T12*solid.EpzeY+comp1_xz_sys.T13*solid.EpzeZ)-solid.eES26*(comp1_xz_sys.T21*solid.EpzeX+comp1_xz_sys.T22*solid.EpzeY+comp1_xz_sys.T23*solid.EpzeZ)-solid.eES36*(comp1_xz_sys.T31*solid.EpzeX+comp1_xz_sys.T32*solid.EpzeY+comp1_xz_sys.T33*solid.EpzeZ) | N/m² | Piezoelectric stress tensor, local coordinate system, 12-component | Domain 5 |  |
| solid.Spze13 | -solid.eES15*(comp1_xz_sys.T11*solid.EpzeX+comp1_xz_sys.T12*solid.EpzeY+comp1_xz_sys.T13*solid.EpzeZ)-solid.eES25*(comp1_xz_sys.T21*solid.EpzeX+comp1_xz_sys.T22*solid.EpzeY+comp1_xz_sys.T23*solid.EpzeZ)-solid.eES35*(comp1_xz_sys.T31*solid.EpzeX+comp1_xz_sys.T32*solid.EpzeY+comp1_xz_sys.T33*solid.EpzeZ) | N/m² | Piezoelectric stress tensor, local coordinate system, 13-component | Domain 5 |  |
| solid.Spze22 | -solid.eES12*(comp1_xz_sys.T11*solid.EpzeX+comp1_xz_sys.T12*solid.EpzeY+comp1_xz_sys.T13*solid.EpzeZ)-solid.eES22*(comp1_xz_sys.T21*solid.EpzeX+comp1_xz_sys.T22*solid.EpzeY+comp1_xz_sys.T23*solid.EpzeZ)-solid.eES32*(comp1_xz_sys.T31*solid.EpzeX+comp1_xz_sys.T32*solid.EpzeY+comp1_xz_sys.T33*solid.EpzeZ) | N/m² | Piezoelectric stress tensor, local coordinate system, 22-component | Domain 5 |  |
| solid.Spze23 | -solid.eES14*(comp1_xz_sys.T11*solid.EpzeX+comp1_xz_sys.T12*solid.EpzeY+comp1_xz_sys.T13*solid.EpzeZ)-solid.eES24*(comp1_xz_sys.T21*solid.EpzeX+comp1_xz_sys.T22*solid.EpzeY+comp1_xz_sys.T23*solid.EpzeZ)-solid.eES34*(comp1_xz_sys.T31*solid.EpzeX+comp1_xz_sys.T32*solid.EpzeY+comp1_xz_sys.T33*solid.EpzeZ) | N/m² | Piezoelectric stress tensor, local coordinate system, 23-component | Domain 5 |  |
| solid.Spze33 | -solid.eES13*(comp1_xz_sys.T11*solid.EpzeX+comp1_xz_sys.T12*solid.EpzeY+comp1_xz_sys.T13*solid.EpzeZ)-solid.eES23*(comp1_xz_sys.T21*solid.EpzeX+comp1_xz_sys.T22*solid.EpzeY+comp1_xz_sys.T23*solid.EpzeZ)-solid.eES33*(comp1_xz_sys.T31*solid.EpzeX+comp1_xz_sys.T32*solid.EpzeY+comp1_xz_sys.T33*solid.EpzeZ) | N/m² | Piezoelectric stress tensor, local coordinate system, 33-component | Domain 5 |  |
| solid.Sil11 | 0 | N/m² | Initial stress tensor, local coordinate system, 11-component | Domain 5 | + operation |
| solid.Sil12 | 0 | N/m² | Initial stress tensor, local coordinate system, 12-component | Domain 5 | + operation |
| solid.Sil13 | 0 | N/m² | Initial stress tensor, local coordinate system, 13-component | Domain 5 | + operation |
| solid.Sil22 | 0 | N/m² | Initial stress tensor, local coordinate system, 22-component | Domain 5 | + operation |
| solid.Sil23 | 0 | N/m² | Initial stress tensor, local coordinate system, 23-component | Domain 5 | + operation |
| solid.Sil33 | 0 | N/m² | Initial stress tensor, local coordinate system, 33-component | Domain 5 | + operation |
| solid.mises_peak | sqrteps(solid.pzm1.cvector1+solid.pzm1.cvector2+solid.pzm1.cvector3+solid.pzm1.cvector4+solid.pzm1.cvector5+solid.pzm1.cvector6+solid.pzm1.cvector7+solid.pzm1.cvector8+solid.pzm1.cvector9+sqrteps(solid.pzm1.avector1^2+solid.pzm1.avector1*solid.pzm1.avector2*cos(solid.pzm1.bvector1-solid.pzm1.bvector2)+solid.pzm1.avector1*solid.pzm1.avector3*cos(solid.pzm1.bvector1-solid.pzm1.bvector3)+solid.pzm1.avector1*solid.pzm1.avector4*cos(solid.pzm1.bvector1-solid.pzm1.bvector4)+solid.pzm1.avector1*solid.pzm1.avector5*cos(solid.pzm1.bvector1-solid.pzm1.bvector5)+solid.pzm1.avector1*solid.pzm1.avector6*cos(solid.pzm1.bvector1-solid.pzm1.bvector6)+solid.pzm1.avector1*solid.pzm1.avector7*cos(solid.pzm1.bvector1-solid.pzm1.bvector7)+solid.pzm1.avector1*solid.pzm1.avector8*cos(solid.pzm1.bvector1-solid.pzm1.bvector8)+solid.pzm1.avector1*solid.pzm1.avector9*cos(solid.pzm1.bvector1-solid.pzm1.bvector9)+solid.pzm1.avector2*solid.pzm1.avector1*cos(solid.pzm1.bvector2-solid.pzm1.bvector1)+solid.pzm1.avector2^2+solid.pzm1.avector2*solid.pzm1.avector3*cos(solid.pzm1.bvector2-solid.pzm1.bvector3)+solid.pzm1.avector2*solid.pzm1.avector4*cos(solid.pzm1.bvector2-solid.pzm1.bvector4)+solid.pzm1.avector2*solid.pzm1.avector5*cos(solid.pzm1.bvector2-solid.pzm1.bvector5)+solid.pzm1.avector2*solid.pzm1.avector6*cos(solid.pzm1.bvector2-solid.pzm1.bvector6)+solid.pzm1.avector2*solid.pzm1.avector7*cos(solid.pzm1.bvector2-solid.pzm1.bvector7)+solid.pzm1.avector2*solid.pzm1.avector8*cos(solid.pzm1.bvector2-solid.pzm1.bvector8)+solid.pzm1.avector2*solid.pzm1.avector9*cos(solid.pzm1.bvector2-solid.pzm1.bvector9)+solid.pzm1.avector3*solid.pzm1.avector1*cos(solid.pzm1.bvector3-solid.pzm1.bvector1)+solid.pzm1.avector3*solid.pzm1.avector2*cos(solid.pzm1.bvector3-solid.pzm1.bvector2)+solid.pzm1.avector3^2+solid.pzm1.avector3*solid.pzm1.avector4*cos(solid.pzm1.bvector3-solid.pzm1.bvector4)+solid.pzm1.avector3*solid.pzm1.avector5*cos(solid.pzm1.bvector3-solid.pzm1.bvector5)+solid.pzm1.avector3*solid.pzm1.avector6*cos(solid.pzm1.bvector3-solid.pzm1.bvector6)+solid.pzm1.avector3*solid.pzm1.avector7*cos(solid.pzm1.bvector3-solid.pzm1.bvector7)+solid.pzm1.avector3*solid.pzm1.avector8*cos(solid.pzm1.bvector3-solid.pzm1.bvector8)+solid.pzm1.avector3*solid.pzm1.avector9*cos(solid.pzm1.bvector3-solid.pzm1.bvector9)+solid.pzm1.avector4*solid.pzm1.avector1*cos(solid.pzm1.bvector4-solid.pzm1.bvector1)+solid.pzm1.avector4*solid.pzm1.avector2*cos(solid.pzm1.bvector4-solid.pzm1.bvector2)+solid.pzm1.avector4*solid.pzm1.avector3*cos(solid.pzm1.bvector4-solid.pzm1.bvector3)+solid.pzm1.avector4^2+solid.pzm1.avector4*solid.pzm1.avector5*cos(solid.pzm1.bvector4-solid.pzm1.bvector5)+solid.pzm1.avector4*solid.pzm1.avector6*cos(solid.pzm1.bvector4-solid.pzm1.bvector6)+solid.pzm1.avector4*solid.pzm1.avector7*cos(solid.pzm1.bvector4-solid.pzm1.bvector7)+solid.pzm1.avector4*solid.pzm1.avector8*cos(solid.pzm1.bvector4-solid.pzm1.bvector8)+solid.pzm1.avector4*solid.pzm1.avector9*cos(solid.pzm1.bvector4-solid.pzm1.bvector9)+solid.pzm1.avector5*solid.pzm1.avector1*cos(solid.pzm1.bvector5-solid.pzm1.bvector1)+solid.pzm1.avector5*solid.pzm1.avector2*cos(solid.pzm1.bvector5-solid.pzm1.bvector2)+solid.pzm1.avector5*solid.pzm1.avector3*cos(solid.pzm1.bvector5-solid.pzm1.bvector3)+solid.pzm1.avector5*solid.pzm1.avector4*cos(solid.pzm1.bvector5-solid.pzm1.bvector4)+solid.pzm1.avector5^2+solid.pzm1.avector5*solid.pzm1.avector6*cos(solid.pzm1.bvector5-solid.pzm1.bvector6)+solid.pzm1.avector5*solid.pzm1.avector7*cos(solid.pzm1.bvector5-solid.pzm1.bvector7)+solid.pzm1.avector5*solid.pzm1.avector8*cos(solid.pzm1.bvector5-solid.pzm1.bvector8)+solid.pzm1.avector5*solid.pzm1.avector9*cos(solid.pzm1.bvector5-solid.pzm1.bvector9)+solid.pzm1.avector6*solid.pzm1.avector1*cos(solid.pzm1.bvector6-solid.pzm1.bvector1)+solid.pzm1.avector6*solid.pzm1.avector2*cos(solid.pzm1.bvector6-solid.pzm1.bvector2)+solid.pzm1.avector6*solid.pzm1.avector3*cos(solid.pzm1.bvector6-solid.pzm1.bvector3)+solid.pzm1.avector6*solid.pzm1.avector4*cos(solid.pzm1.bvector6-solid.pzm1.bvector4)+solid.pzm1.avector6*solid.pzm1.avector5*cos(solid.pzm1.bvector6-solid.pzm1.bvector5)+solid.pzm1.avector6^2+solid.pzm1.avector6*solid.pzm1.avector7*cos(solid.pzm1.bvector6-solid.pzm1.bvector7)+solid.pzm1.avector6*solid.pzm1.avector8*cos(solid.pzm1.bvector6-solid.pzm1.bvector8)+solid.pzm1.avector6*solid.pzm1.avector9*cos(solid.pzm1.bvector6-solid.pzm1.bvector9)+solid.pzm1.avector7*solid.pzm1.avector1*cos(solid.pzm1.bvector7-solid.pzm1.bvector1)+solid.pzm1.avector7*solid.pzm1.avector2*cos(solid.pzm1.bvector7-solid.pzm1.bvector2)+solid.pzm1.avector7*solid.pzm1.avector3*cos(solid.pzm1.bvector7-solid.pzm1.bvector3)+solid.pzm1.avector7*solid.pzm1.avector4*cos(solid.pzm1.bvector7-solid.pzm1.bvector4)+solid.pzm1.avector7*solid.pzm1.avector5*cos(solid.pzm1.bvector7-solid.pzm1.bvector5)+solid.pzm1.avector7*solid.pzm1.avector6*cos(solid.pzm1.bvector7-solid.pzm1.bvector6)+solid.pzm1.avector7^2+solid.pzm1.avector7*solid.pzm1.avector8*cos(solid.pzm1.bvector7-solid.pzm1.bvector8)+solid.pzm1.avector7*solid.pzm1.avector9*cos(solid.pzm1.bvector7-solid.pzm1.bvector9)+solid.pzm1.avector8*solid.pzm1.avector1*cos(solid.pzm1.bvector8-solid.pzm1.bvector1)+solid.pzm1.avector8*solid.pzm1.avector2*cos(solid.pzm1.bvector8-solid.pzm1.bvector2)+solid.pzm1.avector8*solid.pzm1.avector3*cos(solid.pzm1.bvector8-solid.pzm1.bvector3)+solid.pzm1.avector8*solid.pzm1.avector4*cos(solid.pzm1.bvector8-solid.pzm1.bvector4)+solid.pzm1.avector8*solid.pzm1.avector5*cos(solid.pzm1.bvector8-solid.pzm1.bvector5)+solid.pzm1.avector8*solid.pzm1.avector6*cos(solid.pzm1.bvector8-solid.pzm1.bvector6)+solid.pzm1.avector8*solid.pzm1.avector7*cos(solid.pzm1.bvector8-solid.pzm1.bvector7)+solid.pzm1.avector8^2+solid.pzm1.avector8*solid.pzm1.avector9*cos(solid.pzm1.bvector8-solid.pzm1.bvector9)+solid.pzm1.avector9*solid.pzm1.avector1*cos(solid.pzm1.bvector9-solid.pzm1.bvector1)+solid.pzm1.avector9*solid.pzm1.avector2*cos(solid.pzm1.bvector9-solid.pzm1.bvector2)+solid.pzm1.avector9*solid.pzm1.avector3*cos(solid.pzm1.bvector9-solid.pzm1.bvector3)+solid.pzm1.avector9*solid.pzm1.avector4*cos(solid.pzm1.bvector9-solid.pzm1.bvector4)+solid.pzm1.avector9*solid.pzm1.avector5*cos(solid.pzm1.bvector9-solid.pzm1.bvector5)+solid.pzm1.avector9*solid.pzm1.avector6*cos(solid.pzm1.bvector9-solid.pzm1.bvector6)+solid.pzm1.avector9*solid.pzm1.avector7*cos(solid.pzm1.bvector9-solid.pzm1.bvector7)+solid.pzm1.avector9*solid.pzm1.avector8*cos(solid.pzm1.bvector9-solid.pzm1.bvector8)+solid.pzm1.avector9^2)) | N/m² | Von Mises stress, peak | Domain 5 |  |
| solid.tresca | max(max(abs(solid.sp1-solid.sp2),abs(solid.sp1-solid.sp3)),abs(solid.sp2-solid.sp3)) | N/m² | Tresca stress | Domain 5 |  |
| solid.mises | sqrteps(3*solid.II2s) | N/m² | von Mises stress | Domain 5 |  |
| solid.pm | -(solid.sl11+solid.sl22+solid.sl33)/3 | N/m² | Pressure | Domain 5 |  |
| solid.Seli11 | solid.D11*solid.eel11+solid.D12*solid.eel22+solid.D13*solid.eel33+2*solid.D14*solid.eel12+2*solid.D15*solid.eel23+2*solid.D16*solid.eel13 | N/m² | Elastic second Piola–Kirchhoff stress, intermediate configuration, 11-component | Domain 5 |  |
| solid.Seli12 | solid.D14*solid.eel11+solid.D24*solid.eel22+solid.D34*solid.eel33+2*solid.D44*solid.eel12+2*solid.D45*solid.eel23+2*solid.D46*solid.eel13 | N/m² | Elastic second Piola–Kirchhoff stress, intermediate configuration, 12-component | Domain 5 |  |
| solid.Seli13 | solid.D16*solid.eel11+solid.D26*solid.eel22+solid.D36*solid.eel33+2*solid.D46*solid.eel12+2*solid.D56*solid.eel23+2*solid.D66*solid.eel13 | N/m² | Elastic second Piola–Kirchhoff stress, intermediate configuration, 13-component | Domain 5 |  |
| solid.Seli22 | solid.D12*solid.eel11+solid.D22*solid.eel22+solid.D23*solid.eel33+2*solid.D24*solid.eel12+2*solid.D25*solid.eel23+2*solid.D26*solid.eel13 | N/m² | Elastic second Piola–Kirchhoff stress, intermediate configuration, 22-component | Domain 5 |  |
| solid.Seli23 | solid.D15*solid.eel11+solid.D25*solid.eel22+solid.D35*solid.eel33+2*solid.D45*solid.eel12+2*solid.D55*solid.eel23+2*solid.D56*solid.eel13 | N/m² | Elastic second Piola–Kirchhoff stress, intermediate configuration, 23-component | Domain 5 |  |
| solid.Seli33 | solid.D13*solid.eel11+solid.D23*solid.eel22+solid.D33*solid.eel33+2*solid.D34*solid.eel12+2*solid.D35*solid.eel23+2*solid.D36*solid.eel13 | N/m² | Elastic second Piola–Kirchhoff stress, intermediate configuration, 33-component | Domain 5 |  |
| solid.Sieli11 | solid.Siel11 | N/m² | Inelastic second Piola–Kirchhoff stress tensor, intermediate configuration, 11-component | Domain 5 |  |
| solid.Sieli12 | solid.Siel12 | N/m² | Inelastic second Piola–Kirchhoff stress tensor, intermediate configuration, 12-component | Domain 5 |  |
| solid.Sieli13 | solid.Siel13 | N/m² | Inelastic second Piola–Kirchhoff stress tensor, intermediate configuration, 13-component | Domain 5 |  |
| solid.Sieli22 | solid.Siel22 | N/m² | Inelastic second Piola–Kirchhoff stress tensor, intermediate configuration, 22-component | Domain 5 |  |
| solid.Sieli23 | solid.Siel23 | N/m² | Inelastic second Piola–Kirchhoff stress tensor, intermediate configuration, 23-component | Domain 5 |  |
| solid.Sieli33 | solid.Siel33 | N/m² | Inelastic second Piola–Kirchhoff stress tensor, intermediate configuration, 33-component | Domain 5 |  |
| solid.Mandel11 | solid.Sel11 | N/m² | Mandel stress, local coordinate system, 11-component | Domain 5 |  |
| solid.Mandel21 | solid.Sel12 | N/m² | Mandel stress, local coordinate system, 21-component | Domain 5 |  |
| solid.Mandel31 | solid.Sel13 | N/m² | Mandel stress, local coordinate system, 31-component | Domain 5 |  |
| solid.Mandel12 | solid.Sel12 | N/m² | Mandel stress, local coordinate system, 12-component | Domain 5 |  |
| solid.Mandel22 | solid.Sel22 | N/m² | Mandel stress, local coordinate system, 22-component | Domain 5 |  |
| solid.Mandel32 | solid.Sel23 | N/m² | Mandel stress, local coordinate system, 32-component | Domain 5 |  |
| solid.Mandel13 | solid.Sel13 | N/m² | Mandel stress, local coordinate system, 13-component | Domain 5 |  |
| solid.Mandel23 | solid.Sel23 | N/m² | Mandel stress, local coordinate system, 23-component | Domain 5 |  |
| solid.Mandel33 | solid.Sel33 | N/m² | Mandel stress, local coordinate system, 33-component | Domain 5 |  |
| solid.sGpxx | solid.gpeval(solid.sxx) | N/m² | Stress tensor, xx-component | Domain 5 |  |
| solid.sGpxy | solid.gpeval(solid.sxy) | N/m² | Stress tensor, xy-component | Domain 5 |  |
| solid.sGpxz | solid.gpeval(solid.sxz) | N/m² | Stress tensor, xz-component | Domain 5 |  |
| solid.sGpyy | solid.gpeval(solid.syy) | N/m² | Stress tensor, yy-component | Domain 5 |  |
| solid.sGpyz | solid.gpeval(solid.syz) | N/m² | Stress tensor, yz-component | Domain 5 |  |
| solid.sGpzz | solid.gpeval(solid.szz) | N/m² | Stress tensor, zz-component | Domain 5 |  |
| solid.slGp11 | solid.gpeval(solid.sl11) | N/m² | Stress tensor, local coordinate system, 11-component | Domain 5 |  |
| solid.slGp12 | solid.gpeval(solid.sl12) | N/m² | Stress tensor, local coordinate system, 12-component | Domain 5 |  |
| solid.slGp13 | solid.gpeval(solid.sl13) | N/m² | Stress tensor, local coordinate system, 13-component | Domain 5 |  |
| solid.slGp22 | solid.gpeval(solid.sl22) | N/m² | Stress tensor, local coordinate system, 22-component | Domain 5 |  |
| solid.slGp23 | solid.gpeval(solid.sl23) | N/m² | Stress tensor, local coordinate system, 23-component | Domain 5 |  |
| solid.slGp33 | solid.gpeval(solid.sl33) | N/m² | Stress tensor, local coordinate system, 33-component | Domain 5 |  |
| solid.sdevGpxx | solid.gpeval(solid.sdevxx) | N/m² | Deviatoric stress tensor, xx-component | Domain 5 |  |
| solid.sdevGpxy | solid.gpeval(solid.sdevxy) | N/m² | Deviatoric stress tensor, xy-component | Domain 5 |  |
| solid.sdevGpxz | solid.gpeval(solid.sdevxz) | N/m² | Deviatoric stress tensor, xz-component | Domain 5 |  |
| solid.sdevGpyy | solid.gpeval(solid.sdevyy) | N/m² | Deviatoric stress tensor, yy-component | Domain 5 |  |
| solid.sdevGpyz | solid.gpeval(solid.sdevyz) | N/m² | Deviatoric stress tensor, yz-component | Domain 5 |  |
| solid.sdevGpzz | solid.gpeval(solid.sdevzz) | N/m² | Deviatoric stress tensor, zz-component | Domain 5 |  |
| solid.sldevGp11 | solid.gpeval(solid.sldev11) | N/m² | Deviatoric stress tensor, 11-component | Domain 5 |  |
| solid.sldevGp12 | solid.gpeval(solid.sldev12) | N/m² | Deviatoric stress tensor, 12-component | Domain 5 |  |
| solid.sldevGp13 | solid.gpeval(solid.sldev13) | N/m² | Deviatoric stress tensor, 13-component | Domain 5 |  |
| solid.sldevGp22 | solid.gpeval(solid.sldev22) | N/m² | Deviatoric stress tensor, 22-component | Domain 5 |  |
| solid.sldevGp23 | solid.gpeval(solid.sldev23) | N/m² | Deviatoric stress tensor, 23-component | Domain 5 |  |
| solid.sldevGp33 | solid.gpeval(solid.sldev33) | N/m² | Deviatoric stress tensor, 33-component | Domain 5 |  |
| solid.I1sGp | solid.gpeval(solid.I1s) | N/m² | First principal invariant of stress | Domain 5 |  |
| solid.I2sGp | max(0,solid.gpeval(solid.I2s)) | kg²/(m²·s⁴) | Second principal invariant of stress | Domain 5 |  |
| solid.I3sGp | solid.gpeval(solid.I3s) | kg³/(m³·s⁶) | Third principal invariant of stress | Domain 5 |  |
| solid.II3sGp | solid.gpeval(solid.II3s) | kg³/(m³·s⁶) | Third invariant of stress deviator | Domain 5 |  |
| solid.svolGp | solid.gpeval(solid.svol) | N/m² | Hydrostatic stress | Domain 5 |  |
| solid.rdevGp | max(0,solid.gpeval(solid.rdev)) | N/m² | Norm of stress deviator | Domain 5 |  |
| solid.thetaLGp | solid.gpeval(solid.thetaL) | rad | Lode angle | Domain 5 |  |
| solid.SGpXX | solid.gpeval(solid.SXX) | N/m² | Second Piola–Kirchhoff stress, XX-component | Domain 5 |  |
| solid.SGpXY | solid.gpeval(solid.SXY) | N/m² | Second Piola–Kirchhoff stress, XY-component | Domain 5 |  |
| solid.SGpXZ | solid.gpeval(solid.SXZ) | N/m² | Second Piola–Kirchhoff stress, XZ-component | Domain 5 |  |
| solid.SGpYY | solid.gpeval(solid.SYY) | N/m² | Second Piola–Kirchhoff stress, YY-component | Domain 5 |  |
| solid.SGpYZ | solid.gpeval(solid.SYZ) | N/m² | Second Piola–Kirchhoff stress, YZ-component | Domain 5 |  |
| solid.SGpZZ | solid.gpeval(solid.SZZ) | N/m² | Second Piola–Kirchhoff stress, ZZ-component | Domain 5 |  |
| solid.SlGp11 | solid.gpeval(solid.Sl11) | N/m² | Second Piola–Kirchhoff stress, local coordinate system, 11-component | Domain 5 |  |
| solid.SlGp12 | solid.gpeval(solid.Sl12) | N/m² | Second Piola–Kirchhoff stress, local coordinate system, 12-component | Domain 5 |  |
| solid.SlGp13 | solid.gpeval(solid.Sl13) | N/m² | Second Piola–Kirchhoff stress, local coordinate system, 13-component | Domain 5 |  |
| solid.SlGp22 | solid.gpeval(solid.Sl22) | N/m² | Second Piola–Kirchhoff stress, local coordinate system, 22-component | Domain 5 |  |
| solid.SlGp23 | solid.gpeval(solid.Sl23) | N/m² | Second Piola–Kirchhoff stress, local coordinate system, 23-component | Domain 5 |  |
| solid.SlGp33 | solid.gpeval(solid.Sl33) | N/m² | Second Piola–Kirchhoff stress, local coordinate system, 33-component | Domain 5 |  |
| solid.SdevGpXX | solid.gpeval(solid.SdevXX) | N/m² | Deviatoric second Piola–Kirchhoff stress, XX-component | Domain 5 |  |
| solid.SdevGpXY | solid.gpeval(solid.SdevXY) | N/m² | Deviatoric second Piola–Kirchhoff stress, XY-component | Domain 5 |  |
| solid.SdevGpXZ | solid.gpeval(solid.SdevXZ) | N/m² | Deviatoric second Piola–Kirchhoff stress, XZ-component | Domain 5 |  |
| solid.SdevGpYY | solid.gpeval(solid.SdevYY) | N/m² | Deviatoric second Piola–Kirchhoff stress, YY-component | Domain 5 |  |
| solid.SdevGpYZ | solid.gpeval(solid.SdevYZ) | N/m² | Deviatoric second Piola–Kirchhoff stress, YZ-component | Domain 5 |  |
| solid.SdevGpZZ | solid.gpeval(solid.SdevZZ) | N/m² | Deviatoric second Piola–Kirchhoff stress, ZZ-component | Domain 5 |  |
| solid.SldevGp11 | solid.gpeval(solid.Sldev11) | N/m² | Deviatoric second Piola–Kirchhoff stress, local coordinate system, 11-component | Domain 5 |  |
| solid.SldevGp12 | solid.gpeval(solid.Sldev12) | N/m² | Deviatoric second Piola–Kirchhoff stress, local coordinate system, 12-component | Domain 5 |  |
| solid.SldevGp13 | solid.gpeval(solid.Sldev13) | N/m² | Deviatoric second Piola–Kirchhoff stress, local coordinate system, 13-component | Domain 5 |  |
| solid.SldevGp22 | solid.gpeval(solid.Sldev22) | N/m² | Deviatoric second Piola–Kirchhoff stress, local coordinate system, 22-component | Domain 5 |  |
| solid.SldevGp23 | solid.gpeval(solid.Sldev23) | N/m² | Deviatoric second Piola–Kirchhoff stress, local coordinate system, 23-component | Domain 5 |  |
| solid.SldevGp33 | solid.gpeval(solid.Sldev33) | N/m² | Deviatoric second Piola–Kirchhoff stress, local coordinate system, 33-component | Domain 5 |  |
| solid.PGpxX | solid.gpeval(solid.PxX) | N/m² | First Piola–Kirchhoff stress, xX-component | Domain 5 |  |
| solid.PGpyX | solid.gpeval(solid.PyX) | N/m² | First Piola–Kirchhoff stress, yX-component | Domain 5 |  |
| solid.PGpzX | solid.gpeval(solid.PzX) | N/m² | First Piola–Kirchhoff stress, zX-component | Domain 5 |  |
| solid.PGpxY | solid.gpeval(solid.PxY) | N/m² | First Piola–Kirchhoff stress, xY-component | Domain 5 |  |
| solid.PGpyY | solid.gpeval(solid.PyY) | N/m² | First Piola–Kirchhoff stress, yY-component | Domain 5 |  |
| solid.PGpzY | solid.gpeval(solid.PzY) | N/m² | First Piola–Kirchhoff stress, zY-component | Domain 5 |  |
| solid.PGpxZ | solid.gpeval(solid.PxZ) | N/m² | First Piola–Kirchhoff stress, xZ-component | Domain 5 |  |
| solid.PGpyZ | solid.gpeval(solid.PyZ) | N/m² | First Piola–Kirchhoff stress, yZ-component | Domain 5 |  |
| solid.PGpzZ | solid.gpeval(solid.PzZ) | N/m² | First Piola–Kirchhoff stress, zZ-component | Domain 5 |  |
| solid.PlGpx1 | solid.gpeval(solid.Plx1) | N/m² | First Piola–Kirchhoff stress, local coordinate system, x1-component | Domain 5 |  |
| solid.PlGpy1 | solid.gpeval(solid.Ply1) | N/m² | First Piola–Kirchhoff stress, local coordinate system, y1-component | Domain 5 |  |
| solid.PlGpz1 | solid.gpeval(solid.Plz1) | N/m² | First Piola–Kirchhoff stress, local coordinate system, z1-component | Domain 5 |  |
| solid.PlGpx2 | solid.gpeval(solid.Plx2) | N/m² | First Piola–Kirchhoff stress, local coordinate system, x2-component | Domain 5 |  |
| solid.PlGpy2 | solid.gpeval(solid.Ply2) | N/m² | First Piola–Kirchhoff stress, local coordinate system, y2-component | Domain 5 |  |
| solid.PlGpz2 | solid.gpeval(solid.Plz2) | N/m² | First Piola–Kirchhoff stress, local coordinate system, z2-component | Domain 5 |  |
| solid.PlGpx3 | solid.gpeval(solid.Plx3) | N/m² | First Piola–Kirchhoff stress, local coordinate system, x3-component | Domain 5 |  |
| solid.PlGpy3 | solid.gpeval(solid.Ply3) | N/m² | First Piola–Kirchhoff stress, local coordinate system, y3-component | Domain 5 |  |
| solid.PlGpz3 | solid.gpeval(solid.Plz3) | N/m² | First Piola–Kirchhoff stress, local coordinate system, z3-component | Domain 5 |  |
| solid.misesGp | max(0,solid.gpeval(solid.mises)) | N/m² | von Mises stress | Domain 5 |  |
| solid.misesGp_peak | max(0,solid.gpeval(solid.mises_peak)) | N/m² | Von Mises stress, peak | Domain 5 |  |
| solid.trescaGp | max(0,solid.gpeval(solid.tresca)) | N/m² | Tresca stress | Domain 5 |  |
| solid.pmGp | solid.gpeval(solid.pm) | N/m² | Pressure | Domain 5 |  |
| solid.Ws | 0.5*solid.Ji*(solid.Sel11*solid.eel11+2*solid.Sel12*solid.eel12+2*solid.Sel13*solid.eel13+solid.Sel22*solid.eel22+2*solid.Sel23*solid.eel23+solid.Sel33*solid.eel33) | J/m³ | Elastic strain energy density | Domain 5 | + operation |
| solid.WsGp | max(0,solid.gpeval(solid.Ws)) | J/m³ | Elastic strain energy density | Domain 5 |  |
| solid.Ws_tot | solid.pzm1.intDom(solid.Ws*solid.pzm1.integrand*solid.pzm1.gsv) | J | Total elastic strain energy | Global | + operation |
| solid.Ws_current | 0.5*solid.Ji*(real(solid.Sel11)*real(solid.eel11)+2*real(solid.Sel12)*real(solid.eel12)+2*real(solid.Sel13)*real(solid.eel13)+real(solid.Sel22)*real(solid.eel22)+2*real(solid.Sel23)*real(solid.eel23)+real(solid.Sel33)*real(solid.eel33)) | J/m³ | Elastic strain energy density, current phase | Domain 5 | + operation |
| solid.Wh | 0.25*solid.Ji*real(solid.eel11*conj(solid.Sel11)+2*solid.eel12*conj(solid.Sel12)+2*solid.eel13*conj(solid.Sel13)+solid.eel22*conj(solid.Sel22)+2*solid.eel23*conj(solid.Sel23)+solid.eel33*conj(solid.Sel33))*!isPML | J/m³ | Stored energy density | Domain 5 | + operation |
| solid.WhGp | max(0,solid.gpeval(solid.Wh)) | J/m³ | Stored energy density | Domain 5 |  |
| solid.Wh_tot | solid.pzm1.intDom(solid.Wh*solid.pzm1.integrand*solid.pzm1.gsv) | J | Total stored energy | Global | + operation |
| solid.Wk | 0.25*solid.rho*(realdot(u*solid.iomega,u*solid.iomega)+realdot(v*solid.iomega,v*solid.iomega))*!isPML | J/m³ | Kinetic energy density | Domain 5 | + operation |
| solid.Wk_tot | solid.pzm1.intDomFull(solid.Wk*solid.pzm1.integrand*solid.pzm1.gsv) | J | Total kinetic energy | Global | + operation |
| solid.Eequ | 9*solid.Kequ*solid.Gequ/(3*solid.Kequ+solid.Gequ) | Pa | Equivalent Young's modulus | Domain 5 |  |
| solid.nuequ | -1+0.5*solid.Eequ/solid.Gequ | 1 | Equivalent Poisson's ratio | Domain 5 |  |
| solid.Gequ | (solid.D11+solid.D22+solid.D33-solid.D12-solid.D23-solid.D13)/15+0.2*(solid.D44+solid.D55+solid.D66) | N/m² | Equivalent shear modulus | Domain 5 |  |
| solid.Kequ | (solid.D11+solid.D22+solid.D33+2*(solid.D12+solid.D13+solid.D23))/9 | N/m² | Equivalent bulk modulus | Domain 5 |  |
| solid.Eequtot | solid.Eequ | Pa | Total equivalent Young's modulus | Domain 5 | + operation |
| solid.Gequtot | solid.Gequ | N/m² | Total equivalent shear modulus | Domain 5 | + operation |
| solid.Kequtot | solid.Kequ | N/m² | Total equivalent bulk modulus | Domain 5 | + operation |
| solid.K | (solid.Cgl11+solid.Cgl22+solid.Cgl33)/9 | N/m² | Bulk modulus | Domain 5 | * operation |
| solid.D11 | solid.cE11 | Pa | Elasticity matrix, 11-component | Domain 5 | * operation |
| solid.D12 | solid.cE12 | Pa | Elasticity matrix, 12-component | Domain 5 | * operation |
| solid.D13 | solid.cE13 | Pa | Elasticity matrix, 13-component | Domain 5 | * operation |
| solid.D14 | solid.cE16 | Pa | Elasticity matrix, 14-component | Domain 5 | * operation |
| solid.D15 | solid.cE14 | Pa | Elasticity matrix, 15-component | Domain 5 | * operation |
| solid.D16 | solid.cE15 | Pa | Elasticity matrix, 16-component | Domain 5 | * operation |
| solid.D22 | solid.cE22 | Pa | Elasticity matrix, 22-component | Domain 5 | * operation |
| solid.D23 | solid.cE23 | Pa | Elasticity matrix, 23-component | Domain 5 | * operation |
| solid.D24 | solid.cE26 | Pa | Elasticity matrix, 24-component | Domain 5 | * operation |
| solid.D25 | solid.cE24 | Pa | Elasticity matrix, 25-component | Domain 5 | * operation |
| solid.D26 | solid.cE25 | Pa | Elasticity matrix, 26-component | Domain 5 | * operation |
| solid.D33 | solid.cE33 | Pa | Elasticity matrix, 33-component | Domain 5 | * operation |
| solid.D34 | solid.cE36 | Pa | Elasticity matrix, 34-component | Domain 5 | * operation |
| solid.D35 | solid.cE34 | Pa | Elasticity matrix, 35-component | Domain 5 | * operation |
| solid.D36 | solid.cE35 | Pa | Elasticity matrix, 36-component | Domain 5 | * operation |
| solid.D44 | solid.cE66 | Pa | Elasticity matrix, 44-component | Domain 5 | * operation |
| solid.D45 | solid.cE46 | Pa | Elasticity matrix, 45-component | Domain 5 | * operation |
| solid.D46 | solid.cE56 | Pa | Elasticity matrix, 46-component | Domain 5 | * operation |
| solid.D55 | solid.cE44 | Pa | Elasticity matrix, 55-component | Domain 5 | * operation |
| solid.D56 | solid.cE45 | Pa | Elasticity matrix, 56-component | Domain 5 | * operation |
| solid.D66 | solid.cE55 | Pa | Elasticity matrix, 66-component | Domain 5 | * operation |
| solid.DVo11 | solid.cE11 | Pa | Elasticity matrix, Voigt notation, 11-component | Domain 5 |  |
| solid.DVo12 | solid.cE12 | Pa | Elasticity matrix, Voigt notation, 12-component | Domain 5 |  |
| solid.DVo13 | solid.cE13 | Pa | Elasticity matrix, Voigt notation, 13-component | Domain 5 |  |
| solid.DVo14 | solid.cE14 | Pa | Elasticity matrix, Voigt notation, 14-component | Domain 5 |  |
| solid.DVo15 | solid.cE15 | Pa | Elasticity matrix, Voigt notation, 15-component | Domain 5 |  |
| solid.DVo16 | solid.cE16 | Pa | Elasticity matrix, Voigt notation, 16-component | Domain 5 |  |
| solid.DVo22 | solid.cE22 | Pa | Elasticity matrix, Voigt notation, 22-component | Domain 5 |  |
| solid.DVo23 | solid.cE23 | Pa | Elasticity matrix, Voigt notation, 23-component | Domain 5 |  |
| solid.DVo24 | solid.cE24 | Pa | Elasticity matrix, Voigt notation, 24-component | Domain 5 |  |
| solid.DVo25 | solid.cE25 | Pa | Elasticity matrix, Voigt notation, 25-component | Domain 5 |  |
| solid.DVo26 | solid.cE26 | Pa | Elasticity matrix, Voigt notation, 26-component | Domain 5 |  |
| solid.DVo33 | solid.cE33 | Pa | Elasticity matrix, Voigt notation, 33-component | Domain 5 |  |
| solid.DVo34 | solid.cE34 | Pa | Elasticity matrix, Voigt notation, 34-component | Domain 5 |  |
| solid.DVo35 | solid.cE35 | Pa | Elasticity matrix, Voigt notation, 35-component | Domain 5 |  |
| solid.DVo36 | solid.cE36 | Pa | Elasticity matrix, Voigt notation, 36-component | Domain 5 |  |
| solid.DVo44 | solid.cE44 | Pa | Elasticity matrix, Voigt notation, 44-component | Domain 5 |  |
| solid.DVo45 | solid.cE45 | Pa | Elasticity matrix, Voigt notation, 45-component | Domain 5 |  |
| solid.DVo46 | solid.cE46 | Pa | Elasticity matrix, Voigt notation, 46-component | Domain 5 |  |
| solid.DVo55 | solid.cE55 | Pa | Elasticity matrix, Voigt notation, 55-component | Domain 5 |  |
| solid.DVo56 | solid.cE56 | Pa | Elasticity matrix, Voigt notation, 56-component | Domain 5 |  |
| solid.DVo66 | solid.cE66 | Pa | Elasticity matrix, Voigt notation, 66-component | Domain 5 |  |
| solid.DVog11 | (solid.pzm1.bM11*solid.DVo11+solid.pzm1.bM12*solid.DVo12+solid.pzm1.bM13*solid.DVo13+solid.pzm1.bM14*solid.DVo14+solid.pzm1.bM15*solid.DVo15+solid.pzm1.bM16*solid.DVo16)*solid.pzm1.bM11+(solid.pzm1.bM11*solid.DVo12+solid.pzm1.bM12*solid.DVo22+solid.pzm1.bM13*solid.DVo23+solid.pzm1.bM14*solid.DVo24+solid.pzm1.bM15*solid.DVo25+solid.pzm1.bM16*solid.DVo26)*solid.pzm1.bM12+(solid.pzm1.bM11*solid.DVo13+solid.pzm1.bM12*solid.DVo23+solid.pzm1.bM13*solid.DVo33+solid.pzm1.bM14*solid.DVo34+solid.pzm1.bM15*solid.DVo35+solid.pzm1.bM16*solid.DVo36)*solid.pzm1.bM13+(solid.pzm1.bM11*solid.DVo14+solid.pzm1.bM12*solid.DVo24+solid.pzm1.bM13*solid.DVo34+solid.pzm1.bM14*solid.DVo44+solid.pzm1.bM15*solid.DVo45+solid.pzm1.bM16*solid.DVo46)*solid.pzm1.bM14+(solid.pzm1.bM11*solid.DVo15+solid.pzm1.bM12*solid.DVo25+solid.pzm1.bM13*solid.DVo35+solid.pzm1.bM14*solid.DVo45+solid.pzm1.bM15*solid.DVo55+solid.pzm1.bM16*solid.DVo56)*solid.pzm1.bM15+(solid.pzm1.bM11*solid.DVo16+solid.pzm1.bM12*solid.DVo26+solid.pzm1.bM13*solid.DVo36+solid.pzm1.bM14*solid.DVo46+solid.pzm1.bM15*solid.DVo56+solid.pzm1.bM16*solid.DVo66)*solid.pzm1.bM16 | Pa | Elasticity matrix, Voigt notation (global), 11-component | Domain 5 |  |
| solid.DVog12 | (solid.pzm1.bM11*solid.DVo11+solid.pzm1.bM12*solid.DVo12+solid.pzm1.bM13*solid.DVo13+solid.pzm1.bM14*solid.DVo14+solid.pzm1.bM15*solid.DVo15+solid.pzm1.bM16*solid.DVo16)*solid.pzm1.bM21+(solid.pzm1.bM11*solid.DVo12+solid.pzm1.bM12*solid.DVo22+solid.pzm1.bM13*solid.DVo23+solid.pzm1.bM14*solid.DVo24+solid.pzm1.bM15*solid.DVo25+solid.pzm1.bM16*solid.DVo26)*solid.pzm1.bM22+(solid.pzm1.bM11*solid.DVo13+solid.pzm1.bM12*solid.DVo23+solid.pzm1.bM13*solid.DVo33+solid.pzm1.bM14*solid.DVo34+solid.pzm1.bM15*solid.DVo35+solid.pzm1.bM16*solid.DVo36)*solid.pzm1.bM23+(solid.pzm1.bM11*solid.DVo14+solid.pzm1.bM12*solid.DVo24+solid.pzm1.bM13*solid.DVo34+solid.pzm1.bM14*solid.DVo44+solid.pzm1.bM15*solid.DVo45+solid.pzm1.bM16*solid.DVo46)*solid.pzm1.bM24+(solid.pzm1.bM11*solid.DVo15+solid.pzm1.bM12*solid.DVo25+solid.pzm1.bM13*solid.DVo35+solid.pzm1.bM14*solid.DVo45+solid.pzm1.bM15*solid.DVo55+solid.pzm1.bM16*solid.DVo56)*solid.pzm1.bM25+(solid.pzm1.bM11*solid.DVo16+solid.pzm1.bM12*solid.DVo26+solid.pzm1.bM13*solid.DVo36+solid.pzm1.bM14*solid.DVo46+solid.pzm1.bM15*solid.DVo56+solid.pzm1.bM16*solid.DVo66)*solid.pzm1.bM26 | Pa | Elasticity matrix, Voigt notation (global), 12-component | Domain 5 |  |
| solid.DVog13 | (solid.pzm1.bM11*solid.DVo11+solid.pzm1.bM12*solid.DVo12+solid.pzm1.bM13*solid.DVo13+solid.pzm1.bM14*solid.DVo14+solid.pzm1.bM15*solid.DVo15+solid.pzm1.bM16*solid.DVo16)*solid.pzm1.bM31+(solid.pzm1.bM11*solid.DVo12+solid.pzm1.bM12*solid.DVo22+solid.pzm1.bM13*solid.DVo23+solid.pzm1.bM14*solid.DVo24+solid.pzm1.bM15*solid.DVo25+solid.pzm1.bM16*solid.DVo26)*solid.pzm1.bM32+(solid.pzm1.bM11*solid.DVo13+solid.pzm1.bM12*solid.DVo23+solid.pzm1.bM13*solid.DVo33+solid.pzm1.bM14*solid.DVo34+solid.pzm1.bM15*solid.DVo35+solid.pzm1.bM16*solid.DVo36)*solid.pzm1.bM33+(solid.pzm1.bM11*solid.DVo14+solid.pzm1.bM12*solid.DVo24+solid.pzm1.bM13*solid.DVo34+solid.pzm1.bM14*solid.DVo44+solid.pzm1.bM15*solid.DVo45+solid.pzm1.bM16*solid.DVo46)*solid.pzm1.bM34+(solid.pzm1.bM11*solid.DVo15+solid.pzm1.bM12*solid.DVo25+solid.pzm1.bM13*solid.DVo35+solid.pzm1.bM14*solid.DVo45+solid.pzm1.bM15*solid.DVo55+solid.pzm1.bM16*solid.DVo56)*solid.pzm1.bM35+(solid.pzm1.bM11*solid.DVo16+solid.pzm1.bM12*solid.DVo26+solid.pzm1.bM13*solid.DVo36+solid.pzm1.bM14*solid.DVo46+solid.pzm1.bM15*solid.DVo56+solid.pzm1.bM16*solid.DVo66)*solid.pzm1.bM36 | Pa | Elasticity matrix, Voigt notation (global), 13-component | Domain 5 |  |
| solid.DVog14 | (solid.pzm1.bM11*solid.DVo11+solid.pzm1.bM12*solid.DVo12+solid.pzm1.bM13*solid.DVo13+solid.pzm1.bM14*solid.DVo14+solid.pzm1.bM15*solid.DVo15+solid.pzm1.bM16*solid.DVo16)*solid.pzm1.bM41+(solid.pzm1.bM11*solid.DVo12+solid.pzm1.bM12*solid.DVo22+solid.pzm1.bM13*solid.DVo23+solid.pzm1.bM14*solid.DVo24+solid.pzm1.bM15*solid.DVo25+solid.pzm1.bM16*solid.DVo26)*solid.pzm1.bM42+(solid.pzm1.bM11*solid.DVo13+solid.pzm1.bM12*solid.DVo23+solid.pzm1.bM13*solid.DVo33+solid.pzm1.bM14*solid.DVo34+solid.pzm1.bM15*solid.DVo35+solid.pzm1.bM16*solid.DVo36)*solid.pzm1.bM43+(solid.pzm1.bM11*solid.DVo14+solid.pzm1.bM12*solid.DVo24+solid.pzm1.bM13*solid.DVo34+solid.pzm1.bM14*solid.DVo44+solid.pzm1.bM15*solid.DVo45+solid.pzm1.bM16*solid.DVo46)*solid.pzm1.bM44+(solid.pzm1.bM11*solid.DVo15+solid.pzm1.bM12*solid.DVo25+solid.pzm1.bM13*solid.DVo35+solid.pzm1.bM14*solid.DVo45+solid.pzm1.bM15*solid.DVo55+solid.pzm1.bM16*solid.DVo56)*solid.pzm1.bM45+(solid.pzm1.bM11*solid.DVo16+solid.pzm1.bM12*solid.DVo26+solid.pzm1.bM13*solid.DVo36+solid.pzm1.bM14*solid.DVo46+solid.pzm1.bM15*solid.DVo56+solid.pzm1.bM16*solid.DVo66)*solid.pzm1.bM46 | Pa | Elasticity matrix, Voigt notation (global), 14-component | Domain 5 |  |
| solid.DVog15 | (solid.pzm1.bM11*solid.DVo11+solid.pzm1.bM12*solid.DVo12+solid.pzm1.bM13*solid.DVo13+solid.pzm1.bM14*solid.DVo14+solid.pzm1.bM15*solid.DVo15+solid.pzm1.bM16*solid.DVo16)*solid.pzm1.bM51+(solid.pzm1.bM11*solid.DVo12+solid.pzm1.bM12*solid.DVo22+solid.pzm1.bM13*solid.DVo23+solid.pzm1.bM14*solid.DVo24+solid.pzm1.bM15*solid.DVo25+solid.pzm1.bM16*solid.DVo26)*solid.pzm1.bM52+(solid.pzm1.bM11*solid.DVo13+solid.pzm1.bM12*solid.DVo23+solid.pzm1.bM13*solid.DVo33+solid.pzm1.bM14*solid.DVo34+solid.pzm1.bM15*solid.DVo35+solid.pzm1.bM16*solid.DVo36)*solid.pzm1.bM53+(solid.pzm1.bM11*solid.DVo14+solid.pzm1.bM12*solid.DVo24+solid.pzm1.bM13*solid.DVo34+solid.pzm1.bM14*solid.DVo44+solid.pzm1.bM15*solid.DVo45+solid.pzm1.bM16*solid.DVo46)*solid.pzm1.bM54+(solid.pzm1.bM11*solid.DVo15+solid.pzm1.bM12*solid.DVo25+solid.pzm1.bM13*solid.DVo35+solid.pzm1.bM14*solid.DVo45+solid.pzm1.bM15*solid.DVo55+solid.pzm1.bM16*solid.DVo56)*solid.pzm1.bM55+(solid.pzm1.bM11*solid.DVo16+solid.pzm1.bM12*solid.DVo26+solid.pzm1.bM13*solid.DVo36+solid.pzm1.bM14*solid.DVo46+solid.pzm1.bM15*solid.DVo56+solid.pzm1.bM16*solid.DVo66)*solid.pzm1.bM56 | Pa | Elasticity matrix, Voigt notation (global), 15-component | Domain 5 |  |
| solid.DVog16 | (solid.pzm1.bM11*solid.DVo11+solid.pzm1.bM12*solid.DVo12+solid.pzm1.bM13*solid.DVo13+solid.pzm1.bM14*solid.DVo14+solid.pzm1.bM15*solid.DVo15+solid.pzm1.bM16*solid.DVo16)*solid.pzm1.bM61+(solid.pzm1.bM11*solid.DVo12+solid.pzm1.bM12*solid.DVo22+solid.pzm1.bM13*solid.DVo23+solid.pzm1.bM14*solid.DVo24+solid.pzm1.bM15*solid.DVo25+solid.pzm1.bM16*solid.DVo26)*solid.pzm1.bM62+(solid.pzm1.bM11*solid.DVo13+solid.pzm1.bM12*solid.DVo23+solid.pzm1.bM13*solid.DVo33+solid.pzm1.bM14*solid.DVo34+solid.pzm1.bM15*solid.DVo35+solid.pzm1.bM16*solid.DVo36)*solid.pzm1.bM63+(solid.pzm1.bM11*solid.DVo14+solid.pzm1.bM12*solid.DVo24+solid.pzm1.bM13*solid.DVo34+solid.pzm1.bM14*solid.DVo44+solid.pzm1.bM15*solid.DVo45+solid.pzm1.bM16*solid.DVo46)*solid.pzm1.bM64+(solid.pzm1.bM11*solid.DVo15+solid.pzm1.bM12*solid.DVo25+solid.pzm1.bM13*solid.DVo35+solid.pzm1.bM14*solid.DVo45+solid.pzm1.bM15*solid.DVo55+solid.pzm1.bM16*solid.DVo56)*solid.pzm1.bM65+(solid.pzm1.bM11*solid.DVo16+solid.pzm1.bM12*solid.DVo26+solid.pzm1.bM13*solid.DVo36+solid.pzm1.bM14*solid.DVo46+solid.pzm1.bM15*solid.DVo56+solid.pzm1.bM16*solid.DVo66)*solid.pzm1.bM66 | Pa | Elasticity matrix, Voigt notation (global), 16-component | Domain 5 |  |
| solid.DVog22 | (solid.pzm1.bM21*solid.DVo11+solid.pzm1.bM22*solid.DVo12+solid.pzm1.bM23*solid.DVo13+solid.pzm1.bM24*solid.DVo14+solid.pzm1.bM25*solid.DVo15+solid.pzm1.bM26*solid.DVo16)*solid.pzm1.bM21+(solid.pzm1.bM21*solid.DVo12+solid.pzm1.bM22*solid.DVo22+solid.pzm1.bM23*solid.DVo23+solid.pzm1.bM24*solid.DVo24+solid.pzm1.bM25*solid.DVo25+solid.pzm1.bM26*solid.DVo26)*solid.pzm1.bM22+(solid.pzm1.bM21*solid.DVo13+solid.pzm1.bM22*solid.DVo23+solid.pzm1.bM23*solid.DVo33+solid.pzm1.bM24*solid.DVo34+solid.pzm1.bM25*solid.DVo35+solid.pzm1.bM26*solid.DVo36)*solid.pzm1.bM23+(solid.pzm1.bM21*solid.DVo14+solid.pzm1.bM22*solid.DVo24+solid.pzm1.bM23*solid.DVo34+solid.pzm1.bM24*solid.DVo44+solid.pzm1.bM25*solid.DVo45+solid.pzm1.bM26*solid.DVo46)*solid.pzm1.bM24+(solid.pzm1.bM21*solid.DVo15+solid.pzm1.bM22*solid.DVo25+solid.pzm1.bM23*solid.DVo35+solid.pzm1.bM24*solid.DVo45+solid.pzm1.bM25*solid.DVo55+solid.pzm1.bM26*solid.DVo56)*solid.pzm1.bM25+(solid.pzm1.bM21*solid.DVo16+solid.pzm1.bM22*solid.DVo26+solid.pzm1.bM23*solid.DVo36+solid.pzm1.bM24*solid.DVo46+solid.pzm1.bM25*solid.DVo56+solid.pzm1.bM26*solid.DVo66)*solid.pzm1.bM26 | Pa | Elasticity matrix, Voigt notation (global), 22-component | Domain 5 |  |
| solid.DVog23 | (solid.pzm1.bM21*solid.DVo11+solid.pzm1.bM22*solid.DVo12+solid.pzm1.bM23*solid.DVo13+solid.pzm1.bM24*solid.DVo14+solid.pzm1.bM25*solid.DVo15+solid.pzm1.bM26*solid.DVo16)*solid.pzm1.bM31+(solid.pzm1.bM21*solid.DVo12+solid.pzm1.bM22*solid.DVo22+solid.pzm1.bM23*solid.DVo23+solid.pzm1.bM24*solid.DVo24+solid.pzm1.bM25*solid.DVo25+solid.pzm1.bM26*solid.DVo26)*solid.pzm1.bM32+(solid.pzm1.bM21*solid.DVo13+solid.pzm1.bM22*solid.DVo23+solid.pzm1.bM23*solid.DVo33+solid.pzm1.bM24*solid.DVo34+solid.pzm1.bM25*solid.DVo35+solid.pzm1.bM26*solid.DVo36)*solid.pzm1.bM33+(solid.pzm1.bM21*solid.DVo14+solid.pzm1.bM22*solid.DVo24+solid.pzm1.bM23*solid.DVo34+solid.pzm1.bM24*solid.DVo44+solid.pzm1.bM25*solid.DVo45+solid.pzm1.bM26*solid.DVo46)*solid.pzm1.bM34+(solid.pzm1.bM21*solid.DVo15+solid.pzm1.bM22*solid.DVo25+solid.pzm1.bM23*solid.DVo35+solid.pzm1.bM24*solid.DVo45+solid.pzm1.bM25*solid.DVo55+solid.pzm1.bM26*solid.DVo56)*solid.pzm1.bM35+(solid.pzm1.bM21*solid.DVo16+solid.pzm1.bM22*solid.DVo26+solid.pzm1.bM23*solid.DVo36+solid.pzm1.bM24*solid.DVo46+solid.pzm1.bM25*solid.DVo56+solid.pzm1.bM26*solid.DVo66)*solid.pzm1.bM36 | Pa | Elasticity matrix, Voigt notation (global), 23-component | Domain 5 |  |
| solid.DVog24 | (solid.pzm1.bM21*solid.DVo11+solid.pzm1.bM22*solid.DVo12+solid.pzm1.bM23*solid.DVo13+solid.pzm1.bM24*solid.DVo14+solid.pzm1.bM25*solid.DVo15+solid.pzm1.bM26*solid.DVo16)*solid.pzm1.bM41+(solid.pzm1.bM21*solid.DVo12+solid.pzm1.bM22*solid.DVo22+solid.pzm1.bM23*solid.DVo23+solid.pzm1.bM24*solid.DVo24+solid.pzm1.bM25*solid.DVo25+solid.pzm1.bM26*solid.DVo26)*solid.pzm1.bM42+(solid.pzm1.bM21*solid.DVo13+solid.pzm1.bM22*solid.DVo23+solid.pzm1.bM23*solid.DVo33+solid.pzm1.bM24*solid.DVo34+solid.pzm1.bM25*solid.DVo35+solid.pzm1.bM26*solid.DVo36)*solid.pzm1.bM43+(solid.pzm1.bM21*solid.DVo14+solid.pzm1.bM22*solid.DVo24+solid.pzm1.bM23*solid.DVo34+solid.pzm1.bM24*solid.DVo44+solid.pzm1.bM25*solid.DVo45+solid.pzm1.bM26*solid.DVo46)*solid.pzm1.bM44+(solid.pzm1.bM21*solid.DVo15+solid.pzm1.bM22*solid.DVo25+solid.pzm1.bM23*solid.DVo35+solid.pzm1.bM24*solid.DVo45+solid.pzm1.bM25*solid.DVo55+solid.pzm1.bM26*solid.DVo56)*solid.pzm1.bM45+(solid.pzm1.bM21*solid.DVo16+solid.pzm1.bM22*solid.DVo26+solid.pzm1.bM23*solid.DVo36+solid.pzm1.bM24*solid.DVo46+solid.pzm1.bM25*solid.DVo56+solid.pzm1.bM26*solid.DVo66)*solid.pzm1.bM46 | Pa | Elasticity matrix, Voigt notation (global), 24-component | Domain 5 |  |
| solid.DVog25 | (solid.pzm1.bM21*solid.DVo11+solid.pzm1.bM22*solid.DVo12+solid.pzm1.bM23*solid.DVo13+solid.pzm1.bM24*solid.DVo14+solid.pzm1.bM25*solid.DVo15+solid.pzm1.bM26*solid.DVo16)*solid.pzm1.bM51+(solid.pzm1.bM21*solid.DVo12+solid.pzm1.bM22*solid.DVo22+solid.pzm1.bM23*solid.DVo23+solid.pzm1.bM24*solid.DVo24+solid.pzm1.bM25*solid.DVo25+solid.pzm1.bM26*solid.DVo26)*solid.pzm1.bM52+(solid.pzm1.bM21*solid.DVo13+solid.pzm1.bM22*solid.DVo23+solid.pzm1.bM23*solid.DVo33+solid.pzm1.bM24*solid.DVo34+solid.pzm1.bM25*solid.DVo35+solid.pzm1.bM26*solid.DVo36)*solid.pzm1.bM53+(solid.pzm1.bM21*solid.DVo14+solid.pzm1.bM22*solid.DVo24+solid.pzm1.bM23*solid.DVo34+solid.pzm1.bM24*solid.DVo44+solid.pzm1.bM25*solid.DVo45+solid.pzm1.bM26*solid.DVo46)*solid.pzm1.bM54+(solid.pzm1.bM21*solid.DVo15+solid.pzm1.bM22*solid.DVo25+solid.pzm1.bM23*solid.DVo35+solid.pzm1.bM24*solid.DVo45+solid.pzm1.bM25*solid.DVo55+solid.pzm1.bM26*solid.DVo56)*solid.pzm1.bM55+(solid.pzm1.bM21*solid.DVo16+solid.pzm1.bM22*solid.DVo26+solid.pzm1.bM23*solid.DVo36+solid.pzm1.bM24*solid.DVo46+solid.pzm1.bM25*solid.DVo56+solid.pzm1.bM26*solid.DVo66)*solid.pzm1.bM56 | Pa | Elasticity matrix, Voigt notation (global), 25-component | Domain 5 |  |
| solid.DVog26 | (solid.pzm1.bM21*solid.DVo11+solid.pzm1.bM22*solid.DVo12+solid.pzm1.bM23*solid.DVo13+solid.pzm1.bM24*solid.DVo14+solid.pzm1.bM25*solid.DVo15+solid.pzm1.bM26*solid.DVo16)*solid.pzm1.bM61+(solid.pzm1.bM21*solid.DVo12+solid.pzm1.bM22*solid.DVo22+solid.pzm1.bM23*solid.DVo23+solid.pzm1.bM24*solid.DVo24+solid.pzm1.bM25*solid.DVo25+solid.pzm1.bM26*solid.DVo26)*solid.pzm1.bM62+(solid.pzm1.bM21*solid.DVo13+solid.pzm1.bM22*solid.DVo23+solid.pzm1.bM23*solid.DVo33+solid.pzm1.bM24*solid.DVo34+solid.pzm1.bM25*solid.DVo35+solid.pzm1.bM26*solid.DVo36)*solid.pzm1.bM63+(solid.pzm1.bM21*solid.DVo14+solid.pzm1.bM22*solid.DVo24+solid.pzm1.bM23*solid.DVo34+solid.pzm1.bM24*solid.DVo44+solid.pzm1.bM25*solid.DVo45+solid.pzm1.bM26*solid.DVo46)*solid.pzm1.bM64+(solid.pzm1.bM21*solid.DVo15+solid.pzm1.bM22*solid.DVo25+solid.pzm1.bM23*solid.DVo35+solid.pzm1.bM24*solid.DVo45+solid.pzm1.bM25*solid.DVo55+solid.pzm1.bM26*solid.DVo56)*solid.pzm1.bM65+(solid.pzm1.bM21*solid.DVo16+solid.pzm1.bM22*solid.DVo26+solid.pzm1.bM23*solid.DVo36+solid.pzm1.bM24*solid.DVo46+solid.pzm1.bM25*solid.DVo56+solid.pzm1.bM26*solid.DVo66)*solid.pzm1.bM66 | Pa | Elasticity matrix, Voigt notation (global), 26-component | Domain 5 |  |
| solid.DVog33 | (solid.pzm1.bM31*solid.DVo11+solid.pzm1.bM32*solid.DVo12+solid.pzm1.bM33*solid.DVo13+solid.pzm1.bM34*solid.DVo14+solid.pzm1.bM35*solid.DVo15+solid.pzm1.bM36*solid.DVo16)*solid.pzm1.bM31+(solid.pzm1.bM31*solid.DVo12+solid.pzm1.bM32*solid.DVo22+solid.pzm1.bM33*solid.DVo23+solid.pzm1.bM34*solid.DVo24+solid.pzm1.bM35*solid.DVo25+solid.pzm1.bM36*solid.DVo26)*solid.pzm1.bM32+(solid.pzm1.bM31*solid.DVo13+solid.pzm1.bM32*solid.DVo23+solid.pzm1.bM33*solid.DVo33+solid.pzm1.bM34*solid.DVo34+solid.pzm1.bM35*solid.DVo35+solid.pzm1.bM36*solid.DVo36)*solid.pzm1.bM33+(solid.pzm1.bM31*solid.DVo14+solid.pzm1.bM32*solid.DVo24+solid.pzm1.bM33*solid.DVo34+solid.pzm1.bM34*solid.DVo44+solid.pzm1.bM35*solid.DVo45+solid.pzm1.bM36*solid.DVo46)*solid.pzm1.bM34+(solid.pzm1.bM31*solid.DVo15+solid.pzm1.bM32*solid.DVo25+solid.pzm1.bM33*solid.DVo35+solid.pzm1.bM34*solid.DVo45+solid.pzm1.bM35*solid.DVo55+solid.pzm1.bM36*solid.DVo56)*solid.pzm1.bM35+(solid.pzm1.bM31*solid.DVo16+solid.pzm1.bM32*solid.DVo26+solid.pzm1.bM33*solid.DVo36+solid.pzm1.bM34*solid.DVo46+solid.pzm1.bM35*solid.DVo56+solid.pzm1.bM36*solid.DVo66)*solid.pzm1.bM36 | Pa | Elasticity matrix, Voigt notation (global), 33-component | Domain 5 |  |
| solid.DVog34 | (solid.pzm1.bM31*solid.DVo11+solid.pzm1.bM32*solid.DVo12+solid.pzm1.bM33*solid.DVo13+solid.pzm1.bM34*solid.DVo14+solid.pzm1.bM35*solid.DVo15+solid.pzm1.bM36*solid.DVo16)*solid.pzm1.bM41+(solid.pzm1.bM31*solid.DVo12+solid.pzm1.bM32*solid.DVo22+solid.pzm1.bM33*solid.DVo23+solid.pzm1.bM34*solid.DVo24+solid.pzm1.bM35*solid.DVo25+solid.pzm1.bM36*solid.DVo26)*solid.pzm1.bM42+(solid.pzm1.bM31*solid.DVo13+solid.pzm1.bM32*solid.DVo23+solid.pzm1.bM33*solid.DVo33+solid.pzm1.bM34*solid.DVo34+solid.pzm1.bM35*solid.DVo35+solid.pzm1.bM36*solid.DVo36)*solid.pzm1.bM43+(solid.pzm1.bM31*solid.DVo14+solid.pzm1.bM32*solid.DVo24+solid.pzm1.bM33*solid.DVo34+solid.pzm1.bM34*solid.DVo44+solid.pzm1.bM35*solid.DVo45+solid.pzm1.bM36*solid.DVo46)*solid.pzm1.bM44+(solid.pzm1.bM31*solid.DVo15+solid.pzm1.bM32*solid.DVo25+solid.pzm1.bM33*solid.DVo35+solid.pzm1.bM34*solid.DVo45+solid.pzm1.bM35*solid.DVo55+solid.pzm1.bM36*solid.DVo56)*solid.pzm1.bM45+(solid.pzm1.bM31*solid.DVo16+solid.pzm1.bM32*solid.DVo26+solid.pzm1.bM33*solid.DVo36+solid.pzm1.bM34*solid.DVo46+solid.pzm1.bM35*solid.DVo56+solid.pzm1.bM36*solid.DVo66)*solid.pzm1.bM46 | Pa | Elasticity matrix, Voigt notation (global), 34-component | Domain 5 |  |
| solid.DVog35 | (solid.pzm1.bM31*solid.DVo11+solid.pzm1.bM32*solid.DVo12+solid.pzm1.bM33*solid.DVo13+solid.pzm1.bM34*solid.DVo14+solid.pzm1.bM35*solid.DVo15+solid.pzm1.bM36*solid.DVo16)*solid.pzm1.bM51+(solid.pzm1.bM31*solid.DVo12+solid.pzm1.bM32*solid.DVo22+solid.pzm1.bM33*solid.DVo23+solid.pzm1.bM34*solid.DVo24+solid.pzm1.bM35*solid.DVo25+solid.pzm1.bM36*solid.DVo26)*solid.pzm1.bM52+(solid.pzm1.bM31*solid.DVo13+solid.pzm1.bM32*solid.DVo23+solid.pzm1.bM33*solid.DVo33+solid.pzm1.bM34*solid.DVo34+solid.pzm1.bM35*solid.DVo35+solid.pzm1.bM36*solid.DVo36)*solid.pzm1.bM53+(solid.pzm1.bM31*solid.DVo14+solid.pzm1.bM32*solid.DVo24+solid.pzm1.bM33*solid.DVo34+solid.pzm1.bM34*solid.DVo44+solid.pzm1.bM35*solid.DVo45+solid.pzm1.bM36*solid.DVo46)*solid.pzm1.bM54+(solid.pzm1.bM31*solid.DVo15+solid.pzm1.bM32*solid.DVo25+solid.pzm1.bM33*solid.DVo35+solid.pzm1.bM34*solid.DVo45+solid.pzm1.bM35*solid.DVo55+solid.pzm1.bM36*solid.DVo56)*solid.pzm1.bM55+(solid.pzm1.bM31*solid.DVo16+solid.pzm1.bM32*solid.DVo26+solid.pzm1.bM33*solid.DVo36+solid.pzm1.bM34*solid.DVo46+solid.pzm1.bM35*solid.DVo56+solid.pzm1.bM36*solid.DVo66)*solid.pzm1.bM56 | Pa | Elasticity matrix, Voigt notation (global), 35-component | Domain 5 |  |
| solid.DVog36 | (solid.pzm1.bM31*solid.DVo11+solid.pzm1.bM32*solid.DVo12+solid.pzm1.bM33*solid.DVo13+solid.pzm1.bM34*solid.DVo14+solid.pzm1.bM35*solid.DVo15+solid.pzm1.bM36*solid.DVo16)*solid.pzm1.bM61+(solid.pzm1.bM31*solid.DVo12+solid.pzm1.bM32*solid.DVo22+solid.pzm1.bM33*solid.DVo23+solid.pzm1.bM34*solid.DVo24+solid.pzm1.bM35*solid.DVo25+solid.pzm1.bM36*solid.DVo26)*solid.pzm1.bM62+(solid.pzm1.bM31*solid.DVo13+solid.pzm1.bM32*solid.DVo23+solid.pzm1.bM33*solid.DVo33+solid.pzm1.bM34*solid.DVo34+solid.pzm1.bM35*solid.DVo35+solid.pzm1.bM36*solid.DVo36)*solid.pzm1.bM63+(solid.pzm1.bM31*solid.DVo14+solid.pzm1.bM32*solid.DVo24+solid.pzm1.bM33*solid.DVo34+solid.pzm1.bM34*solid.DVo44+solid.pzm1.bM35*solid.DVo45+solid.pzm1.bM36*solid.DVo46)*solid.pzm1.bM64+(solid.pzm1.bM31*solid.DVo15+solid.pzm1.bM32*solid.DVo25+solid.pzm1.bM33*solid.DVo35+solid.pzm1.bM34*solid.DVo45+solid.pzm1.bM35*solid.DVo55+solid.pzm1.bM36*solid.DVo56)*solid.pzm1.bM65+(solid.pzm1.bM31*solid.DVo16+solid.pzm1.bM32*solid.DVo26+solid.pzm1.bM33*solid.DVo36+solid.pzm1.bM34*solid.DVo46+solid.pzm1.bM35*solid.DVo56+solid.pzm1.bM36*solid.DVo66)*solid.pzm1.bM66 | Pa | Elasticity matrix, Voigt notation (global), 36-component | Domain 5 |  |
| solid.DVog44 | (solid.pzm1.bM41*solid.DVo11+solid.pzm1.bM42*solid.DVo12+solid.pzm1.bM43*solid.DVo13+solid.pzm1.bM44*solid.DVo14+solid.pzm1.bM45*solid.DVo15+solid.pzm1.bM46*solid.DVo16)*solid.pzm1.bM41+(solid.pzm1.bM41*solid.DVo12+solid.pzm1.bM42*solid.DVo22+solid.pzm1.bM43*solid.DVo23+solid.pzm1.bM44*solid.DVo24+solid.pzm1.bM45*solid.DVo25+solid.pzm1.bM46*solid.DVo26)*solid.pzm1.bM42+(solid.pzm1.bM41*solid.DVo13+solid.pzm1.bM42*solid.DVo23+solid.pzm1.bM43*solid.DVo33+solid.pzm1.bM44*solid.DVo34+solid.pzm1.bM45*solid.DVo35+solid.pzm1.bM46*solid.DVo36)*solid.pzm1.bM43+(solid.pzm1.bM41*solid.DVo14+solid.pzm1.bM42*solid.DVo24+solid.pzm1.bM43*solid.DVo34+solid.pzm1.bM44*solid.DVo44+solid.pzm1.bM45*solid.DVo45+solid.pzm1.bM46*solid.DVo46)*solid.pzm1.bM44+(solid.pzm1.bM41*solid.DVo15+solid.pzm1.bM42*solid.DVo25+solid.pzm1.bM43*solid.DVo35+solid.pzm1.bM44*solid.DVo45+solid.pzm1.bM45*solid.DVo55+solid.pzm1.bM46*solid.DVo56)*solid.pzm1.bM45+(solid.pzm1.bM41*solid.DVo16+solid.pzm1.bM42*solid.DVo26+solid.pzm1.bM43*solid.DVo36+solid.pzm1.bM44*solid.DVo46+solid.pzm1.bM45*solid.DVo56+solid.pzm1.bM46*solid.DVo66)*solid.pzm1.bM46 | Pa | Elasticity matrix, Voigt notation (global), 44-component | Domain 5 |  |
| solid.DVog45 | (solid.pzm1.bM41*solid.DVo11+solid.pzm1.bM42*solid.DVo12+solid.pzm1.bM43*solid.DVo13+solid.pzm1.bM44*solid.DVo14+solid.pzm1.bM45*solid.DVo15+solid.pzm1.bM46*solid.DVo16)*solid.pzm1.bM51+(solid.pzm1.bM41*solid.DVo12+solid.pzm1.bM42*solid.DVo22+solid.pzm1.bM43*solid.DVo23+solid.pzm1.bM44*solid.DVo24+solid.pzm1.bM45*solid.DVo25+solid.pzm1.bM46*solid.DVo26)*solid.pzm1.bM52+(solid.pzm1.bM41*solid.DVo13+solid.pzm1.bM42*solid.DVo23+solid.pzm1.bM43*solid.DVo33+solid.pzm1.bM44*solid.DVo34+solid.pzm1.bM45*solid.DVo35+solid.pzm1.bM46*solid.DVo36)*solid.pzm1.bM53+(solid.pzm1.bM41*solid.DVo14+solid.pzm1.bM42*solid.DVo24+solid.pzm1.bM43*solid.DVo34+solid.pzm1.bM44*solid.DVo44+solid.pzm1.bM45*solid.DVo45+solid.pzm1.bM46*solid.DVo46)*solid.pzm1.bM54+(solid.pzm1.bM41*solid.DVo15+solid.pzm1.bM42*solid.DVo25+solid.pzm1.bM43*solid.DVo35+solid.pzm1.bM44*solid.DVo45+solid.pzm1.bM45*solid.DVo55+solid.pzm1.bM46*solid.DVo56)*solid.pzm1.bM55+(solid.pzm1.bM41*solid.DVo16+solid.pzm1.bM42*solid.DVo26+solid.pzm1.bM43*solid.DVo36+solid.pzm1.bM44*solid.DVo46+solid.pzm1.bM45*solid.DVo56+solid.pzm1.bM46*solid.DVo66)*solid.pzm1.bM56 | Pa | Elasticity matrix, Voigt notation (global), 45-component | Domain 5 |  |
| solid.DVog46 | (solid.pzm1.bM41*solid.DVo11+solid.pzm1.bM42*solid.DVo12+solid.pzm1.bM43*solid.DVo13+solid.pzm1.bM44*solid.DVo14+solid.pzm1.bM45*solid.DVo15+solid.pzm1.bM46*solid.DVo16)*solid.pzm1.bM61+(solid.pzm1.bM41*solid.DVo12+solid.pzm1.bM42*solid.DVo22+solid.pzm1.bM43*solid.DVo23+solid.pzm1.bM44*solid.DVo24+solid.pzm1.bM45*solid.DVo25+solid.pzm1.bM46*solid.DVo26)*solid.pzm1.bM62+(solid.pzm1.bM41*solid.DVo13+solid.pzm1.bM42*solid.DVo23+solid.pzm1.bM43*solid.DVo33+solid.pzm1.bM44*solid.DVo34+solid.pzm1.bM45*solid.DVo35+solid.pzm1.bM46*solid.DVo36)*solid.pzm1.bM63+(solid.pzm1.bM41*solid.DVo14+solid.pzm1.bM42*solid.DVo24+solid.pzm1.bM43*solid.DVo34+solid.pzm1.bM44*solid.DVo44+solid.pzm1.bM45*solid.DVo45+solid.pzm1.bM46*solid.DVo46)*solid.pzm1.bM64+(solid.pzm1.bM41*solid.DVo15+solid.pzm1.bM42*solid.DVo25+solid.pzm1.bM43*solid.DVo35+solid.pzm1.bM44*solid.DVo45+solid.pzm1.bM45*solid.DVo55+solid.pzm1.bM46*solid.DVo56)*solid.pzm1.bM65+(solid.pzm1.bM41*solid.DVo16+solid.pzm1.bM42*solid.DVo26+solid.pzm1.bM43*solid.DVo36+solid.pzm1.bM44*solid.DVo46+solid.pzm1.bM45*solid.DVo56+solid.pzm1.bM46*solid.DVo66)*solid.pzm1.bM66 | Pa | Elasticity matrix, Voigt notation (global), 46-component | Domain 5 |  |
| solid.DVog55 | (solid.pzm1.bM51*solid.DVo11+solid.pzm1.bM52*solid.DVo12+solid.pzm1.bM53*solid.DVo13+solid.pzm1.bM54*solid.DVo14+solid.pzm1.bM55*solid.DVo15+solid.pzm1.bM56*solid.DVo16)*solid.pzm1.bM51+(solid.pzm1.bM51*solid.DVo12+solid.pzm1.bM52*solid.DVo22+solid.pzm1.bM53*solid.DVo23+solid.pzm1.bM54*solid.DVo24+solid.pzm1.bM55*solid.DVo25+solid.pzm1.bM56*solid.DVo26)*solid.pzm1.bM52+(solid.pzm1.bM51*solid.DVo13+solid.pzm1.bM52*solid.DVo23+solid.pzm1.bM53*solid.DVo33+solid.pzm1.bM54*solid.DVo34+solid.pzm1.bM55*solid.DVo35+solid.pzm1.bM56*solid.DVo36)*solid.pzm1.bM53+(solid.pzm1.bM51*solid.DVo14+solid.pzm1.bM52*solid.DVo24+solid.pzm1.bM53*solid.DVo34+solid.pzm1.bM54*solid.DVo44+solid.pzm1.bM55*solid.DVo45+solid.pzm1.bM56*solid.DVo46)*solid.pzm1.bM54+(solid.pzm1.bM51*solid.DVo15+solid.pzm1.bM52*solid.DVo25+solid.pzm1.bM53*solid.DVo35+solid.pzm1.bM54*solid.DVo45+solid.pzm1.bM55*solid.DVo55+solid.pzm1.bM56*solid.DVo56)*solid.pzm1.bM55+(solid.pzm1.bM51*solid.DVo16+solid.pzm1.bM52*solid.DVo26+solid.pzm1.bM53*solid.DVo36+solid.pzm1.bM54*solid.DVo46+solid.pzm1.bM55*solid.DVo56+solid.pzm1.bM56*solid.DVo66)*solid.pzm1.bM56 | Pa | Elasticity matrix, Voigt notation (global), 55-component | Domain 5 |  |
| solid.DVog56 | (solid.pzm1.bM51*solid.DVo11+solid.pzm1.bM52*solid.DVo12+solid.pzm1.bM53*solid.DVo13+solid.pzm1.bM54*solid.DVo14+solid.pzm1.bM55*solid.DVo15+solid.pzm1.bM56*solid.DVo16)*solid.pzm1.bM61+(solid.pzm1.bM51*solid.DVo12+solid.pzm1.bM52*solid.DVo22+solid.pzm1.bM53*solid.DVo23+solid.pzm1.bM54*solid.DVo24+solid.pzm1.bM55*solid.DVo25+solid.pzm1.bM56*solid.DVo26)*solid.pzm1.bM62+(solid.pzm1.bM51*solid.DVo13+solid.pzm1.bM52*solid.DVo23+solid.pzm1.bM53*solid.DVo33+solid.pzm1.bM54*solid.DVo34+solid.pzm1.bM55*solid.DVo35+solid.pzm1.bM56*solid.DVo36)*solid.pzm1.bM63+(solid.pzm1.bM51*solid.DVo14+solid.pzm1.bM52*solid.DVo24+solid.pzm1.bM53*solid.DVo34+solid.pzm1.bM54*solid.DVo44+solid.pzm1.bM55*solid.DVo45+solid.pzm1.bM56*solid.DVo46)*solid.pzm1.bM64+(solid.pzm1.bM51*solid.DVo15+solid.pzm1.bM52*solid.DVo25+solid.pzm1.bM53*solid.DVo35+solid.pzm1.bM54*solid.DVo45+solid.pzm1.bM55*solid.DVo55+solid.pzm1.bM56*solid.DVo56)*solid.pzm1.bM65+(solid.pzm1.bM51*solid.DVo16+solid.pzm1.bM52*solid.DVo26+solid.pzm1.bM53*solid.DVo36+solid.pzm1.bM54*solid.DVo46+solid.pzm1.bM55*solid.DVo56+solid.pzm1.bM56*solid.DVo66)*solid.pzm1.bM66 | Pa | Elasticity matrix, Voigt notation (global), 56-component | Domain 5 |  |
| solid.DVog66 | (solid.pzm1.bM61*solid.DVo11+solid.pzm1.bM62*solid.DVo12+solid.pzm1.bM63*solid.DVo13+solid.pzm1.bM64*solid.DVo14+solid.pzm1.bM65*solid.DVo15+solid.pzm1.bM66*solid.DVo16)*solid.pzm1.bM61+(solid.pzm1.bM61*solid.DVo12+solid.pzm1.bM62*solid.DVo22+solid.pzm1.bM63*solid.DVo23+solid.pzm1.bM64*solid.DVo24+solid.pzm1.bM65*solid.DVo25+solid.pzm1.bM66*solid.DVo26)*solid.pzm1.bM62+(solid.pzm1.bM61*solid.DVo13+solid.pzm1.bM62*solid.DVo23+solid.pzm1.bM63*solid.DVo33+solid.pzm1.bM64*solid.DVo34+solid.pzm1.bM65*solid.DVo35+solid.pzm1.bM66*solid.DVo36)*solid.pzm1.bM63+(solid.pzm1.bM61*solid.DVo14+solid.pzm1.bM62*solid.DVo24+solid.pzm1.bM63*solid.DVo34+solid.pzm1.bM64*solid.DVo44+solid.pzm1.bM65*solid.DVo45+solid.pzm1.bM66*solid.DVo46)*solid.pzm1.bM64+(solid.pzm1.bM61*solid.DVo15+solid.pzm1.bM62*solid.DVo25+solid.pzm1.bM63*solid.DVo35+solid.pzm1.bM64*solid.DVo45+solid.pzm1.bM65*solid.DVo55+solid.pzm1.bM66*solid.DVo56)*solid.pzm1.bM65+(solid.pzm1.bM61*solid.DVo16+solid.pzm1.bM62*solid.DVo26+solid.pzm1.bM63*solid.DVo36+solid.pzm1.bM64*solid.DVo46+solid.pzm1.bM65*solid.DVo56+solid.pzm1.bM66*solid.DVo66)*solid.pzm1.bM66 | Pa | Elasticity matrix, Voigt notation (global), 66-component | Domain 5 |  |
| solid.cEg11 | (solid.pzm1.bM11*solid.cE11+solid.pzm1.bM12*solid.cE12+solid.pzm1.bM13*solid.cE13+solid.pzm1.bM14*solid.cE14+solid.pzm1.bM15*solid.cE15+solid.pzm1.bM16*solid.cE16)*solid.pzm1.bM11+(solid.pzm1.bM11*solid.cE12+solid.pzm1.bM12*solid.cE22+solid.pzm1.bM13*solid.cE23+solid.pzm1.bM14*solid.cE24+solid.pzm1.bM15*solid.cE25+solid.pzm1.bM16*solid.cE26)*solid.pzm1.bM12+(solid.pzm1.bM11*solid.cE13+solid.pzm1.bM12*solid.cE23+solid.pzm1.bM13*solid.cE33+solid.pzm1.bM14*solid.cE34+solid.pzm1.bM15*solid.cE35+solid.pzm1.bM16*solid.cE36)*solid.pzm1.bM13+(solid.pzm1.bM11*solid.cE14+solid.pzm1.bM12*solid.cE24+solid.pzm1.bM13*solid.cE34+solid.pzm1.bM14*solid.cE44+solid.pzm1.bM15*solid.cE45+solid.pzm1.bM16*solid.cE46)*solid.pzm1.bM14+(solid.pzm1.bM11*solid.cE15+solid.pzm1.bM12*solid.cE25+solid.pzm1.bM13*solid.cE35+solid.pzm1.bM14*solid.cE45+solid.pzm1.bM15*solid.cE55+solid.pzm1.bM16*solid.cE56)*solid.pzm1.bM15+(solid.pzm1.bM11*solid.cE16+solid.pzm1.bM12*solid.cE26+solid.pzm1.bM13*solid.cE36+solid.pzm1.bM14*solid.cE46+solid.pzm1.bM15*solid.cE56+solid.pzm1.bM16*solid.cE66)*solid.pzm1.bM16 | Pa | Elasticity matrix cE (global), Voigt notation, 11-component | Domain 5 |  |
| solid.cEg12 | (solid.pzm1.bM11*solid.cE11+solid.pzm1.bM12*solid.cE12+solid.pzm1.bM13*solid.cE13+solid.pzm1.bM14*solid.cE14+solid.pzm1.bM15*solid.cE15+solid.pzm1.bM16*solid.cE16)*solid.pzm1.bM21+(solid.pzm1.bM11*solid.cE12+solid.pzm1.bM12*solid.cE22+solid.pzm1.bM13*solid.cE23+solid.pzm1.bM14*solid.cE24+solid.pzm1.bM15*solid.cE25+solid.pzm1.bM16*solid.cE26)*solid.pzm1.bM22+(solid.pzm1.bM11*solid.cE13+solid.pzm1.bM12*solid.cE23+solid.pzm1.bM13*solid.cE33+solid.pzm1.bM14*solid.cE34+solid.pzm1.bM15*solid.cE35+solid.pzm1.bM16*solid.cE36)*solid.pzm1.bM23+(solid.pzm1.bM11*solid.cE14+solid.pzm1.bM12*solid.cE24+solid.pzm1.bM13*solid.cE34+solid.pzm1.bM14*solid.cE44+solid.pzm1.bM15*solid.cE45+solid.pzm1.bM16*solid.cE46)*solid.pzm1.bM24+(solid.pzm1.bM11*solid.cE15+solid.pzm1.bM12*solid.cE25+solid.pzm1.bM13*solid.cE35+solid.pzm1.bM14*solid.cE45+solid.pzm1.bM15*solid.cE55+solid.pzm1.bM16*solid.cE56)*solid.pzm1.bM25+(solid.pzm1.bM11*solid.cE16+solid.pzm1.bM12*solid.cE26+solid.pzm1.bM13*solid.cE36+solid.pzm1.bM14*solid.cE46+solid.pzm1.bM15*solid.cE56+solid.pzm1.bM16*solid.cE66)*solid.pzm1.bM26 | Pa | Elasticity matrix cE (global), Voigt notation, 12-component | Domain 5 |  |
| solid.cEg13 | (solid.pzm1.bM11*solid.cE11+solid.pzm1.bM12*solid.cE12+solid.pzm1.bM13*solid.cE13+solid.pzm1.bM14*solid.cE14+solid.pzm1.bM15*solid.cE15+solid.pzm1.bM16*solid.cE16)*solid.pzm1.bM31+(solid.pzm1.bM11*solid.cE12+solid.pzm1.bM12*solid.cE22+solid.pzm1.bM13*solid.cE23+solid.pzm1.bM14*solid.cE24+solid.pzm1.bM15*solid.cE25+solid.pzm1.bM16*solid.cE26)*solid.pzm1.bM32+(solid.pzm1.bM11*solid.cE13+solid.pzm1.bM12*solid.cE23+solid.pzm1.bM13*solid.cE33+solid.pzm1.bM14*solid.cE34+solid.pzm1.bM15*solid.cE35+solid.pzm1.bM16*solid.cE36)*solid.pzm1.bM33+(solid.pzm1.bM11*solid.cE14+solid.pzm1.bM12*solid.cE24+solid.pzm1.bM13*solid.cE34+solid.pzm1.bM14*solid.cE44+solid.pzm1.bM15*solid.cE45+solid.pzm1.bM16*solid.cE46)*solid.pzm1.bM34+(solid.pzm1.bM11*solid.cE15+solid.pzm1.bM12*solid.cE25+solid.pzm1.bM13*solid.cE35+solid.pzm1.bM14*solid.cE45+solid.pzm1.bM15*solid.cE55+solid.pzm1.bM16*solid.cE56)*solid.pzm1.bM35+(solid.pzm1.bM11*solid.cE16+solid.pzm1.bM12*solid.cE26+solid.pzm1.bM13*solid.cE36+solid.pzm1.bM14*solid.cE46+solid.pzm1.bM15*solid.cE56+solid.pzm1.bM16*solid.cE66)*solid.pzm1.bM36 | Pa | Elasticity matrix cE (global), Voigt notation, 13-component | Domain 5 |  |
| solid.cEg14 | (solid.pzm1.bM11*solid.cE11+solid.pzm1.bM12*solid.cE12+solid.pzm1.bM13*solid.cE13+solid.pzm1.bM14*solid.cE14+solid.pzm1.bM15*solid.cE15+solid.pzm1.bM16*solid.cE16)*solid.pzm1.bM41+(solid.pzm1.bM11*solid.cE12+solid.pzm1.bM12*solid.cE22+solid.pzm1.bM13*solid.cE23+solid.pzm1.bM14*solid.cE24+solid.pzm1.bM15*solid.cE25+solid.pzm1.bM16*solid.cE26)*solid.pzm1.bM42+(solid.pzm1.bM11*solid.cE13+solid.pzm1.bM12*solid.cE23+solid.pzm1.bM13*solid.cE33+solid.pzm1.bM14*solid.cE34+solid.pzm1.bM15*solid.cE35+solid.pzm1.bM16*solid.cE36)*solid.pzm1.bM43+(solid.pzm1.bM11*solid.cE14+solid.pzm1.bM12*solid.cE24+solid.pzm1.bM13*solid.cE34+solid.pzm1.bM14*solid.cE44+solid.pzm1.bM15*solid.cE45+solid.pzm1.bM16*solid.cE46)*solid.pzm1.bM44+(solid.pzm1.bM11*solid.cE15+solid.pzm1.bM12*solid.cE25+solid.pzm1.bM13*solid.cE35+solid.pzm1.bM14*solid.cE45+solid.pzm1.bM15*solid.cE55+solid.pzm1.bM16*solid.cE56)*solid.pzm1.bM45+(solid.pzm1.bM11*solid.cE16+solid.pzm1.bM12*solid.cE26+solid.pzm1.bM13*solid.cE36+solid.pzm1.bM14*solid.cE46+solid.pzm1.bM15*solid.cE56+solid.pzm1.bM16*solid.cE66)*solid.pzm1.bM46 | Pa | Elasticity matrix cE (global), Voigt notation, 14-component | Domain 5 |  |
| solid.cEg15 | (solid.pzm1.bM11*solid.cE11+solid.pzm1.bM12*solid.cE12+solid.pzm1.bM13*solid.cE13+solid.pzm1.bM14*solid.cE14+solid.pzm1.bM15*solid.cE15+solid.pzm1.bM16*solid.cE16)*solid.pzm1.bM51+(solid.pzm1.bM11*solid.cE12+solid.pzm1.bM12*solid.cE22+solid.pzm1.bM13*solid.cE23+solid.pzm1.bM14*solid.cE24+solid.pzm1.bM15*solid.cE25+solid.pzm1.bM16*solid.cE26)*solid.pzm1.bM52+(solid.pzm1.bM11*solid.cE13+solid.pzm1.bM12*solid.cE23+solid.pzm1.bM13*solid.cE33+solid.pzm1.bM14*solid.cE34+solid.pzm1.bM15*solid.cE35+solid.pzm1.bM16*solid.cE36)*solid.pzm1.bM53+(solid.pzm1.bM11*solid.cE14+solid.pzm1.bM12*solid.cE24+solid.pzm1.bM13*solid.cE34+solid.pzm1.bM14*solid.cE44+solid.pzm1.bM15*solid.cE45+solid.pzm1.bM16*solid.cE46)*solid.pzm1.bM54+(solid.pzm1.bM11*solid.cE15+solid.pzm1.bM12*solid.cE25+solid.pzm1.bM13*solid.cE35+solid.pzm1.bM14*solid.cE45+solid.pzm1.bM15*solid.cE55+solid.pzm1.bM16*solid.cE56)*solid.pzm1.bM55+(solid.pzm1.bM11*solid.cE16+solid.pzm1.bM12*solid.cE26+solid.pzm1.bM13*solid.cE36+solid.pzm1.bM14*solid.cE46+solid.pzm1.bM15*solid.cE56+solid.pzm1.bM16*solid.cE66)*solid.pzm1.bM56 | Pa | Elasticity matrix cE (global), Voigt notation, 15-component | Domain 5 |  |
| solid.cEg16 | (solid.pzm1.bM11*solid.cE11+solid.pzm1.bM12*solid.cE12+solid.pzm1.bM13*solid.cE13+solid.pzm1.bM14*solid.cE14+solid.pzm1.bM15*solid.cE15+solid.pzm1.bM16*solid.cE16)*solid.pzm1.bM61+(solid.pzm1.bM11*solid.cE12+solid.pzm1.bM12*solid.cE22+solid.pzm1.bM13*solid.cE23+solid.pzm1.bM14*solid.cE24+solid.pzm1.bM15*solid.cE25+solid.pzm1.bM16*solid.cE26)*solid.pzm1.bM62+(solid.pzm1.bM11*solid.cE13+solid.pzm1.bM12*solid.cE23+solid.pzm1.bM13*solid.cE33+solid.pzm1.bM14*solid.cE34+solid.pzm1.bM15*solid.cE35+solid.pzm1.bM16*solid.cE36)*solid.pzm1.bM63+(solid.pzm1.bM11*solid.cE14+solid.pzm1.bM12*solid.cE24+solid.pzm1.bM13*solid.cE34+solid.pzm1.bM14*solid.cE44+solid.pzm1.bM15*solid.cE45+solid.pzm1.bM16*solid.cE46)*solid.pzm1.bM64+(solid.pzm1.bM11*solid.cE15+solid.pzm1.bM12*solid.cE25+solid.pzm1.bM13*solid.cE35+solid.pzm1.bM14*solid.cE45+solid.pzm1.bM15*solid.cE55+solid.pzm1.bM16*solid.cE56)*solid.pzm1.bM65+(solid.pzm1.bM11*solid.cE16+solid.pzm1.bM12*solid.cE26+solid.pzm1.bM13*solid.cE36+solid.pzm1.bM14*solid.cE46+solid.pzm1.bM15*solid.cE56+solid.pzm1.bM16*solid.cE66)*solid.pzm1.bM66 | Pa | Elasticity matrix cE (global), Voigt notation, 16-component | Domain 5 |  |
| solid.cEg22 | (solid.pzm1.bM21*solid.cE11+solid.pzm1.bM22*solid.cE12+solid.pzm1.bM23*solid.cE13+solid.pzm1.bM24*solid.cE14+solid.pzm1.bM25*solid.cE15+solid.pzm1.bM26*solid.cE16)*solid.pzm1.bM21+(solid.pzm1.bM21*solid.cE12+solid.pzm1.bM22*solid.cE22+solid.pzm1.bM23*solid.cE23+solid.pzm1.bM24*solid.cE24+solid.pzm1.bM25*solid.cE25+solid.pzm1.bM26*solid.cE26)*solid.pzm1.bM22+(solid.pzm1.bM21*solid.cE13+solid.pzm1.bM22*solid.cE23+solid.pzm1.bM23*solid.cE33+solid.pzm1.bM24*solid.cE34+solid.pzm1.bM25*solid.cE35+solid.pzm1.bM26*solid.cE36)*solid.pzm1.bM23+(solid.pzm1.bM21*solid.cE14+solid.pzm1.bM22*solid.cE24+solid.pzm1.bM23*solid.cE34+solid.pzm1.bM24*solid.cE44+solid.pzm1.bM25*solid.cE45+solid.pzm1.bM26*solid.cE46)*solid.pzm1.bM24+(solid.pzm1.bM21*solid.cE15+solid.pzm1.bM22*solid.cE25+solid.pzm1.bM23*solid.cE35+solid.pzm1.bM24*solid.cE45+solid.pzm1.bM25*solid.cE55+solid.pzm1.bM26*solid.cE56)*solid.pzm1.bM25+(solid.pzm1.bM21*solid.cE16+solid.pzm1.bM22*solid.cE26+solid.pzm1.bM23*solid.cE36+solid.pzm1.bM24*solid.cE46+solid.pzm1.bM25*solid.cE56+solid.pzm1.bM26*solid.cE66)*solid.pzm1.bM26 | Pa | Elasticity matrix cE (global), Voigt notation, 22-component | Domain 5 |  |
| solid.cEg23 | (solid.pzm1.bM21*solid.cE11+solid.pzm1.bM22*solid.cE12+solid.pzm1.bM23*solid.cE13+solid.pzm1.bM24*solid.cE14+solid.pzm1.bM25*solid.cE15+solid.pzm1.bM26*solid.cE16)*solid.pzm1.bM31+(solid.pzm1.bM21*solid.cE12+solid.pzm1.bM22*solid.cE22+solid.pzm1.bM23*solid.cE23+solid.pzm1.bM24*solid.cE24+solid.pzm1.bM25*solid.cE25+solid.pzm1.bM26*solid.cE26)*solid.pzm1.bM32+(solid.pzm1.bM21*solid.cE13+solid.pzm1.bM22*solid.cE23+solid.pzm1.bM23*solid.cE33+solid.pzm1.bM24*solid.cE34+solid.pzm1.bM25*solid.cE35+solid.pzm1.bM26*solid.cE36)*solid.pzm1.bM33+(solid.pzm1.bM21*solid.cE14+solid.pzm1.bM22*solid.cE24+solid.pzm1.bM23*solid.cE34+solid.pzm1.bM24*solid.cE44+solid.pzm1.bM25*solid.cE45+solid.pzm1.bM26*solid.cE46)*solid.pzm1.bM34+(solid.pzm1.bM21*solid.cE15+solid.pzm1.bM22*solid.cE25+solid.pzm1.bM23*solid.cE35+solid.pzm1.bM24*solid.cE45+solid.pzm1.bM25*solid.cE55+solid.pzm1.bM26*solid.cE56)*solid.pzm1.bM35+(solid.pzm1.bM21*solid.cE16+solid.pzm1.bM22*solid.cE26+solid.pzm1.bM23*solid.cE36+solid.pzm1.bM24*solid.cE46+solid.pzm1.bM25*solid.cE56+solid.pzm1.bM26*solid.cE66)*solid.pzm1.bM36 | Pa | Elasticity matrix cE (global), Voigt notation, 23-component | Domain 5 |  |
| solid.cEg24 | (solid.pzm1.bM21*solid.cE11+solid.pzm1.bM22*solid.cE12+solid.pzm1.bM23*solid.cE13+solid.pzm1.bM24*solid.cE14+solid.pzm1.bM25*solid.cE15+solid.pzm1.bM26*solid.cE16)*solid.pzm1.bM41+(solid.pzm1.bM21*solid.cE12+solid.pzm1.bM22*solid.cE22+solid.pzm1.bM23*solid.cE23+solid.pzm1.bM24*solid.cE24+solid.pzm1.bM25*solid.cE25+solid.pzm1.bM26*solid.cE26)*solid.pzm1.bM42+(solid.pzm1.bM21*solid.cE13+solid.pzm1.bM22*solid.cE23+solid.pzm1.bM23*solid.cE33+solid.pzm1.bM24*solid.cE34+solid.pzm1.bM25*solid.cE35+solid.pzm1.bM26*solid.cE36)*solid.pzm1.bM43+(solid.pzm1.bM21*solid.cE14+solid.pzm1.bM22*solid.cE24+solid.pzm1.bM23*solid.cE34+solid.pzm1.bM24*solid.cE44+solid.pzm1.bM25*solid.cE45+solid.pzm1.bM26*solid.cE46)*solid.pzm1.bM44+(solid.pzm1.bM21*solid.cE15+solid.pzm1.bM22*solid.cE25+solid.pzm1.bM23*solid.cE35+solid.pzm1.bM24*solid.cE45+solid.pzm1.bM25*solid.cE55+solid.pzm1.bM26*solid.cE56)*solid.pzm1.bM45+(solid.pzm1.bM21*solid.cE16+solid.pzm1.bM22*solid.cE26+solid.pzm1.bM23*solid.cE36+solid.pzm1.bM24*solid.cE46+solid.pzm1.bM25*solid.cE56+solid.pzm1.bM26*solid.cE66)*solid.pzm1.bM46 | Pa | Elasticity matrix cE (global), Voigt notation, 24-component | Domain 5 |  |
| solid.cEg25 | (solid.pzm1.bM21*solid.cE11+solid.pzm1.bM22*solid.cE12+solid.pzm1.bM23*solid.cE13+solid.pzm1.bM24*solid.cE14+solid.pzm1.bM25*solid.cE15+solid.pzm1.bM26*solid.cE16)*solid.pzm1.bM51+(solid.pzm1.bM21*solid.cE12+solid.pzm1.bM22*solid.cE22+solid.pzm1.bM23*solid.cE23+solid.pzm1.bM24*solid.cE24+solid.pzm1.bM25*solid.cE25+solid.pzm1.bM26*solid.cE26)*solid.pzm1.bM52+(solid.pzm1.bM21*solid.cE13+solid.pzm1.bM22*solid.cE23+solid.pzm1.bM23*solid.cE33+solid.pzm1.bM24*solid.cE34+solid.pzm1.bM25*solid.cE35+solid.pzm1.bM26*solid.cE36)*solid.pzm1.bM53+(solid.pzm1.bM21*solid.cE14+solid.pzm1.bM22*solid.cE24+solid.pzm1.bM23*solid.cE34+solid.pzm1.bM24*solid.cE44+solid.pzm1.bM25*solid.cE45+solid.pzm1.bM26*solid.cE46)*solid.pzm1.bM54+(solid.pzm1.bM21*solid.cE15+solid.pzm1.bM22*solid.cE25+solid.pzm1.bM23*solid.cE35+solid.pzm1.bM24*solid.cE45+solid.pzm1.bM25*solid.cE55+solid.pzm1.bM26*solid.cE56)*solid.pzm1.bM55+(solid.pzm1.bM21*solid.cE16+solid.pzm1.bM22*solid.cE26+solid.pzm1.bM23*solid.cE36+solid.pzm1.bM24*solid.cE46+solid.pzm1.bM25*solid.cE56+solid.pzm1.bM26*solid.cE66)*solid.pzm1.bM56 | Pa | Elasticity matrix cE (global), Voigt notation, 25-component | Domain 5 |  |
| solid.cEg26 | (solid.pzm1.bM21*solid.cE11+solid.pzm1.bM22*solid.cE12+solid.pzm1.bM23*solid.cE13+solid.pzm1.bM24*solid.cE14+solid.pzm1.bM25*solid.cE15+solid.pzm1.bM26*solid.cE16)*solid.pzm1.bM61+(solid.pzm1.bM21*solid.cE12+solid.pzm1.bM22*solid.cE22+solid.pzm1.bM23*solid.cE23+solid.pzm1.bM24*solid.cE24+solid.pzm1.bM25*solid.cE25+solid.pzm1.bM26*solid.cE26)*solid.pzm1.bM62+(solid.pzm1.bM21*solid.cE13+solid.pzm1.bM22*solid.cE23+solid.pzm1.bM23*solid.cE33+solid.pzm1.bM24*solid.cE34+solid.pzm1.bM25*solid.cE35+solid.pzm1.bM26*solid.cE36)*solid.pzm1.bM63+(solid.pzm1.bM21*solid.cE14+solid.pzm1.bM22*solid.cE24+solid.pzm1.bM23*solid.cE34+solid.pzm1.bM24*solid.cE44+solid.pzm1.bM25*solid.cE45+solid.pzm1.bM26*solid.cE46)*solid.pzm1.bM64+(solid.pzm1.bM21*solid.cE15+solid.pzm1.bM22*solid.cE25+solid.pzm1.bM23*solid.cE35+solid.pzm1.bM24*solid.cE45+solid.pzm1.bM25*solid.cE55+solid.pzm1.bM26*solid.cE56)*solid.pzm1.bM65+(solid.pzm1.bM21*solid.cE16+solid.pzm1.bM22*solid.cE26+solid.pzm1.bM23*solid.cE36+solid.pzm1.bM24*solid.cE46+solid.pzm1.bM25*solid.cE56+solid.pzm1.bM26*solid.cE66)*solid.pzm1.bM66 | Pa | Elasticity matrix cE (global), Voigt notation, 26-component | Domain 5 |  |
| solid.cEg33 | (solid.pzm1.bM31*solid.cE11+solid.pzm1.bM32*solid.cE12+solid.pzm1.bM33*solid.cE13+solid.pzm1.bM34*solid.cE14+solid.pzm1.bM35*solid.cE15+solid.pzm1.bM36*solid.cE16)*solid.pzm1.bM31+(solid.pzm1.bM31*solid.cE12+solid.pzm1.bM32*solid.cE22+solid.pzm1.bM33*solid.cE23+solid.pzm1.bM34*solid.cE24+solid.pzm1.bM35*solid.cE25+solid.pzm1.bM36*solid.cE26)*solid.pzm1.bM32+(solid.pzm1.bM31*solid.cE13+solid.pzm1.bM32*solid.cE23+solid.pzm1.bM33*solid.cE33+solid.pzm1.bM34*solid.cE34+solid.pzm1.bM35*solid.cE35+solid.pzm1.bM36*solid.cE36)*solid.pzm1.bM33+(solid.pzm1.bM31*solid.cE14+solid.pzm1.bM32*solid.cE24+solid.pzm1.bM33*solid.cE34+solid.pzm1.bM34*solid.cE44+solid.pzm1.bM35*solid.cE45+solid.pzm1.bM36*solid.cE46)*solid.pzm1.bM34+(solid.pzm1.bM31*solid.cE15+solid.pzm1.bM32*solid.cE25+solid.pzm1.bM33*solid.cE35+solid.pzm1.bM34*solid.cE45+solid.pzm1.bM35*solid.cE55+solid.pzm1.bM36*solid.cE56)*solid.pzm1.bM35+(solid.pzm1.bM31*solid.cE16+solid.pzm1.bM32*solid.cE26+solid.pzm1.bM33*solid.cE36+solid.pzm1.bM34*solid.cE46+solid.pzm1.bM35*solid.cE56+solid.pzm1.bM36*solid.cE66)*solid.pzm1.bM36 | Pa | Elasticity matrix cE (global), Voigt notation, 33-component | Domain 5 |  |
| solid.cEg34 | (solid.pzm1.bM31*solid.cE11+solid.pzm1.bM32*solid.cE12+solid.pzm1.bM33*solid.cE13+solid.pzm1.bM34*solid.cE14+solid.pzm1.bM35*solid.cE15+solid.pzm1.bM36*solid.cE16)*solid.pzm1.bM41+(solid.pzm1.bM31*solid.cE12+solid.pzm1.bM32*solid.cE22+solid.pzm1.bM33*solid.cE23+solid.pzm1.bM34*solid.cE24+solid.pzm1.bM35*solid.cE25+solid.pzm1.bM36*solid.cE26)*solid.pzm1.bM42+(solid.pzm1.bM31*solid.cE13+solid.pzm1.bM32*solid.cE23+solid.pzm1.bM33*solid.cE33+solid.pzm1.bM34*solid.cE34+solid.pzm1.bM35*solid.cE35+solid.pzm1.bM36*solid.cE36)*solid.pzm1.bM43+(solid.pzm1.bM31*solid.cE14+solid.pzm1.bM32*solid.cE24+solid.pzm1.bM33*solid.cE34+solid.pzm1.bM34*solid.cE44+solid.pzm1.bM35*solid.cE45+solid.pzm1.bM36*solid.cE46)*solid.pzm1.bM44+(solid.pzm1.bM31*solid.cE15+solid.pzm1.bM32*solid.cE25+solid.pzm1.bM33*solid.cE35+solid.pzm1.bM34*solid.cE45+solid.pzm1.bM35*solid.cE55+solid.pzm1.bM36*solid.cE56)*solid.pzm1.bM45+(solid.pzm1.bM31*solid.cE16+solid.pzm1.bM32*solid.cE26+solid.pzm1.bM33*solid.cE36+solid.pzm1.bM34*solid.cE46+solid.pzm1.bM35*solid.cE56+solid.pzm1.bM36*solid.cE66)*solid.pzm1.bM46 | Pa | Elasticity matrix cE (global), Voigt notation, 34-component | Domain 5 |  |
| solid.cEg35 | (solid.pzm1.bM31*solid.cE11+solid.pzm1.bM32*solid.cE12+solid.pzm1.bM33*solid.cE13+solid.pzm1.bM34*solid.cE14+solid.pzm1.bM35*solid.cE15+solid.pzm1.bM36*solid.cE16)*solid.pzm1.bM51+(solid.pzm1.bM31*solid.cE12+solid.pzm1.bM32*solid.cE22+solid.pzm1.bM33*solid.cE23+solid.pzm1.bM34*solid.cE24+solid.pzm1.bM35*solid.cE25+solid.pzm1.bM36*solid.cE26)*solid.pzm1.bM52+(solid.pzm1.bM31*solid.cE13+solid.pzm1.bM32*solid.cE23+solid.pzm1.bM33*solid.cE33+solid.pzm1.bM34*solid.cE34+solid.pzm1.bM35*solid.cE35+solid.pzm1.bM36*solid.cE36)*solid.pzm1.bM53+(solid.pzm1.bM31*solid.cE14+solid.pzm1.bM32*solid.cE24+solid.pzm1.bM33*solid.cE34+solid.pzm1.bM34*solid.cE44+solid.pzm1.bM35*solid.cE45+solid.pzm1.bM36*solid.cE46)*solid.pzm1.bM54+(solid.pzm1.bM31*solid.cE15+solid.pzm1.bM32*solid.cE25+solid.pzm1.bM33*solid.cE35+solid.pzm1.bM34*solid.cE45+solid.pzm1.bM35*solid.cE55+solid.pzm1.bM36*solid.cE56)*solid.pzm1.bM55+(solid.pzm1.bM31*solid.cE16+solid.pzm1.bM32*solid.cE26+solid.pzm1.bM33*solid.cE36+solid.pzm1.bM34*solid.cE46+solid.pzm1.bM35*solid.cE56+solid.pzm1.bM36*solid.cE66)*solid.pzm1.bM56 | Pa | Elasticity matrix cE (global), Voigt notation, 35-component | Domain 5 |  |
| solid.cEg36 | (solid.pzm1.bM31*solid.cE11+solid.pzm1.bM32*solid.cE12+solid.pzm1.bM33*solid.cE13+solid.pzm1.bM34*solid.cE14+solid.pzm1.bM35*solid.cE15+solid.pzm1.bM36*solid.cE16)*solid.pzm1.bM61+(solid.pzm1.bM31*solid.cE12+solid.pzm1.bM32*solid.cE22+solid.pzm1.bM33*solid.cE23+solid.pzm1.bM34*solid.cE24+solid.pzm1.bM35*solid.cE25+solid.pzm1.bM36*solid.cE26)*solid.pzm1.bM62+(solid.pzm1.bM31*solid.cE13+solid.pzm1.bM32*solid.cE23+solid.pzm1.bM33*solid.cE33+solid.pzm1.bM34*solid.cE34+solid.pzm1.bM35*solid.cE35+solid.pzm1.bM36*solid.cE36)*solid.pzm1.bM63+(solid.pzm1.bM31*solid.cE14+solid.pzm1.bM32*solid.cE24+solid.pzm1.bM33*solid.cE34+solid.pzm1.bM34*solid.cE44+solid.pzm1.bM35*solid.cE45+solid.pzm1.bM36*solid.cE46)*solid.pzm1.bM64+(solid.pzm1.bM31*solid.cE15+solid.pzm1.bM32*solid.cE25+solid.pzm1.bM33*solid.cE35+solid.pzm1.bM34*solid.cE45+solid.pzm1.bM35*solid.cE55+solid.pzm1.bM36*solid.cE56)*solid.pzm1.bM65+(solid.pzm1.bM31*solid.cE16+solid.pzm1.bM32*solid.cE26+solid.pzm1.bM33*solid.cE36+solid.pzm1.bM34*solid.cE46+solid.pzm1.bM35*solid.cE56+solid.pzm1.bM36*solid.cE66)*solid.pzm1.bM66 | Pa | Elasticity matrix cE (global), Voigt notation, 36-component | Domain 5 |  |
| solid.cEg44 | (solid.pzm1.bM41*solid.cE11+solid.pzm1.bM42*solid.cE12+solid.pzm1.bM43*solid.cE13+solid.pzm1.bM44*solid.cE14+solid.pzm1.bM45*solid.cE15+solid.pzm1.bM46*solid.cE16)*solid.pzm1.bM41+(solid.pzm1.bM41*solid.cE12+solid.pzm1.bM42*solid.cE22+solid.pzm1.bM43*solid.cE23+solid.pzm1.bM44*solid.cE24+solid.pzm1.bM45*solid.cE25+solid.pzm1.bM46*solid.cE26)*solid.pzm1.bM42+(solid.pzm1.bM41*solid.cE13+solid.pzm1.bM42*solid.cE23+solid.pzm1.bM43*solid.cE33+solid.pzm1.bM44*solid.cE34+solid.pzm1.bM45*solid.cE35+solid.pzm1.bM46*solid.cE36)*solid.pzm1.bM43+(solid.pzm1.bM41*solid.cE14+solid.pzm1.bM42*solid.cE24+solid.pzm1.bM43*solid.cE34+solid.pzm1.bM44*solid.cE44+solid.pzm1.bM45*solid.cE45+solid.pzm1.bM46*solid.cE46)*solid.pzm1.bM44+(solid.pzm1.bM41*solid.cE15+solid.pzm1.bM42*solid.cE25+solid.pzm1.bM43*solid.cE35+solid.pzm1.bM44*solid.cE45+solid.pzm1.bM45*solid.cE55+solid.pzm1.bM46*solid.cE56)*solid.pzm1.bM45+(solid.pzm1.bM41*solid.cE16+solid.pzm1.bM42*solid.cE26+solid.pzm1.bM43*solid.cE36+solid.pzm1.bM44*solid.cE46+solid.pzm1.bM45*solid.cE56+solid.pzm1.bM46*solid.cE66)*solid.pzm1.bM46 | Pa | Elasticity matrix cE (global), Voigt notation, 44-component | Domain 5 |  |
| solid.cEg45 | (solid.pzm1.bM41*solid.cE11+solid.pzm1.bM42*solid.cE12+solid.pzm1.bM43*solid.cE13+solid.pzm1.bM44*solid.cE14+solid.pzm1.bM45*solid.cE15+solid.pzm1.bM46*solid.cE16)*solid.pzm1.bM51+(solid.pzm1.bM41*solid.cE12+solid.pzm1.bM42*solid.cE22+solid.pzm1.bM43*solid.cE23+solid.pzm1.bM44*solid.cE24+solid.pzm1.bM45*solid.cE25+solid.pzm1.bM46*solid.cE26)*solid.pzm1.bM52+(solid.pzm1.bM41*solid.cE13+solid.pzm1.bM42*solid.cE23+solid.pzm1.bM43*solid.cE33+solid.pzm1.bM44*solid.cE34+solid.pzm1.bM45*solid.cE35+solid.pzm1.bM46*solid.cE36)*solid.pzm1.bM53+(solid.pzm1.bM41*solid.cE14+solid.pzm1.bM42*solid.cE24+solid.pzm1.bM43*solid.cE34+solid.pzm1.bM44*solid.cE44+solid.pzm1.bM45*solid.cE45+solid.pzm1.bM46*solid.cE46)*solid.pzm1.bM54+(solid.pzm1.bM41*solid.cE15+solid.pzm1.bM42*solid.cE25+solid.pzm1.bM43*solid.cE35+solid.pzm1.bM44*solid.cE45+solid.pzm1.bM45*solid.cE55+solid.pzm1.bM46*solid.cE56)*solid.pzm1.bM55+(solid.pzm1.bM41*solid.cE16+solid.pzm1.bM42*solid.cE26+solid.pzm1.bM43*solid.cE36+solid.pzm1.bM44*solid.cE46+solid.pzm1.bM45*solid.cE56+solid.pzm1.bM46*solid.cE66)*solid.pzm1.bM56 | Pa | Elasticity matrix cE (global), Voigt notation, 45-component | Domain 5 |  |
| solid.cEg46 | (solid.pzm1.bM41*solid.cE11+solid.pzm1.bM42*solid.cE12+solid.pzm1.bM43*solid.cE13+solid.pzm1.bM44*solid.cE14+solid.pzm1.bM45*solid.cE15+solid.pzm1.bM46*solid.cE16)*solid.pzm1.bM61+(solid.pzm1.bM41*solid.cE12+solid.pzm1.bM42*solid.cE22+solid.pzm1.bM43*solid.cE23+solid.pzm1.bM44*solid.cE24+solid.pzm1.bM45*solid.cE25+solid.pzm1.bM46*solid.cE26)*solid.pzm1.bM62+(solid.pzm1.bM41*solid.cE13+solid.pzm1.bM42*solid.cE23+solid.pzm1.bM43*solid.cE33+solid.pzm1.bM44*solid.cE34+solid.pzm1.bM45*solid.cE35+solid.pzm1.bM46*solid.cE36)*solid.pzm1.bM63+(solid.pzm1.bM41*solid.cE14+solid.pzm1.bM42*solid.cE24+solid.pzm1.bM43*solid.cE34+solid.pzm1.bM44*solid.cE44+solid.pzm1.bM45*solid.cE45+solid.pzm1.bM46*solid.cE46)*solid.pzm1.bM64+(solid.pzm1.bM41*solid.cE15+solid.pzm1.bM42*solid.cE25+solid.pzm1.bM43*solid.cE35+solid.pzm1.bM44*solid.cE45+solid.pzm1.bM45*solid.cE55+solid.pzm1.bM46*solid.cE56)*solid.pzm1.bM65+(solid.pzm1.bM41*solid.cE16+solid.pzm1.bM42*solid.cE26+solid.pzm1.bM43*solid.cE36+solid.pzm1.bM44*solid.cE46+solid.pzm1.bM45*solid.cE56+solid.pzm1.bM46*solid.cE66)*solid.pzm1.bM66 | Pa | Elasticity matrix cE (global), Voigt notation, 46-component | Domain 5 |  |
| solid.cEg55 | (solid.pzm1.bM51*solid.cE11+solid.pzm1.bM52*solid.cE12+solid.pzm1.bM53*solid.cE13+solid.pzm1.bM54*solid.cE14+solid.pzm1.bM55*solid.cE15+solid.pzm1.bM56*solid.cE16)*solid.pzm1.bM51+(solid.pzm1.bM51*solid.cE12+solid.pzm1.bM52*solid.cE22+solid.pzm1.bM53*solid.cE23+solid.pzm1.bM54*solid.cE24+solid.pzm1.bM55*solid.cE25+solid.pzm1.bM56*solid.cE26)*solid.pzm1.bM52+(solid.pzm1.bM51*solid.cE13+solid.pzm1.bM52*solid.cE23+solid.pzm1.bM53*solid.cE33+solid.pzm1.bM54*solid.cE34+solid.pzm1.bM55*solid.cE35+solid.pzm1.bM56*solid.cE36)*solid.pzm1.bM53+(solid.pzm1.bM51*solid.cE14+solid.pzm1.bM52*solid.cE24+solid.pzm1.bM53*solid.cE34+solid.pzm1.bM54*solid.cE44+solid.pzm1.bM55*solid.cE45+solid.pzm1.bM56*solid.cE46)*solid.pzm1.bM54+(solid.pzm1.bM51*solid.cE15+solid.pzm1.bM52*solid.cE25+solid.pzm1.bM53*solid.cE35+solid.pzm1.bM54*solid.cE45+solid.pzm1.bM55*solid.cE55+solid.pzm1.bM56*solid.cE56)*solid.pzm1.bM55+(solid.pzm1.bM51*solid.cE16+solid.pzm1.bM52*solid.cE26+solid.pzm1.bM53*solid.cE36+solid.pzm1.bM54*solid.cE46+solid.pzm1.bM55*solid.cE56+solid.pzm1.bM56*solid.cE66)*solid.pzm1.bM56 | Pa | Elasticity matrix cE (global), Voigt notation, 55-component | Domain 5 |  |
| solid.cEg56 | (solid.pzm1.bM51*solid.cE11+solid.pzm1.bM52*solid.cE12+solid.pzm1.bM53*solid.cE13+solid.pzm1.bM54*solid.cE14+solid.pzm1.bM55*solid.cE15+solid.pzm1.bM56*solid.cE16)*solid.pzm1.bM61+(solid.pzm1.bM51*solid.cE12+solid.pzm1.bM52*solid.cE22+solid.pzm1.bM53*solid.cE23+solid.pzm1.bM54*solid.cE24+solid.pzm1.bM55*solid.cE25+solid.pzm1.bM56*solid.cE26)*solid.pzm1.bM62+(solid.pzm1.bM51*solid.cE13+solid.pzm1.bM52*solid.cE23+solid.pzm1.bM53*solid.cE33+solid.pzm1.bM54*solid.cE34+solid.pzm1.bM55*solid.cE35+solid.pzm1.bM56*solid.cE36)*solid.pzm1.bM63+(solid.pzm1.bM51*solid.cE14+solid.pzm1.bM52*solid.cE24+solid.pzm1.bM53*solid.cE34+solid.pzm1.bM54*solid.cE44+solid.pzm1.bM55*solid.cE45+solid.pzm1.bM56*solid.cE46)*solid.pzm1.bM64+(solid.pzm1.bM51*solid.cE15+solid.pzm1.bM52*solid.cE25+solid.pzm1.bM53*solid.cE35+solid.pzm1.bM54*solid.cE45+solid.pzm1.bM55*solid.cE55+solid.pzm1.bM56*solid.cE56)*solid.pzm1.bM65+(solid.pzm1.bM51*solid.cE16+solid.pzm1.bM52*solid.cE26+solid.pzm1.bM53*solid.cE36+solid.pzm1.bM54*solid.cE46+solid.pzm1.bM55*solid.cE56+solid.pzm1.bM56*solid.cE66)*solid.pzm1.bM66 | Pa | Elasticity matrix cE (global), Voigt notation, 56-component | Domain 5 |  |
| solid.cEg66 | (solid.pzm1.bM61*solid.cE11+solid.pzm1.bM62*solid.cE12+solid.pzm1.bM63*solid.cE13+solid.pzm1.bM64*solid.cE14+solid.pzm1.bM65*solid.cE15+solid.pzm1.bM66*solid.cE16)*solid.pzm1.bM61+(solid.pzm1.bM61*solid.cE12+solid.pzm1.bM62*solid.cE22+solid.pzm1.bM63*solid.cE23+solid.pzm1.bM64*solid.cE24+solid.pzm1.bM65*solid.cE25+solid.pzm1.bM66*solid.cE26)*solid.pzm1.bM62+(solid.pzm1.bM61*solid.cE13+solid.pzm1.bM62*solid.cE23+solid.pzm1.bM63*solid.cE33+solid.pzm1.bM64*solid.cE34+solid.pzm1.bM65*solid.cE35+solid.pzm1.bM66*solid.cE36)*solid.pzm1.bM63+(solid.pzm1.bM61*solid.cE14+solid.pzm1.bM62*solid.cE24+solid.pzm1.bM63*solid.cE34+solid.pzm1.bM64*solid.cE44+solid.pzm1.bM65*solid.cE45+solid.pzm1.bM66*solid.cE46)*solid.pzm1.bM64+(solid.pzm1.bM61*solid.cE15+solid.pzm1.bM62*solid.cE25+solid.pzm1.bM63*solid.cE35+solid.pzm1.bM64*solid.cE45+solid.pzm1.bM65*solid.cE55+solid.pzm1.bM66*solid.cE56)*solid.pzm1.bM65+(solid.pzm1.bM61*solid.cE16+solid.pzm1.bM62*solid.cE26+solid.pzm1.bM63*solid.cE36+solid.pzm1.bM64*solid.cE46+solid.pzm1.bM65*solid.cE56+solid.pzm1.bM66*solid.cE66)*solid.pzm1.bM66 | Pa | Elasticity matrix cE (global), Voigt notation, 66-component | Domain 5 |  |
| solid.sEg11 | (solid.pzm1.bN11*solid.sE11+solid.pzm1.bN12*solid.sE12+solid.pzm1.bN13*solid.sE13+solid.pzm1.bN14*solid.sE14+solid.pzm1.bN15*solid.sE15+solid.pzm1.bN16*solid.sE16)*solid.pzm1.bN11+(solid.pzm1.bN11*solid.sE12+solid.pzm1.bN12*solid.sE22+solid.pzm1.bN13*solid.sE23+solid.pzm1.bN14*solid.sE24+solid.pzm1.bN15*solid.sE25+solid.pzm1.bN16*solid.sE26)*solid.pzm1.bN12+(solid.pzm1.bN11*solid.sE13+solid.pzm1.bN12*solid.sE23+solid.pzm1.bN13*solid.sE33+solid.pzm1.bN14*solid.sE34+solid.pzm1.bN15*solid.sE35+solid.pzm1.bN16*solid.sE36)*solid.pzm1.bN13+(solid.pzm1.bN11*solid.sE14+solid.pzm1.bN12*solid.sE24+solid.pzm1.bN13*solid.sE34+solid.pzm1.bN14*solid.sE44+solid.pzm1.bN15*solid.sE45+solid.pzm1.bN16*solid.sE46)*solid.pzm1.bN14+(solid.pzm1.bN11*solid.sE15+solid.pzm1.bN12*solid.sE25+solid.pzm1.bN13*solid.sE35+solid.pzm1.bN14*solid.sE45+solid.pzm1.bN15*solid.sE55+solid.pzm1.bN16*solid.sE56)*solid.pzm1.bN15+(solid.pzm1.bN11*solid.sE16+solid.pzm1.bN12*solid.sE26+solid.pzm1.bN13*solid.sE36+solid.pzm1.bN14*solid.sE46+solid.pzm1.bN15*solid.sE56+solid.pzm1.bN16*solid.sE66)*solid.pzm1.bN16 | 1/Pa | Compliance matrix sE (global), Voigt notation, 11-component | Domain 5 |  |
| solid.sEg12 | (solid.pzm1.bN11*solid.sE11+solid.pzm1.bN12*solid.sE12+solid.pzm1.bN13*solid.sE13+solid.pzm1.bN14*solid.sE14+solid.pzm1.bN15*solid.sE15+solid.pzm1.bN16*solid.sE16)*solid.pzm1.bN21+(solid.pzm1.bN11*solid.sE12+solid.pzm1.bN12*solid.sE22+solid.pzm1.bN13*solid.sE23+solid.pzm1.bN14*solid.sE24+solid.pzm1.bN15*solid.sE25+solid.pzm1.bN16*solid.sE26)*solid.pzm1.bN22+(solid.pzm1.bN11*solid.sE13+solid.pzm1.bN12*solid.sE23+solid.pzm1.bN13*solid.sE33+solid.pzm1.bN14*solid.sE34+solid.pzm1.bN15*solid.sE35+solid.pzm1.bN16*solid.sE36)*solid.pzm1.bN23+(solid.pzm1.bN11*solid.sE14+solid.pzm1.bN12*solid.sE24+solid.pzm1.bN13*solid.sE34+solid.pzm1.bN14*solid.sE44+solid.pzm1.bN15*solid.sE45+solid.pzm1.bN16*solid.sE46)*solid.pzm1.bN24+(solid.pzm1.bN11*solid.sE15+solid.pzm1.bN12*solid.sE25+solid.pzm1.bN13*solid.sE35+solid.pzm1.bN14*solid.sE45+solid.pzm1.bN15*solid.sE55+solid.pzm1.bN16*solid.sE56)*solid.pzm1.bN25+(solid.pzm1.bN11*solid.sE16+solid.pzm1.bN12*solid.sE26+solid.pzm1.bN13*solid.sE36+solid.pzm1.bN14*solid.sE46+solid.pzm1.bN15*solid.sE56+solid.pzm1.bN16*solid.sE66)*solid.pzm1.bN26 | 1/Pa | Compliance matrix sE (global), Voigt notation, 12-component | Domain 5 |  |
| solid.sEg13 | (solid.pzm1.bN11*solid.sE11+solid.pzm1.bN12*solid.sE12+solid.pzm1.bN13*solid.sE13+solid.pzm1.bN14*solid.sE14+solid.pzm1.bN15*solid.sE15+solid.pzm1.bN16*solid.sE16)*solid.pzm1.bN31+(solid.pzm1.bN11*solid.sE12+solid.pzm1.bN12*solid.sE22+solid.pzm1.bN13*solid.sE23+solid.pzm1.bN14*solid.sE24+solid.pzm1.bN15*solid.sE25+solid.pzm1.bN16*solid.sE26)*solid.pzm1.bN32+(solid.pzm1.bN11*solid.sE13+solid.pzm1.bN12*solid.sE23+solid.pzm1.bN13*solid.sE33+solid.pzm1.bN14*solid.sE34+solid.pzm1.bN15*solid.sE35+solid.pzm1.bN16*solid.sE36)*solid.pzm1.bN33+(solid.pzm1.bN11*solid.sE14+solid.pzm1.bN12*solid.sE24+solid.pzm1.bN13*solid.sE34+solid.pzm1.bN14*solid.sE44+solid.pzm1.bN15*solid.sE45+solid.pzm1.bN16*solid.sE46)*solid.pzm1.bN34+(solid.pzm1.bN11*solid.sE15+solid.pzm1.bN12*solid.sE25+solid.pzm1.bN13*solid.sE35+solid.pzm1.bN14*solid.sE45+solid.pzm1.bN15*solid.sE55+solid.pzm1.bN16*solid.sE56)*solid.pzm1.bN35+(solid.pzm1.bN11*solid.sE16+solid.pzm1.bN12*solid.sE26+solid.pzm1.bN13*solid.sE36+solid.pzm1.bN14*solid.sE46+solid.pzm1.bN15*solid.sE56+solid.pzm1.bN16*solid.sE66)*solid.pzm1.bN36 | 1/Pa | Compliance matrix sE (global), Voigt notation, 13-component | Domain 5 |  |
| solid.sEg14 | (solid.pzm1.bN11*solid.sE11+solid.pzm1.bN12*solid.sE12+solid.pzm1.bN13*solid.sE13+solid.pzm1.bN14*solid.sE14+solid.pzm1.bN15*solid.sE15+solid.pzm1.bN16*solid.sE16)*solid.pzm1.bN41+(solid.pzm1.bN11*solid.sE12+solid.pzm1.bN12*solid.sE22+solid.pzm1.bN13*solid.sE23+solid.pzm1.bN14*solid.sE24+solid.pzm1.bN15*solid.sE25+solid.pzm1.bN16*solid.sE26)*solid.pzm1.bN42+(solid.pzm1.bN11*solid.sE13+solid.pzm1.bN12*solid.sE23+solid.pzm1.bN13*solid.sE33+solid.pzm1.bN14*solid.sE34+solid.pzm1.bN15*solid.sE35+solid.pzm1.bN16*solid.sE36)*solid.pzm1.bN43+(solid.pzm1.bN11*solid.sE14+solid.pzm1.bN12*solid.sE24+solid.pzm1.bN13*solid.sE34+solid.pzm1.bN14*solid.sE44+solid.pzm1.bN15*solid.sE45+solid.pzm1.bN16*solid.sE46)*solid.pzm1.bN44+(solid.pzm1.bN11*solid.sE15+solid.pzm1.bN12*solid.sE25+solid.pzm1.bN13*solid.sE35+solid.pzm1.bN14*solid.sE45+solid.pzm1.bN15*solid.sE55+solid.pzm1.bN16*solid.sE56)*solid.pzm1.bN45+(solid.pzm1.bN11*solid.sE16+solid.pzm1.bN12*solid.sE26+solid.pzm1.bN13*solid.sE36+solid.pzm1.bN14*solid.sE46+solid.pzm1.bN15*solid.sE56+solid.pzm1.bN16*solid.sE66)*solid.pzm1.bN46 | 1/Pa | Compliance matrix sE (global), Voigt notation, 14-component | Domain 5 |  |
| solid.sEg15 | (solid.pzm1.bN11*solid.sE11+solid.pzm1.bN12*solid.sE12+solid.pzm1.bN13*solid.sE13+solid.pzm1.bN14*solid.sE14+solid.pzm1.bN15*solid.sE15+solid.pzm1.bN16*solid.sE16)*solid.pzm1.bN51+(solid.pzm1.bN11*solid.sE12+solid.pzm1.bN12*solid.sE22+solid.pzm1.bN13*solid.sE23+solid.pzm1.bN14*solid.sE24+solid.pzm1.bN15*solid.sE25+solid.pzm1.bN16*solid.sE26)*solid.pzm1.bN52+(solid.pzm1.bN11*solid.sE13+solid.pzm1.bN12*solid.sE23+solid.pzm1.bN13*solid.sE33+solid.pzm1.bN14*solid.sE34+solid.pzm1.bN15*solid.sE35+solid.pzm1.bN16*solid.sE36)*solid.pzm1.bN53+(solid.pzm1.bN11*solid.sE14+solid.pzm1.bN12*solid.sE24+solid.pzm1.bN13*solid.sE34+solid.pzm1.bN14*solid.sE44+solid.pzm1.bN15*solid.sE45+solid.pzm1.bN16*solid.sE46)*solid.pzm1.bN54+(solid.pzm1.bN11*solid.sE15+solid.pzm1.bN12*solid.sE25+solid.pzm1.bN13*solid.sE35+solid.pzm1.bN14*solid.sE45+solid.pzm1.bN15*solid.sE55+solid.pzm1.bN16*solid.sE56)*solid.pzm1.bN55+(solid.pzm1.bN11*solid.sE16+solid.pzm1.bN12*solid.sE26+solid.pzm1.bN13*solid.sE36+solid.pzm1.bN14*solid.sE46+solid.pzm1.bN15*solid.sE56+solid.pzm1.bN16*solid.sE66)*solid.pzm1.bN56 | 1/Pa | Compliance matrix sE (global), Voigt notation, 15-component | Domain 5 |  |
| solid.sEg16 | (solid.pzm1.bN11*solid.sE11+solid.pzm1.bN12*solid.sE12+solid.pzm1.bN13*solid.sE13+solid.pzm1.bN14*solid.sE14+solid.pzm1.bN15*solid.sE15+solid.pzm1.bN16*solid.sE16)*solid.pzm1.bN61+(solid.pzm1.bN11*solid.sE12+solid.pzm1.bN12*solid.sE22+solid.pzm1.bN13*solid.sE23+solid.pzm1.bN14*solid.sE24+solid.pzm1.bN15*solid.sE25+solid.pzm1.bN16*solid.sE26)*solid.pzm1.bN62+(solid.pzm1.bN11*solid.sE13+solid.pzm1.bN12*solid.sE23+solid.pzm1.bN13*solid.sE33+solid.pzm1.bN14*solid.sE34+solid.pzm1.bN15*solid.sE35+solid.pzm1.bN16*solid.sE36)*solid.pzm1.bN63+(solid.pzm1.bN11*solid.sE14+solid.pzm1.bN12*solid.sE24+solid.pzm1.bN13*solid.sE34+solid.pzm1.bN14*solid.sE44+solid.pzm1.bN15*solid.sE45+solid.pzm1.bN16*solid.sE46)*solid.pzm1.bN64+(solid.pzm1.bN11*solid.sE15+solid.pzm1.bN12*solid.sE25+solid.pzm1.bN13*solid.sE35+solid.pzm1.bN14*solid.sE45+solid.pzm1.bN15*solid.sE55+solid.pzm1.bN16*solid.sE56)*solid.pzm1.bN65+(solid.pzm1.bN11*solid.sE16+solid.pzm1.bN12*solid.sE26+solid.pzm1.bN13*solid.sE36+solid.pzm1.bN14*solid.sE46+solid.pzm1.bN15*solid.sE56+solid.pzm1.bN16*solid.sE66)*solid.pzm1.bN66 | 1/Pa | Compliance matrix sE (global), Voigt notation, 16-component | Domain 5 |  |
| solid.sEg22 | (solid.pzm1.bN21*solid.sE11+solid.pzm1.bN22*solid.sE12+solid.pzm1.bN23*solid.sE13+solid.pzm1.bN24*solid.sE14+solid.pzm1.bN25*solid.sE15+solid.pzm1.bN26*solid.sE16)*solid.pzm1.bN21+(solid.pzm1.bN21*solid.sE12+solid.pzm1.bN22*solid.sE22+solid.pzm1.bN23*solid.sE23+solid.pzm1.bN24*solid.sE24+solid.pzm1.bN25*solid.sE25+solid.pzm1.bN26*solid.sE26)*solid.pzm1.bN22+(solid.pzm1.bN21*solid.sE13+solid.pzm1.bN22*solid.sE23+solid.pzm1.bN23*solid.sE33+solid.pzm1.bN24*solid.sE34+solid.pzm1.bN25*solid.sE35+solid.pzm1.bN26*solid.sE36)*solid.pzm1.bN23+(solid.pzm1.bN21*solid.sE14+solid.pzm1.bN22*solid.sE24+solid.pzm1.bN23*solid.sE34+solid.pzm1.bN24*solid.sE44+solid.pzm1.bN25*solid.sE45+solid.pzm1.bN26*solid.sE46)*solid.pzm1.bN24+(solid.pzm1.bN21*solid.sE15+solid.pzm1.bN22*solid.sE25+solid.pzm1.bN23*solid.sE35+solid.pzm1.bN24*solid.sE45+solid.pzm1.bN25*solid.sE55+solid.pzm1.bN26*solid.sE56)*solid.pzm1.bN25+(solid.pzm1.bN21*solid.sE16+solid.pzm1.bN22*solid.sE26+solid.pzm1.bN23*solid.sE36+solid.pzm1.bN24*solid.sE46+solid.pzm1.bN25*solid.sE56+solid.pzm1.bN26*solid.sE66)*solid.pzm1.bN26 | 1/Pa | Compliance matrix sE (global), Voigt notation, 22-component | Domain 5 |  |
| solid.sEg23 | (solid.pzm1.bN21*solid.sE11+solid.pzm1.bN22*solid.sE12+solid.pzm1.bN23*solid.sE13+solid.pzm1.bN24*solid.sE14+solid.pzm1.bN25*solid.sE15+solid.pzm1.bN26*solid.sE16)*solid.pzm1.bN31+(solid.pzm1.bN21*solid.sE12+solid.pzm1.bN22*solid.sE22+solid.pzm1.bN23*solid.sE23+solid.pzm1.bN24*solid.sE24+solid.pzm1.bN25*solid.sE25+solid.pzm1.bN26*solid.sE26)*solid.pzm1.bN32+(solid.pzm1.bN21*solid.sE13+solid.pzm1.bN22*solid.sE23+solid.pzm1.bN23*solid.sE33+solid.pzm1.bN24*solid.sE34+solid.pzm1.bN25*solid.sE35+solid.pzm1.bN26*solid.sE36)*solid.pzm1.bN33+(solid.pzm1.bN21*solid.sE14+solid.pzm1.bN22*solid.sE24+solid.pzm1.bN23*solid.sE34+solid.pzm1.bN24*solid.sE44+solid.pzm1.bN25*solid.sE45+solid.pzm1.bN26*solid.sE46)*solid.pzm1.bN34+(solid.pzm1.bN21*solid.sE15+solid.pzm1.bN22*solid.sE25+solid.pzm1.bN23*solid.sE35+solid.pzm1.bN24*solid.sE45+solid.pzm1.bN25*solid.sE55+solid.pzm1.bN26*solid.sE56)*solid.pzm1.bN35+(solid.pzm1.bN21*solid.sE16+solid.pzm1.bN22*solid.sE26+solid.pzm1.bN23*solid.sE36+solid.pzm1.bN24*solid.sE46+solid.pzm1.bN25*solid.sE56+solid.pzm1.bN26*solid.sE66)*solid.pzm1.bN36 | 1/Pa | Compliance matrix sE (global), Voigt notation, 23-component | Domain 5 |  |
| solid.sEg24 | (solid.pzm1.bN21*solid.sE11+solid.pzm1.bN22*solid.sE12+solid.pzm1.bN23*solid.sE13+solid.pzm1.bN24*solid.sE14+solid.pzm1.bN25*solid.sE15+solid.pzm1.bN26*solid.sE16)*solid.pzm1.bN41+(solid.pzm1.bN21*solid.sE12+solid.pzm1.bN22*solid.sE22+solid.pzm1.bN23*solid.sE23+solid.pzm1.bN24*solid.sE24+solid.pzm1.bN25*solid.sE25+solid.pzm1.bN26*solid.sE26)*solid.pzm1.bN42+(solid.pzm1.bN21*solid.sE13+solid.pzm1.bN22*solid.sE23+solid.pzm1.bN23*solid.sE33+solid.pzm1.bN24*solid.sE34+solid.pzm1.bN25*solid.sE35+solid.pzm1.bN26*solid.sE36)*solid.pzm1.bN43+(solid.pzm1.bN21*solid.sE14+solid.pzm1.bN22*solid.sE24+solid.pzm1.bN23*solid.sE34+solid.pzm1.bN24*solid.sE44+solid.pzm1.bN25*solid.sE45+solid.pzm1.bN26*solid.sE46)*solid.pzm1.bN44+(solid.pzm1.bN21*solid.sE15+solid.pzm1.bN22*solid.sE25+solid.pzm1.bN23*solid.sE35+solid.pzm1.bN24*solid.sE45+solid.pzm1.bN25*solid.sE55+solid.pzm1.bN26*solid.sE56)*solid.pzm1.bN45+(solid.pzm1.bN21*solid.sE16+solid.pzm1.bN22*solid.sE26+solid.pzm1.bN23*solid.sE36+solid.pzm1.bN24*solid.sE46+solid.pzm1.bN25*solid.sE56+solid.pzm1.bN26*solid.sE66)*solid.pzm1.bN46 | 1/Pa | Compliance matrix sE (global), Voigt notation, 24-component | Domain 5 |  |
| solid.sEg25 | (solid.pzm1.bN21*solid.sE11+solid.pzm1.bN22*solid.sE12+solid.pzm1.bN23*solid.sE13+solid.pzm1.bN24*solid.sE14+solid.pzm1.bN25*solid.sE15+solid.pzm1.bN26*solid.sE16)*solid.pzm1.bN51+(solid.pzm1.bN21*solid.sE12+solid.pzm1.bN22*solid.sE22+solid.pzm1.bN23*solid.sE23+solid.pzm1.bN24*solid.sE24+solid.pzm1.bN25*solid.sE25+solid.pzm1.bN26*solid.sE26)*solid.pzm1.bN52+(solid.pzm1.bN21*solid.sE13+solid.pzm1.bN22*solid.sE23+solid.pzm1.bN23*solid.sE33+solid.pzm1.bN24*solid.sE34+solid.pzm1.bN25*solid.sE35+solid.pzm1.bN26*solid.sE36)*solid.pzm1.bN53+(solid.pzm1.bN21*solid.sE14+solid.pzm1.bN22*solid.sE24+solid.pzm1.bN23*solid.sE34+solid.pzm1.bN24*solid.sE44+solid.pzm1.bN25*solid.sE45+solid.pzm1.bN26*solid.sE46)*solid.pzm1.bN54+(solid.pzm1.bN21*solid.sE15+solid.pzm1.bN22*solid.sE25+solid.pzm1.bN23*solid.sE35+solid.pzm1.bN24*solid.sE45+solid.pzm1.bN25*solid.sE55+solid.pzm1.bN26*solid.sE56)*solid.pzm1.bN55+(solid.pzm1.bN21*solid.sE16+solid.pzm1.bN22*solid.sE26+solid.pzm1.bN23*solid.sE36+solid.pzm1.bN24*solid.sE46+solid.pzm1.bN25*solid.sE56+solid.pzm1.bN26*solid.sE66)*solid.pzm1.bN56 | 1/Pa | Compliance matrix sE (global), Voigt notation, 25-component | Domain 5 |  |
| solid.sEg26 | (solid.pzm1.bN21*solid.sE11+solid.pzm1.bN22*solid.sE12+solid.pzm1.bN23*solid.sE13+solid.pzm1.bN24*solid.sE14+solid.pzm1.bN25*solid.sE15+solid.pzm1.bN26*solid.sE16)*solid.pzm1.bN61+(solid.pzm1.bN21*solid.sE12+solid.pzm1.bN22*solid.sE22+solid.pzm1.bN23*solid.sE23+solid.pzm1.bN24*solid.sE24+solid.pzm1.bN25*solid.sE25+solid.pzm1.bN26*solid.sE26)*solid.pzm1.bN62+(solid.pzm1.bN21*solid.sE13+solid.pzm1.bN22*solid.sE23+solid.pzm1.bN23*solid.sE33+solid.pzm1.bN24*solid.sE34+solid.pzm1.bN25*solid.sE35+solid.pzm1.bN26*solid.sE36)*solid.pzm1.bN63+(solid.pzm1.bN21*solid.sE14+solid.pzm1.bN22*solid.sE24+solid.pzm1.bN23*solid.sE34+solid.pzm1.bN24*solid.sE44+solid.pzm1.bN25*solid.sE45+solid.pzm1.bN26*solid.sE46)*solid.pzm1.bN64+(solid.pzm1.bN21*solid.sE15+solid.pzm1.bN22*solid.sE25+solid.pzm1.bN23*solid.sE35+solid.pzm1.bN24*solid.sE45+solid.pzm1.bN25*solid.sE55+solid.pzm1.bN26*solid.sE56)*solid.pzm1.bN65+(solid.pzm1.bN21*solid.sE16+solid.pzm1.bN22*solid.sE26+solid.pzm1.bN23*solid.sE36+solid.pzm1.bN24*solid.sE46+solid.pzm1.bN25*solid.sE56+solid.pzm1.bN26*solid.sE66)*solid.pzm1.bN66 | 1/Pa | Compliance matrix sE (global), Voigt notation, 26-component | Domain 5 |  |
| solid.sEg33 | (solid.pzm1.bN31*solid.sE11+solid.pzm1.bN32*solid.sE12+solid.pzm1.bN33*solid.sE13+solid.pzm1.bN34*solid.sE14+solid.pzm1.bN35*solid.sE15+solid.pzm1.bN36*solid.sE16)*solid.pzm1.bN31+(solid.pzm1.bN31*solid.sE12+solid.pzm1.bN32*solid.sE22+solid.pzm1.bN33*solid.sE23+solid.pzm1.bN34*solid.sE24+solid.pzm1.bN35*solid.sE25+solid.pzm1.bN36*solid.sE26)*solid.pzm1.bN32+(solid.pzm1.bN31*solid.sE13+solid.pzm1.bN32*solid.sE23+solid.pzm1.bN33*solid.sE33+solid.pzm1.bN34*solid.sE34+solid.pzm1.bN35*solid.sE35+solid.pzm1.bN36*solid.sE36)*solid.pzm1.bN33+(solid.pzm1.bN31*solid.sE14+solid.pzm1.bN32*solid.sE24+solid.pzm1.bN33*solid.sE34+solid.pzm1.bN34*solid.sE44+solid.pzm1.bN35*solid.sE45+solid.pzm1.bN36*solid.sE46)*solid.pzm1.bN34+(solid.pzm1.bN31*solid.sE15+solid.pzm1.bN32*solid.sE25+solid.pzm1.bN33*solid.sE35+solid.pzm1.bN34*solid.sE45+solid.pzm1.bN35*solid.sE55+solid.pzm1.bN36*solid.sE56)*solid.pzm1.bN35+(solid.pzm1.bN31*solid.sE16+solid.pzm1.bN32*solid.sE26+solid.pzm1.bN33*solid.sE36+solid.pzm1.bN34*solid.sE46+solid.pzm1.bN35*solid.sE56+solid.pzm1.bN36*solid.sE66)*solid.pzm1.bN36 | 1/Pa | Compliance matrix sE (global), Voigt notation, 33-component | Domain 5 |  |
| solid.sEg34 | (solid.pzm1.bN31*solid.sE11+solid.pzm1.bN32*solid.sE12+solid.pzm1.bN33*solid.sE13+solid.pzm1.bN34*solid.sE14+solid.pzm1.bN35*solid.sE15+solid.pzm1.bN36*solid.sE16)*solid.pzm1.bN41+(solid.pzm1.bN31*solid.sE12+solid.pzm1.bN32*solid.sE22+solid.pzm1.bN33*solid.sE23+solid.pzm1.bN34*solid.sE24+solid.pzm1.bN35*solid.sE25+solid.pzm1.bN36*solid.sE26)*solid.pzm1.bN42+(solid.pzm1.bN31*solid.sE13+solid.pzm1.bN32*solid.sE23+solid.pzm1.bN33*solid.sE33+solid.pzm1.bN34*solid.sE34+solid.pzm1.bN35*solid.sE35+solid.pzm1.bN36*solid.sE36)*solid.pzm1.bN43+(solid.pzm1.bN31*solid.sE14+solid.pzm1.bN32*solid.sE24+solid.pzm1.bN33*solid.sE34+solid.pzm1.bN34*solid.sE44+solid.pzm1.bN35*solid.sE45+solid.pzm1.bN36*solid.sE46)*solid.pzm1.bN44+(solid.pzm1.bN31*solid.sE15+solid.pzm1.bN32*solid.sE25+solid.pzm1.bN33*solid.sE35+solid.pzm1.bN34*solid.sE45+solid.pzm1.bN35*solid.sE55+solid.pzm1.bN36*solid.sE56)*solid.pzm1.bN45+(solid.pzm1.bN31*solid.sE16+solid.pzm1.bN32*solid.sE26+solid.pzm1.bN33*solid.sE36+solid.pzm1.bN34*solid.sE46+solid.pzm1.bN35*solid.sE56+solid.pzm1.bN36*solid.sE66)*solid.pzm1.bN46 | 1/Pa | Compliance matrix sE (global), Voigt notation, 34-component | Domain 5 |  |
| solid.sEg35 | (solid.pzm1.bN31*solid.sE11+solid.pzm1.bN32*solid.sE12+solid.pzm1.bN33*solid.sE13+solid.pzm1.bN34*solid.sE14+solid.pzm1.bN35*solid.sE15+solid.pzm1.bN36*solid.sE16)*solid.pzm1.bN51+(solid.pzm1.bN31*solid.sE12+solid.pzm1.bN32*solid.sE22+solid.pzm1.bN33*solid.sE23+solid.pzm1.bN34*solid.sE24+solid.pzm1.bN35*solid.sE25+solid.pzm1.bN36*solid.sE26)*solid.pzm1.bN52+(solid.pzm1.bN31*solid.sE13+solid.pzm1.bN32*solid.sE23+solid.pzm1.bN33*solid.sE33+solid.pzm1.bN34*solid.sE34+solid.pzm1.bN35*solid.sE35+solid.pzm1.bN36*solid.sE36)*solid.pzm1.bN53+(solid.pzm1.bN31*solid.sE14+solid.pzm1.bN32*solid.sE24+solid.pzm1.bN33*solid.sE34+solid.pzm1.bN34*solid.sE44+solid.pzm1.bN35*solid.sE45+solid.pzm1.bN36*solid.sE46)*solid.pzm1.bN54+(solid.pzm1.bN31*solid.sE15+solid.pzm1.bN32*solid.sE25+solid.pzm1.bN33*solid.sE35+solid.pzm1.bN34*solid.sE45+solid.pzm1.bN35*solid.sE55+solid.pzm1.bN36*solid.sE56)*solid.pzm1.bN55+(solid.pzm1.bN31*solid.sE16+solid.pzm1.bN32*solid.sE26+solid.pzm1.bN33*solid.sE36+solid.pzm1.bN34*solid.sE46+solid.pzm1.bN35*solid.sE56+solid.pzm1.bN36*solid.sE66)*solid.pzm1.bN56 | 1/Pa | Compliance matrix sE (global), Voigt notation, 35-component | Domain 5 |  |
| solid.sEg36 | (solid.pzm1.bN31*solid.sE11+solid.pzm1.bN32*solid.sE12+solid.pzm1.bN33*solid.sE13+solid.pzm1.bN34*solid.sE14+solid.pzm1.bN35*solid.sE15+solid.pzm1.bN36*solid.sE16)*solid.pzm1.bN61+(solid.pzm1.bN31*solid.sE12+solid.pzm1.bN32*solid.sE22+solid.pzm1.bN33*solid.sE23+solid.pzm1.bN34*solid.sE24+solid.pzm1.bN35*solid.sE25+solid.pzm1.bN36*solid.sE26)*solid.pzm1.bN62+(solid.pzm1.bN31*solid.sE13+solid.pzm1.bN32*solid.sE23+solid.pzm1.bN33*solid.sE33+solid.pzm1.bN34*solid.sE34+solid.pzm1.bN35*solid.sE35+solid.pzm1.bN36*solid.sE36)*solid.pzm1.bN63+(solid.pzm1.bN31*solid.sE14+solid.pzm1.bN32*solid.sE24+solid.pzm1.bN33*solid.sE34+solid.pzm1.bN34*solid.sE44+solid.pzm1.bN35*solid.sE45+solid.pzm1.bN36*solid.sE46)*solid.pzm1.bN64+(solid.pzm1.bN31*solid.sE15+solid.pzm1.bN32*solid.sE25+solid.pzm1.bN33*solid.sE35+solid.pzm1.bN34*solid.sE45+solid.pzm1.bN35*solid.sE55+solid.pzm1.bN36*solid.sE56)*solid.pzm1.bN65+(solid.pzm1.bN31*solid.sE16+solid.pzm1.bN32*solid.sE26+solid.pzm1.bN33*solid.sE36+solid.pzm1.bN34*solid.sE46+solid.pzm1.bN35*solid.sE56+solid.pzm1.bN36*solid.sE66)*solid.pzm1.bN66 | 1/Pa | Compliance matrix sE (global), Voigt notation, 36-component | Domain 5 |  |
| solid.sEg44 | (solid.pzm1.bN41*solid.sE11+solid.pzm1.bN42*solid.sE12+solid.pzm1.bN43*solid.sE13+solid.pzm1.bN44*solid.sE14+solid.pzm1.bN45*solid.sE15+solid.pzm1.bN46*solid.sE16)*solid.pzm1.bN41+(solid.pzm1.bN41*solid.sE12+solid.pzm1.bN42*solid.sE22+solid.pzm1.bN43*solid.sE23+solid.pzm1.bN44*solid.sE24+solid.pzm1.bN45*solid.sE25+solid.pzm1.bN46*solid.sE26)*solid.pzm1.bN42+(solid.pzm1.bN41*solid.sE13+solid.pzm1.bN42*solid.sE23+solid.pzm1.bN43*solid.sE33+solid.pzm1.bN44*solid.sE34+solid.pzm1.bN45*solid.sE35+solid.pzm1.bN46*solid.sE36)*solid.pzm1.bN43+(solid.pzm1.bN41*solid.sE14+solid.pzm1.bN42*solid.sE24+solid.pzm1.bN43*solid.sE34+solid.pzm1.bN44*solid.sE44+solid.pzm1.bN45*solid.sE45+solid.pzm1.bN46*solid.sE46)*solid.pzm1.bN44+(solid.pzm1.bN41*solid.sE15+solid.pzm1.bN42*solid.sE25+solid.pzm1.bN43*solid.sE35+solid.pzm1.bN44*solid.sE45+solid.pzm1.bN45*solid.sE55+solid.pzm1.bN46*solid.sE56)*solid.pzm1.bN45+(solid.pzm1.bN41*solid.sE16+solid.pzm1.bN42*solid.sE26+solid.pzm1.bN43*solid.sE36+solid.pzm1.bN44*solid.sE46+solid.pzm1.bN45*solid.sE56+solid.pzm1.bN46*solid.sE66)*solid.pzm1.bN46 | 1/Pa | Compliance matrix sE (global), Voigt notation, 44-component | Domain 5 |  |
| solid.sEg45 | (solid.pzm1.bN41*solid.sE11+solid.pzm1.bN42*solid.sE12+solid.pzm1.bN43*solid.sE13+solid.pzm1.bN44*solid.sE14+solid.pzm1.bN45*solid.sE15+solid.pzm1.bN46*solid.sE16)*solid.pzm1.bN51+(solid.pzm1.bN41*solid.sE12+solid.pzm1.bN42*solid.sE22+solid.pzm1.bN43*solid.sE23+solid.pzm1.bN44*solid.sE24+solid.pzm1.bN45*solid.sE25+solid.pzm1.bN46*solid.sE26)*solid.pzm1.bN52+(solid.pzm1.bN41*solid.sE13+solid.pzm1.bN42*solid.sE23+solid.pzm1.bN43*solid.sE33+solid.pzm1.bN44*solid.sE34+solid.pzm1.bN45*solid.sE35+solid.pzm1.bN46*solid.sE36)*solid.pzm1.bN53+(solid.pzm1.bN41*solid.sE14+solid.pzm1.bN42*solid.sE24+solid.pzm1.bN43*solid.sE34+solid.pzm1.bN44*solid.sE44+solid.pzm1.bN45*solid.sE45+solid.pzm1.bN46*solid.sE46)*solid.pzm1.bN54+(solid.pzm1.bN41*solid.sE15+solid.pzm1.bN42*solid.sE25+solid.pzm1.bN43*solid.sE35+solid.pzm1.bN44*solid.sE45+solid.pzm1.bN45*solid.sE55+solid.pzm1.bN46*solid.sE56)*solid.pzm1.bN55+(solid.pzm1.bN41*solid.sE16+solid.pzm1.bN42*solid.sE26+solid.pzm1.bN43*solid.sE36+solid.pzm1.bN44*solid.sE46+solid.pzm1.bN45*solid.sE56+solid.pzm1.bN46*solid.sE66)*solid.pzm1.bN56 | 1/Pa | Compliance matrix sE (global), Voigt notation, 45-component | Domain 5 |  |
| solid.sEg46 | (solid.pzm1.bN41*solid.sE11+solid.pzm1.bN42*solid.sE12+solid.pzm1.bN43*solid.sE13+solid.pzm1.bN44*solid.sE14+solid.pzm1.bN45*solid.sE15+solid.pzm1.bN46*solid.sE16)*solid.pzm1.bN61+(solid.pzm1.bN41*solid.sE12+solid.pzm1.bN42*solid.sE22+solid.pzm1.bN43*solid.sE23+solid.pzm1.bN44*solid.sE24+solid.pzm1.bN45*solid.sE25+solid.pzm1.bN46*solid.sE26)*solid.pzm1.bN62+(solid.pzm1.bN41*solid.sE13+solid.pzm1.bN42*solid.sE23+solid.pzm1.bN43*solid.sE33+solid.pzm1.bN44*solid.sE34+solid.pzm1.bN45*solid.sE35+solid.pzm1.bN46*solid.sE36)*solid.pzm1.bN63+(solid.pzm1.bN41*solid.sE14+solid.pzm1.bN42*solid.sE24+solid.pzm1.bN43*solid.sE34+solid.pzm1.bN44*solid.sE44+solid.pzm1.bN45*solid.sE45+solid.pzm1.bN46*solid.sE46)*solid.pzm1.bN64+(solid.pzm1.bN41*solid.sE15+solid.pzm1.bN42*solid.sE25+solid.pzm1.bN43*solid.sE35+solid.pzm1.bN44*solid.sE45+solid.pzm1.bN45*solid.sE55+solid.pzm1.bN46*solid.sE56)*solid.pzm1.bN65+(solid.pzm1.bN41*solid.sE16+solid.pzm1.bN42*solid.sE26+solid.pzm1.bN43*solid.sE36+solid.pzm1.bN44*solid.sE46+solid.pzm1.bN45*solid.sE56+solid.pzm1.bN46*solid.sE66)*solid.pzm1.bN66 | 1/Pa | Compliance matrix sE (global), Voigt notation, 46-component | Domain 5 |  |
| solid.sEg55 | (solid.pzm1.bN51*solid.sE11+solid.pzm1.bN52*solid.sE12+solid.pzm1.bN53*solid.sE13+solid.pzm1.bN54*solid.sE14+solid.pzm1.bN55*solid.sE15+solid.pzm1.bN56*solid.sE16)*solid.pzm1.bN51+(solid.pzm1.bN51*solid.sE12+solid.pzm1.bN52*solid.sE22+solid.pzm1.bN53*solid.sE23+solid.pzm1.bN54*solid.sE24+solid.pzm1.bN55*solid.sE25+solid.pzm1.bN56*solid.sE26)*solid.pzm1.bN52+(solid.pzm1.bN51*solid.sE13+solid.pzm1.bN52*solid.sE23+solid.pzm1.bN53*solid.sE33+solid.pzm1.bN54*solid.sE34+solid.pzm1.bN55*solid.sE35+solid.pzm1.bN56*solid.sE36)*solid.pzm1.bN53+(solid.pzm1.bN51*solid.sE14+solid.pzm1.bN52*solid.sE24+solid.pzm1.bN53*solid.sE34+solid.pzm1.bN54*solid.sE44+solid.pzm1.bN55*solid.sE45+solid.pzm1.bN56*solid.sE46)*solid.pzm1.bN54+(solid.pzm1.bN51*solid.sE15+solid.pzm1.bN52*solid.sE25+solid.pzm1.bN53*solid.sE35+solid.pzm1.bN54*solid.sE45+solid.pzm1.bN55*solid.sE55+solid.pzm1.bN56*solid.sE56)*solid.pzm1.bN55+(solid.pzm1.bN51*solid.sE16+solid.pzm1.bN52*solid.sE26+solid.pzm1.bN53*solid.sE36+solid.pzm1.bN54*solid.sE46+solid.pzm1.bN55*solid.sE56+solid.pzm1.bN56*solid.sE66)*solid.pzm1.bN56 | 1/Pa | Compliance matrix sE (global), Voigt notation, 55-component | Domain 5 |  |
| solid.sEg56 | (solid.pzm1.bN51*solid.sE11+solid.pzm1.bN52*solid.sE12+solid.pzm1.bN53*solid.sE13+solid.pzm1.bN54*solid.sE14+solid.pzm1.bN55*solid.sE15+solid.pzm1.bN56*solid.sE16)*solid.pzm1.bN61+(solid.pzm1.bN51*solid.sE12+solid.pzm1.bN52*solid.sE22+solid.pzm1.bN53*solid.sE23+solid.pzm1.bN54*solid.sE24+solid.pzm1.bN55*solid.sE25+solid.pzm1.bN56*solid.sE26)*solid.pzm1.bN62+(solid.pzm1.bN51*solid.sE13+solid.pzm1.bN52*solid.sE23+solid.pzm1.bN53*solid.sE33+solid.pzm1.bN54*solid.sE34+solid.pzm1.bN55*solid.sE35+solid.pzm1.bN56*solid.sE36)*solid.pzm1.bN63+(solid.pzm1.bN51*solid.sE14+solid.pzm1.bN52*solid.sE24+solid.pzm1.bN53*solid.sE34+solid.pzm1.bN54*solid.sE44+solid.pzm1.bN55*solid.sE45+solid.pzm1.bN56*solid.sE46)*solid.pzm1.bN64+(solid.pzm1.bN51*solid.sE15+solid.pzm1.bN52*solid.sE25+solid.pzm1.bN53*solid.sE35+solid.pzm1.bN54*solid.sE45+solid.pzm1.bN55*solid.sE55+solid.pzm1.bN56*solid.sE56)*solid.pzm1.bN65+(solid.pzm1.bN51*solid.sE16+solid.pzm1.bN52*solid.sE26+solid.pzm1.bN53*solid.sE36+solid.pzm1.bN54*solid.sE46+solid.pzm1.bN55*solid.sE56+solid.pzm1.bN56*solid.sE66)*solid.pzm1.bN66 | 1/Pa | Compliance matrix sE (global), Voigt notation, 56-component | Domain 5 |  |
| solid.sEg66 | (solid.pzm1.bN61*solid.sE11+solid.pzm1.bN62*solid.sE12+solid.pzm1.bN63*solid.sE13+solid.pzm1.bN64*solid.sE14+solid.pzm1.bN65*solid.sE15+solid.pzm1.bN66*solid.sE16)*solid.pzm1.bN61+(solid.pzm1.bN61*solid.sE12+solid.pzm1.bN62*solid.sE22+solid.pzm1.bN63*solid.sE23+solid.pzm1.bN64*solid.sE24+solid.pzm1.bN65*solid.sE25+solid.pzm1.bN66*solid.sE26)*solid.pzm1.bN62+(solid.pzm1.bN61*solid.sE13+solid.pzm1.bN62*solid.sE23+solid.pzm1.bN63*solid.sE33+solid.pzm1.bN64*solid.sE34+solid.pzm1.bN65*solid.sE35+solid.pzm1.bN66*solid.sE36)*solid.pzm1.bN63+(solid.pzm1.bN61*solid.sE14+solid.pzm1.bN62*solid.sE24+solid.pzm1.bN63*solid.sE34+solid.pzm1.bN64*solid.sE44+solid.pzm1.bN65*solid.sE45+solid.pzm1.bN66*solid.sE46)*solid.pzm1.bN64+(solid.pzm1.bN61*solid.sE15+solid.pzm1.bN62*solid.sE25+solid.pzm1.bN63*solid.sE35+solid.pzm1.bN64*solid.sE45+solid.pzm1.bN65*solid.sE55+solid.pzm1.bN66*solid.sE56)*solid.pzm1.bN65+(solid.pzm1.bN61*solid.sE16+solid.pzm1.bN62*solid.sE26+solid.pzm1.bN63*solid.sE36+solid.pzm1.bN64*solid.sE46+solid.pzm1.bN65*solid.sE56+solid.pzm1.bN66*solid.sE66)*solid.pzm1.bN66 | 1/Pa | Compliance matrix sE (global), Voigt notation, 66-component | Domain 5 |  |
| solid.eESgX1 | (comp1_xz_sys.T11*solid.eES11+comp1_xz_sys.T21*solid.eES21+comp1_xz_sys.T31*solid.eES31)*solid.pzm1.bM11+(comp1_xz_sys.T11*solid.eES12+comp1_xz_sys.T21*solid.eES22+comp1_xz_sys.T31*solid.eES32)*solid.pzm1.bM12+(comp1_xz_sys.T11*solid.eES13+comp1_xz_sys.T21*solid.eES23+comp1_xz_sys.T31*solid.eES33)*solid.pzm1.bM13+(comp1_xz_sys.T11*solid.eES14+comp1_xz_sys.T21*solid.eES24+comp1_xz_sys.T31*solid.eES34)*solid.pzm1.bM14+(comp1_xz_sys.T11*solid.eES15+comp1_xz_sys.T21*solid.eES25+comp1_xz_sys.T31*solid.eES35)*solid.pzm1.bM15+(comp1_xz_sys.T11*solid.eES16+comp1_xz_sys.T21*solid.eES26+comp1_xz_sys.T31*solid.eES36)*solid.pzm1.bM16 | C/m² | Coupling matrix eES (global), Voigt notation, X1 component | Domain 5 |  |
| solid.eESgY1 | (comp1_xz_sys.T12*solid.eES11+comp1_xz_sys.T22*solid.eES21+comp1_xz_sys.T32*solid.eES31)*solid.pzm1.bM11+(comp1_xz_sys.T12*solid.eES12+comp1_xz_sys.T22*solid.eES22+comp1_xz_sys.T32*solid.eES32)*solid.pzm1.bM12+(comp1_xz_sys.T12*solid.eES13+comp1_xz_sys.T22*solid.eES23+comp1_xz_sys.T32*solid.eES33)*solid.pzm1.bM13+(comp1_xz_sys.T12*solid.eES14+comp1_xz_sys.T22*solid.eES24+comp1_xz_sys.T32*solid.eES34)*solid.pzm1.bM14+(comp1_xz_sys.T12*solid.eES15+comp1_xz_sys.T22*solid.eES25+comp1_xz_sys.T32*solid.eES35)*solid.pzm1.bM15+(comp1_xz_sys.T12*solid.eES16+comp1_xz_sys.T22*solid.eES26+comp1_xz_sys.T32*solid.eES36)*solid.pzm1.bM16 | C/m² | Coupling matrix eES (global), Voigt notation, Y1 component | Domain 5 |  |
| solid.eESgZ1 | (comp1_xz_sys.T13*solid.eES11+comp1_xz_sys.T23*solid.eES21+comp1_xz_sys.T33*solid.eES31)*solid.pzm1.bM11+(comp1_xz_sys.T13*solid.eES12+comp1_xz_sys.T23*solid.eES22+comp1_xz_sys.T33*solid.eES32)*solid.pzm1.bM12+(comp1_xz_sys.T13*solid.eES13+comp1_xz_sys.T23*solid.eES23+comp1_xz_sys.T33*solid.eES33)*solid.pzm1.bM13+(comp1_xz_sys.T13*solid.eES14+comp1_xz_sys.T23*solid.eES24+comp1_xz_sys.T33*solid.eES34)*solid.pzm1.bM14+(comp1_xz_sys.T13*solid.eES15+comp1_xz_sys.T23*solid.eES25+comp1_xz_sys.T33*solid.eES35)*solid.pzm1.bM15+(comp1_xz_sys.T13*solid.eES16+comp1_xz_sys.T23*solid.eES26+comp1_xz_sys.T33*solid.eES36)*solid.pzm1.bM16 | C/m² | Coupling matrix eES (global), Voigt notation, Z1 component | Domain 5 |  |
| solid.eESgX2 | (comp1_xz_sys.T11*solid.eES11+comp1_xz_sys.T21*solid.eES21+comp1_xz_sys.T31*solid.eES31)*solid.pzm1.bM21+(comp1_xz_sys.T11*solid.eES12+comp1_xz_sys.T21*solid.eES22+comp1_xz_sys.T31*solid.eES32)*solid.pzm1.bM22+(comp1_xz_sys.T11*solid.eES13+comp1_xz_sys.T21*solid.eES23+comp1_xz_sys.T31*solid.eES33)*solid.pzm1.bM23+(comp1_xz_sys.T11*solid.eES14+comp1_xz_sys.T21*solid.eES24+comp1_xz_sys.T31*solid.eES34)*solid.pzm1.bM24+(comp1_xz_sys.T11*solid.eES15+comp1_xz_sys.T21*solid.eES25+comp1_xz_sys.T31*solid.eES35)*solid.pzm1.bM25+(comp1_xz_sys.T11*solid.eES16+comp1_xz_sys.T21*solid.eES26+comp1_xz_sys.T31*solid.eES36)*solid.pzm1.bM26 | C/m² | Coupling matrix eES (global), Voigt notation, X2 component | Domain 5 |  |
| solid.eESgY2 | (comp1_xz_sys.T12*solid.eES11+comp1_xz_sys.T22*solid.eES21+comp1_xz_sys.T32*solid.eES31)*solid.pzm1.bM21+(comp1_xz_sys.T12*solid.eES12+comp1_xz_sys.T22*solid.eES22+comp1_xz_sys.T32*solid.eES32)*solid.pzm1.bM22+(comp1_xz_sys.T12*solid.eES13+comp1_xz_sys.T22*solid.eES23+comp1_xz_sys.T32*solid.eES33)*solid.pzm1.bM23+(comp1_xz_sys.T12*solid.eES14+comp1_xz_sys.T22*solid.eES24+comp1_xz_sys.T32*solid.eES34)*solid.pzm1.bM24+(comp1_xz_sys.T12*solid.eES15+comp1_xz_sys.T22*solid.eES25+comp1_xz_sys.T32*solid.eES35)*solid.pzm1.bM25+(comp1_xz_sys.T12*solid.eES16+comp1_xz_sys.T22*solid.eES26+comp1_xz_sys.T32*solid.eES36)*solid.pzm1.bM26 | C/m² | Coupling matrix eES (global), Voigt notation, Y2 component | Domain 5 |  |
| solid.eESgZ2 | (comp1_xz_sys.T13*solid.eES11+comp1_xz_sys.T23*solid.eES21+comp1_xz_sys.T33*solid.eES31)*solid.pzm1.bM21+(comp1_xz_sys.T13*solid.eES12+comp1_xz_sys.T23*solid.eES22+comp1_xz_sys.T33*solid.eES32)*solid.pzm1.bM22+(comp1_xz_sys.T13*solid.eES13+comp1_xz_sys.T23*solid.eES23+comp1_xz_sys.T33*solid.eES33)*solid.pzm1.bM23+(comp1_xz_sys.T13*solid.eES14+comp1_xz_sys.T23*solid.eES24+comp1_xz_sys.T33*solid.eES34)*solid.pzm1.bM24+(comp1_xz_sys.T13*solid.eES15+comp1_xz_sys.T23*solid.eES25+comp1_xz_sys.T33*solid.eES35)*solid.pzm1.bM25+(comp1_xz_sys.T13*solid.eES16+comp1_xz_sys.T23*solid.eES26+comp1_xz_sys.T33*solid.eES36)*solid.pzm1.bM26 | C/m² | Coupling matrix eES (global), Voigt notation, Z2 component | Domain 5 |  |
| solid.eESgX3 | (comp1_xz_sys.T11*solid.eES11+comp1_xz_sys.T21*solid.eES21+comp1_xz_sys.T31*solid.eES31)*solid.pzm1.bM31+(comp1_xz_sys.T11*solid.eES12+comp1_xz_sys.T21*solid.eES22+comp1_xz_sys.T31*solid.eES32)*solid.pzm1.bM32+(comp1_xz_sys.T11*solid.eES13+comp1_xz_sys.T21*solid.eES23+comp1_xz_sys.T31*solid.eES33)*solid.pzm1.bM33+(comp1_xz_sys.T11*solid.eES14+comp1_xz_sys.T21*solid.eES24+comp1_xz_sys.T31*solid.eES34)*solid.pzm1.bM34+(comp1_xz_sys.T11*solid.eES15+comp1_xz_sys.T21*solid.eES25+comp1_xz_sys.T31*solid.eES35)*solid.pzm1.bM35+(comp1_xz_sys.T11*solid.eES16+comp1_xz_sys.T21*solid.eES26+comp1_xz_sys.T31*solid.eES36)*solid.pzm1.bM36 | C/m² | Coupling matrix eES (global), Voigt notation, X3 component | Domain 5 |  |
| solid.eESgY3 | (comp1_xz_sys.T12*solid.eES11+comp1_xz_sys.T22*solid.eES21+comp1_xz_sys.T32*solid.eES31)*solid.pzm1.bM31+(comp1_xz_sys.T12*solid.eES12+comp1_xz_sys.T22*solid.eES22+comp1_xz_sys.T32*solid.eES32)*solid.pzm1.bM32+(comp1_xz_sys.T12*solid.eES13+comp1_xz_sys.T22*solid.eES23+comp1_xz_sys.T32*solid.eES33)*solid.pzm1.bM33+(comp1_xz_sys.T12*solid.eES14+comp1_xz_sys.T22*solid.eES24+comp1_xz_sys.T32*solid.eES34)*solid.pzm1.bM34+(comp1_xz_sys.T12*solid.eES15+comp1_xz_sys.T22*solid.eES25+comp1_xz_sys.T32*solid.eES35)*solid.pzm1.bM35+(comp1_xz_sys.T12*solid.eES16+comp1_xz_sys.T22*solid.eES26+comp1_xz_sys.T32*solid.eES36)*solid.pzm1.bM36 | C/m² | Coupling matrix eES (global), Voigt notation, Y3 component | Domain 5 |  |
| solid.eESgZ3 | (comp1_xz_sys.T13*solid.eES11+comp1_xz_sys.T23*solid.eES21+comp1_xz_sys.T33*solid.eES31)*solid.pzm1.bM31+(comp1_xz_sys.T13*solid.eES12+comp1_xz_sys.T23*solid.eES22+comp1_xz_sys.T33*solid.eES32)*solid.pzm1.bM32+(comp1_xz_sys.T13*solid.eES13+comp1_xz_sys.T23*solid.eES23+comp1_xz_sys.T33*solid.eES33)*solid.pzm1.bM33+(comp1_xz_sys.T13*solid.eES14+comp1_xz_sys.T23*solid.eES24+comp1_xz_sys.T33*solid.eES34)*solid.pzm1.bM34+(comp1_xz_sys.T13*solid.eES15+comp1_xz_sys.T23*solid.eES25+comp1_xz_sys.T33*solid.eES35)*solid.pzm1.bM35+(comp1_xz_sys.T13*solid.eES16+comp1_xz_sys.T23*solid.eES26+comp1_xz_sys.T33*solid.eES36)*solid.pzm1.bM36 | C/m² | Coupling matrix eES (global), Voigt notation, Z3 component | Domain 5 |  |
| solid.eESgX4 | (comp1_xz_sys.T11*solid.eES11+comp1_xz_sys.T21*solid.eES21+comp1_xz_sys.T31*solid.eES31)*solid.pzm1.bM41+(comp1_xz_sys.T11*solid.eES12+comp1_xz_sys.T21*solid.eES22+comp1_xz_sys.T31*solid.eES32)*solid.pzm1.bM42+(comp1_xz_sys.T11*solid.eES13+comp1_xz_sys.T21*solid.eES23+comp1_xz_sys.T31*solid.eES33)*solid.pzm1.bM43+(comp1_xz_sys.T11*solid.eES14+comp1_xz_sys.T21*solid.eES24+comp1_xz_sys.T31*solid.eES34)*solid.pzm1.bM44+(comp1_xz_sys.T11*solid.eES15+comp1_xz_sys.T21*solid.eES25+comp1_xz_sys.T31*solid.eES35)*solid.pzm1.bM45+(comp1_xz_sys.T11*solid.eES16+comp1_xz_sys.T21*solid.eES26+comp1_xz_sys.T31*solid.eES36)*solid.pzm1.bM46 | C/m² | Coupling matrix eES (global), Voigt notation, X4 component | Domain 5 |  |
| solid.eESgY4 | (comp1_xz_sys.T12*solid.eES11+comp1_xz_sys.T22*solid.eES21+comp1_xz_sys.T32*solid.eES31)*solid.pzm1.bM41+(comp1_xz_sys.T12*solid.eES12+comp1_xz_sys.T22*solid.eES22+comp1_xz_sys.T32*solid.eES32)*solid.pzm1.bM42+(comp1_xz_sys.T12*solid.eES13+comp1_xz_sys.T22*solid.eES23+comp1_xz_sys.T32*solid.eES33)*solid.pzm1.bM43+(comp1_xz_sys.T12*solid.eES14+comp1_xz_sys.T22*solid.eES24+comp1_xz_sys.T32*solid.eES34)*solid.pzm1.bM44+(comp1_xz_sys.T12*solid.eES15+comp1_xz_sys.T22*solid.eES25+comp1_xz_sys.T32*solid.eES35)*solid.pzm1.bM45+(comp1_xz_sys.T12*solid.eES16+comp1_xz_sys.T22*solid.eES26+comp1_xz_sys.T32*solid.eES36)*solid.pzm1.bM46 | C/m² | Coupling matrix eES (global), Voigt notation, Y4 component | Domain 5 |  |
| solid.eESgZ4 | (comp1_xz_sys.T13*solid.eES11+comp1_xz_sys.T23*solid.eES21+comp1_xz_sys.T33*solid.eES31)*solid.pzm1.bM41+(comp1_xz_sys.T13*solid.eES12+comp1_xz_sys.T23*solid.eES22+comp1_xz_sys.T33*solid.eES32)*solid.pzm1.bM42+(comp1_xz_sys.T13*solid.eES13+comp1_xz_sys.T23*solid.eES23+comp1_xz_sys.T33*solid.eES33)*solid.pzm1.bM43+(comp1_xz_sys.T13*solid.eES14+comp1_xz_sys.T23*solid.eES24+comp1_xz_sys.T33*solid.eES34)*solid.pzm1.bM44+(comp1_xz_sys.T13*solid.eES15+comp1_xz_sys.T23*solid.eES25+comp1_xz_sys.T33*solid.eES35)*solid.pzm1.bM45+(comp1_xz_sys.T13*solid.eES16+comp1_xz_sys.T23*solid.eES26+comp1_xz_sys.T33*solid.eES36)*solid.pzm1.bM46 | C/m² | Coupling matrix eES (global), Voigt notation, Z4 component | Domain 5 |  |
| solid.eESgX5 | (comp1_xz_sys.T11*solid.eES11+comp1_xz_sys.T21*solid.eES21+comp1_xz_sys.T31*solid.eES31)*solid.pzm1.bM51+(comp1_xz_sys.T11*solid.eES12+comp1_xz_sys.T21*solid.eES22+comp1_xz_sys.T31*solid.eES32)*solid.pzm1.bM52+(comp1_xz_sys.T11*solid.eES13+comp1_xz_sys.T21*solid.eES23+comp1_xz_sys.T31*solid.eES33)*solid.pzm1.bM53+(comp1_xz_sys.T11*solid.eES14+comp1_xz_sys.T21*solid.eES24+comp1_xz_sys.T31*solid.eES34)*solid.pzm1.bM54+(comp1_xz_sys.T11*solid.eES15+comp1_xz_sys.T21*solid.eES25+comp1_xz_sys.T31*solid.eES35)*solid.pzm1.bM55+(comp1_xz_sys.T11*solid.eES16+comp1_xz_sys.T21*solid.eES26+comp1_xz_sys.T31*solid.eES36)*solid.pzm1.bM56 | C/m² | Coupling matrix eES (global), Voigt notation, X5 component | Domain 5 |  |
| solid.eESgY5 | (comp1_xz_sys.T12*solid.eES11+comp1_xz_sys.T22*solid.eES21+comp1_xz_sys.T32*solid.eES31)*solid.pzm1.bM51+(comp1_xz_sys.T12*solid.eES12+comp1_xz_sys.T22*solid.eES22+comp1_xz_sys.T32*solid.eES32)*solid.pzm1.bM52+(comp1_xz_sys.T12*solid.eES13+comp1_xz_sys.T22*solid.eES23+comp1_xz_sys.T32*solid.eES33)*solid.pzm1.bM53+(comp1_xz_sys.T12*solid.eES14+comp1_xz_sys.T22*solid.eES24+comp1_xz_sys.T32*solid.eES34)*solid.pzm1.bM54+(comp1_xz_sys.T12*solid.eES15+comp1_xz_sys.T22*solid.eES25+comp1_xz_sys.T32*solid.eES35)*solid.pzm1.bM55+(comp1_xz_sys.T12*solid.eES16+comp1_xz_sys.T22*solid.eES26+comp1_xz_sys.T32*solid.eES36)*solid.pzm1.bM56 | C/m² | Coupling matrix eES (global), Voigt notation, Y5 component | Domain 5 |  |
| solid.eESgZ5 | (comp1_xz_sys.T13*solid.eES11+comp1_xz_sys.T23*solid.eES21+comp1_xz_sys.T33*solid.eES31)*solid.pzm1.bM51+(comp1_xz_sys.T13*solid.eES12+comp1_xz_sys.T23*solid.eES22+comp1_xz_sys.T33*solid.eES32)*solid.pzm1.bM52+(comp1_xz_sys.T13*solid.eES13+comp1_xz_sys.T23*solid.eES23+comp1_xz_sys.T33*solid.eES33)*solid.pzm1.bM53+(comp1_xz_sys.T13*solid.eES14+comp1_xz_sys.T23*solid.eES24+comp1_xz_sys.T33*solid.eES34)*solid.pzm1.bM54+(comp1_xz_sys.T13*solid.eES15+comp1_xz_sys.T23*solid.eES25+comp1_xz_sys.T33*solid.eES35)*solid.pzm1.bM55+(comp1_xz_sys.T13*solid.eES16+comp1_xz_sys.T23*solid.eES26+comp1_xz_sys.T33*solid.eES36)*solid.pzm1.bM56 | C/m² | Coupling matrix eES (global), Voigt notation, Z5 component | Domain 5 |  |
| solid.eESgX6 | (comp1_xz_sys.T11*solid.eES11+comp1_xz_sys.T21*solid.eES21+comp1_xz_sys.T31*solid.eES31)*solid.pzm1.bM61+(comp1_xz_sys.T11*solid.eES12+comp1_xz_sys.T21*solid.eES22+comp1_xz_sys.T31*solid.eES32)*solid.pzm1.bM62+(comp1_xz_sys.T11*solid.eES13+comp1_xz_sys.T21*solid.eES23+comp1_xz_sys.T31*solid.eES33)*solid.pzm1.bM63+(comp1_xz_sys.T11*solid.eES14+comp1_xz_sys.T21*solid.eES24+comp1_xz_sys.T31*solid.eES34)*solid.pzm1.bM64+(comp1_xz_sys.T11*solid.eES15+comp1_xz_sys.T21*solid.eES25+comp1_xz_sys.T31*solid.eES35)*solid.pzm1.bM65+(comp1_xz_sys.T11*solid.eES16+comp1_xz_sys.T21*solid.eES26+comp1_xz_sys.T31*solid.eES36)*solid.pzm1.bM66 | C/m² | Coupling matrix eES (global), Voigt notation, X6 component | Domain 5 |  |
| solid.eESgY6 | (comp1_xz_sys.T12*solid.eES11+comp1_xz_sys.T22*solid.eES21+comp1_xz_sys.T32*solid.eES31)*solid.pzm1.bM61+(comp1_xz_sys.T12*solid.eES12+comp1_xz_sys.T22*solid.eES22+comp1_xz_sys.T32*solid.eES32)*solid.pzm1.bM62+(comp1_xz_sys.T12*solid.eES13+comp1_xz_sys.T22*solid.eES23+comp1_xz_sys.T32*solid.eES33)*solid.pzm1.bM63+(comp1_xz_sys.T12*solid.eES14+comp1_xz_sys.T22*solid.eES24+comp1_xz_sys.T32*solid.eES34)*solid.pzm1.bM64+(comp1_xz_sys.T12*solid.eES15+comp1_xz_sys.T22*solid.eES25+comp1_xz_sys.T32*solid.eES35)*solid.pzm1.bM65+(comp1_xz_sys.T12*solid.eES16+comp1_xz_sys.T22*solid.eES26+comp1_xz_sys.T32*solid.eES36)*solid.pzm1.bM66 | C/m² | Coupling matrix eES (global), Voigt notation, Y6 component | Domain 5 |  |
| solid.eESgZ6 | (comp1_xz_sys.T13*solid.eES11+comp1_xz_sys.T23*solid.eES21+comp1_xz_sys.T33*solid.eES31)*solid.pzm1.bM61+(comp1_xz_sys.T13*solid.eES12+comp1_xz_sys.T23*solid.eES22+comp1_xz_sys.T33*solid.eES32)*solid.pzm1.bM62+(comp1_xz_sys.T13*solid.eES13+comp1_xz_sys.T23*solid.eES23+comp1_xz_sys.T33*solid.eES33)*solid.pzm1.bM63+(comp1_xz_sys.T13*solid.eES14+comp1_xz_sys.T23*solid.eES24+comp1_xz_sys.T33*solid.eES34)*solid.pzm1.bM64+(comp1_xz_sys.T13*solid.eES15+comp1_xz_sys.T23*solid.eES25+comp1_xz_sys.T33*solid.eES35)*solid.pzm1.bM65+(comp1_xz_sys.T13*solid.eES16+comp1_xz_sys.T23*solid.eES26+comp1_xz_sys.T33*solid.eES36)*solid.pzm1.bM66 | C/m² | Coupling matrix eES (global), Voigt notation, Z6 component | Domain 5 |  |
| solid.dETgX1 | (comp1_xz_sys.T11*solid.dET11+comp1_xz_sys.T21*solid.dET21+comp1_xz_sys.T31*solid.dET31)*solid.pzm1.bN11+(comp1_xz_sys.T11*solid.dET12+comp1_xz_sys.T21*solid.dET22+comp1_xz_sys.T31*solid.dET32)*solid.pzm1.bN12+(comp1_xz_sys.T11*solid.dET13+comp1_xz_sys.T21*solid.dET23+comp1_xz_sys.T31*solid.dET33)*solid.pzm1.bN13+(comp1_xz_sys.T11*solid.dET14+comp1_xz_sys.T21*solid.dET24+comp1_xz_sys.T31*solid.dET34)*solid.pzm1.bN14+(comp1_xz_sys.T11*solid.dET15+comp1_xz_sys.T21*solid.dET25+comp1_xz_sys.T31*solid.dET35)*solid.pzm1.bN15+(comp1_xz_sys.T11*solid.dET16+comp1_xz_sys.T21*solid.dET26+comp1_xz_sys.T31*solid.dET36)*solid.pzm1.bN16 | C/N | Coupling matrix dET (global), Voigt notation, X1 component | Domain 5 |  |
| solid.dETgY1 | (comp1_xz_sys.T12*solid.dET11+comp1_xz_sys.T22*solid.dET21+comp1_xz_sys.T32*solid.dET31)*solid.pzm1.bN11+(comp1_xz_sys.T12*solid.dET12+comp1_xz_sys.T22*solid.dET22+comp1_xz_sys.T32*solid.dET32)*solid.pzm1.bN12+(comp1_xz_sys.T12*solid.dET13+comp1_xz_sys.T22*solid.dET23+comp1_xz_sys.T32*solid.dET33)*solid.pzm1.bN13+(comp1_xz_sys.T12*solid.dET14+comp1_xz_sys.T22*solid.dET24+comp1_xz_sys.T32*solid.dET34)*solid.pzm1.bN14+(comp1_xz_sys.T12*solid.dET15+comp1_xz_sys.T22*solid.dET25+comp1_xz_sys.T32*solid.dET35)*solid.pzm1.bN15+(comp1_xz_sys.T12*solid.dET16+comp1_xz_sys.T22*solid.dET26+comp1_xz_sys.T32*solid.dET36)*solid.pzm1.bN16 | C/N | Coupling matrix dET (global), Voigt notation, Y1 component | Domain 5 |  |
| solid.dETgZ1 | (comp1_xz_sys.T13*solid.dET11+comp1_xz_sys.T23*solid.dET21+comp1_xz_sys.T33*solid.dET31)*solid.pzm1.bN11+(comp1_xz_sys.T13*solid.dET12+comp1_xz_sys.T23*solid.dET22+comp1_xz_sys.T33*solid.dET32)*solid.pzm1.bN12+(comp1_xz_sys.T13*solid.dET13+comp1_xz_sys.T23*solid.dET23+comp1_xz_sys.T33*solid.dET33)*solid.pzm1.bN13+(comp1_xz_sys.T13*solid.dET14+comp1_xz_sys.T23*solid.dET24+comp1_xz_sys.T33*solid.dET34)*solid.pzm1.bN14+(comp1_xz_sys.T13*solid.dET15+comp1_xz_sys.T23*solid.dET25+comp1_xz_sys.T33*solid.dET35)*solid.pzm1.bN15+(comp1_xz_sys.T13*solid.dET16+comp1_xz_sys.T23*solid.dET26+comp1_xz_sys.T33*solid.dET36)*solid.pzm1.bN16 | C/N | Coupling matrix dET (global), Voigt notation, Z1 component | Domain 5 |  |
| solid.dETgX2 | (comp1_xz_sys.T11*solid.dET11+comp1_xz_sys.T21*solid.dET21+comp1_xz_sys.T31*solid.dET31)*solid.pzm1.bN21+(comp1_xz_sys.T11*solid.dET12+comp1_xz_sys.T21*solid.dET22+comp1_xz_sys.T31*solid.dET32)*solid.pzm1.bN22+(comp1_xz_sys.T11*solid.dET13+comp1_xz_sys.T21*solid.dET23+comp1_xz_sys.T31*solid.dET33)*solid.pzm1.bN23+(comp1_xz_sys.T11*solid.dET14+comp1_xz_sys.T21*solid.dET24+comp1_xz_sys.T31*solid.dET34)*solid.pzm1.bN24+(comp1_xz_sys.T11*solid.dET15+comp1_xz_sys.T21*solid.dET25+comp1_xz_sys.T31*solid.dET35)*solid.pzm1.bN25+(comp1_xz_sys.T11*solid.dET16+comp1_xz_sys.T21*solid.dET26+comp1_xz_sys.T31*solid.dET36)*solid.pzm1.bN26 | C/N | Coupling matrix dET (global), Voigt notation, X2 component | Domain 5 |  |
| solid.dETgY2 | (comp1_xz_sys.T12*solid.dET11+comp1_xz_sys.T22*solid.dET21+comp1_xz_sys.T32*solid.dET31)*solid.pzm1.bN21+(comp1_xz_sys.T12*solid.dET12+comp1_xz_sys.T22*solid.dET22+comp1_xz_sys.T32*solid.dET32)*solid.pzm1.bN22+(comp1_xz_sys.T12*solid.dET13+comp1_xz_sys.T22*solid.dET23+comp1_xz_sys.T32*solid.dET33)*solid.pzm1.bN23+(comp1_xz_sys.T12*solid.dET14+comp1_xz_sys.T22*solid.dET24+comp1_xz_sys.T32*solid.dET34)*solid.pzm1.bN24+(comp1_xz_sys.T12*solid.dET15+comp1_xz_sys.T22*solid.dET25+comp1_xz_sys.T32*solid.dET35)*solid.pzm1.bN25+(comp1_xz_sys.T12*solid.dET16+comp1_xz_sys.T22*solid.dET26+comp1_xz_sys.T32*solid.dET36)*solid.pzm1.bN26 | C/N | Coupling matrix dET (global), Voigt notation, Y2 component | Domain 5 |  |
| solid.dETgZ2 | (comp1_xz_sys.T13*solid.dET11+comp1_xz_sys.T23*solid.dET21+comp1_xz_sys.T33*solid.dET31)*solid.pzm1.bN21+(comp1_xz_sys.T13*solid.dET12+comp1_xz_sys.T23*solid.dET22+comp1_xz_sys.T33*solid.dET32)*solid.pzm1.bN22+(comp1_xz_sys.T13*solid.dET13+comp1_xz_sys.T23*solid.dET23+comp1_xz_sys.T33*solid.dET33)*solid.pzm1.bN23+(comp1_xz_sys.T13*solid.dET14+comp1_xz_sys.T23*solid.dET24+comp1_xz_sys.T33*solid.dET34)*solid.pzm1.bN24+(comp1_xz_sys.T13*solid.dET15+comp1_xz_sys.T23*solid.dET25+comp1_xz_sys.T33*solid.dET35)*solid.pzm1.bN25+(comp1_xz_sys.T13*solid.dET16+comp1_xz_sys.T23*solid.dET26+comp1_xz_sys.T33*solid.dET36)*solid.pzm1.bN26 | C/N | Coupling matrix dET (global), Voigt notation, Z2 component | Domain 5 |  |
| solid.dETgX3 | (comp1_xz_sys.T11*solid.dET11+comp1_xz_sys.T21*solid.dET21+comp1_xz_sys.T31*solid.dET31)*solid.pzm1.bN31+(comp1_xz_sys.T11*solid.dET12+comp1_xz_sys.T21*solid.dET22+comp1_xz_sys.T31*solid.dET32)*solid.pzm1.bN32+(comp1_xz_sys.T11*solid.dET13+comp1_xz_sys.T21*solid.dET23+comp1_xz_sys.T31*solid.dET33)*solid.pzm1.bN33+(comp1_xz_sys.T11*solid.dET14+comp1_xz_sys.T21*solid.dET24+comp1_xz_sys.T31*solid.dET34)*solid.pzm1.bN34+(comp1_xz_sys.T11*solid.dET15+comp1_xz_sys.T21*solid.dET25+comp1_xz_sys.T31*solid.dET35)*solid.pzm1.bN35+(comp1_xz_sys.T11*solid.dET16+comp1_xz_sys.T21*solid.dET26+comp1_xz_sys.T31*solid.dET36)*solid.pzm1.bN36 | C/N | Coupling matrix dET (global), Voigt notation, X3 component | Domain 5 |  |
| solid.dETgY3 | (comp1_xz_sys.T12*solid.dET11+comp1_xz_sys.T22*solid.dET21+comp1_xz_sys.T32*solid.dET31)*solid.pzm1.bN31+(comp1_xz_sys.T12*solid.dET12+comp1_xz_sys.T22*solid.dET22+comp1_xz_sys.T32*solid.dET32)*solid.pzm1.bN32+(comp1_xz_sys.T12*solid.dET13+comp1_xz_sys.T22*solid.dET23+comp1_xz_sys.T32*solid.dET33)*solid.pzm1.bN33+(comp1_xz_sys.T12*solid.dET14+comp1_xz_sys.T22*solid.dET24+comp1_xz_sys.T32*solid.dET34)*solid.pzm1.bN34+(comp1_xz_sys.T12*solid.dET15+comp1_xz_sys.T22*solid.dET25+comp1_xz_sys.T32*solid.dET35)*solid.pzm1.bN35+(comp1_xz_sys.T12*solid.dET16+comp1_xz_sys.T22*solid.dET26+comp1_xz_sys.T32*solid.dET36)*solid.pzm1.bN36 | C/N | Coupling matrix dET (global), Voigt notation, Y3 component | Domain 5 |  |
| solid.dETgZ3 | (comp1_xz_sys.T13*solid.dET11+comp1_xz_sys.T23*solid.dET21+comp1_xz_sys.T33*solid.dET31)*solid.pzm1.bN31+(comp1_xz_sys.T13*solid.dET12+comp1_xz_sys.T23*solid.dET22+comp1_xz_sys.T33*solid.dET32)*solid.pzm1.bN32+(comp1_xz_sys.T13*solid.dET13+comp1_xz_sys.T23*solid.dET23+comp1_xz_sys.T33*solid.dET33)*solid.pzm1.bN33+(comp1_xz_sys.T13*solid.dET14+comp1_xz_sys.T23*solid.dET24+comp1_xz_sys.T33*solid.dET34)*solid.pzm1.bN34+(comp1_xz_sys.T13*solid.dET15+comp1_xz_sys.T23*solid.dET25+comp1_xz_sys.T33*solid.dET35)*solid.pzm1.bN35+(comp1_xz_sys.T13*solid.dET16+comp1_xz_sys.T23*solid.dET26+comp1_xz_sys.T33*solid.dET36)*solid.pzm1.bN36 | C/N | Coupling matrix dET (global), Voigt notation, Z3 component | Domain 5 |  |
| solid.dETgX4 | (comp1_xz_sys.T11*solid.dET11+comp1_xz_sys.T21*solid.dET21+comp1_xz_sys.T31*solid.dET31)*solid.pzm1.bN41+(comp1_xz_sys.T11*solid.dET12+comp1_xz_sys.T21*solid.dET22+comp1_xz_sys.T31*solid.dET32)*solid.pzm1.bN42+(comp1_xz_sys.T11*solid.dET13+comp1_xz_sys.T21*solid.dET23+comp1_xz_sys.T31*solid.dET33)*solid.pzm1.bN43+(comp1_xz_sys.T11*solid.dET14+comp1_xz_sys.T21*solid.dET24+comp1_xz_sys.T31*solid.dET34)*solid.pzm1.bN44+(comp1_xz_sys.T11*solid.dET15+comp1_xz_sys.T21*solid.dET25+comp1_xz_sys.T31*solid.dET35)*solid.pzm1.bN45+(comp1_xz_sys.T11*solid.dET16+comp1_xz_sys.T21*solid.dET26+comp1_xz_sys.T31*solid.dET36)*solid.pzm1.bN46 | C/N | Coupling matrix dET (global), Voigt notation, X4 component | Domain 5 |  |
| solid.dETgY4 | (comp1_xz_sys.T12*solid.dET11+comp1_xz_sys.T22*solid.dET21+comp1_xz_sys.T32*solid.dET31)*solid.pzm1.bN41+(comp1_xz_sys.T12*solid.dET12+comp1_xz_sys.T22*solid.dET22+comp1_xz_sys.T32*solid.dET32)*solid.pzm1.bN42+(comp1_xz_sys.T12*solid.dET13+comp1_xz_sys.T22*solid.dET23+comp1_xz_sys.T32*solid.dET33)*solid.pzm1.bN43+(comp1_xz_sys.T12*solid.dET14+comp1_xz_sys.T22*solid.dET24+comp1_xz_sys.T32*solid.dET34)*solid.pzm1.bN44+(comp1_xz_sys.T12*solid.dET15+comp1_xz_sys.T22*solid.dET25+comp1_xz_sys.T32*solid.dET35)*solid.pzm1.bN45+(comp1_xz_sys.T12*solid.dET16+comp1_xz_sys.T22*solid.dET26+comp1_xz_sys.T32*solid.dET36)*solid.pzm1.bN46 | C/N | Coupling matrix dET (global), Voigt notation, Y4 component | Domain 5 |  |
| solid.dETgZ4 | (comp1_xz_sys.T13*solid.dET11+comp1_xz_sys.T23*solid.dET21+comp1_xz_sys.T33*solid.dET31)*solid.pzm1.bN41+(comp1_xz_sys.T13*solid.dET12+comp1_xz_sys.T23*solid.dET22+comp1_xz_sys.T33*solid.dET32)*solid.pzm1.bN42+(comp1_xz_sys.T13*solid.dET13+comp1_xz_sys.T23*solid.dET23+comp1_xz_sys.T33*solid.dET33)*solid.pzm1.bN43+(comp1_xz_sys.T13*solid.dET14+comp1_xz_sys.T23*solid.dET24+comp1_xz_sys.T33*solid.dET34)*solid.pzm1.bN44+(comp1_xz_sys.T13*solid.dET15+comp1_xz_sys.T23*solid.dET25+comp1_xz_sys.T33*solid.dET35)*solid.pzm1.bN45+(comp1_xz_sys.T13*solid.dET16+comp1_xz_sys.T23*solid.dET26+comp1_xz_sys.T33*solid.dET36)*solid.pzm1.bN46 | C/N | Coupling matrix dET (global), Voigt notation, Z4 component | Domain 5 |  |
| solid.dETgX5 | (comp1_xz_sys.T11*solid.dET11+comp1_xz_sys.T21*solid.dET21+comp1_xz_sys.T31*solid.dET31)*solid.pzm1.bN51+(comp1_xz_sys.T11*solid.dET12+comp1_xz_sys.T21*solid.dET22+comp1_xz_sys.T31*solid.dET32)*solid.pzm1.bN52+(comp1_xz_sys.T11*solid.dET13+comp1_xz_sys.T21*solid.dET23+comp1_xz_sys.T31*solid.dET33)*solid.pzm1.bN53+(comp1_xz_sys.T11*solid.dET14+comp1_xz_sys.T21*solid.dET24+comp1_xz_sys.T31*solid.dET34)*solid.pzm1.bN54+(comp1_xz_sys.T11*solid.dET15+comp1_xz_sys.T21*solid.dET25+comp1_xz_sys.T31*solid.dET35)*solid.pzm1.bN55+(comp1_xz_sys.T11*solid.dET16+comp1_xz_sys.T21*solid.dET26+comp1_xz_sys.T31*solid.dET36)*solid.pzm1.bN56 | C/N | Coupling matrix dET (global), Voigt notation, X5 component | Domain 5 |  |
| solid.dETgY5 | (comp1_xz_sys.T12*solid.dET11+comp1_xz_sys.T22*solid.dET21+comp1_xz_sys.T32*solid.dET31)*solid.pzm1.bN51+(comp1_xz_sys.T12*solid.dET12+comp1_xz_sys.T22*solid.dET22+comp1_xz_sys.T32*solid.dET32)*solid.pzm1.bN52+(comp1_xz_sys.T12*solid.dET13+comp1_xz_sys.T22*solid.dET23+comp1_xz_sys.T32*solid.dET33)*solid.pzm1.bN53+(comp1_xz_sys.T12*solid.dET14+comp1_xz_sys.T22*solid.dET24+comp1_xz_sys.T32*solid.dET34)*solid.pzm1.bN54+(comp1_xz_sys.T12*solid.dET15+comp1_xz_sys.T22*solid.dET25+comp1_xz_sys.T32*solid.dET35)*solid.pzm1.bN55+(comp1_xz_sys.T12*solid.dET16+comp1_xz_sys.T22*solid.dET26+comp1_xz_sys.T32*solid.dET36)*solid.pzm1.bN56 | C/N | Coupling matrix dET (global), Voigt notation, Y5 component | Domain 5 |  |
| solid.dETgZ5 | (comp1_xz_sys.T13*solid.dET11+comp1_xz_sys.T23*solid.dET21+comp1_xz_sys.T33*solid.dET31)*solid.pzm1.bN51+(comp1_xz_sys.T13*solid.dET12+comp1_xz_sys.T23*solid.dET22+comp1_xz_sys.T33*solid.dET32)*solid.pzm1.bN52+(comp1_xz_sys.T13*solid.dET13+comp1_xz_sys.T23*solid.dET23+comp1_xz_sys.T33*solid.dET33)*solid.pzm1.bN53+(comp1_xz_sys.T13*solid.dET14+comp1_xz_sys.T23*solid.dET24+comp1_xz_sys.T33*solid.dET34)*solid.pzm1.bN54+(comp1_xz_sys.T13*solid.dET15+comp1_xz_sys.T23*solid.dET25+comp1_xz_sys.T33*solid.dET35)*solid.pzm1.bN55+(comp1_xz_sys.T13*solid.dET16+comp1_xz_sys.T23*solid.dET26+comp1_xz_sys.T33*solid.dET36)*solid.pzm1.bN56 | C/N | Coupling matrix dET (global), Voigt notation, Z5 component | Domain 5 |  |
| solid.dETgX6 | (comp1_xz_sys.T11*solid.dET11+comp1_xz_sys.T21*solid.dET21+comp1_xz_sys.T31*solid.dET31)*solid.pzm1.bN61+(comp1_xz_sys.T11*solid.dET12+comp1_xz_sys.T21*solid.dET22+comp1_xz_sys.T31*solid.dET32)*solid.pzm1.bN62+(comp1_xz_sys.T11*solid.dET13+comp1_xz_sys.T21*solid.dET23+comp1_xz_sys.T31*solid.dET33)*solid.pzm1.bN63+(comp1_xz_sys.T11*solid.dET14+comp1_xz_sys.T21*solid.dET24+comp1_xz_sys.T31*solid.dET34)*solid.pzm1.bN64+(comp1_xz_sys.T11*solid.dET15+comp1_xz_sys.T21*solid.dET25+comp1_xz_sys.T31*solid.dET35)*solid.pzm1.bN65+(comp1_xz_sys.T11*solid.dET16+comp1_xz_sys.T21*solid.dET26+comp1_xz_sys.T31*solid.dET36)*solid.pzm1.bN66 | C/N | Coupling matrix dET (global), Voigt notation, X6 component | Domain 5 |  |
| solid.dETgY6 | (comp1_xz_sys.T12*solid.dET11+comp1_xz_sys.T22*solid.dET21+comp1_xz_sys.T32*solid.dET31)*solid.pzm1.bN61+(comp1_xz_sys.T12*solid.dET12+comp1_xz_sys.T22*solid.dET22+comp1_xz_sys.T32*solid.dET32)*solid.pzm1.bN62+(comp1_xz_sys.T12*solid.dET13+comp1_xz_sys.T22*solid.dET23+comp1_xz_sys.T32*solid.dET33)*solid.pzm1.bN63+(comp1_xz_sys.T12*solid.dET14+comp1_xz_sys.T22*solid.dET24+comp1_xz_sys.T32*solid.dET34)*solid.pzm1.bN64+(comp1_xz_sys.T12*solid.dET15+comp1_xz_sys.T22*solid.dET25+comp1_xz_sys.T32*solid.dET35)*solid.pzm1.bN65+(comp1_xz_sys.T12*solid.dET16+comp1_xz_sys.T22*solid.dET26+comp1_xz_sys.T32*solid.dET36)*solid.pzm1.bN66 | C/N | Coupling matrix dET (global), Voigt notation, Y6 component | Domain 5 |  |
| solid.dETgZ6 | (comp1_xz_sys.T13*solid.dET11+comp1_xz_sys.T23*solid.dET21+comp1_xz_sys.T33*solid.dET31)*solid.pzm1.bN61+(comp1_xz_sys.T13*solid.dET12+comp1_xz_sys.T23*solid.dET22+comp1_xz_sys.T33*solid.dET32)*solid.pzm1.bN62+(comp1_xz_sys.T13*solid.dET13+comp1_xz_sys.T23*solid.dET23+comp1_xz_sys.T33*solid.dET33)*solid.pzm1.bN63+(comp1_xz_sys.T13*solid.dET14+comp1_xz_sys.T23*solid.dET24+comp1_xz_sys.T33*solid.dET34)*solid.pzm1.bN64+(comp1_xz_sys.T13*solid.dET15+comp1_xz_sys.T23*solid.dET25+comp1_xz_sys.T33*solid.dET35)*solid.pzm1.bN65+(comp1_xz_sys.T13*solid.dET16+comp1_xz_sys.T23*solid.dET26+comp1_xz_sys.T33*solid.dET36)*solid.pzm1.bN66 | C/N | Coupling matrix dET (global), Voigt notation, Z6 component | Domain 5 |  |
| solid.epsilonrSgXX | (comp1_xz_sys.T11*solid.epsilonrS11+comp1_xz_sys.T21*solid.epsilonrS12+comp1_xz_sys.T31*solid.epsilonrS13)*comp1_xz_sys.T11+(comp1_xz_sys.T11*solid.epsilonrS12+comp1_xz_sys.T21*solid.epsilonrS22+comp1_xz_sys.T31*solid.epsilonrS23)*comp1_xz_sys.T21+(comp1_xz_sys.T11*solid.epsilonrS13+comp1_xz_sys.T21*solid.epsilonrS23+comp1_xz_sys.T31*solid.epsilonrS33)*comp1_xz_sys.T31 | 1 | Relative permittivity at constant strain (global), XX-component | Domain 5 |  |
| solid.epsilonrSgXY | (comp1_xz_sys.T11*solid.epsilonrS11+comp1_xz_sys.T21*solid.epsilonrS12+comp1_xz_sys.T31*solid.epsilonrS13)*comp1_xz_sys.T12+(comp1_xz_sys.T11*solid.epsilonrS12+comp1_xz_sys.T21*solid.epsilonrS22+comp1_xz_sys.T31*solid.epsilonrS23)*comp1_xz_sys.T22+(comp1_xz_sys.T11*solid.epsilonrS13+comp1_xz_sys.T21*solid.epsilonrS23+comp1_xz_sys.T31*solid.epsilonrS33)*comp1_xz_sys.T32 | 1 | Relative permittivity at constant strain (global), XY-component | Domain 5 |  |
| solid.epsilonrSgXZ | (comp1_xz_sys.T11*solid.epsilonrS11+comp1_xz_sys.T21*solid.epsilonrS12+comp1_xz_sys.T31*solid.epsilonrS13)*comp1_xz_sys.T13+(comp1_xz_sys.T11*solid.epsilonrS12+comp1_xz_sys.T21*solid.epsilonrS22+comp1_xz_sys.T31*solid.epsilonrS23)*comp1_xz_sys.T23+(comp1_xz_sys.T11*solid.epsilonrS13+comp1_xz_sys.T21*solid.epsilonrS23+comp1_xz_sys.T31*solid.epsilonrS33)*comp1_xz_sys.T33 | 1 | Relative permittivity at constant strain (global), XZ-component | Domain 5 |  |
| solid.epsilonrSgYY | (comp1_xz_sys.T12*solid.epsilonrS11+comp1_xz_sys.T22*solid.epsilonrS12+comp1_xz_sys.T32*solid.epsilonrS13)*comp1_xz_sys.T12+(comp1_xz_sys.T12*solid.epsilonrS12+comp1_xz_sys.T22*solid.epsilonrS22+comp1_xz_sys.T32*solid.epsilonrS23)*comp1_xz_sys.T22+(comp1_xz_sys.T12*solid.epsilonrS13+comp1_xz_sys.T22*solid.epsilonrS23+comp1_xz_sys.T32*solid.epsilonrS33)*comp1_xz_sys.T32 | 1 | Relative permittivity at constant strain (global), YY-component | Domain 5 |  |
| solid.epsilonrSgYZ | (comp1_xz_sys.T12*solid.epsilonrS11+comp1_xz_sys.T22*solid.epsilonrS12+comp1_xz_sys.T32*solid.epsilonrS13)*comp1_xz_sys.T13+(comp1_xz_sys.T12*solid.epsilonrS12+comp1_xz_sys.T22*solid.epsilonrS22+comp1_xz_sys.T32*solid.epsilonrS23)*comp1_xz_sys.T23+(comp1_xz_sys.T12*solid.epsilonrS13+comp1_xz_sys.T22*solid.epsilonrS23+comp1_xz_sys.T32*solid.epsilonrS33)*comp1_xz_sys.T33 | 1 | Relative permittivity at constant strain (global), YZ-component | Domain 5 |  |
| solid.epsilonrSgZZ | (comp1_xz_sys.T13*solid.epsilonrS11+comp1_xz_sys.T23*solid.epsilonrS12+comp1_xz_sys.T33*solid.epsilonrS13)*comp1_xz_sys.T13+(comp1_xz_sys.T13*solid.epsilonrS12+comp1_xz_sys.T23*solid.epsilonrS22+comp1_xz_sys.T33*solid.epsilonrS23)*comp1_xz_sys.T23+(comp1_xz_sys.T13*solid.epsilonrS13+comp1_xz_sys.T23*solid.epsilonrS23+comp1_xz_sys.T33*solid.epsilonrS33)*comp1_xz_sys.T33 | 1 | Relative permittivity at constant strain (global), ZZ-component | Domain 5 |  |
| solid.epsilonrTgXX | (comp1_xz_sys.T11*solid.epsilonrT11+comp1_xz_sys.T21*solid.epsilonrT12+comp1_xz_sys.T31*solid.epsilonrT13)*comp1_xz_sys.T11+(comp1_xz_sys.T11*solid.epsilonrT12+comp1_xz_sys.T21*solid.epsilonrT22+comp1_xz_sys.T31*solid.epsilonrT23)*comp1_xz_sys.T21+(comp1_xz_sys.T11*solid.epsilonrT13+comp1_xz_sys.T21*solid.epsilonrT23+comp1_xz_sys.T31*solid.epsilonrT33)*comp1_xz_sys.T31 | 1 | Relative permittivity at constant stress (global), XX-component | Domain 5 |  |
| solid.epsilonrTgXY | (comp1_xz_sys.T11*solid.epsilonrT11+comp1_xz_sys.T21*solid.epsilonrT12+comp1_xz_sys.T31*solid.epsilonrT13)*comp1_xz_sys.T12+(comp1_xz_sys.T11*solid.epsilonrT12+comp1_xz_sys.T21*solid.epsilonrT22+comp1_xz_sys.T31*solid.epsilonrT23)*comp1_xz_sys.T22+(comp1_xz_sys.T11*solid.epsilonrT13+comp1_xz_sys.T21*solid.epsilonrT23+comp1_xz_sys.T31*solid.epsilonrT33)*comp1_xz_sys.T32 | 1 | Relative permittivity at constant stress (global), XY-component | Domain 5 |  |
| solid.epsilonrTgXZ | (comp1_xz_sys.T11*solid.epsilonrT11+comp1_xz_sys.T21*solid.epsilonrT12+comp1_xz_sys.T31*solid.epsilonrT13)*comp1_xz_sys.T13+(comp1_xz_sys.T11*solid.epsilonrT12+comp1_xz_sys.T21*solid.epsilonrT22+comp1_xz_sys.T31*solid.epsilonrT23)*comp1_xz_sys.T23+(comp1_xz_sys.T11*solid.epsilonrT13+comp1_xz_sys.T21*solid.epsilonrT23+comp1_xz_sys.T31*solid.epsilonrT33)*comp1_xz_sys.T33 | 1 | Relative permittivity at constant stress (global), XZ-component | Domain 5 |  |
| solid.epsilonrTgYY | (comp1_xz_sys.T12*solid.epsilonrT11+comp1_xz_sys.T22*solid.epsilonrT12+comp1_xz_sys.T32*solid.epsilonrT13)*comp1_xz_sys.T12+(comp1_xz_sys.T12*solid.epsilonrT12+comp1_xz_sys.T22*solid.epsilonrT22+comp1_xz_sys.T32*solid.epsilonrT23)*comp1_xz_sys.T22+(comp1_xz_sys.T12*solid.epsilonrT13+comp1_xz_sys.T22*solid.epsilonrT23+comp1_xz_sys.T32*solid.epsilonrT33)*comp1_xz_sys.T32 | 1 | Relative permittivity at constant stress (global), YY-component | Domain 5 |  |
| solid.epsilonrTgYZ | (comp1_xz_sys.T12*solid.epsilonrT11+comp1_xz_sys.T22*solid.epsilonrT12+comp1_xz_sys.T32*solid.epsilonrT13)*comp1_xz_sys.T13+(comp1_xz_sys.T12*solid.epsilonrT12+comp1_xz_sys.T22*solid.epsilonrT22+comp1_xz_sys.T32*solid.epsilonrT23)*comp1_xz_sys.T23+(comp1_xz_sys.T12*solid.epsilonrT13+comp1_xz_sys.T22*solid.epsilonrT23+comp1_xz_sys.T32*solid.epsilonrT33)*comp1_xz_sys.T33 | 1 | Relative permittivity at constant stress (global), YZ-component | Domain 5 |  |
| solid.epsilonrTgZZ | (comp1_xz_sys.T13*solid.epsilonrT11+comp1_xz_sys.T23*solid.epsilonrT12+comp1_xz_sys.T33*solid.epsilonrT13)*comp1_xz_sys.T13+(comp1_xz_sys.T13*solid.epsilonrT12+comp1_xz_sys.T23*solid.epsilonrT22+comp1_xz_sys.T33*solid.epsilonrT23)*comp1_xz_sys.T23+(comp1_xz_sys.T13*solid.epsilonrT13+comp1_xz_sys.T23*solid.epsilonrT23+comp1_xz_sys.T33*solid.epsilonrT33)*comp1_xz_sys.T33 | 1 | Relative permittivity at constant stress (global), ZZ-component | Domain 5 |  |
| solid.Jser0 | 0 | 1/Pa | Instantaneous shear compliance | Domain 5 | + operation |
| solid.Gpar0 | 0 |  | Instantaneous shear modulus | Domain 5 | + operation |
| solid.G0 | 1/(1/(solid.Gequ+solid.Gpar0)+solid.Jser0) | N/m² | Instantaneous shear modulus | Domain 5 |  |
| solid.Qh | 0.5*solid.omega*real((imag(solid.D11)*solid.eel11+imag(solid.D12)*solid.eel22+imag(solid.D13)*solid.eel33+2*imag(solid.D14)*solid.eel12+2*imag(solid.D15)*solid.eel23+2*imag(solid.D16)*solid.eel13)*conj(solid.eel11)+2*(imag(solid.D14)*solid.eel11+imag(solid.D24)*solid.eel22+imag(solid.D34)*solid.eel33+2*imag(solid.D44)*solid.eel12+2*imag(solid.D45)*solid.eel23+2*imag(solid.D46)*solid.eel13)*conj(solid.eel12)+2*(imag(solid.D16)*solid.eel11+imag(solid.D26)*solid.eel22+imag(solid.D36)*solid.eel33+2*imag(solid.D46)*solid.eel12+2*imag(solid.D56)*solid.eel23+2*imag(solid.D66)*solid.eel13)*conj(solid.eel13)+(imag(solid.D12)*solid.eel11+imag(solid.D22)*solid.eel22+imag(solid.D23)*solid.eel33+2*imag(solid.D24)*solid.eel12+2*imag(solid.D25)*solid.eel23+2*imag(solid.D26)*solid.eel13)*conj(solid.eel22)+2*(imag(solid.D15)*solid.eel11+imag(solid.D25)*solid.eel22+imag(solid.D35)*solid.eel33+2*imag(solid.D45)*solid.eel12+2*imag(solid.D55)*solid.eel23+2*imag(solid.D56)*solid.eel13)*conj(solid.eel23)+(imag(solid.D13)*solid.eel11+imag(solid.D23)*solid.eel22+imag(solid.D33)*solid.eel33+2*imag(solid.D34)*solid.eel12+2*imag(solid.D35)*solid.eel23+2*imag(solid.D36)*solid.eel13)*conj(solid.eel33))*!isPML | W/m³ | Total power dissipation density | Domain 5 | + operation |
| solid.rho | material.rho | kg/m³ | Density | Domain 5 | Meta, * operation |
| solid.cp | sqrt((solid.Kequ+4*solid.Gequ/3)/solid.rho) | m/s | Equivalent speed of pressure wave | Domain 5 |  |
| solid.cs | sqrt(solid.Gequ/solid.rho) | m/s | Equivalent speed of shear wave | Domain 5 |  |
| solid.Cgl11 | solid.D11+solid.D12+solid.D13 | Pa | C:g tensor, local coordinate system, 11-component | Domain 5 |  |
| solid.Cgl12 | solid.D14+solid.D24+solid.D34 | Pa | C:g tensor, local coordinate system, 12-component | Domain 5 |  |
| solid.Cgl13 | solid.D16+solid.D26+solid.D36 | Pa | C:g tensor, local coordinate system, 13-component | Domain 5 |  |
| solid.Cgl22 | solid.D12+solid.D22+solid.D23 | Pa | C:g tensor, local coordinate system, 22-component | Domain 5 |  |
| solid.Cgl23 | solid.D15+solid.D25+solid.D35 | Pa | C:g tensor, local coordinate system, 23-component | Domain 5 |  |
| solid.Cgl33 | solid.D13+solid.D23+solid.D33 | Pa | C:g tensor, local coordinate system, 33-component | Domain 5 |  |
| solid.elogxx | log(solid.stchp1)*solid.LW11^2+log(solid.stchp2)*solid.LW12^2+log(solid.stchp3)*solid.LW13^2 | 1 | Logarithmic strain tensor, xx-component | Domain 5 |  |
| solid.elogxy | log(solid.stchp1)*solid.LW11*solid.LW21+log(solid.stchp2)*solid.LW12*solid.LW22+log(solid.stchp3)*solid.LW13*solid.LW23 | 1 | Logarithmic strain tensor, xy-component | Domain 5 |  |
| solid.elogxz | log(solid.stchp1)*solid.LW11*solid.LW31+log(solid.stchp2)*solid.LW12*solid.LW32+log(solid.stchp3)*solid.LW13*solid.LW33 | 1 | Logarithmic strain tensor, xz-component | Domain 5 |  |
| solid.elogyy | log(solid.stchp1)*solid.LW21^2+log(solid.stchp2)*solid.LW22^2+log(solid.stchp3)*solid.LW23^2 | 1 | Logarithmic strain tensor, yy-component | Domain 5 |  |
| solid.elogyz | log(solid.stchp1)*solid.LW21*solid.LW31+log(solid.stchp2)*solid.LW22*solid.LW32+log(solid.stchp3)*solid.LW23*solid.LW33 | 1 | Logarithmic strain tensor, yz-component | Domain 5 |  |
| solid.elogzz | log(solid.stchp1)*solid.LW31^2+log(solid.stchp2)*solid.LW32^2+log(solid.stchp3)*solid.LW33^2 | 1 | Logarithmic strain tensor, zz-component | Domain 5 |  |
| solid.ellog11 | (solid.sysT11*solid.elogxx+solid.sysT12*solid.elogxy+solid.sysT13*solid.elogxz)*solid.sysT11+(solid.sysT11*solid.elogxy+solid.sysT12*solid.elogyy+solid.sysT13*solid.elogyz)*solid.sysT12+(solid.sysT11*solid.elogxz+solid.sysT12*solid.elogyz+solid.sysT13*solid.elogzz)*solid.sysT13 | 1 | Logarithmic strain tensor, local coordinate system, 11-component | Domain 5 |  |
| solid.ellog12 | (solid.sysT11*solid.elogxx+solid.sysT12*solid.elogxy+solid.sysT13*solid.elogxz)*solid.sysT21+(solid.sysT11*solid.elogxy+solid.sysT12*solid.elogyy+solid.sysT13*solid.elogyz)*solid.sysT22+(solid.sysT11*solid.elogxz+solid.sysT12*solid.elogyz+solid.sysT13*solid.elogzz)*solid.sysT23 | 1 | Logarithmic strain tensor, local coordinate system, 12-component | Domain 5 |  |
| solid.ellog13 | (solid.sysT11*solid.elogxx+solid.sysT12*solid.elogxy+solid.sysT13*solid.elogxz)*solid.sysT31+(solid.sysT11*solid.elogxy+solid.sysT12*solid.elogyy+solid.sysT13*solid.elogyz)*solid.sysT32+(solid.sysT11*solid.elogxz+solid.sysT12*solid.elogyz+solid.sysT13*solid.elogzz)*solid.sysT33 | 1 | Logarithmic strain tensor, local coordinate system, 13-component | Domain 5 |  |
| solid.ellog22 | (solid.sysT21*solid.elogxx+solid.sysT22*solid.elogxy+solid.sysT23*solid.elogxz)*solid.sysT21+(solid.sysT21*solid.elogxy+solid.sysT22*solid.elogyy+solid.sysT23*solid.elogyz)*solid.sysT22+(solid.sysT21*solid.elogxz+solid.sysT22*solid.elogyz+solid.sysT23*solid.elogzz)*solid.sysT23 | 1 | Logarithmic strain tensor, local coordinate system, 22-component | Domain 5 |  |
| solid.ellog23 | (solid.sysT21*solid.elogxx+solid.sysT22*solid.elogxy+solid.sysT23*solid.elogxz)*solid.sysT31+(solid.sysT21*solid.elogxy+solid.sysT22*solid.elogyy+solid.sysT23*solid.elogyz)*solid.sysT32+(solid.sysT21*solid.elogxz+solid.sysT22*solid.elogyz+solid.sysT23*solid.elogzz)*solid.sysT33 | 1 | Logarithmic strain tensor, local coordinate system, 23-component | Domain 5 |  |
| solid.ellog33 | (solid.sysT31*solid.elogxx+solid.sysT32*solid.elogxy+solid.sysT33*solid.elogxz)*solid.sysT31+(solid.sysT31*solid.elogxy+solid.sysT32*solid.elogyy+solid.sysT33*solid.elogyz)*solid.sysT32+(solid.sysT31*solid.elogxz+solid.sysT32*solid.elogyz+solid.sysT33*solid.elogzz)*solid.sysT33 | 1 | Logarithmic strain tensor, local coordinate system, 33-component | Domain 5 |  |
| solid.disp_rv | sqrt(u^2+v^2+eps) | m | Displacement magnitude | Domain 5 |  |
| solid.vel_rv | 2*solid.disp_rv*pi*solid.freq | m/s | Velocity magnitude | Domain 5 |  |
| solid.utt_rv | solid.disp_rv*(2*pi*solid.freq)^2 | m/s² | Acceleration magnitude | Domain 5 |  |
| solid.mises_rv | sqrt(3*solid.II2s+eps) | N/m² | von Mises stress | Domain 5 |  |
| solid.u_tt_rvX | -(2*pi*solid.freq)^2*u | m/s² | Acceleration, X-component | Domain 5 |  |
| solid.u_tt_rvY | -(2*pi*solid.freq)^2*v | m/s² | Acceleration, Y-component | Domain 5 |  |
| solid.u_tt_rvZ | 0 | m/s² | Acceleration, Z-component | Domain 5 |  |
| solid.u_ttX | material.dt(material.dt(u)) | m/s² | Acceleration, X-component | Domain 5 |  |
| solid.u_ttY | material.dt(material.dt(v)) | m/s² | Acceleration, Y-component | Domain 5 |  |
| solid.u_ttZ | material.dt(material.dt(0)) | m/s² | Acceleration, Z-component | Domain 5 |  |
| solid.u_tX | material.dt(u) | m/s | Velocity, X-component | Domain 5 |  |
| solid.u_tY | material.dt(v) | m/s | Velocity, Y-component | Domain 5 |  |
| solid.u_tZ | material.dt(0) | m/s | Velocity, Z-component | Domain 5 |  |
| solid.vel_rms | sqrt(0.5*(realdot(solid.u_tX,solid.u_tX)+realdot(solid.u_tY,solid.u_tY)+realdot(solid.u_tZ,solid.u_tZ))) | m/s | Velocity magnitude, RMS | Domain 5 |  |
| solid.acc_rms | sqrt(0.5*(realdot(solid.u_ttX,solid.u_ttX)+realdot(solid.u_ttY,solid.u_ttY)+realdot(solid.u_ttZ,solid.u_ttZ))) | m/s² | Acceleration magnitude, RMS | Domain 5 |  |
| solid.uAmpX | abs(u) | m | Displacement amplitude, X-component | Domain 5 |  |
| solid.uAmpY | abs(v) | m | Displacement amplitude, Y-component | Domain 5 |  |
| solid.uAmpZ | 0 | m | Displacement amplitude, Z-component | Domain 5 |  |
| solid.uAmp_tX | solid.uAmpX*abs(solid.omega) | m/s | Velocity amplitude, X-component | Domain 5 |  |
| solid.uAmp_tY | solid.uAmpY*abs(solid.omega) | m/s | Velocity amplitude, Y-component | Domain 5 |  |
| solid.uAmp_tZ | solid.uAmpZ*abs(solid.omega) | m/s | Velocity amplitude, Z-component | Domain 5 |  |
| solid.uAmp_ttX | solid.uAmpX*solid.omega^2 | m/s² | Acceleration amplitude, X-component | Domain 5 |  |
| solid.uAmp_ttY | solid.uAmpY*solid.omega^2 | m/s² | Acceleration amplitude, Y-component | Domain 5 |  |
| solid.uAmp_ttZ | solid.uAmpZ*solid.omega^2 | m/s² | Acceleration amplitude, Z-component | Domain 5 |  |
| solid.uPhaseX | mod(arg(u),2*pi) | rad | Displacement phase, X-component | Domain 5 |  |
| solid.uPhaseY | mod(arg(v),2*pi) | rad | Displacement phase, Y-component | Domain 5 |  |
| solid.uPhaseZ | mod(arg(0),2*pi) | rad | Displacement phase, Z-component | Domain 5 |  |
| solid.uPhase_tX | mod(0.5*pi+solid.uPhaseX,2*pi) | rad | Velocity phase, X-component | Domain 5 |  |
| solid.uPhase_tY | mod(0.5*pi+solid.uPhaseY,2*pi) | rad | Velocity phase, Y-component | Domain 5 |  |
| solid.uPhase_tZ | mod(0.5*pi+solid.uPhaseZ,2*pi) | rad | Velocity phase, Z-component | Domain 5 |  |
| solid.uPhase_ttX | mod(pi+solid.uPhaseX,2*pi) | rad | Acceleration phase, X-component | Domain 5 |  |
| solid.uPhase_ttY | mod(pi+solid.uPhaseY,2*pi) | rad | Acceleration phase, Y-component | Domain 5 |  |
| solid.uPhase_ttZ | mod(pi+solid.uPhaseZ,2*pi) | rad | Acceleration phase, Z-component | Domain 5 |  |
| solid.disp_peak | sqrteps(0.5*(solid.uAmpX^2+solid.uAmpY^2+solid.uAmpZ^2+sqrteps((comp1_xz_sys.invT11*solid.uAmpX^2+comp1_xz_sys.invT21*solid.uAmpY^2+comp1_xz_sys.invT31*solid.uAmpZ^2)*(comp1_xz_sys.T11*solid.uAmpX^2+comp1_xz_sys.T12*solid.uAmpY^2+comp1_xz_sys.T13*solid.uAmpZ^2+cos(2*(solid.uPhaseX-solid.uPhaseY))*(comp1_xz_sys.T21*solid.uAmpX^2+comp1_xz_sys.T22*solid.uAmpY^2+comp1_xz_sys.T23*solid.uAmpZ^2)+cos(2*(solid.uPhaseX-solid.uPhaseZ))*(comp1_xz_sys.T31*solid.uAmpX^2+comp1_xz_sys.T32*solid.uAmpY^2+comp1_xz_sys.T33*solid.uAmpZ^2))+(comp1_xz_sys.invT12*solid.uAmpX^2+comp1_xz_sys.invT22*solid.uAmpY^2+comp1_xz_sys.invT32*solid.uAmpZ^2)*(cos(2*(solid.uPhaseX-solid.uPhaseY))*(comp1_xz_sys.T11*solid.uAmpX^2+comp1_xz_sys.T12*solid.uAmpY^2+comp1_xz_sys.T13*solid.uAmpZ^2)+comp1_xz_sys.T21*solid.uAmpX^2+comp1_xz_sys.T22*solid.uAmpY^2+comp1_xz_sys.T23*solid.uAmpZ^2+cos(2*(solid.uPhaseY-solid.uPhaseZ))*(comp1_xz_sys.T31*solid.uAmpX^2+comp1_xz_sys.T32*solid.uAmpY^2+comp1_xz_sys.T33*solid.uAmpZ^2))+(comp1_xz_sys.invT13*solid.uAmpX^2+comp1_xz_sys.invT23*solid.uAmpY^2+comp1_xz_sys.invT33*solid.uAmpZ^2)*(cos(2*(solid.uPhaseX-solid.uPhaseZ))*(comp1_xz_sys.T11*solid.uAmpX^2+comp1_xz_sys.T12*solid.uAmpY^2+comp1_xz_sys.T13*solid.uAmpZ^2)+cos(2*(solid.uPhaseY-solid.uPhaseZ))*(comp1_xz_sys.T21*solid.uAmpX^2+comp1_xz_sys.T22*solid.uAmpY^2+comp1_xz_sys.T23*solid.uAmpZ^2)+comp1_xz_sys.T31*solid.uAmpX^2+comp1_xz_sys.T32*solid.uAmpY^2+comp1_xz_sys.T33*solid.uAmpZ^2)))) | m | Displacement magnitude, peak | Domain 5 |  |
| solid.vel_peak | sqrteps(0.5*(solid.uAmp_tX^2+solid.uAmp_tY^2+solid.uAmp_tZ^2+sqrteps((comp1_xz_sys.invT11*solid.uAmp_tX^2+comp1_xz_sys.invT21*solid.uAmp_tY^2+comp1_xz_sys.invT31*solid.uAmp_tZ^2)*(comp1_xz_sys.T11*solid.uAmp_tX^2+comp1_xz_sys.T12*solid.uAmp_tY^2+comp1_xz_sys.T13*solid.uAmp_tZ^2+cos(2*(solid.uPhase_tX-solid.uPhase_tY))*(comp1_xz_sys.T21*solid.uAmp_tX^2+comp1_xz_sys.T22*solid.uAmp_tY^2+comp1_xz_sys.T23*solid.uAmp_tZ^2)+cos(2*(solid.uPhase_tX-solid.uPhase_tZ))*(comp1_xz_sys.T31*solid.uAmp_tX^2+comp1_xz_sys.T32*solid.uAmp_tY^2+comp1_xz_sys.T33*solid.uAmp_tZ^2))+(comp1_xz_sys.invT12*solid.uAmp_tX^2+comp1_xz_sys.invT22*solid.uAmp_tY^2+comp1_xz_sys.invT32*solid.uAmp_tZ^2)*(cos(2*(solid.uPhase_tX-solid.uPhase_tY))*(comp1_xz_sys.T11*solid.uAmp_tX^2+comp1_xz_sys.T12*solid.uAmp_tY^2+comp1_xz_sys.T13*solid.uAmp_tZ^2)+comp1_xz_sys.T21*solid.uAmp_tX^2+comp1_xz_sys.T22*solid.uAmp_tY^2+comp1_xz_sys.T23*solid.uAmp_tZ^2+cos(2*(solid.uPhase_tY-solid.uPhase_tZ))*(comp1_xz_sys.T31*solid.uAmp_tX^2+comp1_xz_sys.T32*solid.uAmp_tY^2+comp1_xz_sys.T33*solid.uAmp_tZ^2))+(comp1_xz_sys.invT13*solid.uAmp_tX^2+comp1_xz_sys.invT23*solid.uAmp_tY^2+comp1_xz_sys.invT33*solid.uAmp_tZ^2)*(cos(2*(solid.uPhase_tX-solid.uPhase_tZ))*(comp1_xz_sys.T11*solid.uAmp_tX^2+comp1_xz_sys.T12*solid.uAmp_tY^2+comp1_xz_sys.T13*solid.uAmp_tZ^2)+cos(2*(solid.uPhase_tY-solid.uPhase_tZ))*(comp1_xz_sys.T21*solid.uAmp_tX^2+comp1_xz_sys.T22*solid.uAmp_tY^2+comp1_xz_sys.T23*solid.uAmp_tZ^2)+comp1_xz_sys.T31*solid.uAmp_tX^2+comp1_xz_sys.T32*solid.uAmp_tY^2+comp1_xz_sys.T33*solid.uAmp_tZ^2)))) | m/s | Velocity magnitude, peak | Domain 5 |  |
| solid.u_tt_peak | sqrteps(0.5*(solid.uAmp_ttX^2+solid.uAmp_ttY^2+solid.uAmp_ttZ^2+sqrteps((comp1_xz_sys.invT11*solid.uAmp_ttX^2+comp1_xz_sys.invT21*solid.uAmp_ttY^2+comp1_xz_sys.invT31*solid.uAmp_ttZ^2)*(comp1_xz_sys.T11*solid.uAmp_ttX^2+comp1_xz_sys.T12*solid.uAmp_ttY^2+comp1_xz_sys.T13*solid.uAmp_ttZ^2+cos(2*(solid.uPhase_ttX-solid.uPhase_ttY))*(comp1_xz_sys.T21*solid.uAmp_ttX^2+comp1_xz_sys.T22*solid.uAmp_ttY^2+comp1_xz_sys.T23*solid.uAmp_ttZ^2)+cos(2*(solid.uPhase_ttX-solid.uPhase_ttZ))*(comp1_xz_sys.T31*solid.uAmp_ttX^2+comp1_xz_sys.T32*solid.uAmp_ttY^2+comp1_xz_sys.T33*solid.uAmp_ttZ^2))+(comp1_xz_sys.invT12*solid.uAmp_ttX^2+comp1_xz_sys.invT22*solid.uAmp_ttY^2+comp1_xz_sys.invT32*solid.uAmp_ttZ^2)*(cos(2*(solid.uPhase_ttX-solid.uPhase_ttY))*(comp1_xz_sys.T11*solid.uAmp_ttX^2+comp1_xz_sys.T12*solid.uAmp_ttY^2+comp1_xz_sys.T13*solid.uAmp_ttZ^2)+comp1_xz_sys.T21*solid.uAmp_ttX^2+comp1_xz_sys.T22*solid.uAmp_ttY^2+comp1_xz_sys.T23*solid.uAmp_ttZ^2+cos(2*(solid.uPhase_ttY-solid.uPhase_ttZ))*(comp1_xz_sys.T31*solid.uAmp_ttX^2+comp1_xz_sys.T32*solid.uAmp_ttY^2+comp1_xz_sys.T33*solid.uAmp_ttZ^2))+(comp1_xz_sys.invT13*solid.uAmp_ttX^2+comp1_xz_sys.invT23*solid.uAmp_ttY^2+comp1_xz_sys.invT33*solid.uAmp_ttZ^2)*(cos(2*(solid.uPhase_ttX-solid.uPhase_ttZ))*(comp1_xz_sys.T11*solid.uAmp_ttX^2+comp1_xz_sys.T12*solid.uAmp_ttY^2+comp1_xz_sys.T13*solid.uAmp_ttZ^2)+cos(2*(solid.uPhase_ttY-solid.uPhase_ttZ))*(comp1_xz_sys.T21*solid.uAmp_ttX^2+comp1_xz_sys.T22*solid.uAmp_ttY^2+comp1_xz_sys.T23*solid.uAmp_ttZ^2)+comp1_xz_sys.T31*solid.uAmp_ttX^2+comp1_xz_sys.T32*solid.uAmp_ttY^2+comp1_xz_sys.T33*solid.uAmp_ttZ^2)))) | m/s² | Acceleration magnitude, peak | Domain 5 |  |
| solid.afX | 0 | m/s² | Frame acceleration, X-component | Domain 5 | + operation |
| solid.afY | 0 | m/s² | Frame acceleration, Y-component | Domain 5 | + operation |
| solid.afZ | 0 | m/s² | Frame acceleration, Z-component | Domain 5 | + operation |
| solid.accX | solid.u_ttX | m/s² | Effective acceleration, X-component | Domain 5 | + operation |
| solid.accY | solid.u_ttY | m/s² | Effective acceleration, Y-component | Domain 5 | + operation |
| solid.accZ | solid.u_ttZ | m/s² | Effective acceleration, Z-component | Domain 5 | + operation |
| solid.vel | sqrteps(real(solid.u_tX)^2+real(solid.u_tY)^2+real(solid.u_tZ)^2) | m/s | Velocity magnitude | Domain 5 |  |
| solid.acc | sqrteps(real(solid.accX)^2+real(solid.accY)^2+real(solid.accZ)^2) | m/s² | Effective acceleration magnitude | Domain 5 |  |
| solid.u_tt | sqrteps(real(solid.u_ttX)^2+real(solid.u_ttY)^2+real(solid.u_ttZ)^2) | m/s² | Acceleration magnitude | Domain 5 |  |
| solid.Tax | mean(solid.sxx)*solid.nx+mean(solid.sxy)*solid.ny+mean(solid.sxz)*solid.nz | N/m² | Traction (force/area), x-component | Boundaries 13–15, 21 |  |
| solid.Tay | mean(solid.sxy)*solid.nx+mean(solid.syy)*solid.ny+mean(solid.syz)*solid.nz | N/m² | Traction (force/area), y-component | Boundaries 13–15, 21 |  |
| solid.Taz | mean(solid.sxz)*solid.nx+mean(solid.syz)*solid.ny+mean(solid.szz)*solid.nz | N/m² | Traction (force/area), z-component | Boundaries 13–15, 21 |  |
| solid.stn | solid.Tax*solid.nx+solid.Tay*solid.ny+solid.Taz*solid.nz | N/m² | Normal stress | Boundaries 13–15, 21 |  |
| solid.Tanx | solid.stn*solid.nx | N/m² | Normal component of traction, x-component | Boundaries 13–15, 21 |  |
| solid.Tany | solid.stn*solid.ny | N/m² | Normal component of traction, y-component | Boundaries 13–15, 21 |  |
| solid.Tanz | solid.stn*solid.nz | N/m² | Normal component of traction, z-component | Boundaries 13–15, 21 |  |
| solid.Tatx | solid.Tax-solid.Tanx | N/m² | Shear component of traction, x-component | Boundaries 13–15, 21 |  |
| solid.Taty | solid.Tay-solid.Tany | N/m² | Shear component of traction, y-component | Boundaries 13–15, 21 |  |
| solid.Tatz | solid.Taz-solid.Tanz | N/m² | Shear component of traction, z-component | Boundaries 13–15, 21 |  |
| solid.stt | sqrt(solid.Tatx^2+solid.Taty^2+solid.Tatz^2) | N/m² | Shear stress | Boundaries 13–15, 21 |  |
| solid.sysT11 | comp1_xz_sys.T11 | 1 | Transform to global system, 11-component | Domain 5 |  |
| solid.sysT21 | comp1_xz_sys.T21 | 1 | Transform to global system, 21-component | Domain 5 |  |
| solid.sysT31 | comp1_xz_sys.T31 | 1 | Transform to global system, 31-component | Domain 5 |  |
| solid.sysT12 | comp1_xz_sys.T12 | 1 | Transform to global system, 12-component | Domain 5 |  |
| solid.sysT22 | comp1_xz_sys.T22 | 1 | Transform to global system, 22-component | Domain 5 |  |
| solid.sysT32 | comp1_xz_sys.T32 | 1 | Transform to global system, 32-component | Domain 5 |  |
| solid.sysT13 | comp1_xz_sys.T13 | 1 | Transform to global system, 13-component | Domain 5 |  |
| solid.sysT23 | comp1_xz_sys.T23 | 1 | Transform to global system, 23-component | Domain 5 |  |
| solid.sysT33 | comp1_xz_sys.T33 | 1 | Transform to global system, 33-component | Domain 5 |  |
| uTXt | d(uTX,TIME) | 1/s | Tangential gradient of u, X component, first time derivative | Boundaries 13–15, 21 |  |
| uTYt | d(uTY,TIME) | 1/s | Tangential gradient of u, Y component, first time derivative | Boundaries 13–15, 21 |  |
| uTXtt | d(d(uTX,TIME),TIME) | 1/s² | Tangential gradient of u, X component, second time derivative | Boundaries 13–15, 21 |  |
| uTYtt | d(d(uTY,TIME),TIME) | 1/s² | Tangential gradient of u, Y component, second time derivative | Boundaries 13–15, 21 |  |
| uXt | d(uX,TIME) | 1/s | Gradient of u, X component, first time derivative | Domain 5 |  |
| uYt | d(uY,TIME) | 1/s | Gradient of u, Y component, first time derivative | Domain 5 |  |
| uXtt | d(d(uX,TIME),TIME) | 1/s² | Gradient of u, X component, second time derivative | Domain 5 |  |
| uYtt | d(d(uY,TIME),TIME) | 1/s² | Gradient of u, Y component, second time derivative | Domain 5 |  |
| vTXt | d(vTX,TIME) | 1/s | Tangential gradient of v, X component, first time derivative | Boundaries 13–15, 21 |  |
| vTYt | d(vTY,TIME) | 1/s | Tangential gradient of v, Y component, first time derivative | Boundaries 13–15, 21 |  |
| vTXtt | d(d(vTX,TIME),TIME) | 1/s² | Tangential gradient of v, X component, second time derivative | Boundaries 13–15, 21 |  |
| vTYtt | d(d(vTY,TIME),TIME) | 1/s² | Tangential gradient of v, Y component, second time derivative | Boundaries 13–15, 21 |  |
| vXt | d(vX,TIME) | 1/s | Gradient of v, X component, first time derivative | Domain 5 |  |
| vYt | d(vY,TIME) | 1/s | Gradient of v, Y component, first time derivative | Domain 5 |  |
| vXtt | d(d(vX,TIME),TIME) | 1/s² | Gradient of v, X component, second time derivative | Domain 5 |  |
| vYtt | d(d(vY,TIME),TIME) | 1/s² | Gradient of v, Y component, second time derivative | Domain 5 |  |
| ut | d(u,TIME) | m/s | Structural velocity field, X-component | Domain 5 |  |
| vt | d(v,TIME) | m/s | Structural velocity field, Y-component | Domain 5 |  |
| utt | d(d(u,TIME),TIME) | m/s² | Acceleration field, X-component | Domain 5 |  |
| vtt | d(d(v,TIME),TIME) | m/s² | Acceleration field, Y-component | Domain 5 |  |
| solid.Dr1 | 0 | C/m² | Remanent electric displacement, 1-component | Domain 5 |  |
| solid.Dr2 | 0 | C/m² | Remanent electric displacement, 2-component | Domain 5 |  |
| solid.Dr3 | 0 | C/m² | Remanent electric displacement, 3-component | Domain 5 |  |
| solid.cE11 | material.cE11 | Pa | Elasticity matrix cE, Voigt notation, 11-component | Domain 5 | Meta, * operation |
| solid.cE12 | material.cE12 | Pa | Elasticity matrix cE, Voigt notation, 12-component | Domain 5 | Meta, * operation |
| solid.cE13 | material.cE13 | Pa | Elasticity matrix cE, Voigt notation, 13-component | Domain 5 | Meta, * operation |
| solid.cE14 | material.cE14 | Pa | Elasticity matrix cE, Voigt notation, 14-component | Domain 5 | Meta, * operation |
| solid.cE15 | material.cE15 | Pa | Elasticity matrix cE, Voigt notation, 15-component | Domain 5 | Meta, * operation |
| solid.cE16 | material.cE16 | Pa | Elasticity matrix cE, Voigt notation, 16-component | Domain 5 | Meta, * operation |
| solid.cE22 | material.cE22 | Pa | Elasticity matrix cE, Voigt notation, 22-component | Domain 5 | Meta, * operation |
| solid.cE23 | material.cE23 | Pa | Elasticity matrix cE, Voigt notation, 23-component | Domain 5 | Meta, * operation |
| solid.cE24 | material.cE24 | Pa | Elasticity matrix cE, Voigt notation, 24-component | Domain 5 | Meta, * operation |
| solid.cE25 | material.cE25 | Pa | Elasticity matrix cE, Voigt notation, 25-component | Domain 5 | Meta, * operation |
| solid.cE26 | material.cE26 | Pa | Elasticity matrix cE, Voigt notation, 26-component | Domain 5 | Meta, * operation |
| solid.cE33 | material.cE33 | Pa | Elasticity matrix cE, Voigt notation, 33-component | Domain 5 | Meta, * operation |
| solid.cE34 | material.cE34 | Pa | Elasticity matrix cE, Voigt notation, 34-component | Domain 5 | Meta, * operation |
| solid.cE35 | material.cE35 | Pa | Elasticity matrix cE, Voigt notation, 35-component | Domain 5 | Meta, * operation |
| solid.cE36 | material.cE36 | Pa | Elasticity matrix cE, Voigt notation, 36-component | Domain 5 | Meta, * operation |
| solid.cE44 | material.cE44 | Pa | Elasticity matrix cE, Voigt notation, 44-component | Domain 5 | Meta, * operation |
| solid.cE45 | material.cE45 | Pa | Elasticity matrix cE, Voigt notation, 45-component | Domain 5 | Meta, * operation |
| solid.cE46 | material.cE46 | Pa | Elasticity matrix cE, Voigt notation, 46-component | Domain 5 | Meta, * operation |
| solid.cE55 | material.cE55 | Pa | Elasticity matrix cE, Voigt notation, 55-component | Domain 5 | Meta, * operation |
| solid.cE56 | material.cE56 | Pa | Elasticity matrix cE, Voigt notation, 56-component | Domain 5 | Meta, * operation |
| solid.cE66 | material.cE66 | Pa | Elasticity matrix cE, Voigt notation, 66-component | Domain 5 | Meta, * operation |
| solid.eES11 | material.eES11 | C/m² | Coupling matrix eES, Voigt notation, 11-component | Domain 5 | Meta, * operation |
| solid.eES21 | material.eES21 | C/m² | Coupling matrix eES, Voigt notation, 21-component | Domain 5 | Meta, * operation |
| solid.eES31 | material.eES31 | C/m² | Coupling matrix eES, Voigt notation, 31-component | Domain 5 | Meta, * operation |
| solid.eES12 | material.eES12 | C/m² | Coupling matrix eES, Voigt notation, 12-component | Domain 5 | Meta, * operation |
| solid.eES22 | material.eES22 | C/m² | Coupling matrix eES, Voigt notation, 22-component | Domain 5 | Meta, * operation |
| solid.eES32 | material.eES32 | C/m² | Coupling matrix eES, Voigt notation, 32-component | Domain 5 | Meta, * operation |
| solid.eES13 | material.eES13 | C/m² | Coupling matrix eES, Voigt notation, 13-component | Domain 5 | Meta, * operation |
| solid.eES23 | material.eES23 | C/m² | Coupling matrix eES, Voigt notation, 23-component | Domain 5 | Meta, * operation |
| solid.eES33 | material.eES33 | C/m² | Coupling matrix eES, Voigt notation, 33-component | Domain 5 | Meta, * operation |
| solid.eES14 | material.eES14 | C/m² | Coupling matrix eES, Voigt notation, 14-component | Domain 5 | Meta, * operation |
| solid.eES24 | material.eES24 | C/m² | Coupling matrix eES, Voigt notation, 24-component | Domain 5 | Meta, * operation |
| solid.eES34 | material.eES34 | C/m² | Coupling matrix eES, Voigt notation, 34-component | Domain 5 | Meta, * operation |
| solid.eES15 | material.eES15 | C/m² | Coupling matrix eES, Voigt notation, 15-component | Domain 5 | Meta, * operation |
| solid.eES25 | material.eES25 | C/m² | Coupling matrix eES, Voigt notation, 25-component | Domain 5 | Meta, * operation |
| solid.eES35 | material.eES35 | C/m² | Coupling matrix eES, Voigt notation, 35-component | Domain 5 | Meta, * operation |
| solid.eES16 | material.eES16 | C/m² | Coupling matrix eES, Voigt notation, 16-component | Domain 5 | Meta, * operation |
| solid.eES26 | material.eES26 | C/m² | Coupling matrix eES, Voigt notation, 26-component | Domain 5 | Meta, * operation |
| solid.eES36 | material.eES36 | C/m² | Coupling matrix eES, Voigt notation, 36-component | Domain 5 | Meta, * operation |
| solid.dET11 | solid.eES11*solid.sE11+solid.eES12*solid.sE12+solid.eES13*solid.sE13+solid.eES14*solid.sE14+solid.eES15*solid.sE15+solid.eES16*solid.sE16 | C/N | Coupling matrix dET, Voigt notation, 11-component | Domain 5 | * operation |
| solid.dET21 | solid.eES21*solid.sE11+solid.eES22*solid.sE12+solid.eES23*solid.sE13+solid.eES24*solid.sE14+solid.eES25*solid.sE15+solid.eES26*solid.sE16 | C/N | Coupling matrix dET, Voigt notation, 21-component | Domain 5 | * operation |
| solid.dET31 | solid.eES31*solid.sE11+solid.eES32*solid.sE12+solid.eES33*solid.sE13+solid.eES34*solid.sE14+solid.eES35*solid.sE15+solid.eES36*solid.sE16 | C/N | Coupling matrix dET, Voigt notation, 31-component | Domain 5 | * operation |
| solid.dET12 | solid.eES11*solid.sE12+solid.eES12*solid.sE22+solid.eES13*solid.sE23+solid.eES14*solid.sE24+solid.eES15*solid.sE25+solid.eES16*solid.sE26 | C/N | Coupling matrix dET, Voigt notation, 12-component | Domain 5 | * operation |
| solid.dET22 | solid.eES21*solid.sE12+solid.eES22*solid.sE22+solid.eES23*solid.sE23+solid.eES24*solid.sE24+solid.eES25*solid.sE25+solid.eES26*solid.sE26 | C/N | Coupling matrix dET, Voigt notation, 22-component | Domain 5 | * operation |
| solid.dET32 | solid.eES31*solid.sE12+solid.eES32*solid.sE22+solid.eES33*solid.sE23+solid.eES34*solid.sE24+solid.eES35*solid.sE25+solid.eES36*solid.sE26 | C/N | Coupling matrix dET, Voigt notation, 32-component | Domain 5 | * operation |
| solid.dET13 | solid.eES11*solid.sE13+solid.eES12*solid.sE23+solid.eES13*solid.sE33+solid.eES14*solid.sE34+solid.eES15*solid.sE35+solid.eES16*solid.sE36 | C/N | Coupling matrix dET, Voigt notation, 13-component | Domain 5 | * operation |
| solid.dET23 | solid.eES21*solid.sE13+solid.eES22*solid.sE23+solid.eES23*solid.sE33+solid.eES24*solid.sE34+solid.eES25*solid.sE35+solid.eES26*solid.sE36 | C/N | Coupling matrix dET, Voigt notation, 23-component | Domain 5 | * operation |
| solid.dET33 | solid.eES31*solid.sE13+solid.eES32*solid.sE23+solid.eES33*solid.sE33+solid.eES34*solid.sE34+solid.eES35*solid.sE35+solid.eES36*solid.sE36 | C/N | Coupling matrix dET, Voigt notation, 33-component | Domain 5 | * operation |
| solid.dET14 | solid.eES11*solid.sE14+solid.eES12*solid.sE24+solid.eES13*solid.sE34+solid.eES14*solid.sE44+solid.eES15*solid.sE45+solid.eES16*solid.sE46 | C/N | Coupling matrix dET, Voigt notation, 14-component | Domain 5 | * operation |
| solid.dET24 | solid.eES21*solid.sE14+solid.eES22*solid.sE24+solid.eES23*solid.sE34+solid.eES24*solid.sE44+solid.eES25*solid.sE45+solid.eES26*solid.sE46 | C/N | Coupling matrix dET, Voigt notation, 24-component | Domain 5 | * operation |
| solid.dET34 | solid.eES31*solid.sE14+solid.eES32*solid.sE24+solid.eES33*solid.sE34+solid.eES34*solid.sE44+solid.eES35*solid.sE45+solid.eES36*solid.sE46 | C/N | Coupling matrix dET, Voigt notation, 34-component | Domain 5 | * operation |
| solid.dET15 | solid.eES11*solid.sE15+solid.eES12*solid.sE25+solid.eES13*solid.sE35+solid.eES14*solid.sE45+solid.eES15*solid.sE55+solid.eES16*solid.sE56 | C/N | Coupling matrix dET, Voigt notation, 15-component | Domain 5 | * operation |
| solid.dET25 | solid.eES21*solid.sE15+solid.eES22*solid.sE25+solid.eES23*solid.sE35+solid.eES24*solid.sE45+solid.eES25*solid.sE55+solid.eES26*solid.sE56 | C/N | Coupling matrix dET, Voigt notation, 25-component | Domain 5 | * operation |
| solid.dET35 | solid.eES31*solid.sE15+solid.eES32*solid.sE25+solid.eES33*solid.sE35+solid.eES34*solid.sE45+solid.eES35*solid.sE55+solid.eES36*solid.sE56 | C/N | Coupling matrix dET, Voigt notation, 35-component | Domain 5 | * operation |
| solid.dET16 | solid.eES11*solid.sE16+solid.eES12*solid.sE26+solid.eES13*solid.sE36+solid.eES14*solid.sE46+solid.eES15*solid.sE56+solid.eES16*solid.sE66 | C/N | Coupling matrix dET, Voigt notation, 16-component | Domain 5 | * operation |
| solid.dET26 | solid.eES21*solid.sE16+solid.eES22*solid.sE26+solid.eES23*solid.sE36+solid.eES24*solid.sE46+solid.eES25*solid.sE56+solid.eES26*solid.sE66 | C/N | Coupling matrix dET, Voigt notation, 26-component | Domain 5 | * operation |
| solid.dET36 | solid.eES31*solid.sE16+solid.eES32*solid.sE26+solid.eES33*solid.sE36+solid.eES34*solid.sE46+solid.eES35*solid.sE56+solid.eES36*solid.sE66 | C/N | Coupling matrix dET, Voigt notation, 36-component | Domain 5 | * operation |
| solid.epsilonrS11 | material.epsilonrS11 | 1 | Relative permittivity at constant strain, 11-component | Domain 5 | Meta, * operation |
| solid.epsilonrS12 | material.epsilonrS12 | 1 | Relative permittivity at constant strain, 12-component | Domain 5 | Meta, * operation |
| solid.epsilonrS13 | material.epsilonrS13 | 1 | Relative permittivity at constant strain, 13-component | Domain 5 | Meta, * operation |
| solid.epsilonrS22 | material.epsilonrS22 | 1 | Relative permittivity at constant strain, 22-component | Domain 5 | Meta, * operation |
| solid.epsilonrS23 | material.epsilonrS23 | 1 | Relative permittivity at constant strain, 23-component | Domain 5 | Meta, * operation |
| solid.epsilonrS33 | material.epsilonrS33 | 1 | Relative permittivity at constant strain, 33-component | Domain 5 | Meta, * operation |
| solid.epsilonrT11 | solid.epsilonrS11+((solid.dET11*solid.cE11+solid.dET12*solid.cE12+solid.dET13*solid.cE13+solid.dET14*solid.cE14+solid.dET15*solid.cE15+solid.dET16*solid.cE16)*solid.dET11+(solid.dET11*solid.cE12+solid.dET12*solid.cE22+solid.dET13*solid.cE23+solid.dET14*solid.cE24+solid.dET15*solid.cE25+solid.dET16*solid.cE26)*solid.dET12+(solid.dET11*solid.cE13+solid.dET12*solid.cE23+solid.dET13*solid.cE33+solid.dET14*solid.cE34+solid.dET15*solid.cE35+solid.dET16*solid.cE36)*solid.dET13+(solid.dET11*solid.cE14+solid.dET12*solid.cE24+solid.dET13*solid.cE34+solid.dET14*solid.cE44+solid.dET15*solid.cE45+solid.dET16*solid.cE46)*solid.dET14+(solid.dET11*solid.cE15+solid.dET12*solid.cE25+solid.dET13*solid.cE35+solid.dET14*solid.cE45+solid.dET15*solid.cE55+solid.dET16*solid.cE56)*solid.dET15+(solid.dET11*solid.cE16+solid.dET12*solid.cE26+solid.dET13*solid.cE36+solid.dET14*solid.cE46+solid.dET15*solid.cE56+solid.dET16*solid.cE66)*solid.dET16)/epsilon0_const | 1 | Relative permittivity at constant stress, 11-component | Domain 5 | * operation |
| solid.epsilonrT12 | solid.epsilonrS12+((solid.dET11*solid.cE11+solid.dET12*solid.cE12+solid.dET13*solid.cE13+solid.dET14*solid.cE14+solid.dET15*solid.cE15+solid.dET16*solid.cE16)*solid.dET21+(solid.dET11*solid.cE12+solid.dET12*solid.cE22+solid.dET13*solid.cE23+solid.dET14*solid.cE24+solid.dET15*solid.cE25+solid.dET16*solid.cE26)*solid.dET22+(solid.dET11*solid.cE13+solid.dET12*solid.cE23+solid.dET13*solid.cE33+solid.dET14*solid.cE34+solid.dET15*solid.cE35+solid.dET16*solid.cE36)*solid.dET23+(solid.dET11*solid.cE14+solid.dET12*solid.cE24+solid.dET13*solid.cE34+solid.dET14*solid.cE44+solid.dET15*solid.cE45+solid.dET16*solid.cE46)*solid.dET24+(solid.dET11*solid.cE15+solid.dET12*solid.cE25+solid.dET13*solid.cE35+solid.dET14*solid.cE45+solid.dET15*solid.cE55+solid.dET16*solid.cE56)*solid.dET25+(solid.dET11*solid.cE16+solid.dET12*solid.cE26+solid.dET13*solid.cE36+solid.dET14*solid.cE46+solid.dET15*solid.cE56+solid.dET16*solid.cE66)*solid.dET26)/epsilon0_const | 1 | Relative permittivity at constant stress, 12-component | Domain 5 | * operation |
| solid.epsilonrT13 | solid.epsilonrS13+((solid.dET11*solid.cE11+solid.dET12*solid.cE12+solid.dET13*solid.cE13+solid.dET14*solid.cE14+solid.dET15*solid.cE15+solid.dET16*solid.cE16)*solid.dET31+(solid.dET11*solid.cE12+solid.dET12*solid.cE22+solid.dET13*solid.cE23+solid.dET14*solid.cE24+solid.dET15*solid.cE25+solid.dET16*solid.cE26)*solid.dET32+(solid.dET11*solid.cE13+solid.dET12*solid.cE23+solid.dET13*solid.cE33+solid.dET14*solid.cE34+solid.dET15*solid.cE35+solid.dET16*solid.cE36)*solid.dET33+(solid.dET11*solid.cE14+solid.dET12*solid.cE24+solid.dET13*solid.cE34+solid.dET14*solid.cE44+solid.dET15*solid.cE45+solid.dET16*solid.cE46)*solid.dET34+(solid.dET11*solid.cE15+solid.dET12*solid.cE25+solid.dET13*solid.cE35+solid.dET14*solid.cE45+solid.dET15*solid.cE55+solid.dET16*solid.cE56)*solid.dET35+(solid.dET11*solid.cE16+solid.dET12*solid.cE26+solid.dET13*solid.cE36+solid.dET14*solid.cE46+solid.dET15*solid.cE56+solid.dET16*solid.cE66)*solid.dET36)/epsilon0_const | 1 | Relative permittivity at constant stress, 13-component | Domain 5 | * operation |
| solid.epsilonrT22 | solid.epsilonrS22+((solid.dET21*solid.cE11+solid.dET22*solid.cE12+solid.dET23*solid.cE13+solid.dET24*solid.cE14+solid.dET25*solid.cE15+solid.dET26*solid.cE16)*solid.dET21+(solid.dET21*solid.cE12+solid.dET22*solid.cE22+solid.dET23*solid.cE23+solid.dET24*solid.cE24+solid.dET25*solid.cE25+solid.dET26*solid.cE26)*solid.dET22+(solid.dET21*solid.cE13+solid.dET22*solid.cE23+solid.dET23*solid.cE33+solid.dET24*solid.cE34+solid.dET25*solid.cE35+solid.dET26*solid.cE36)*solid.dET23+(solid.dET21*solid.cE14+solid.dET22*solid.cE24+solid.dET23*solid.cE34+solid.dET24*solid.cE44+solid.dET25*solid.cE45+solid.dET26*solid.cE46)*solid.dET24+(solid.dET21*solid.cE15+solid.dET22*solid.cE25+solid.dET23*solid.cE35+solid.dET24*solid.cE45+solid.dET25*solid.cE55+solid.dET26*solid.cE56)*solid.dET25+(solid.dET21*solid.cE16+solid.dET22*solid.cE26+solid.dET23*solid.cE36+solid.dET24*solid.cE46+solid.dET25*solid.cE56+solid.dET26*solid.cE66)*solid.dET26)/epsilon0_const | 1 | Relative permittivity at constant stress, 22-component | Domain 5 | * operation |
| solid.epsilonrT23 | solid.epsilonrS23+((solid.dET21*solid.cE11+solid.dET22*solid.cE12+solid.dET23*solid.cE13+solid.dET24*solid.cE14+solid.dET25*solid.cE15+solid.dET26*solid.cE16)*solid.dET31+(solid.dET21*solid.cE12+solid.dET22*solid.cE22+solid.dET23*solid.cE23+solid.dET24*solid.cE24+solid.dET25*solid.cE25+solid.dET26*solid.cE26)*solid.dET32+(solid.dET21*solid.cE13+solid.dET22*solid.cE23+solid.dET23*solid.cE33+solid.dET24*solid.cE34+solid.dET25*solid.cE35+solid.dET26*solid.cE36)*solid.dET33+(solid.dET21*solid.cE14+solid.dET22*solid.cE24+solid.dET23*solid.cE34+solid.dET24*solid.cE44+solid.dET25*solid.cE45+solid.dET26*solid.cE46)*solid.dET34+(solid.dET21*solid.cE15+solid.dET22*solid.cE25+solid.dET23*solid.cE35+solid.dET24*solid.cE45+solid.dET25*solid.cE55+solid.dET26*solid.cE56)*solid.dET35+(solid.dET21*solid.cE16+solid.dET22*solid.cE26+solid.dET23*solid.cE36+solid.dET24*solid.cE46+solid.dET25*solid.cE56+solid.dET26*solid.cE66)*solid.dET36)/epsilon0_const | 1 | Relative permittivity at constant stress, 23-component | Domain 5 | * operation |
| solid.epsilonrT33 | solid.epsilonrS33+((solid.dET31*solid.cE11+solid.dET32*solid.cE12+solid.dET33*solid.cE13+solid.dET34*solid.cE14+solid.dET35*solid.cE15+solid.dET36*solid.cE16)*solid.dET31+(solid.dET31*solid.cE12+solid.dET32*solid.cE22+solid.dET33*solid.cE23+solid.dET34*solid.cE24+solid.dET35*solid.cE25+solid.dET36*solid.cE26)*solid.dET32+(solid.dET31*solid.cE13+solid.dET32*solid.cE23+solid.dET33*solid.cE33+solid.dET34*solid.cE34+solid.dET35*solid.cE35+solid.dET36*solid.cE36)*solid.dET33+(solid.dET31*solid.cE14+solid.dET32*solid.cE24+solid.dET33*solid.cE34+solid.dET34*solid.cE44+solid.dET35*solid.cE45+solid.dET36*solid.cE46)*solid.dET34+(solid.dET31*solid.cE15+solid.dET32*solid.cE25+solid.dET33*solid.cE35+solid.dET34*solid.cE45+solid.dET35*solid.cE55+solid.dET36*solid.cE56)*solid.dET35+(solid.dET31*solid.cE16+solid.dET32*solid.cE26+solid.dET33*solid.cE36+solid.dET34*solid.cE46+solid.dET35*solid.cE56+solid.dET36*solid.cE66)*solid.dET36)/epsilon0_const | 1 | Relative permittivity at constant stress, 33-component | Domain 5 | * operation |
| solid.epzeXX | (comp1_xz_sys.T11*solid.pzm1.epl11+comp1_xz_sys.T21*solid.pzm1.epl12+comp1_xz_sys.T31*solid.pzm1.epl13)*comp1_xz_sys.T11+(comp1_xz_sys.T11*solid.pzm1.epl12+comp1_xz_sys.T21*solid.pzm1.epl22+comp1_xz_sys.T31*solid.pzm1.epl23)*comp1_xz_sys.T21+(comp1_xz_sys.T11*solid.pzm1.epl13+comp1_xz_sys.T21*solid.pzm1.epl23+comp1_xz_sys.T31*solid.pzm1.epl33)*comp1_xz_sys.T31 | 1 | Piezoelectric strain tensor, XX-component | Domain 5 |  |
| solid.epzeXY | (comp1_xz_sys.T11*solid.pzm1.epl11+comp1_xz_sys.T21*solid.pzm1.epl12+comp1_xz_sys.T31*solid.pzm1.epl13)*comp1_xz_sys.T12+(comp1_xz_sys.T11*solid.pzm1.epl12+comp1_xz_sys.T21*solid.pzm1.epl22+comp1_xz_sys.T31*solid.pzm1.epl23)*comp1_xz_sys.T22+(comp1_xz_sys.T11*solid.pzm1.epl13+comp1_xz_sys.T21*solid.pzm1.epl23+comp1_xz_sys.T31*solid.pzm1.epl33)*comp1_xz_sys.T32 | 1 | Piezoelectric strain tensor, XY-component | Domain 5 |  |
| solid.epzeXZ | (comp1_xz_sys.T11*solid.pzm1.epl11+comp1_xz_sys.T21*solid.pzm1.epl12+comp1_xz_sys.T31*solid.pzm1.epl13)*comp1_xz_sys.T13+(comp1_xz_sys.T11*solid.pzm1.epl12+comp1_xz_sys.T21*solid.pzm1.epl22+comp1_xz_sys.T31*solid.pzm1.epl23)*comp1_xz_sys.T23+(comp1_xz_sys.T11*solid.pzm1.epl13+comp1_xz_sys.T21*solid.pzm1.epl23+comp1_xz_sys.T31*solid.pzm1.epl33)*comp1_xz_sys.T33 | 1 | Piezoelectric strain tensor, XZ-component | Domain 5 |  |
| solid.epzeYY | (comp1_xz_sys.T12*solid.pzm1.epl11+comp1_xz_sys.T22*solid.pzm1.epl12+comp1_xz_sys.T32*solid.pzm1.epl13)*comp1_xz_sys.T12+(comp1_xz_sys.T12*solid.pzm1.epl12+comp1_xz_sys.T22*solid.pzm1.epl22+comp1_xz_sys.T32*solid.pzm1.epl23)*comp1_xz_sys.T22+(comp1_xz_sys.T12*solid.pzm1.epl13+comp1_xz_sys.T22*solid.pzm1.epl23+comp1_xz_sys.T32*solid.pzm1.epl33)*comp1_xz_sys.T32 | 1 | Piezoelectric strain tensor, YY-component | Domain 5 |  |
| solid.epzeYZ | (comp1_xz_sys.T12*solid.pzm1.epl11+comp1_xz_sys.T22*solid.pzm1.epl12+comp1_xz_sys.T32*solid.pzm1.epl13)*comp1_xz_sys.T13+(comp1_xz_sys.T12*solid.pzm1.epl12+comp1_xz_sys.T22*solid.pzm1.epl22+comp1_xz_sys.T32*solid.pzm1.epl23)*comp1_xz_sys.T23+(comp1_xz_sys.T12*solid.pzm1.epl13+comp1_xz_sys.T22*solid.pzm1.epl23+comp1_xz_sys.T32*solid.pzm1.epl33)*comp1_xz_sys.T33 | 1 | Piezoelectric strain tensor, YZ-component | Domain 5 |  |
| solid.epzeZZ | (comp1_xz_sys.T13*solid.pzm1.epl11+comp1_xz_sys.T23*solid.pzm1.epl12+comp1_xz_sys.T33*solid.pzm1.epl13)*comp1_xz_sys.T13+(comp1_xz_sys.T13*solid.pzm1.epl12+comp1_xz_sys.T23*solid.pzm1.epl22+comp1_xz_sys.T33*solid.pzm1.epl23)*comp1_xz_sys.T23+(comp1_xz_sys.T13*solid.pzm1.epl13+comp1_xz_sys.T23*solid.pzm1.epl23+comp1_xz_sys.T33*solid.pzm1.epl33)*comp1_xz_sys.T33 | 1 | Piezoelectric strain tensor, ZZ-component | Domain 5 |  |
| solid.Ldx | solid.gradUxX*solid.iomega | 1/s | Rate of strain tensor, x-component | Domain 5 |  |
| solid.Ldxy | 0.5*solid.iomega*(solid.gradUxY+solid.gradUyX) | 1/s | Rate of strain tensor, xy-component | Domain 5 |  |
| solid.Ldxz | 0.5*solid.iomega*(solid.gradUxZ+solid.gradUzX) | 1/s | Rate of strain tensor, xz-component | Domain 5 |  |
| solid.Ldy | solid.gradUyY*solid.iomega | 1/s | Rate of strain tensor, y-component | Domain 5 |  |
| solid.Ldyz | 0.5*solid.iomega*(solid.gradUyZ+solid.gradUzY) | 1/s | Rate of strain tensor, yz-component | Domain 5 |  |
| solid.Ldz | solid.gradUzZ*solid.iomega | 1/s | Rate of strain tensor, z-component | Domain 5 |  |
| solid.Lwx | 0 | 1/s | Spin tensor, x-component | Domain 5 |  |
| solid.Lwxy | 0.5*solid.iomega*(solid.gradUxY-solid.gradUyX) | 1/s | Spin tensor, xy-component | Domain 5 |  |
| solid.Lwxz | 0.5*solid.iomega*(solid.gradUxZ-solid.gradUzX) | 1/s | Spin tensor, xz-component | Domain 5 |  |
| solid.Lwy | 0 | 1/s | Spin tensor, y-component | Domain 5 |  |
| solid.Lwyz | 0.5*solid.iomega*(solid.gradUyZ-solid.gradUzY) | 1/s | Spin tensor, yz-component | Domain 5 |  |
| solid.Lwz | 0 | 1/s | Spin tensor, z-component | Domain 5 |  |
| solid.sp1Gp | solid.gpeval(solid.sp1) | N/m² | First principal stress | Domain 5 |  |
| solid.sp2Gp | solid.gpeval(solid.sp2) | N/m² | Second principal stress | Domain 5 |  |
| solid.sp3Gp | solid.gpeval(solid.sp3) | N/m² | Third principal stress | Domain 5 |  |
| solid.RFx | reacf(u) | N | Reaction force, x-component | Domain 5 |  |
| solid.RFy | reacf(v) | N | Reaction force, y-component | Domain 5 |  |
| solid.RFz | 0 | N | Reaction force, z-component | Domain 5 |  |
| solid.RMx | solid.RFz*(y-solid.refpnty)+solid.RFy*solid.refpntz | N·m | Reaction moment, x-component | Domain 5 |  |
| solid.RMy | -solid.RFz*(x-solid.refpntx)-solid.RFx*solid.refpntz | N·m | Reaction moment, y-component | Domain 5 |  |
| solid.RMz | solid.RFy*(x-solid.refpntx)-solid.RFx*(y-solid.refpnty) | N·m | Reaction moment, z-component | Domain 5 |  |
| solid.Qh_tot | solid.pzm1.int11(solid.Qh*solid.d) | W | Total power dissipation | Global | + operation |
| solid.IX | 0.5*real(-solid.SX*conj(solid.u_tX)-solid.SXY*conj(solid.u_tY)-solid.SXZ*conj(solid.u_tZ)) | W/m² | Mechanical energy flux, X-component | Domain 5 |  |
| solid.IY | 0.5*real(-solid.SXY*conj(solid.u_tX)-solid.SY*conj(solid.u_tY)-solid.SYZ*conj(solid.u_tZ)) | W/m² | Mechanical energy flux, Y-component | Domain 5 |  |
| solid.IZ | 0.5*real(-solid.SXZ*conj(solid.u_tX)-solid.SYZ*conj(solid.u_tY)-solid.SZ*conj(solid.u_tZ)) | W/m² | Mechanical energy flux, Z-component | Domain 5 |  |
| solid.IcomplexX | -solid.SX*conj(solid.u_tX)-solid.SXY*conj(solid.u_tY)-solid.SXZ*conj(solid.u_tZ) | W/m² | Complex mechanical energy flux, X-component | Domain 5 |  |
| solid.IcomplexY | -solid.SXY*conj(solid.u_tX)-solid.SY*conj(solid.u_tY)-solid.SYZ*conj(solid.u_tZ) | W/m² | Complex mechanical energy flux, Y-component | Domain 5 |  |
| solid.IcomplexZ | -solid.SXZ*conj(solid.u_tX)-solid.SYZ*conj(solid.u_tY)-solid.SZ*conj(solid.u_tZ) | W/m² | Complex mechanical energy flux, Z-component | Domain 5 |  |
| solid.nI | nX*solid.IX+nY*solid.IY | W/m² | Outward mechanical energy flux | Boundaries 13, 15, 21 | Meta |
| solid.nIcomplex | nX*solid.IcomplexX+nY*solid.IcomplexY | W/m² | Outward complex mechanical energy flux | Boundaries 13, 15, 21 | Meta |
| solid.pzm1.integrand | 1 |  | Intermediate variable | Domain 5 | Meta |
| solid.pzm1.gsv | solid.d | m | Geometry scale factor (volume) | Domain 5 |  |
| solid.pzm1.epl11 | solid.dET11*(solid.sysT11*solid.EpzeX+solid.sysT12*solid.EpzeY+solid.sysT13*solid.EpzeZ)+solid.dET21*(solid.sysT21*solid.EpzeX+solid.sysT22*solid.EpzeY+solid.sysT23*solid.EpzeZ)+solid.dET31*(solid.sysT31*solid.EpzeX+solid.sysT32*solid.EpzeY+solid.sysT33*solid.EpzeZ) | 1 | Piezoelectric strain tensor, local coordinate system, 11-component | Domain 5 |  |
| solid.pzm1.epl12 | 0.5*(solid.dET16*(solid.sysT11*solid.EpzeX+solid.sysT12*solid.EpzeY+solid.sysT13*solid.EpzeZ)+solid.dET26*(solid.sysT21*solid.EpzeX+solid.sysT22*solid.EpzeY+solid.sysT23*solid.EpzeZ)+solid.dET36*(solid.sysT31*solid.EpzeX+solid.sysT32*solid.EpzeY+solid.sysT33*solid.EpzeZ)) | 1 | Piezoelectric strain tensor, local coordinate system, 12-component | Domain 5 |  |
| solid.pzm1.epl13 | 0.5*(solid.dET15*(solid.sysT11*solid.EpzeX+solid.sysT12*solid.EpzeY+solid.sysT13*solid.EpzeZ)+solid.dET25*(solid.sysT21*solid.EpzeX+solid.sysT22*solid.EpzeY+solid.sysT23*solid.EpzeZ)+solid.dET35*(solid.sysT31*solid.EpzeX+solid.sysT32*solid.EpzeY+solid.sysT33*solid.EpzeZ)) | 1 | Piezoelectric strain tensor, local coordinate system, 13-component | Domain 5 |  |
| solid.pzm1.epl22 | solid.dET12*(solid.sysT11*solid.EpzeX+solid.sysT12*solid.EpzeY+solid.sysT13*solid.EpzeZ)+solid.dET22*(solid.sysT21*solid.EpzeX+solid.sysT22*solid.EpzeY+solid.sysT23*solid.EpzeZ)+solid.dET32*(solid.sysT31*solid.EpzeX+solid.sysT32*solid.EpzeY+solid.sysT33*solid.EpzeZ) | 1 | Piezoelectric strain tensor, local coordinate system, 22-component | Domain 5 |  |
| solid.pzm1.epl23 | 0.5*(solid.dET14*(solid.sysT11*solid.EpzeX+solid.sysT12*solid.EpzeY+solid.sysT13*solid.EpzeZ)+solid.dET24*(solid.sysT21*solid.EpzeX+solid.sysT22*solid.EpzeY+solid.sysT23*solid.EpzeZ)+solid.dET34*(solid.sysT31*solid.EpzeX+solid.sysT32*solid.EpzeY+solid.sysT33*solid.EpzeZ)) | 1 | Piezoelectric strain tensor, local coordinate system, 23-component | Domain 5 |  |
| solid.pzm1.epl33 | solid.dET13*(solid.sysT11*solid.EpzeX+solid.sysT12*solid.EpzeY+solid.sysT13*solid.EpzeZ)+solid.dET23*(solid.sysT21*solid.EpzeX+solid.sysT22*solid.EpzeY+solid.sysT23*solid.EpzeZ)+solid.dET33*(solid.sysT31*solid.EpzeX+solid.sysT32*solid.EpzeY+solid.sysT33*solid.EpzeZ) | 1 | Piezoelectric strain tensor, local coordinate system, 33-component | Domain 5 |  |
| solid.pzm1.bM11 | comp1_xz_sys.T11^2 | 1 | Bond transformation matrix, 11-component | Domain 5 |  |
| solid.pzm1.bM21 | comp1_xz_sys.T12^2 | 1 | Bond transformation matrix, 21-component | Domain 5 |  |
| solid.pzm1.bM31 | comp1_xz_sys.T13^2 | 1 | Bond transformation matrix, 31-component | Domain 5 |  |
| solid.pzm1.bM41 | comp1_xz_sys.T12*comp1_xz_sys.T13 | 1 | Bond transformation matrix, 41-component | Domain 5 |  |
| solid.pzm1.bM51 | comp1_xz_sys.T13*comp1_xz_sys.T11 | 1 | Bond transformation matrix, 51-component | Domain 5 |  |
| solid.pzm1.bM61 | comp1_xz_sys.T11*comp1_xz_sys.T12 | 1 | Bond transformation matrix, 61-component | Domain 5 |  |
| solid.pzm1.bM12 | comp1_xz_sys.T21^2 | 1 | Bond transformation matrix, 12-component | Domain 5 |  |
| solid.pzm1.bM22 | comp1_xz_sys.T22^2 | 1 | Bond transformation matrix, 22-component | Domain 5 |  |
| solid.pzm1.bM32 | comp1_xz_sys.T23^2 | 1 | Bond transformation matrix, 32-component | Domain 5 |  |
| solid.pzm1.bM42 | comp1_xz_sys.T22*comp1_xz_sys.T23 | 1 | Bond transformation matrix, 42-component | Domain 5 |  |
| solid.pzm1.bM52 | comp1_xz_sys.T23*comp1_xz_sys.T21 | 1 | Bond transformation matrix, 52-component | Domain 5 |  |
| solid.pzm1.bM62 | comp1_xz_sys.T21*comp1_xz_sys.T22 | 1 | Bond transformation matrix, 62-component | Domain 5 |  |
| solid.pzm1.bM13 | comp1_xz_sys.T31^2 | 1 | Bond transformation matrix, 13-component | Domain 5 |  |
| solid.pzm1.bM23 | comp1_xz_sys.T32^2 | 1 | Bond transformation matrix, 23-component | Domain 5 |  |
| solid.pzm1.bM33 | comp1_xz_sys.T33^2 | 1 | Bond transformation matrix, 33-component | Domain 5 |  |
| solid.pzm1.bM43 | comp1_xz_sys.T32*comp1_xz_sys.T33 | 1 | Bond transformation matrix, 43-component | Domain 5 |  |
| solid.pzm1.bM53 | comp1_xz_sys.T33*comp1_xz_sys.T31 | 1 | Bond transformation matrix, 53-component | Domain 5 |  |
| solid.pzm1.bM63 | comp1_xz_sys.T31*comp1_xz_sys.T32 | 1 | Bond transformation matrix, 63-component | Domain 5 |  |
| solid.pzm1.bM14 | 2*comp1_xz_sys.T21*comp1_xz_sys.T31 | 1 | Bond transformation matrix, 14-component | Domain 5 |  |
| solid.pzm1.bM24 | 2*comp1_xz_sys.T22*comp1_xz_sys.T32 | 1 | Bond transformation matrix, 24-component | Domain 5 |  |
| solid.pzm1.bM34 | 2*comp1_xz_sys.T23*comp1_xz_sys.T33 | 1 | Bond transformation matrix, 34-component | Domain 5 |  |
| solid.pzm1.bM44 | comp1_xz_sys.T22*comp1_xz_sys.T33+comp1_xz_sys.T23*comp1_xz_sys.T32 | 1 | Bond transformation matrix, 44-component | Domain 5 |  |
| solid.pzm1.bM54 | comp1_xz_sys.T23*comp1_xz_sys.T31+comp1_xz_sys.T21*comp1_xz_sys.T33 | 1 | Bond transformation matrix, 54-component | Domain 5 |  |
| solid.pzm1.bM64 | comp1_xz_sys.T21*comp1_xz_sys.T32+comp1_xz_sys.T22*comp1_xz_sys.T31 | 1 | Bond transformation matrix, 64-component | Domain 5 |  |
| solid.pzm1.bM15 | 2*comp1_xz_sys.T31*comp1_xz_sys.T11 | 1 | Bond transformation matrix, 15-component | Domain 5 |  |
| solid.pzm1.bM25 | 2*comp1_xz_sys.T32*comp1_xz_sys.T12 | 1 | Bond transformation matrix, 25-component | Domain 5 |  |
| solid.pzm1.bM35 | 2*comp1_xz_sys.T33*comp1_xz_sys.T13 | 1 | Bond transformation matrix, 35-component | Domain 5 |  |
| solid.pzm1.bM45 | comp1_xz_sys.T32*comp1_xz_sys.T13+comp1_xz_sys.T33*comp1_xz_sys.T12 | 1 | Bond transformation matrix, 45-component | Domain 5 |  |
| solid.pzm1.bM55 | comp1_xz_sys.T33*comp1_xz_sys.T11+comp1_xz_sys.T31*comp1_xz_sys.T13 | 1 | Bond transformation matrix, 55-component | Domain 5 |  |
| solid.pzm1.bM65 | comp1_xz_sys.T31*comp1_xz_sys.T12+comp1_xz_sys.T32*comp1_xz_sys.T11 | 1 | Bond transformation matrix, 65-component | Domain 5 |  |
| solid.pzm1.bM16 | 2*comp1_xz_sys.T11*comp1_xz_sys.T21 | 1 | Bond transformation matrix, 16-component | Domain 5 |  |
| solid.pzm1.bM26 | 2*comp1_xz_sys.T12*comp1_xz_sys.T22 | 1 | Bond transformation matrix, 26-component | Domain 5 |  |
| solid.pzm1.bM36 | 2*comp1_xz_sys.T13*comp1_xz_sys.T23 | 1 | Bond transformation matrix, 36-component | Domain 5 |  |
| solid.pzm1.bM46 | comp1_xz_sys.T12*comp1_xz_sys.T23+comp1_xz_sys.T13*comp1_xz_sys.T22 | 1 | Bond transformation matrix, 46-component | Domain 5 |  |
| solid.pzm1.bM56 | comp1_xz_sys.T13*comp1_xz_sys.T21+comp1_xz_sys.T11*comp1_xz_sys.T23 | 1 | Bond transformation matrix, 56-component | Domain 5 |  |
| solid.pzm1.bM66 | comp1_xz_sys.T11*comp1_xz_sys.T22+comp1_xz_sys.T12*comp1_xz_sys.T21 | 1 | Bond transformation matrix, 66-component | Domain 5 |  |
| solid.pzm1.bN11 | comp1_xz_sys.T11^2 | 1 | Bond transformation matrix, 11-component | Domain 5 |  |
| solid.pzm1.bN21 | comp1_xz_sys.T12^2 | 1 | Bond transformation matrix, 21-component | Domain 5 |  |
| solid.pzm1.bN31 | comp1_xz_sys.T13^2 | 1 | Bond transformation matrix, 31-component | Domain 5 |  |
| solid.pzm1.bN41 | 2*comp1_xz_sys.T12*comp1_xz_sys.T13 | 1 | Bond transformation matrix, 41-component | Domain 5 |  |
| solid.pzm1.bN51 | 2*comp1_xz_sys.T13*comp1_xz_sys.T11 | 1 | Bond transformation matrix, 51-component | Domain 5 |  |
| solid.pzm1.bN61 | 2*comp1_xz_sys.T11*comp1_xz_sys.T12 | 1 | Bond transformation matrix, 61-component | Domain 5 |  |
| solid.pzm1.bN12 | comp1_xz_sys.T21^2 | 1 | Bond transformation matrix, 12-component | Domain 5 |  |
| solid.pzm1.bN22 | comp1_xz_sys.T22^2 | 1 | Bond transformation matrix, 22-component | Domain 5 |  |
| solid.pzm1.bN32 | comp1_xz_sys.T23^2 | 1 | Bond transformation matrix, 32-component | Domain 5 |  |
| solid.pzm1.bN42 | 2*comp1_xz_sys.T22*comp1_xz_sys.T23 | 1 | Bond transformation matrix, 42-component | Domain 5 |  |
| solid.pzm1.bN52 | 2*comp1_xz_sys.T23*comp1_xz_sys.T21 | 1 | Bond transformation matrix, 52-component | Domain 5 |  |
| solid.pzm1.bN62 | 2*comp1_xz_sys.T21*comp1_xz_sys.T22 | 1 | Bond transformation matrix, 62-component | Domain 5 |  |
| solid.pzm1.bN13 | comp1_xz_sys.T31^2 | 1 | Bond transformation matrix, 13-component | Domain 5 |  |
| solid.pzm1.bN23 | comp1_xz_sys.T32^2 | 1 | Bond transformation matrix, 23-component | Domain 5 |  |
| solid.pzm1.bN33 | comp1_xz_sys.T33^2 | 1 | Bond transformation matrix, 33-component | Domain 5 |  |
| solid.pzm1.bN43 | 2*comp1_xz_sys.T32*comp1_xz_sys.T33 | 1 | Bond transformation matrix, 43-component | Domain 5 |  |
| solid.pzm1.bN53 | 2*comp1_xz_sys.T33*comp1_xz_sys.T31 | 1 | Bond transformation matrix, 53-component | Domain 5 |  |
| solid.pzm1.bN63 | 2*comp1_xz_sys.T31*comp1_xz_sys.T32 | 1 | Bond transformation matrix, 63-component | Domain 5 |  |
| solid.pzm1.bN14 | comp1_xz_sys.T21*comp1_xz_sys.T31 | 1 | Bond transformation matrix, 14-component | Domain 5 |  |
| solid.pzm1.bN24 | comp1_xz_sys.T22*comp1_xz_sys.T32 | 1 | Bond transformation matrix, 24-component | Domain 5 |  |
| solid.pzm1.bN34 | comp1_xz_sys.T23*comp1_xz_sys.T33 | 1 | Bond transformation matrix, 34-component | Domain 5 |  |
| solid.pzm1.bN44 | comp1_xz_sys.T22*comp1_xz_sys.T33+comp1_xz_sys.T23*comp1_xz_sys.T32 | 1 | Bond transformation matrix, 44-component | Domain 5 |  |
| solid.pzm1.bN54 | comp1_xz_sys.T23*comp1_xz_sys.T31+comp1_xz_sys.T21*comp1_xz_sys.T33 | 1 | Bond transformation matrix, 54-component | Domain 5 |  |
| solid.pzm1.bN64 | comp1_xz_sys.T21*comp1_xz_sys.T32+comp1_xz_sys.T22*comp1_xz_sys.T31 | 1 | Bond transformation matrix, 64-component | Domain 5 |  |
| solid.pzm1.bN15 | comp1_xz_sys.T31*comp1_xz_sys.T11 | 1 | Bond transformation matrix, 15-component | Domain 5 |  |
| solid.pzm1.bN25 | comp1_xz_sys.T32*comp1_xz_sys.T12 | 1 | Bond transformation matrix, 25-component | Domain 5 |  |
| solid.pzm1.bN35 | comp1_xz_sys.T33*comp1_xz_sys.T13 | 1 | Bond transformation matrix, 35-component | Domain 5 |  |
| solid.pzm1.bN45 | comp1_xz_sys.T32*comp1_xz_sys.T13+comp1_xz_sys.T33*comp1_xz_sys.T12 | 1 | Bond transformation matrix, 45-component | Domain 5 |  |
| solid.pzm1.bN55 | comp1_xz_sys.T33*comp1_xz_sys.T11+comp1_xz_sys.T31*comp1_xz_sys.T13 | 1 | Bond transformation matrix, 55-component | Domain 5 |  |
| solid.pzm1.bN65 | comp1_xz_sys.T31*comp1_xz_sys.T12+comp1_xz_sys.T32*comp1_xz_sys.T11 | 1 | Bond transformation matrix, 65-component | Domain 5 |  |
| solid.pzm1.bN16 | comp1_xz_sys.T11*comp1_xz_sys.T21 | 1 | Bond transformation matrix, 16-component | Domain 5 |  |
| solid.pzm1.bN26 | comp1_xz_sys.T12*comp1_xz_sys.T22 | 1 | Bond transformation matrix, 26-component | Domain 5 |  |
| solid.pzm1.bN36 | comp1_xz_sys.T13*comp1_xz_sys.T23 | 1 | Bond transformation matrix, 36-component | Domain 5 |  |
| solid.pzm1.bN46 | comp1_xz_sys.T12*comp1_xz_sys.T23+comp1_xz_sys.T13*comp1_xz_sys.T22 | 1 | Bond transformation matrix, 46-component | Domain 5 |  |
| solid.pzm1.bN56 | comp1_xz_sys.T13*comp1_xz_sys.T21+comp1_xz_sys.T11*comp1_xz_sys.T23 | 1 | Bond transformation matrix, 56-component | Domain 5 |  |
| solid.pzm1.bN66 | comp1_xz_sys.T11*comp1_xz_sys.T22+comp1_xz_sys.T12*comp1_xz_sys.T21 | 1 | Bond transformation matrix, 66-component | Domain 5 |  |
| solid.pzm1.nI | nX*solid.IX+nY*solid.IY | W/m² | Outward mechanical energy flux | Boundaries 13–15, 21 | Meta |
| solid.pzm1.nIcomplex | nX*solid.IcomplexX+nY*solid.IcomplexY | W/m² | Outward complex mechanical energy flux | Boundaries 13–15, 21 | Meta |

#### Shape functions

| **Name** | **Shape function** | **Unit** | **Description** | **Shape frame** | **Selection** |
| --- | --- | --- | --- | --- | --- |
| u | Nodal serendipity (Quadratic) | m | Displacement field, X-component | Material | Domain 5 |
| v | Nodal serendipity (Quadratic) | m | Displacement field, Y-component | Material | Domain 5 |

#### Weak Expressions

| **Weak expression** | **Integration order** | **Integration frame** | **Selection** |
| --- | --- | --- | --- |
| (-solid.Sl11*test(solid.el11)-2*solid.Sl12*test(solid.el12)-2*solid.Sl13*test(solid.el13)-solid.Sl22*test(solid.el22)-2*solid.Sl23*test(solid.el23)-solid.Sl33*test(solid.el33))*solid.d | 4 | Material | Domain 5 |
| -solid.rho*solid.iomega^2*(u*test(u)+v*test(v))*solid.d | 4 | Material | Domain 5 |

### Piezoelectric Material bottom


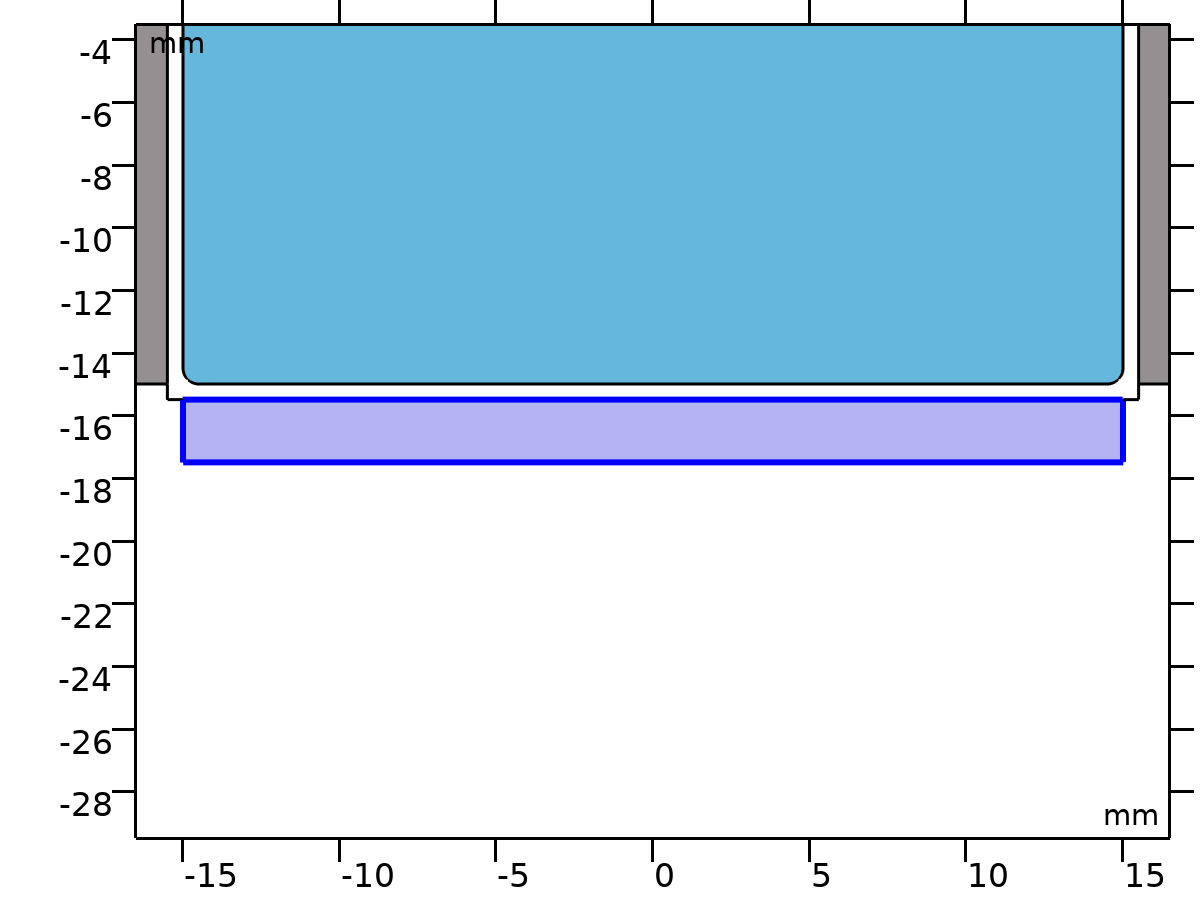


Piezoelectric Material bottom

Selection

| Geometric entity level | Domain |
| --- | --- |
| Selection | Geometry geom1: Dimension 2: Domain 3 |

Equations


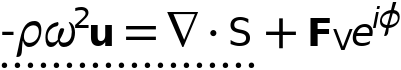


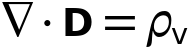


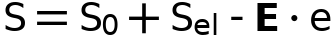


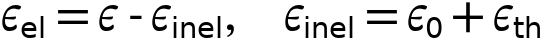


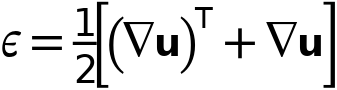


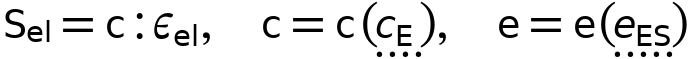


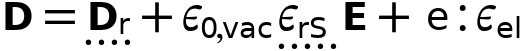


#### Piezoelectric Material Properties

Settings

| **Description** | **Value** | **Unit** |
| --- | --- | --- |
| Constitutive relation | Stress - charge form |  |
| Use multiplicative formulation | Off |  |
| Elasticity matrix, Voigt notation | From material |  |
| Coupling matrix, Voigt notation | From material |  |
| Relative permittivity | From material |  |
| Use mixed formulation | None |  |
| Remanent electric displacement, x1-component | 0 | C/m² |
| Remanent electric displacement, x2-component | 0 | C/m² |
| Remanent electric displacement, x3-component | 0 | C/m² |
| Density | From material |  |

#### Geometric Nonlinearity

Settings

| **Description** | **Value** |
| --- | --- |
| Formulation | From study step |
| Strain decomposition | Automatic |

#### Energy Dissipation

Settings

| **Description** | **Value** |
| --- | --- |
| Calculate dissipated energy | Off |

#### Quadrature Settings

Settings

| **Description** | **Value** |
| --- | --- |
| Reduced integration | Off |

#### Coordinate System Selection

Settings

| **Description** | **Value** |
| --- | --- |
| Coordinate system | Coordinate system for bottom piezo (sys3) |

#### Model Input

Settings

| **Description** | **Value** |
| --- | --- |
| Volume reference temperature | Common model input |

Properties from material

| **Property** | **Material** | **Property group** |
| --- | --- | --- |
| Elasticity matrix, Voigt notation | Lead Zirconate Titanate (PZT-5H) | Stress-charge form |
| Coupling matrix, Voigt notation | Lead Zirconate Titanate (PZT-5H) | Stress-charge form |
| Relative permittivity | Lead Zirconate Titanate (PZT-5H) | Stress-charge form |
| Density | Lead Zirconate Titanate (PZT-5H) | Basic |
| Density | Polystyrene (PS) [solid] | Basic |

#### Variables

| **Name** | **Expression** | **Unit** | **Description** | **Selection** | **Details** |
| --- | --- | --- | --- | --- | --- |
| solid.DrX | sys3.T11*solid.Dr1+sys3.T21*solid.Dr2+sys3.T31*solid.Dr3 | C/m² | Remanent electric displacement, X-component | Domain 3 |  |
| solid.DrY | sys3.T12*solid.Dr1+sys3.T22*solid.Dr2+sys3.T32*solid.Dr3 | C/m² | Remanent electric displacement, Y-component | Domain 3 |  |
| solid.DrZ | sys3.T13*solid.Dr1+sys3.T23*solid.Dr2+sys3.T33*solid.Dr3 | C/m² | Remanent electric displacement, Z-component | Domain 3 |  |
| solid.epsilonrXX | (sys3.T11*solid.epsilonrS11+sys3.T21*solid.epsilonrS12+sys3.T31*solid.epsilonrS13)*sys3.T11+(sys3.T11*solid.epsilonrS12+sys3.T21*solid.epsilonrS22+sys3.T31*solid.epsilonrS23)*sys3.T21+(sys3.T11*solid.epsilonrS13+sys3.T21*solid.epsilonrS23+sys3.T31*solid.epsilonrS33)*sys3.T31 | 1 | Relative permittivity, piezoelectric solid, XX-component | Domain 3 |  |
| solid.epsilonrYX | (sys3.T12*solid.epsilonrS11+sys3.T22*solid.epsilonrS12+sys3.T32*solid.epsilonrS13)*sys3.T11+(sys3.T12*solid.epsilonrS12+sys3.T22*solid.epsilonrS22+sys3.T32*solid.epsilonrS23)*sys3.T21+(sys3.T12*solid.epsilonrS13+sys3.T22*solid.epsilonrS23+sys3.T32*solid.epsilonrS33)*sys3.T31 | 1 | Relative permittivity, piezoelectric solid, YX-component | Domain 3 |  |
| solid.epsilonrZX | (sys3.T13*solid.epsilonrS11+sys3.T23*solid.epsilonrS12+sys3.T33*solid.epsilonrS13)*sys3.T11+(sys3.T13*solid.epsilonrS12+sys3.T23*solid.epsilonrS22+sys3.T33*solid.epsilonrS23)*sys3.T21+(sys3.T13*solid.epsilonrS13+sys3.T23*solid.epsilonrS23+sys3.T33*solid.epsilonrS33)*sys3.T31 | 1 | Relative permittivity, piezoelectric solid, ZX-component | Domain 3 |  |
| solid.epsilonrXY | (sys3.T11*solid.epsilonrS11+sys3.T21*solid.epsilonrS12+sys3.T31*solid.epsilonrS13)*sys3.T12+(sys3.T11*solid.epsilonrS12+sys3.T21*solid.epsilonrS22+sys3.T31*solid.epsilonrS23)*sys3.T22+(sys3.T11*solid.epsilonrS13+sys3.T21*solid.epsilonrS23+sys3.T31*solid.epsilonrS33)*sys3.T32 | 1 | Relative permittivity, piezoelectric solid, XY-component | Domain 3 |  |
| solid.epsilonrYY | (sys3.T12*solid.epsilonrS11+sys3.T22*solid.epsilonrS12+sys3.T32*solid.epsilonrS13)*sys3.T12+(sys3.T12*solid.epsilonrS12+sys3.T22*solid.epsilonrS22+sys3.T32*solid.epsilonrS23)*sys3.T22+(sys3.T12*solid.epsilonrS13+sys3.T22*solid.epsilonrS23+sys3.T32*solid.epsilonrS33)*sys3.T32 | 1 | Relative permittivity, piezoelectric solid, YY-component | Domain 3 |  |
| solid.epsilonrZY | (sys3.T13*solid.epsilonrS11+sys3.T23*solid.epsilonrS12+sys3.T33*solid.epsilonrS13)*sys3.T12+(sys3.T13*solid.epsilonrS12+sys3.T23*solid.epsilonrS22+sys3.T33*solid.epsilonrS23)*sys3.T22+(sys3.T13*solid.epsilonrS13+sys3.T23*solid.epsilonrS23+sys3.T33*solid.epsilonrS33)*sys3.T32 | 1 | Relative permittivity, piezoelectric solid, ZY-component | Domain 3 |  |
| solid.epsilonrXZ | (sys3.T11*solid.epsilonrS11+sys3.T21*solid.epsilonrS12+sys3.T31*solid.epsilonrS13)*sys3.T13+(sys3.T11*solid.epsilonrS12+sys3.T21*solid.epsilonrS22+sys3.T31*solid.epsilonrS23)*sys3.T23+(sys3.T11*solid.epsilonrS13+sys3.T21*solid.epsilonrS23+sys3.T31*solid.epsilonrS33)*sys3.T33 | 1 | Relative permittivity, piezoelectric solid, XZ-component | Domain 3 |  |
| solid.epsilonrYZ | (sys3.T12*solid.epsilonrS11+sys3.T22*solid.epsilonrS12+sys3.T32*solid.epsilonrS13)*sys3.T13+(sys3.T12*solid.epsilonrS12+sys3.T22*solid.epsilonrS22+sys3.T32*solid.epsilonrS23)*sys3.T23+(sys3.T12*solid.epsilonrS13+sys3.T22*solid.epsilonrS23+sys3.T32*solid.epsilonrS33)*sys3.T33 | 1 | Relative permittivity, piezoelectric solid, YZ-component | Domain 3 |  |
| solid.epsilonrZZ | (sys3.T13*solid.epsilonrS11+sys3.T23*solid.epsilonrS12+sys3.T33*solid.epsilonrS13)*sys3.T13+(sys3.T13*solid.epsilonrS12+sys3.T23*solid.epsilonrS22+sys3.T33*solid.epsilonrS23)*sys3.T23+(sys3.T13*solid.epsilonrS13+sys3.T23*solid.epsilonrS23+sys3.T33*solid.epsilonrS33)*sys3.T33 | 1 | Relative permittivity, piezoelectric solid, ZZ-component | Domain 3 |  |
| solid.PpzeX | sys3.T11*(solid.eES11*solid.eel11+solid.eES12*solid.eel22+solid.eES13*solid.eel33+2*solid.eES14*solid.eel23+2*solid.eES15*solid.eel13+2*solid.eES16*solid.eel12)+sys3.T21*(solid.eES21*solid.eel11+solid.eES22*solid.eel22+solid.eES23*solid.eel33+2*solid.eES24*solid.eel23+2*solid.eES25*solid.eel13+2*solid.eES26*solid.eel12)+sys3.T31*(solid.eES31*solid.eel11+solid.eES32*solid.eel22+solid.eES33*solid.eel33+2*solid.eES34*solid.eel23+2*solid.eES35*solid.eel13+2*solid.eES36*solid.eel12) | C/m² | Piezoelectric polarization, X-component | Domain 3 | + operation |
| solid.PpzeY | sys3.T12*(solid.eES11*solid.eel11+solid.eES12*solid.eel22+solid.eES13*solid.eel33+2*solid.eES14*solid.eel23+2*solid.eES15*solid.eel13+2*solid.eES16*solid.eel12)+sys3.T22*(solid.eES21*solid.eel11+solid.eES22*solid.eel22+solid.eES23*solid.eel33+2*solid.eES24*solid.eel23+2*solid.eES25*solid.eel13+2*solid.eES26*solid.eel12)+sys3.T32*(solid.eES31*solid.eel11+solid.eES32*solid.eel22+solid.eES33*solid.eel33+2*solid.eES34*solid.eel23+2*solid.eES35*solid.eel13+2*solid.eES36*solid.eel12) | C/m² | Piezoelectric polarization, Y-component | Domain 3 | + operation |
| solid.PpzeZ | sys3.T13*(solid.eES11*solid.eel11+solid.eES12*solid.eel22+solid.eES13*solid.eel33+2*solid.eES14*solid.eel23+2*solid.eES15*solid.eel13+2*solid.eES16*solid.eel12)+sys3.T23*(solid.eES21*solid.eel11+solid.eES22*solid.eel22+solid.eES23*solid.eel33+2*solid.eES24*solid.eel23+2*solid.eES25*solid.eel13+2*solid.eES26*solid.eel12)+sys3.T33*(solid.eES31*solid.eel11+solid.eES32*solid.eel22+solid.eES33*solid.eel33+2*solid.eES34*solid.eel23+2*solid.eES35*solid.eel13+2*solid.eES36*solid.eel12) | C/m² | Piezoelectric polarization, Z-component | Domain 3 | + operation |
| solid.DpzeX | 0 | C/m² | Electric displacement field, X-component | Domain 3 | + operation |
| solid.DpzeY | 0 | C/m² | Electric displacement field, Y-component | Domain 3 | + operation |
| solid.DpzeZ | 0 | C/m² | Electric displacement field, Z-component | Domain 3 | + operation |
| solid.EpzeX | 0 | V/m | Electric field, X-component | Domain 3 | + operation |
| solid.EpzeY | 0 | V/m | Electric field, Y-component | Domain 3 | + operation |
| solid.EpzeZ | 0 | V/m | Electric field, Z-component | Domain 3 | + operation |
| solid.disp | sqrteps(real(u)^2+real(v)^2) | m | Displacement magnitude | Domain 3 |  |
| solid.disp_rms | sqrt(0.5*(realdot(u,u)+realdot(v,v))) | m | Displacement, RMS | Domain 3 |  |
| solid.curlUX | 0 | 1 | Curl of displacement, X-component | Domain 3 |  |
| solid.curlUY | 0 | 1 | Curl of displacement, Y-component | Domain 3 |  |
| solid.curlUZ | solid.gradUyX-solid.gradUxY | 1 | Curl of displacement, Z-component | Domain 3 |  |
| solid.gradUxX | uX | 1 | Displacement gradient, xX-component | Domain 3 |  |
| solid.gradUyX | vX | 1 | Displacement gradient, yX-component | Domain 3 |  |
| solid.gradUzX | 0 | 1 | Displacement gradient, zX-component | Domain 3 |  |
| solid.gradUxY | uY | 1 | Displacement gradient, xY-component | Domain 3 |  |
| solid.gradUyY | vY | 1 | Displacement gradient, yY-component | Domain 3 |  |
| solid.gradUzY | 0 | 1 | Displacement gradient, zY-component | Domain 3 |  |
| solid.gradUxZ | 0 | 1 | Displacement gradient, xZ-component | Domain 3 |  |
| solid.gradUyZ | 0 | 1 | Displacement gradient, yZ-component | Domain 3 |  |
| solid.gradUzZ | 0 | 1 | Displacement gradient, zZ-component | Domain 3 |  |
| solid.FdxX | 1+solid.gradUxX | 1 | Deformation gradient, xX-component | Domain 3 |  |
| solid.FdyX | solid.gradUyX | 1 | Deformation gradient, yX-component | Domain 3 |  |
| solid.FdzX | solid.gradUzX | 1 | Deformation gradient, zX-component | Domain 3 |  |
| solid.FdxY | solid.gradUxY | 1 | Deformation gradient, xY-component | Domain 3 |  |
| solid.FdyY | 1+solid.gradUyY | 1 | Deformation gradient, yY-component | Domain 3 |  |
| solid.FdzY | solid.gradUzY | 1 | Deformation gradient, zY-component | Domain 3 |  |
| solid.FdxZ | solid.gradUxZ | 1 | Deformation gradient, xZ-component | Domain 3 |  |
| solid.FdyZ | solid.gradUyZ | 1 | Deformation gradient, yZ-component | Domain 3 |  |
| solid.FdzZ | 1+solid.gradUzZ | 1 | Deformation gradient, zZ-component | Domain 3 |  |
| solid.FdiXx | (solid.FdyY*solid.FdzZ-solid.FdyZ*solid.FdzY)/solid.J | 1 | Deformation gradient inverse, Xx-component | Domain 3 |  |
| solid.FdiYx | (solid.FdyZ*solid.FdzX-solid.FdyX*solid.FdzZ)/solid.J | 1 | Deformation gradient inverse, Yx-component | Domain 3 |  |
| solid.FdiZx | (solid.FdyX*solid.FdzY-solid.FdyY*solid.FdzX)/solid.J | 1 | Deformation gradient inverse, Zx-component | Domain 3 |  |
| solid.FdiXy | (solid.FdxZ*solid.FdzY-solid.FdxY*solid.FdzZ)/solid.J | 1 | Deformation gradient inverse, Xy-component | Domain 3 |  |
| solid.FdiYy | (solid.FdxX*solid.FdzZ-solid.FdxZ*solid.FdzX)/solid.J | 1 | Deformation gradient inverse, Yy-component | Domain 3 |  |
| solid.FdiZy | (solid.FdxY*solid.FdzX-solid.FdxX*solid.FdzY)/solid.J | 1 | Deformation gradient inverse, Zy-component | Domain 3 |  |
| solid.FdiXz | (solid.FdxY*solid.FdyZ-solid.FdxZ*solid.FdyY)/solid.J | 1 | Deformation gradient inverse, Xz-component | Domain 3 |  |
| solid.FdiYz | (solid.FdxZ*solid.FdyX-solid.FdxX*solid.FdyZ)/solid.J | 1 | Deformation gradient inverse, Yz-component | Domain 3 |  |
| solid.FdiZz | (solid.FdxX*solid.FdyY-solid.FdxY*solid.FdyX)/solid.J | 1 | Deformation gradient inverse, Zz-component | Domain 3 |  |
| solid.J | solid.FdxX*solid.FdyY*solid.FdzZ+solid.FdxY*solid.FdyZ*solid.FdzX+solid.FdxZ*solid.FdyX*solid.FdzY-solid.FdxX*solid.FdyZ*solid.FdzY-solid.FdxY*solid.FdyX*solid.FdzZ-solid.FdxZ*solid.FdyY*solid.FdzX | 1 | Volume ratio | Domain 3 |  |
| solid.Fdlx1 | solid.FdxX*solid.sysT11+solid.FdxY*solid.sysT12+solid.FdxZ*solid.sysT13 | 1 | Deformation gradient, local, x1-component | Domain 3 |  |
| solid.Fdly1 | solid.FdyX*solid.sysT11+solid.FdyY*solid.sysT12+solid.FdyZ*solid.sysT13 | 1 | Deformation gradient, local, y1-component | Domain 3 |  |
| solid.Fdlz1 | solid.FdzX*solid.sysT11+solid.FdzY*solid.sysT12+solid.FdzZ*solid.sysT13 | 1 | Deformation gradient, local, z1-component | Domain 3 |  |
| solid.Fdlx2 | solid.FdxX*solid.sysT21+solid.FdxY*solid.sysT22+solid.FdxZ*solid.sysT23 | 1 | Deformation gradient, local, x2-component | Domain 3 |  |
| solid.Fdly2 | solid.FdyX*solid.sysT21+solid.FdyY*solid.sysT22+solid.FdyZ*solid.sysT23 | 1 | Deformation gradient, local, y2-component | Domain 3 |  |
| solid.Fdlz2 | solid.FdzX*solid.sysT21+solid.FdzY*solid.sysT22+solid.FdzZ*solid.sysT23 | 1 | Deformation gradient, local, z2-component | Domain 3 |  |
| solid.Fdlx3 | solid.FdxX*solid.sysT31+solid.FdxY*solid.sysT32+solid.FdxZ*solid.sysT33 | 1 | Deformation gradient, local, x3-component | Domain 3 |  |
| solid.Fdly3 | solid.FdyX*solid.sysT31+solid.FdyY*solid.sysT32+solid.FdyZ*solid.sysT33 | 1 | Deformation gradient, local, y3-component | Domain 3 |  |
| solid.Fdlz3 | solid.FdzX*solid.sysT31+solid.FdzY*solid.sysT32+solid.FdzZ*solid.sysT33 | 1 | Deformation gradient, local, z3-component | Domain 3 |  |
| solid.Fdil1x | (solid.Fdly2*solid.Fdlz3-solid.Fdly3*solid.Fdlz2)/solid.J | 1 | Deformation gradient inverse, local coordinate system, 1x-component | Domain 3 |  |
| solid.Fdil2x | (solid.Fdly3*solid.Fdlz1-solid.Fdly1*solid.Fdlz3)/solid.J | 1 | Deformation gradient inverse, local coordinate system, 2x-component | Domain 3 |  |
| solid.Fdil3x | (solid.Fdly1*solid.Fdlz2-solid.Fdly2*solid.Fdlz1)/solid.J | 1 | Deformation gradient inverse, local coordinate system, 3x-component | Domain 3 |  |
| solid.Fdil1y | (solid.Fdlx3*solid.Fdlz2-solid.Fdlx2*solid.Fdlz3)/solid.J | 1 | Deformation gradient inverse, local coordinate system, 1y-component | Domain 3 |  |
| solid.Fdil2y | (solid.Fdlx1*solid.Fdlz3-solid.Fdlx3*solid.Fdlz1)/solid.J | 1 | Deformation gradient inverse, local coordinate system, 2y-component | Domain 3 |  |
| solid.Fdil3y | (solid.Fdlx2*solid.Fdlz1-solid.Fdlx1*solid.Fdlz2)/solid.J | 1 | Deformation gradient inverse, local coordinate system, 3y-component | Domain 3 |  |
| solid.Fdil1z | (solid.Fdlx2*solid.Fdly3-solid.Fdlx3*solid.Fdly2)/solid.J | 1 | Deformation gradient inverse, local coordinate system, 1z-component | Domain 3 |  |
| solid.Fdil2z | (solid.Fdlx3*solid.Fdly1-solid.Fdlx1*solid.Fdly3)/solid.J | 1 | Deformation gradient inverse, local coordinate system, 2z-component | Domain 3 |  |
| solid.Fdil3z | (solid.Fdlx1*solid.Fdly2-solid.Fdlx2*solid.Fdly1)/solid.J | 1 | Deformation gradient inverse, local coordinate system, 3z-component | Domain 3 |  |
| solid.Ji | 1/(solid.Fiil11*solid.Fiil22*solid.Fiil33+solid.Fiil12*solid.Fiil23*solid.Fiil31+solid.Fiil13*solid.Fiil21*solid.Fiil32-solid.Fiil11*solid.Fiil23*solid.Fiil32-solid.Fiil12*solid.Fiil21*solid.Fiil33-solid.Fiil13*solid.Fiil22*solid.Fiil31) | 1 | Inelastic volume ratio | Domain 3 |  |
| solid.Fil11 | (solid.Fiil22*solid.Fiil33-solid.Fiil23*solid.Fiil32)*solid.Ji | 1 | Inelastic deformation gradient, local coordinate system, 11-component | Domain 3 | Matrix multiplication |
| solid.Fil21 | (solid.Fiil23*solid.Fiil31-solid.Fiil21*solid.Fiil33)*solid.Ji | 1 | Inelastic deformation gradient, local coordinate system, 21-component | Domain 3 | Matrix multiplication |
| solid.Fil31 | (solid.Fiil21*solid.Fiil32-solid.Fiil22*solid.Fiil31)*solid.Ji | 1 | Inelastic deformation gradient, local coordinate system, 31-component | Domain 3 | Matrix multiplication |
| solid.Fil12 | (solid.Fiil13*solid.Fiil32-solid.Fiil12*solid.Fiil33)*solid.Ji | 1 | Inelastic deformation gradient, local coordinate system, 12-component | Domain 3 | Matrix multiplication |
| solid.Fil22 | (solid.Fiil11*solid.Fiil33-solid.Fiil13*solid.Fiil31)*solid.Ji | 1 | Inelastic deformation gradient, local coordinate system, 22-component | Domain 3 | Matrix multiplication |
| solid.Fil32 | (solid.Fiil12*solid.Fiil31-solid.Fiil11*solid.Fiil32)*solid.Ji | 1 | Inelastic deformation gradient, local coordinate system, 32-component | Domain 3 | Matrix multiplication |
| solid.Fil13 | (solid.Fiil12*solid.Fiil23-solid.Fiil13*solid.Fiil22)*solid.Ji | 1 | Inelastic deformation gradient, local coordinate system, 13-component | Domain 3 | Matrix multiplication |
| solid.Fil23 | (solid.Fiil13*solid.Fiil21-solid.Fiil11*solid.Fiil23)*solid.Ji | 1 | Inelastic deformation gradient, local coordinate system, 23-component | Domain 3 | Matrix multiplication |
| solid.Fil33 | (solid.Fiil11*solid.Fiil22-solid.Fiil12*solid.Fiil21)*solid.Ji | 1 | Inelastic deformation gradient, local coordinate system, 33-component | Domain 3 | Matrix multiplication |
| solid.Fiil11 | 1 | 1 | Inelastic deformation gradient inverse, local coordinate system, 11-component | Domain 3 | Matrix multiplication |
| solid.Fiil21 | 0 | 1 | Inelastic deformation gradient inverse, local coordinate system, 21-component | Domain 3 | Matrix multiplication |
| solid.Fiil31 | 0 | 1 | Inelastic deformation gradient inverse, local coordinate system, 31-component | Domain 3 | Matrix multiplication |
| solid.Fiil12 | 0 | 1 | Inelastic deformation gradient inverse, local coordinate system, 12-component | Domain 3 | Matrix multiplication |
| solid.Fiil22 | 1 | 1 | Inelastic deformation gradient inverse, local coordinate system, 22-component | Domain 3 | Matrix multiplication |
| solid.Fiil32 | 0 | 1 | Inelastic deformation gradient inverse, local coordinate system, 32-component | Domain 3 | Matrix multiplication |
| solid.Fiil13 | 0 | 1 | Inelastic deformation gradient inverse, local coordinate system, 13-component | Domain 3 | Matrix multiplication |
| solid.Fiil23 | 0 | 1 | Inelastic deformation gradient inverse, local coordinate system, 23-component | Domain 3 | Matrix multiplication |
| solid.Fiil33 | 1 | 1 | Inelastic deformation gradient inverse, local coordinate system, 33-component | Domain 3 | Matrix multiplication |
| solid.Jel | sqrt(solid.Cel11*solid.Cel22*solid.Cel33+2*solid.Cel12*solid.Cel23*solid.Cel13-solid.Cel11*solid.Cel23^2-solid.Cel12^2*solid.Cel33-solid.Cel22*solid.Cel13^2) | 1 | Elastic volume ratio | Domain 3 |  |
| solid.CXX | solid.FdxX^2+solid.FdyX^2+solid.FdzX^2 | 1 | Cauchy–Green tensor, XX-component | Domain 3 |  |
| solid.CXY | solid.FdxX*solid.FdxY+solid.FdyX*solid.FdyY+solid.FdzX*solid.FdzY | 1 | Cauchy–Green tensor, XY-component | Domain 3 |  |
| solid.CXZ | solid.FdxX*solid.FdxZ+solid.FdyX*solid.FdyZ+solid.FdzX*solid.FdzZ | 1 | Cauchy–Green tensor, XZ-component | Domain 3 |  |
| solid.CYY | solid.FdxY^2+solid.FdyY^2+solid.FdzY^2 | 1 | Cauchy–Green tensor, YY-component | Domain 3 |  |
| solid.CYZ | solid.FdxY*solid.FdxZ+solid.FdyY*solid.FdyZ+solid.FdzY*solid.FdzZ | 1 | Cauchy–Green tensor, YZ-component | Domain 3 |  |
| solid.CZZ | solid.FdxZ^2+solid.FdyZ^2+solid.FdzZ^2 | 1 | Cauchy–Green tensor, ZZ-component | Domain 3 |  |
| solid.Cl11 | 1+2*solid.el11 | 1 | Cauchy–Green tensor, local coordinate system, 11-component | Domain 3 |  |
| solid.Cl12 | 2*solid.el12 | 1 | Cauchy–Green tensor, local coordinate system, 12-component | Domain 3 |  |
| solid.Cl13 | 2*solid.el13 | 1 | Cauchy–Green tensor, local coordinate system, 13-component | Domain 3 |  |
| solid.Cl22 | 1+2*solid.el22 | 1 | Cauchy–Green tensor, local coordinate system, 22-component | Domain 3 |  |
| solid.Cl23 | 2*solid.el23 | 1 | Cauchy–Green tensor, local coordinate system, 23-component | Domain 3 |  |
| solid.Cl33 | 1+2*solid.el33 | 1 | Cauchy–Green tensor, local coordinate system, 33-component | Domain 3 |  |
| solid.Cel11 | solid.FiilCl11*solid.Fiil11+solid.FiilCl12*solid.Fiil21+solid.FiilCl13*solid.Fiil31 | 1 | Elastic Cauchy–Green tensor, local coordinate system, 11-component | Domain 3 |  |
| solid.Cel12 | solid.FiilCl11*solid.Fiil12+solid.FiilCl12*solid.Fiil22+solid.FiilCl13*solid.Fiil32 | 1 | Elastic Cauchy–Green tensor, local coordinate system, 12-component | Domain 3 |  |
| solid.Cel13 | solid.FiilCl11*solid.Fiil13+solid.FiilCl12*solid.Fiil23+solid.FiilCl13*solid.Fiil33 | 1 | Elastic Cauchy–Green tensor, local coordinate system, 13-component | Domain 3 |  |
| solid.Cel22 | solid.FiilCl21*solid.Fiil12+solid.FiilCl22*solid.Fiil22+solid.FiilCl23*solid.Fiil32 | 1 | Elastic Cauchy–Green tensor, local coordinate system, 22-component | Domain 3 |  |
| solid.Cel23 | solid.FiilCl21*solid.Fiil13+solid.FiilCl22*solid.Fiil23+solid.FiilCl23*solid.Fiil33 | 1 | Elastic Cauchy–Green tensor, local coordinate system, 23-component | Domain 3 |  |
| solid.Cel33 | solid.FiilCl31*solid.Fiil13+solid.FiilCl32*solid.Fiil23+solid.FiilCl33*solid.Fiil33 | 1 | Elastic Cauchy–Green tensor, local coordinate system, 33-component | Domain 3 |  |
| solid.Ceil11 | (solid.Cel22*solid.Cel33-solid.Cel23^2)/(solid.Cel11*solid.Cel22*solid.Cel33+2*solid.Cel12*solid.Cel23*solid.Cel13-solid.Cel11*solid.Cel23^2-solid.Cel12^2*solid.Cel33-solid.Cel22*solid.Cel13^2) | 1 | Elastic Cauchy–Green tensor inverse, local coordinate system, 11-component | Domain 3 |  |
| solid.Ceil12 | (solid.Cel23*solid.Cel13-solid.Cel12*solid.Cel33)/(solid.Cel11*solid.Cel22*solid.Cel33+2*solid.Cel12*solid.Cel23*solid.Cel13-solid.Cel11*solid.Cel23^2-solid.Cel12^2*solid.Cel33-solid.Cel22*solid.Cel13^2) | 1 | Elastic Cauchy–Green tensor inverse, local coordinate system, 12-component | Domain 3 |  |
| solid.Ceil13 | (solid.Cel12*solid.Cel23-solid.Cel22*solid.Cel13)/(solid.Cel11*solid.Cel22*solid.Cel33+2*solid.Cel12*solid.Cel23*solid.Cel13-solid.Cel11*solid.Cel23^2-solid.Cel12^2*solid.Cel33-solid.Cel22*solid.Cel13^2) | 1 | Elastic Cauchy–Green tensor inverse, local coordinate system, 13-component | Domain 3 |  |
| solid.Ceil22 | (solid.Cel11*solid.Cel33-solid.Cel13^2)/(solid.Cel11*solid.Cel22*solid.Cel33+2*solid.Cel12*solid.Cel23*solid.Cel13-solid.Cel11*solid.Cel23^2-solid.Cel12^2*solid.Cel33-solid.Cel22*solid.Cel13^2) | 1 | Elastic Cauchy–Green tensor inverse, local coordinate system, 22-component | Domain 3 |  |
| solid.Ceil23 | (solid.Cel12*solid.Cel13-solid.Cel11*solid.Cel23)/(solid.Cel11*solid.Cel22*solid.Cel33+2*solid.Cel12*solid.Cel23*solid.Cel13-solid.Cel11*solid.Cel23^2-solid.Cel12^2*solid.Cel33-solid.Cel22*solid.Cel13^2) | 1 | Elastic Cauchy–Green tensor inverse, local coordinate system, 23-component | Domain 3 |  |
| solid.Ceil33 | (solid.Cel11*solid.Cel22-solid.Cel12^2)/(solid.Cel11*solid.Cel22*solid.Cel33+2*solid.Cel12*solid.Cel23*solid.Cel13-solid.Cel11*solid.Cel23^2-solid.Cel12^2*solid.Cel33-solid.Cel22*solid.Cel13^2) | 1 | Elastic Cauchy–Green tensor inverse, local coordinate system, 33-component | Domain 3 |  |
| solid.eXX | solid.gradUxX | 1 | Strain tensor, XX-component | Domain 3 | + operation |
| solid.eXY | 0.5*(solid.gradUxY+solid.gradUyX) | 1 | Strain tensor, XY-component | Domain 3 | + operation |
| solid.eXZ | 0.5*(solid.gradUxZ+solid.gradUzX) | 1 | Strain tensor, XZ-component | Domain 3 | + operation |
| solid.eYY | solid.gradUyY | 1 | Strain tensor, YY-component | Domain 3 | + operation |
| solid.eYZ | 0.5*(solid.gradUyZ+solid.gradUzY) | 1 | Strain tensor, YZ-component | Domain 3 | + operation |
| solid.eZZ | solid.gradUzZ | 1 | Strain tensor, ZZ-component | Domain 3 | + operation |
| solid.el11 | (solid.sysT11*solid.eXX+solid.sysT12*solid.eXY+solid.sysT13*solid.eXZ)*solid.sysT11+(solid.sysT11*solid.eXY+solid.sysT12*solid.eYY+solid.sysT13*solid.eYZ)*solid.sysT12+(solid.sysT11*solid.eXZ+solid.sysT12*solid.eYZ+solid.sysT13*solid.eZZ)*solid.sysT13 | 1 | Strain tensor, local coordinate system, 11-component | Domain 3 | + operation |
[truncated: 1,154,224 more chars]
